# Exploring Causal Links Between 91 Circulating Inflammatory Proteins and Hashimoto’s Thyroiditis: A Bidirectional Mendelian Randomization Study

**DOI:** 10.1155/mi/6651720

**Published:** 2026-09-07

**Authors:** Shuting Ding, Weiqiang Su, Minghao Wu, Min Fu, Meng Wang, Hongzhi Ji, Bin Yang, Zhen Zhang, Chenyu Ma, Jiani Pan, Xue Di, Xin Zhang, Xiaohua Zhao, Xiaodong Sun, Yujia Kong

**Affiliations:** ^1^ School of Public Health, Shandong Second Medical University, Weifang, China; ^2^ Department of Pharmacology, The First Affiliated Hospital of Shandong Second Medical University, Weifang, China; ^3^ Department of Hepatobiliary, The Affiliated Hospital of Shandong Second Medical University, Weifang, China; ^4^ Department of Respiratory, The Affiliated Hospital of Shandong Second Medical University, Weifang, China; ^5^ Department of Imaging, Baoji Maternal and Child Health Hospital, Baoji, China; ^6^ Department of Endocrinology, The Affiliated Hospital of Shandong Second Medical University, Weifang, China; ^7^ Department of Oncology, The Affiliated Hospital of Shandong Second Medical University, Weifang, China

**Keywords:** causal inference, Hashimoto’s thyroiditis, inflammation, inflammatory proteins, Mendelian randomization

## Abstract

**Background:**

Increasing evidence has linked inflammation to Hashimoto’s thyroiditis (HT) etiology. However, the causal role of circulating inflammatory proteins in HT remains uncertain. To investigate this, we conducted a bidirectional Mendelian randomization (MR) study.

**Methods:**

Genetic data for 91 inflammatory proteins and HT were sourced from publicly available GWAS databases. The PhenoScanner database was then searched for pleiotropic SNPs associated with potential confounders. Inverse variance weighted (IVW) analysis was used as the primary analysis, simultaneously supplemented by five sensitivity analyses to strengthen the results.

**Results:**

The results revealed that, after false discovery rate (FDR) correction, interleukin (IL)‐12p40 was causally associated with increased risk of HT (OR [95% CI] = 1.295 [1.172, 1.431], *p* = 3.66 × 10^−7^, *P*_FDR_ = 3.33×10^−5^). Conversely, none of the inflammatory proteins was a consequence of HT.

**Conclusion:**

This study suggests that IL‐12p40 is probably one of the factors correlated with HT etiology, contributing to a better understanding of the pathogenesis of HT and underscoring the potential for therapeutic interventions targeting inflammatory proteins.

## 1. Introduction

Hashimoto’s thyroiditis (HT), also known as autoimmune thyroiditis or Hashimoto’s disease, is a prevalent autoimmune condition [1]. It is marked by elevated levels of autoantibodies, including thyroglobulin antibodies (TgAbs) and thyroid peroxidase antibodies (TPOAbs), which serve as key markers for lymphocytic infiltration and subsequent thyroid tissue destruction [2].

HT results from a complex interplay of genetic, epigenetic, and environmental factors [3]. Both cellular immune responses and humoral responses are integral to the pathogenesis of the disease. However, the mechanisms underlying the extensive lymphocyte infiltration and thyroid cell destruction in HT patients remain poorly understood. HT is thought to involve inflammatory immune responses in which thyroid‐specific T cells target thyroglobulin [4]. Th1 and TH17 cells contribute to thyroiditis development by inducing apoptotic cell death in the thyroid tissue and promoting inflammation and damage through the production of inflammatory proteins [5, 6]. Proteins involved in inflammation that contribute to disease progression have the capacity to modulate immune responses and directly influence thyroid follicular cells (TFCs) [7, 8].

Previous epidemiological research has reported associations between certain inflammatory proteins and HT. Interleukin (IL)‐37 shows increased expression in HT and may play a protective role by reducing oxidative stress and inflammation [9]. A case–control study indicated that patients with HT had elevated levels of IL‐12 and IL‐1B^7^. Additionally, another study highlighted the crucial involvement of the IL‐23/IL‐17 pathway and Th17 cells in the development of autoimmune thyroid diseases [10].

Observational studies examining inflammatory proteins in relation to HT are susceptible to confounding and reverse causation. Mendelian randomization (MR) overcomes these issues by using genetic variants as instrumental variables, offering a robust method for causal inference when randomized trials are impractical [11] Therefore, MR provides a more robust framework for causal inference. Previous MR studies have identified significant causal associations between proteins related to lymphocyte function and hypothyroidism risk, as well as between inflammatory proteins (e.g., IL‐12p70, IL‐7, and MIP‐1β) and autoimmune thyroid disease and hypothyroidism [12–14]. These findings indicated that immune and inflammatory responses play crucial roles in the development of these thyroid diseases. A recent study using genome‐wide protein quantitative trait locus (pQTL) analysis discovered genetic variants linked to 91 inflammatory proteins [15]. Subsequent studies have incorporated the GWAS data of these proteins into MR analyses, providing more comprehensive insights into the mechanistic involvement of these proteins in disease [16]. To evaluate the potential causal links between 91 inflammatory proteins and HT, a bidirectional MR study was performed, utilizing GWAS summary statistics for inflammatory proteins and HT.

## 2. Methods

### 2.1. Study Design and Data Sources

**Figure 1:**
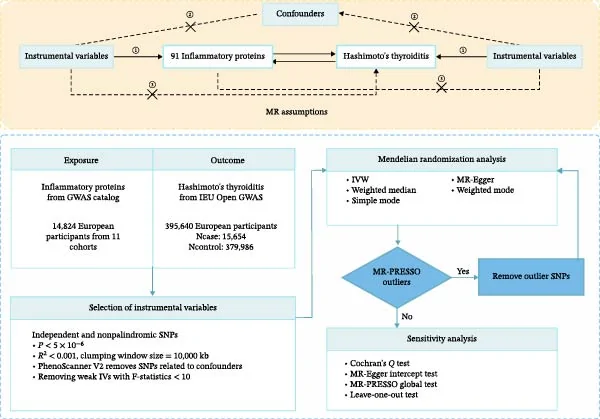
Three basic assumptions must be satisfied by the IVs selected. Datasets and MR design for exploring the associations between 91 inflammatory proteins and HT. Abbreviations: GWAS, Genome‐Wide Association study; HT, Hashimoto’s thyroiditis; IV, instrumental variable; IVW, inverse‐variance weighted; MR, Mendelian randomization; MR‐PRESSO, Mendelian randomization pleiotropy residual sum and outlier; SNP, single‐nucleotide polymorphism.

Figure 1 provides a concise overview of the bidirectional MR design employed in this study. Inflammatory proteins and HT‐related SNPs were obtained from two separate samples. The 91 inflammatory proteins were obtained from summary statistics data of the large‐scale GWAS meta‐analysis [15]. This research included a total of 14,824 individuals of European descent from 11 cohort studies. The data for HT are sourced from the IEU Open GWAS project (available at https://gwas.mrcieu.ac.uk/) [17], where HT cases were defined based on clinical diagnosis using ICD‐10 (E06.3) and ICD‐9 (245.21). This dataset comprises participants of European ancestry, including 15,654 cases and 379,986 controls.

We employed a two‐sample MR approach to infer causality and considered 91 proteins serving as the exposure and HT as the outcome. For the primary analyses, we selected genetic variants for 91 proteins to test potential causal effects from each inflammatory protein to HT. For the secondary analyses, genetic variants associated with HT were exploited to test potential causal effects from HT on inflammatory proteins.

MR relies on three core IV assumptions: (1) these SNPs must be significantly associated with the exposure (inflammatory proteins); (2) these SNPs are independent of the outcome (HT); and (3) these SNPs can only exert an effect through the inflammatory proteins to HT [18].

### 2.2. Selection of Instrumental Variables

First, for inflammatory proteins, since very few SNPs reached the standard genome‐wide significance level (*p* < 5 × 10^−8^), a more relaxed threshold of *p*  < 5 × 10^−6^ was applied to obtain a more comprehensive and reliable result. Second, the clustering program in PLINK (v1.90) was employed to prune these SNPs, using a linkage disequilibrium (LD) *r* ^2^ threshold of less than 0.001 and within a 10000 kb distance. Third, SNPs were searched and obtained via PhenoScanner V2 (http://www.phenoscanner.medschl.cam.ac.uk/, accessed on December 20, 2023) to detect potential confounders [19]. For each SNP and its proxy (*r* ^2^ > 0.80), we systematically investigated any previously reported associations (*p* < 5 × 10^−6^) based on relevant published studies. Three potential confounding factors were identified and appropriately controlled for: allergic disease [20], B‐cell lymphoma [21], and rheumatoid arthritis [22]. Four SNPs (rs705705, rs186975: allergic disease, rs72993671: B‐cell lymphoma, and rs181816009: rheumatoid arthritis) associated with these potential confounding factors were identified and eliminated from the analysis. Lastly, the *F* statistic was used to evaluate the relationship between SNPs and exposure; if *F* < 10, the correlation between SNPs and exposure is considered weak [23]. SNPs with a palindromic structure were automatically excluded during the analysis.

### 2.3. Bidirectional MR Analysis

Inverse variance weighted (IVW) was used as the main analysis method to examine whether there was a causal association between inflammatory proteins and HT. Weighted median, MR‐Egger regression, weighted mode, simple mode, and MR pleiotropy residual sum and outlier (MR‐PRESSO) were used as supplementary methods. In the main analysis, we generated MR estimates for each individual SNP using the Wald ratio and approximated the standard error using the delta method. Subsequently, we employed a random‐effects inverse‐variance weighting meta‐analysis approach to combine individual MR estimates. Moreover, the aim is to mitigate the risk of Type I errors resulting from a relaxed threshold requirement and to assess the impact of multiple testing on research findings. The Benjamini–Hochberg method was used to control the false discovery rate (FDR), *p*  < 0.05 but *P*_FDR_ > 0.05 was regarded as indicative of a possible association. In addition, our study also employed the weighted median, MR‐Egger, simple mode, and weighted mode as supplementary approaches. Due to the limited statistical power of MR‐Egger, our focus was on assessing the consistency of the estimate directions rather than their significance.

To ensure the stability and reliability of the results, our study employed a series of comprehensive methods for sensitivity analysis. The MR‐Egger intercept method was used to assess potential directional pleiotropy [24]. Additionally, MR‐PRESSO was employed to further ensure that the study results were not influenced by confounding factors [25]. Furthermore, a leave‐one‐out sensitivity analysis was conducted to meticulously evaluate the impact of each IV [26]. In assessing the heterogeneity among SNPs, Cochran’s *Q* statistic was chosen as the tool for heterogeneity evaluation [27]. If the data indicated no significant heterogeneity (*p* > 0.05), a fixed‐effects model was applied for the MR analysis; conversely, a random‐effects model was used to assess the potential causal relationship between proteins and HT. MR analyses were conducted in R (Version 4.2.3) using the MendelianRandomization (Version 0.9.0) and TwoSampleMR (Version 0.5.7) packages. All data utilized in this study were derived from research that had obtained participant consent.

## 3. Results

### 3.1. Primary Analyses

**Figure 2:**
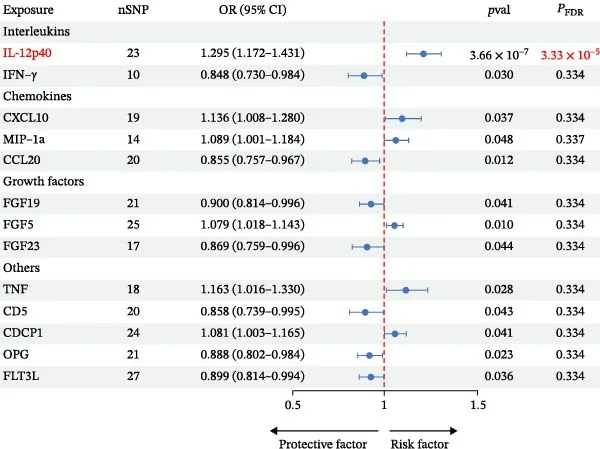
Causal correlations of 13 inflammatory proteins on HT. nSNP: the final number of MR analysis of 13 circulating inflammatory proteins on HT. Abbreviations: CCL20, C–C motif chemokine ligand 20; CD5, cluster of differentiation 5; CDCP1, CUB domain‐containing protein 1; CI, confidence interval; CXCL10, C–X–C motif chemokine ligand 10; FGF19, fibroblast growth factor 19; FGF23, fibroblast growth factor 23; FGF5, fibroblast growth factor 5; FLT3L, Fms‐related tyrosine kinase 3 ligand; IFN‐γ, interferon‐gamma; IL‐12p40, interleukin‐12p40; MIP‐1α, macrophage inflammatory protein‐1 alpha; nSNP, number of single‐nucleotide polymorphisms; OPG, osteoprotegerin; OR, odds ratio; *P*_FDR_, false discovery rate‐adjusted *p* value; *p*val, *p* value; TNF, tumor necrosis factor.

**Figure 3:**
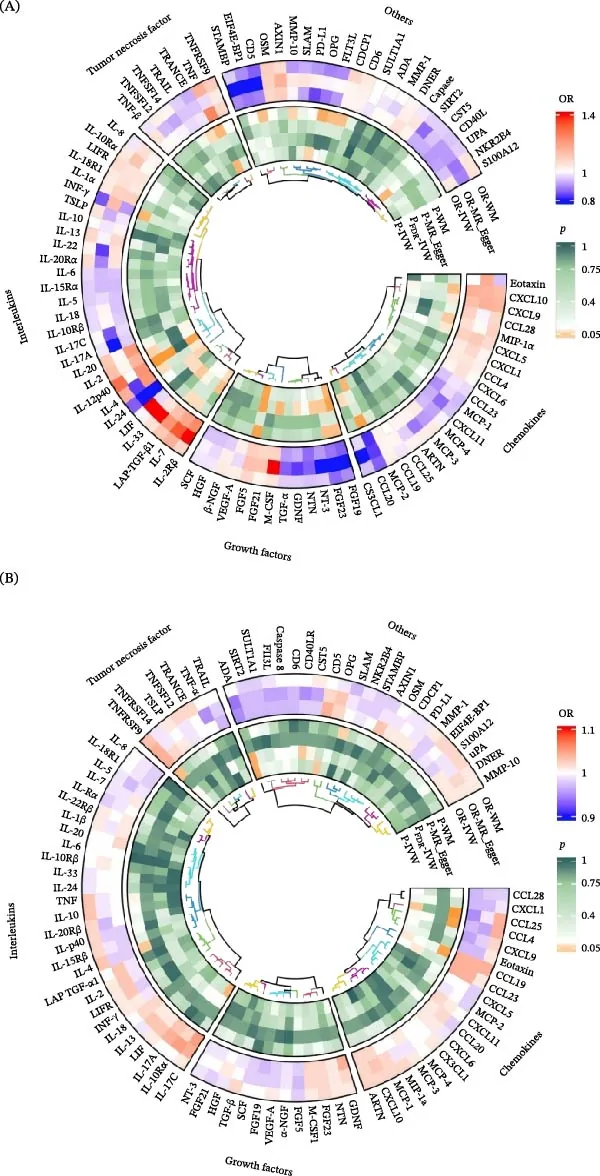
(A) Ring heatmap showing the MR analysis of the causal relationship between inflammatory proteins and HT. (B) Ring heatmap showing the MR analysis of the causal relationship between HT and inflammatory proteins. Outer and inner rings represent ORs and *p* values, respectively. Abbreviations: OR, odds ratio; *p*, *p* value.

A slightly relaxed cutoff (*p* < 5 × 10^−6^) involved all 91 inflammatory proteins, with *F*‐statistics ranging between 22.22 and 131.91 (Supporting Information 1: Table S1). To evaluate the influence of 91 inflammatory proteins on HT, IVW served as the primary method [28]. Figure 2 shows IVW analytical findings for these 13 inflammatory proteins, the MR analyses between the 91 inflammatory proteins and the HT can be found in Figure 3A and Supporting Information 1: Table S3. Both the exposure and outcome GWAS datasets were derived from individuals of European ancestry and originated from independent cohorts, which reduces the likelihood of population heterogeneity and potential sample overlap.

Of the 91 inflammatory proteins, 13 relevant inflammatory proteins that reached a nominal significance (*p* < 0.05) were screened from the primary analysis, including IL‐12 subunit beta (IL‐12p40), FGF family members (FGF5, FGF19, and FGF23), chemokine regulators (CCL20, CXCL10, and MIP‐1α), osteoprotegerin (OPG), tumor necrosis factor (TNF), interferon‐gamma (IFN‐γ), fms‐related tyrosine kinase 3 ligand (FLT3L), CUB domain‐containing protein 1 (CDCP1), T‐cell surface glycoprotein CD5 (CD5).

After FDR correction, only genetically predicted IL‐12p40 was significantly associated with increased HT risk (IVW: OR [95% CI] = 1.295 [1.172, 1.431], *p* = 3.66 × 10^−7^, *P*_FDR_ = 3.33 × 10^−5^). Although the results of other MR analysis methods did not reach statistical significance, they showed consistent trends or directions with the IVW analysis results (MR‐Egger: OR [95% CI] = 1.012 [0.758−1.351], *p* = 0.935; weighted median: OR [95% CI] = 1.242 [1.059−1.457], *p* = 0.008; weighted mode: OR [95% CI] = 1.084 [0.819−1.433], *p* = 0.579; simple mode: OR [95% CI] = 1.097 [0.794−1.517], *p* = 0.581). *Q* statistics (*p* = 0.141) showed no statistically significant differences, suggesting no heterogeneity (Supporting Information 1: Table S3). The graphical representation of sensitivity analysis can be found in the Graphic Abstract. After applying the MR‐PRESSO outlier test to remove outlier SNPs (outliers: rs10043720), both the MR‐PRESSO global test (*p* = 0.082) and the MR‐Egger intercept analysis (intercept = 0.026, *p* = 0.082) revealed no significant horizontal pleiotropy. For horizontal pleiotropy, the “leave‐one‐out” analysis demonstrated the robustness of our MR analysis as it was not affected by any individual SNP. No invalid SNPs were detected using the MR‐Steiger filtering method.

### 3.2. Secondary Analyses

Using 91 inflammatory proteins as the outcome to investigate the possibility of reverse causation, we thoroughly examined 46 SNPs that showed strong associations with HT, using a threshold of *p*  < 5 × 10^−6^. Genetic instrument strength was confirmed by *F*‐statistics exceeding the empirical threshold of 10 (range: 32.82−38.58), demonstrating methodological robustness against weak instrument bias. Comprehensive SNP characteristics are presented in Supporting Information 1: Table S2, while the effect of HT on 91 inflammatory proteins is shown in Figure 3B and Supporting Information 1: Table S4.

**Figure 4:**
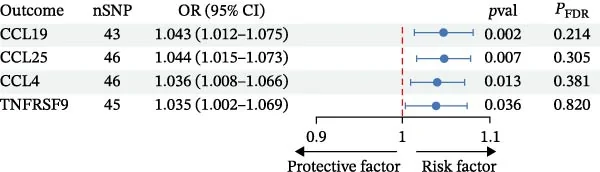
Causal correlations of HT on 4 inflammatory proteins. Abbreviations: CCL19, C–C motif chemokine ligand 19; CCL25, C–C motif chemokine ligand 25; CCL4, C–C motif chemokine ligand 4; CI, confidence interval; nSNP, number of single‐nucleotide polymorphisms; OR, odds ratio; *P*_FDR_, false discovery rate‐adjusted *p* value; *p*val, *p* value; TNFRSF9, tumor necrosis factor receptor superfamily member 9.

Subsequently, the IVW analysis identified four inflammatory casually linked to HT (*p* < 0.05; Figure 4), including CCL19, CCL25, CCL4, and TNFRSF9. For the analyses involving CCL19 and TNFRSF9, 3 and 1 SNPs were not present in the outcome GWAS, respectively, and no proxy SNPs were available, leaving 43 and 45 SNPs for analysis. After FDR correction, no inflammatory proteins were observed as a downstream consequence of HT. The MR‐Egger intercept test provided no evidence of potential pleiotropy (all *p*  > 0.05; Supporting Information 1: Table S4), while MR‐PRESSO showed the presence of pleiotropic SNPs in the GWAS dataset for TNFRSF9 (*p* = 0.043, Supporting Information 1: Table S4). The results of the sensitivity analysis can be found in Supporting Information 1: Table S4.

## 4. Discussion

In this study, we performed MR analyses to explore the association between 91 inflammatory proteins and the risk of HT. Our findings indicate a genetically predicted association between IL‐12p40 and HT, providing evidence for a potential causal role of IL‐12p40 in the HT etiology. Before FDR correction, MR analyses identified 12 inflammatory proteins associated with the HT risk. In the reverse‐direction analysis, HT was associated with four inflammatory proteins. However, after FDR correction, no causal relationship was identified between these 16 inflammatory proteins and HT. Notably, MR primarily informs etiological relevance, whereas the therapeutic implications of targeting this pathway warrant further investigation.

Numerous inflammatory proteins originating from infiltrating lymphocytes have been implicated in the pathogenesis of HT [29]. One study identified significant causal associations between proteins associated with lymphocyte function and the risk of hypothyroidism [12]. Moreover, two other studies found causal associations between several inflammatory proteins, including IL‐12p70, IL‐7, and MIP‐1β, and both autoimmune thyroid disease and hypothyroidism [13, 14]. Additionally, HT may manifest Th1 and Th17 immune responses, promoting cell‐mediated immunity and triggering the apoptosis‐driven destruction of TFCs. The inflammatory proteins produced in these responses play a nonnegligible role [5, 6]. These findings indicate that proteins associated with immune and inflammatory responses may serve as essential contributors to the onset and progression of HT.

IL‐12p40 (also referred to as IL‐12β) is a shared subunit of IL‐12 and IL‐23 but is believed to exert distinct biological functions [30]. Activated inflammatory immune cells produce IL‐12p40, which performs intricate and diverse functions in various inflammatory microenvironments [31]. Several research findings propose a potential correlation between IL‐12p40 and HT. Previous experimental research has demonstrated a notable elevation in plasma levels of IL‐12p40 and IL‐12p70 in the study group relative to the control group [32]. Patients with IL‐12p40 deficiency exhibit undetectable serum concentrations of both IL‐12p40 and IL‐12p70. Furthermore, animal studies indicate that mice lacking IL‐12p40 or IL‐18 experience a transition from a Th1 to a Th2 cytokine response, along with a decline in cytotoxic natural killer (NK) cells and cytotoxic T‐lymphocytes (CTLs) [33]. Mice deficient in IL‐12p40 exhibit resistance to disease in various preclinical models of chronic inflammation and autoimmunity [34, 35]. A recent study indicated that IL‐12p40 potentially exerts an enhancing effect on Th17 cell differentiation while promoting the polarization of proinflammatory macrophages in murine models [36]. Th17 cells, a subpopulation of proinflammatory T‐lymphocytes identified subsequent to TH1 and TH2 cells, are known to play a significant role in immunoinflammatory processes [37]. Usually, patients with HT have higher levels of TgAb [38]. Studies have shown that the IL‐12p40 concentration in unstimulated whole saliva is positively correlated with the plasma TgAb concentration [32]. These findings support our study. However, it is important to note that there is heterogeneity in the findings across various studies. Ikeda et al. reported no statistically significant correlation between IL‐12B gene polymorphisms and thyroiditis [39]. These differences might stem from study design variations, sample size constraints, and population heterogeneity. Moreover, HT is a multifactorial disease shaped by various genetic and environmental influences. The collective impact of polymorphisms in inflammatory proteins may have a greater influence on the susceptibility and severity of HT, while IL‐12B gene polymorphisms may constitute only a fraction of the overall mechanism [40]. Hence, additional research is necessary to further explore the contentious nature of the relationship between IL‐12B gene polymorphisms and thyroiditis.

IL‐12 is an inflammatory protein composed of two subunits, the p40 subunit and the p35 subunit [41]. Notably, the absence of the p40 subunit leads to the complete lack of both IL‐12 and IL‐23. Studies have shown that serum IL‐12 levels are elevated in patients with HT when compared to individuals with toxic nodular goiter, Graves’ disease (GD), or healthy controls [6]. A separate study utilized IL‐12 transgenic mouse models to investigate the role of IL‐12 in the pathogenesis of HT and its associated immune mechanisms, revealing that the presence of IL‐12 in the thyroid leads to thyroiditis [42]. IL‐12p40 combines with IL‐12p35 to form bioactive IL‐12, which can induce CD4+ T‐lymphocytes to differentiate from the Th0 to the Th1 phenotype [43]. The presence of IL‐12 can also activate immune cells, including NKT cells, NK cells, and T cells. The activation of these immune cells may lead to enhanced inflammation and cell‐mediated immune responses, further damaging the TFCs. While we have understood that IL‐12 may play a detrimental role in HT, the specific mechanisms and their sequential order still require further in‐depth research to ascertain.

IL‐23 consists of a distinct p19 subunit combined with the p40 subunit of IL‐12 [44]. Although the measurement of the p40 chain is often interpreted as a measurement of the complete p75 heterodimer, detecting p40 is only a measurement of one subunit of IL‐12. Therefore, if p40 is detected, it implies the detection of both IL‐12 and IL‐23 simultaneously. Antigen‐producing cells, including macrophages and dendritic cells, produce IL‐23, which plays a pivotal role in the differentiation of pathogenic Th17 lymphocytes [44]. Previous research suggests that a higher Th17 cell count in the blood of HT patients is positively associated with structural damage to the thyroid. IL‐23 drives HT pathogenesis by enhancing Th17 cell formation and inducing the release of IL‐17 [45, 46].

In addition, the accumulation of abnormal reactive oxygen species (ROS) is recognized as a detrimental factor contributing to the development of HT, and autophagy plays a crucial role in mitigating cellular damage and preventing cell death by serving as a significant mechanism for the removal of excessive ROS [47, 48]. Research by Zheng et al. demonstrated heightened IL‐23 production within TFCs obtained from HT patients and confirmed that IL‐23 can induce ROS accumulation and autophagy inhibition in TFCs [49]. A recent study revealed a noteworthy elevation in serum IL‐23 levels in patients diagnosed with HT when compared to both GD patients and healthy individuals [50]. The pathogenesis and pathophysiology of HT involve multiple complex processes, of which immune‐mediated inflammation represents only one component. It is worth noting that whether the potential role of IL‐23 in HT is associated with IL‐12p40 remains to be further explored.

Interestingly, our study suggests a possible link between IFN‐γ and a decreased risk of HT. TH1‐ and TH17‐mediated cell immune responses have been implicated in HT development. IFN‐γ, produced by TH1 cells, stimulates antigen‐presenting cells and enhances TH1 cell differentiation by upregulating the transcription factor T‐bet [51]. However, it impedes the initial differentiation of T cells into TH17 subsets [52]. Notably, studies have demonstrated that a deficiency in IFN‐γ can worsen autoimmune inflammation, as observed in experimental autoimmune encephalomyelitis. Hence, further investigation is required to uncover the mechanisms involved and investigate the therapeutic potential of IFN‐γ in HT and related autoimmune inflammatory disorders.

This study systematically investigated the associations between HT and 91 circulating inflammatory proteins using MR. Our findings are supported by prior literature that has examined both upstream and downstream inflammatory proteins in HT. Clinical trials of treating immune disorders by targeting IL‐12p40 have provided satisfactory therapeutic results [53]. The treatment for HT has remained largely unchanged over the past few decades. Patients may need the option of long‐term treatment with lifelong thyroid hormone replacement therapy if they have hypothyroidism. Future studies should aim to better understand the pathogenesis of HT in order to obtain safe, conservative, and definitive treatments that target the cause. This study adhered to MR procedures, with efforts made to minimize potential sample overlap and enhance the reliability of the findings. Our findings offer further insights into the inflammatory mechanisms underlying HT and may inform future studies on therapeutic target development.

However, this study has several limitations. First, due to the limitations of MR analysis, it may not fully address the second and third hypotheses, potentially introducing bias. Second, further experimental studies and well‐designed clinical trials in patients with HT are required to validate these findings and assess their potential clinical relevance. Third, this study was limited to participants of European ancestry, which may restrict the generalizability of our findings to populations of other ethnic backgrounds. Fourth, a relaxed SNP selection threshold (*p* < 5 × 10^−6^) was used to identify instrumental variables, which may increase the risk of false‐positive findings. Therefore, the results should be interpreted with caution. Fifth, the limited number of SNPs and the fact that some confounders are unknown make it possible for unmeasured confounders to bias the results, potentially affecting both HT and inflammatory protein levels. Finally, the HT phenotype may be clinically heterogeneous, encompassing euthyroid HT, overt hypothyroidism, and antibody‐positive individuals. Such phenotype misclassification could attenuate the associations between genetic instruments and the true disease status, potentially biasing MR estimates toward the null. Future studies incorporating more precise phenotyping, including antibody titers, thyroid function assessments, or ultrasound features, are warranted to enable stratified analyses and reveal more specific causal relationships.

## 5. Conclusion

In conclusion, we conducted a bidirectional MR study to explore the associations of 91 inflammatory proteins with the risk of HT. The results suggest that genetically determined IL‐12p40 levels may contribute to a higher risk of HT. Further investigation is necessary to validate these associations and determine the potential of these proteins as therapeutic or preventive targets for HT.

## Author Contributions

**Shuting Ding**: conceptualization, methodology, formal analysis, writing – original draft, writing – review and editing. **Weiqiang Su**: methodology, writing – original draft. **Minghao Wu and Bin Yang:** writing – review and editing. **Min Fu**, **Meng Wang, and Hongzhi Ji**: data curation, writing – review and editing. **Zhen Zhang**: formal analysis. **Chenyu Ma and Jiani Pan**: conceptualization, supervision. **Xue Di and Xin Zhang**: formal analysis, visualization. **Xiaohua Zhao**: supervision, writing – review and editing. **Xiaodong Sun and Yujia Kong**: conceptualization, supervision, writing – review and editing, funding acquisition.

## Funding

This work was supported by the National Natural Science Foundation of China (Grant 82003560 to Yujia Kong and Grant 82170865 to Xiaodong Sun), the National Natural Science Foundation of Shandong (Grant ZR2020MH340 to Yujia Kong), and the Science and Technology Program of Shaanxi Province (Grant 2024SF‐YBXM‐256 to Bin Yang).

## Ethics Statement

This study utilized previously published research and collaborations that provided publicly accessible summary statistics. Individual‐level data were not employed in this investigation, thereby obviating the need for a new ethical review board approval.

## Conflicts of Interest

The authors declare no conflicts of interest.

## Supporting Information

Additional supporting information can be found online in the Supporting Information section.

## Supporting information


**Supporting Information 1** Table S1: Details of systemic inflammatory proteins predicting SNPs with Hashimoto’s Thyroiditis from GWAS (with SNPs reaching *p*  < 5 × 10^−6^). Table S2: Details of Hashimoto’s thyroiditis predicting SNPs with inflammatory proteins from IEU Open GWAS (with SNPs reaching *p*  < 5 × 10^−6^). Table S3: Associations of inflammatory proteins with Hashimoto’s Thyroiditis from GWAS using Mendelian randomization (with SNPs reaching *p*  < 5 × 10^−6^). Table S4: Associations of Hashimoto’s thyroiditis with inflammatory proteins from IEU Open GWAS using Mendelian randomization (with SNPs reaching *p*  < 5 × 10^−6^).

**Supporting Information 2** STROBE‐MR checklist of recommended items to address in reports of Mendelian randomization studies.

## Data Availability

The data for 91 inflammatory proteins were derived from the GWAS Catalog (https://www.ebi.ac.uk/gwas/). The data for HT were derived from the IEU Open GWAS (https://gwas.mrcieu.ac.uk/).
