# Supplementary material for: Exploring Causal Links Between 91 Circulating Inflammatory Proteins and Hashimoto’s Thyroiditis: A Bidirectional Mendelian Randomization Study
Source: Mediators Inflamm. 2026 Sep 7;2026:6651720. doi: 10.1155/mi/6651720 (PMC13550872; doi:10.1155/mi/6651720)
Supplement: Supplementary file 1 [file MI-2026-6651720-s001.pdf]

STROBE-MR checklist of recommended items to address in reports of Mendelian randomization studies[1 2]

Item No.	Section	Checklist item	Page No.	Relevant text from manuscript
1	**TITLE and ABSTRACT**	Indicate Mendelian randomization (MR) as the study's design in the title and/or the abstract if that is a main purpose of the study	Page 1, Lines 1–2;<br><br>Page 2, Lines 27–36	Exploring Causal Links between 91 Circulating Inflammatory Proteins and Hashimoto's Thyroiditis: a Bidirectional Mendelian Randomization Study.<br><br>Background: Increasing evidence has linked inflammation to Hashimoto's thyroiditis (HT) etiology. However, the causal role of circulating inflammatory proteins in HT remains uncertain. To investigate this, we conducted a bidirectional Mendelian randomization (MR) study. Methods: Genetic data for 91 inflammatory proteins and HT were sourced from publicly available GWAS databases. The PhenoScanner database was then searched for pleiotropic SNPs associated with potential confounders. Inverse variance weighted (IVW) analysis was used as the primary analysis, simultaneously supplemented by five sensitivity analyses to strengthen the results.
	**INTRODUCTION**			
2	**Background**	Explain the scientific background and rationale for the reported study. What is the exposure? Is a potential causal relationship between exposure and outcome plausible? Justify why MR is a helpful method to address the study question	Page 3-4, Lines 48–85	Hashimoto's thyroiditis (HT), also known as autoimmune thyroiditis or Hashimoto's disease, is a prevalent autoimmune condition1. It is marked by elevated levels of autoantibodies, including thyroglobulin antibodies (TgAb) and thyroid peroxidase antibodies (TPOAb), which serve as key markers for lymphocytic infiltration and subsequent thyroid tissue destruction 2.<br><br>HT results from a complex interplay of genetic, epigenetic, and environmental factors3. Both cellular immune responses and humoral responses are integral to the pathogenesis of the disease. However, the mechanisms underlying the extensive lymphocyte infiltration and thyroid cell destruction in HT patients remain poorly understood. HT is thought to involve inflammatory immune responses in which thyroid-specific T cells target thyroglobulin4. Th1 and TH17 cells contribute to thyroiditis development by inducing apoptotic cell death in thyroid tissue and promoting inflammation and damage through the production of inflammatory proteins 5, 6. Proteins involved in inflammation that contribute to disease progression have

				the capacity to modulate immune responses and directly influence thyroid follicular cells 7, 8.<br><br>Previous epidemiological research have reported associations between certain inflammatory proteins and HT. Interleukin (IL) -37 shows increased expression in HT and may play a protective role by reducing oxidative stress and inflammation9. A case-control study indicated that patients with HT had elevated levels of IL-12 and IL-1B7. Additionally, another study highlighted the crucial involvement of the IL-23/IL-17 pathway and Th17 cells in the development of autoimmune thyroid diseases10.<br><br>Observational studies examining inflammatory proteins in relation to HT are susceptible to confounding and reverse causation. Mendelian randomization (MR) overcomes these issues by using genetic variants as instrumental variables, offering a robust method for causal inference when randomized trials are impractical11 Therefore, MR provides a more robust framework for causal inference. Previous MR studies have identified significant causal associations between proteins related to lymphocyte function and hypothyroidism risk, as well as between inflammatory proteins (e.g., IL-12p70, IL-7, MIP-1β) and autoimmune thyroid disease and hypothyroidism12-14. These findings indicated that immune and inflammatory responses play crucial roles in the development of these thyroid diseases. A recent study using genome-wide protein quantitative trait locus (pQTL) analysis discovered genetic variants linked to 91 inflammatory proteins15. Subsequent studies have incorporated the GWAS data of these proteins into MR analyses, providing more comprehensive insights into the mechanistic involvement of these proteins in disease16. To evaluate the potential causal links between 91 inflammatory proteins and HT, a bidirectional MR study was performed, utilizing GWAS summary statistics for inflammatory proteins and HT.
3	**Objectives**	State specific objectives clearly, including pre-specified causal hypotheses (if any). State that MR is a method that, under specific assumptions, intends to estimate causal effects	Page 4, Lines 83–85	To evaluate the potential causal links between 91 inflammatory proteins and HT, a bidirectional MR study was performed, utilizing GWAS summary statistics for inflammatory proteins and HT.
	**METHODS**			

4	**Study design and data sources**		Present key elements of the study design early in the article. Consider including a table listing sources of data for all phases of the study. For each data source contributing to the analysis, describe the following:		
		a)	Setting: Describe the study design and the underlying population, if possible. Describe the setting, locations, and relevant dates, including periods of recruitment, exposure, follow-up, and data collection, when available.	Page 4, Lines 88–96	Figure 1 provides a concise overview of the bidirectional MR design employed in this study. Inflammatory proteins and HT-related SNPs were obtained from two separate samples. The 91 inflammatory proteins were obtained from summary statistics data of the large-scale GWAS meta-analysis[15]. This research included a total of 14,824 individuals of European descent from 11 cohort studies. The data for HT are sourced from the IEU Open GWAS project (available at https://gwas.mrcieu.ac.uk/)[17], where HT cases were defined based on clinical diagnosis using ICD-10 (E06.3) and ICD-9 (245.21). This dataset comprises participants of European ancestry, including 15,654 cases and 379,986 controls.
		b)	Participants: Give the eligibility criteria, and the sources and methods of selection of participants. Report the sample size, and whether any power or sample size calculations were carried out prior to the main analysis	Page 4, Lines 90–96	The 91 inflammatory proteins were obtained from summary statistics data of the large-scale GWAS meta-analysis[15]. This research included a total of 14,824 individuals of European descent from 11 cohort studies. The data for HT are sourced from the IEU Open GWAS project (available at https://gwas.mrcieu.ac.uk/)[17], where HT cases were defined based on clinical diagnosis using ICD-10 (E06.3) and ICD-9 (245.21). This dataset comprises participants of European ancestry, including 15,654 cases and 379,986 controls.
		c)	Describe measurement, quality control and selection of genetic variants	Page 5, Lines 107–124	2.2. Selection of instrumental variables<br>First, for inflammatory proteins, since very few SNPs reached the standard genome-wide significance level ($P < 5\times10^{-8}$), a more relaxed threshold of $P < 5\times10^{-6}$ was applied to obtain a more comprehensive and reliable result. Second, the clustering program in PLINK (v1.90) was employed to prune these SNPs, using a linkage disequilibrium (LD) r^2 threshold of less than 0.001 and within 10000 kb distance. Third, SNPs were searched and obtained via PhenoScanner V2 (http://www.phenoscanner.medschl.cam.ac.uk/, accessed on 20 Dec 2023) to detect potential confounders[19]. For each SNP and its proxy ($r^2 > 0.80$), we systematically investigated any previously reported associations ($P < 5\times10^{-6}$) based on relevant published

					studies. Three potential confounding factors were identified and appropriately controlled for: allergic disease[20], B-cell lymphoma[21] and rheumatoid arthritis[22]. Four SNPs (rs705705, rs186975: allergic disease, rs72993671: B-cell lymphoma, and rs181816009: rheumatoid arthritis) associated with these potential confounding factors were identified and eliminated from the analysis. Lastly, F statistic was used to evaluate the relationship between SNPs and exposure, if $F<10$, the correlation between SNPs and exposure is considered weak [23]. SNPs with a palindromic structure were automatically excluded during the analysis.
		d)	For each exposure, outcome, and other relevant variables, describe methods of assessment and diagnostic criteria for diseases	Page 4, Lines 92–95	The data for HT are sourced from the IEU Open GWAS project (available at https://gwas.mrcieu.ac.uk/)[17], where HT cases were defined based on clinical diagnosis usin g ICD-10 (E06.3) and ICD-9 (245.21).
		e)	Provide details of ethics committee approval and participant informed consent, if relevant	Page 6, Lines 152–153	All data utilized in this study were derived from research that had obtained participant consent.
5	**Assumptions**		Explicitly state the three core IV assumptions for the main analysis (relevance, independence and exclusion restriction) as well assumptions for any additional or sensitivity analysis	Page 4, Lines 103–1066	MR relies on three core IV assumptions: (1) these SNPs must be significantly associated with the exposure (inflammatory proteins); (2) these SNPs are independent of the outcome (HT); (3) these SNPs can only exert an effect through the inflammatory proteins to HT[18].
6	**Statistical methods: main analysis**		Describe statistical methods and statistics used		
		a)	Describe how quantitative variables were handled in the analyses (i.e., scale, units, model)	NA	This study used summary-level genetic association data from published GWAS rather than individual-level quantitative variables.
		b)	Describe how genetic variants were handled in the analyses and, if applicable, how their weights were selected	Page 5, Lines 108–124	First, for inflammatory proteins, since very few SNPs reached the standard genome-wide significance level ($P < 5\times10^{-8}$), a more relaxed threshold of $P < 5\times10^{-6}$ was applied to obtain a more comprehensive and reliable result. Second, the clustering program in PLINK (v1.90) was employed to prune these SNPs, using a linkage disequilibrium (LD) r^2 threshold of less than 0.001 and within 10000 kb distance. Third, SNPs were searched and obtained via PhenoScanner V2 (http://www.phenoscanner.medschl.cam.ac.uk/,

			accessed on 20 Dec 2023) to detect potential confounders[19]. For each SNP and its proxy (r^2 > 0.80), we systematically investigated any previously reported associations ($P < 5\times10^{-6}$) based on relevant published studies. Three potential confounding factors were identified and appropriately controlled for: allergic disease[20], B-cell lymphoma[21] and rheumatoid arthritis[22]. Four SNPs (rs705705, rs186975: allergic disease, rs72993671: B-cell lymphoma, and rs181816009: rheumatoid arthritis) associated with these potential confounding factors were identified and eliminated from the analysis. Lastly, *F* statistic was used to evaluate the relationship between SNPs and exposure, if F<10, the correlation between SNPs and exposure is considered weak [23]. SNPs with a palindromic structure were automatically excluded during the analysis.
c)	Describe the MR estimator (e.g. two-stage least squares, Wald ratio) and related statistics. Detail the included covariates and, in case of two-sample MR, whether the same covariate set was used for adjustment in the two samples	Page 5-6, Lines 126–140	Inverse variance weighted (IVW) was used as the main analysis method to examine whether there was a causal association between inflammatory proteins and HT. weighted median, MR-Egger regression, weighted mode, simple mode and Mendelian randomization pleiotropy residual sum and outlier (MR-PRESSO) were used as supplementary methods. In the main analysis, we generated MR estimates for each individual SNP using the Wald ratio, and approximated the standard error using the delta method. Subsequently, we employed a random-effects inverse-variance weighting meta-analysis approach to combine individual MR estimates. Moreover, to mitigate the risk of Type I errors resulting from a relaxed threshold requirement and to assess the impact of multiple testing on research findings. The Benjamini-Hochberg method was used to control the false discovery rate (FDR), P < 0.05 but P_{FDR} > 0.05 was regarded as indicative of a possible association. In addition, our study also employed the weighted median, MR Egger, simple mode and weighted mode as supplementary approaches. Due to the limited statistical power of MR-Egger, our focus was on assessing the consistency of the estimate directions rather than their significance.

		d)	Explain how missing data were addressed	NA	This study used publicly available GWAS summary-level data, and no additional missing data processing was required in the MR analysis.
		e)	If applicable, indicate how multiple testing was addressed	Page 5, Lines 135–136	The Benjamini-Hochberg method was used to control the false discovery rate (FDR), P < 0.05 but $P_{FDR} > 0.05$ was regarded as indicative of a possible association.
7	**Assessment of assumptions**		Describe any methods or prior knowledge used to assess the assumptions or justify their validity	Page 5 Lines 108–125	First, for inflammatory proteins, since very few SNPs reached the standard genome-wide significance level ($P < 5×10^{-8}$), a more relaxed threshold of $P < 5×10^{-6}$ was applied to obtain a more comprehensive and reliable result. Second, the clustering program in PLINK (v1.90) was employed to prune these SNPs, using a linkage disequilibrium (LD) r^2 threshold of less than 0.001 and within 10000 kb distance. Third, SNPs were searched and obtained via PhenoScanner V2 (http://www.phenoscanner.medschl.cam.ac.uk/, accessed on 20 Dec 2023) to detect potential confounders[19]. For each SNP and its proxy ($r^2 > 0.80$), we systematically investigated any previously reported associations ($P < 5×10^{-6}$) based on relevant published studies. Three potential confounding factors were identified and appropriately controlled for: allergic disease[20], B-cell lymphoma[21] and rheumatoid arthritis[22]. Four SNPs (rs705705, rs186975: allergic disease, rs72993671: B-cell lymphoma, and rs181816009: rheumatoid arthritis) associated with these potential confounding factors were identified and eliminated from the analysis. Lastly, *F* statistic was used to evaluate the relationship between SNPs and exposure, if $F<10$, the correlation between SNPs and exposure is considered weak [23]. SNPs with a palindromic structure were automatically excluded during the analysis.
8	**Sensitivity analyses and additional analyses**		Describe any sensitivity analyses or additional analyses performed (e.g. comparison of effect estimates from different approaches, independent replication, bias analytic techniques, validation of instruments, simulations)	Page 6, Lines 141–150	To ensure the stability and reliability of the results, our study employed a series of comprehensive methods for sensitivity analysis. The MR Egger intercept method was used to assess potential directional pleiotropy [24]. Additionally, the MR-PRESSO was employed to further ensure that the study results were not influenced by

					confounding factors[25]. Furthermore, a leave-one-out sensitivity analysis was conducted to meticulously evaluate the impact of each IV[26]. In assessing the heterogeneity among SNPs, Cochran's Q statistic was chosen as the tool for heterogeneity evaluation[27]. If the data indicated no significant heterogeneity ($P > 0.05$), a fixed-effects model was applied for the MR analysis, conversely, a random-effects model was used to assess the potential causal relationship between proteins and HT.
9	**Software and pre-registration**				
		a)	Name statistical software and package(s), including version and settings used	Page 6, Lines 150–153	MR analyses were conducted in R (version 4.2.3) using the MendelianRandomization (version 0.9.0) and TwoSampleMR (version 0.5.7) packages.
		b)	State whether the study protocol and details were pre-registered (as well as when and where)	NA	This study used publicly available GWAS summary-level data and did not involve primary data collection or a prospective study protocol; therefore, pre-registration was not applicable.
	**RESULTS**				
10	**Descriptive data**				
		a)	Report the numbers of individuals at each stage of included studies and reasons for exclusion. Consider use of a flow diagram	NA	This study used publicly available GWAS summary-level data and did not involve individual-level participant selection or staged inclusion/exclusion processes
		b)	Report summary statistics for phenotypic exposure(s), outcome(s), and other relevant variables (e.g. means, SDs, proportions)	NA	Individual-level phenotypic data were not available in this study, as analyses were conducted using GWAS summary statistics; therefore, descriptive statistics such as means and standard deviations were not generated.
		c)	If the data sources include meta-analyses of previous studies, provide the assessments of heterogeneity across these studies	NA	Heterogeneity across studies included in the GWAS meta-analyses was assessed in the original publications, and details are available in the corresponding GWAS reports.

		d) For two-sample MR:<br>i. Provide justification of the similarity of the genetic variant-exposure associations between the exposure and outcome samples<br>ii. Provide information on the number of individuals who overlap between the exposure and outcome studies	Page 6, Lines 156–163	A slightly relaxed cut-off (P < 5×10-6) involved all 91 inflammatory proteins, with F-statistics ranging between 22.22 and 131.91 (Supplementary Table 1). To evaluate the influence of 91 inflammatory proteins on HT, IVW served as the primary method[28]. The MR analyses between the 91 inflammatory proteins and the HT can be found in Figure 3A and Supplementary Table 3. Both the exposure and outcome GWAS datasets were derived from individuals of European ancestry and originated from independent cohorts, which reduces the likelihood of population heterogeneity and potential sample overlap.
11	**Main results**			
		a) Report the associations between genetic variant and exposure, and between genetic variant and outcome, preferably on an interpretable scale	Page 6, Lines 156–157	A slightly relaxed cut-off (P < 5×10-6) involved all 91 inflammatory proteins, with F-statistics ranging between 22.22 and 131.91 (Supplementary Table 1).
		b) Report MR estimates of the relationship between exposure and outcome, and the measures of uncertainty from the MR analysis, on an interpretable scale, such as odds ratio or relative risk per SD difference	Page 6-7, Lines 164–202	Of the 91 inflammatory proteins, thirteen relevant inflammatory proteins that reached a nominal significance (*P* < 0.05) were screened from the primary analysis, including interleukin-12 subunit beta (IL-12p40) , FGF family members (FGF5, FGF19, FGF23), chemokine regulators (CCL20, CXCL10, MIP-1α), osteoprotegerin (OPG) , tumor necrosis factor (TNF), interferon gamma (IFN-γ), fms-related tyrosine kinase 3 ligand (FLT3L), CUB domain-containing protein 1 (CDCP1), T-cell surface glycoprotein CD5 (CD5), Figure 2 shows IVW analytical findings for these thirteen inflammatory proteins.<br>After FDR correction, only genetically predicted IL-12p40 was significantly associated with increased HT risk (IVW: OR [95% CI] = 1.295 [1.172, 1.431] , $P = 3.66\times10^{-7}$, $P_{FDR} = 3.33\times10^{-5}$) .Although the results of other MR analysis methods did not reach statistical significance , they showed consistent trends or directions with the IVW analysis results(MR-Egger: OR [95% CI] = 1.012 [0.758-1.351] , *P* = 0.935; Weighted median: OR [95% CI] = 1.242 [1.059-1.457], *P* = 0.008; Weighted mode: OR

			[95% CI] = 1.084 [0.819-1.433], *P* = 0.579; Simple mode: OR [95% CI] = 1.097 [0.794-1.517], *P* = 0.581).<br>Using 91 inflammatory proteins as the outcome to investigate the possibility of reverse causation, we thoroughly examined 46 SNPs that showed strong associations with HT, using a threshold of $P < 5\times10^{-6}$. Genetic instrument strength was confirmed by F-statistics exceeding the empirical threshold of 10 (range:32.82-38.58), demonstrating methodological robustness against weak instrument bias. Comprehensive SNP characteristics are presented in Supplementary Table 2, while the effect of HT on 91 inflammatory proteins is shown in Figure 3B and Supplementary Table 4.<br>Subsequently, the IVW analysis identified four inflammatory casually linked to HT ($P < 0.05$; Figure 4), including CCL19, CCL25, CCL4 and TNFRSF9. For the analyses involving CCL19 and TNFRSF9, 3 and 1 SNPs were not present in the outcome GWAS, respectively, and no proxy SNPs were available, leaving 43 and 45 SNPs for analysis. After FDR correction, no inflammatory proteins were observed as a downstream consequence of HT.
	c) If relevant, consider translating estimates of relative risk into absolute risk for a meaningful time period	NA	Mendelian randomization estimates represent genetically predicted causal effects rather than time-specific relative risks; therefore, conversion to absolute risk over a defined time period was not applicable.
	d) Consider plots to visualize results (e.g. forest plot, scatterplot of associations between genetic variants and outcome versus between genetic variants and exposure)	Page 7, Lines 170–171;<br>Page 8, Lines 197–198	Figure 2 shows IVW analytical findings for these thirteen inflammatory proteins.<br>Subsequently, the IVW analysis identified four inflammatory casually linked to HT (P < 0.05; Figure 4)
12	**Assessment of assumptions**		

		a)	Report the assessment of the validity of the assumptions	Page 7, Lines 181–186; Page 8, Lines 202–206	The graphical representation of sensitivity analysis can be found in Graphic Abstract. After applying the MR-PRESSO outlier test to remove outlier SNPs (Outliers: rs10043720), Both MR-PRESSO global test (P = 0.082) and the MR-Egger intercept analysis (intercept = 0.026, P = 0.082) revealed no significant horizontal pleiotropy. for horizontal pleiotropy. The "leave one out" analysis demonstrated the robustness of our MR analysis, as it was not affected by any individual SNP. No invalid SNPs were detected using the MR-Steiger filtering method.<br>The MR- Egger intercept test provided no evidence of potential pleiotropy (all P > 0.05; Supplementary Table 4), while MR-PRESSO showed the presence of pleiotropic SNPs in the GWAS dataset for TNFRSF9 (P = 0.043, Supplementary Table 4). The results of the sensitivity analysis can be found in the supplementary Table 4.
		b)	Report any additional statistics (e.g., assessments of heterogeneity across genetic variants, such as I^2, Q statistic or E-value)	Page 7, Lines 179–180	Q statistics (P = 0.141) showed no statistically significant differences, suggesting no heterogeneity (Supplementary Table 3).
13	**Sensitivity analyses and additional analyses**				
		a)	Report any sensitivity analyses to assess the robustness of the main results to violations of the assumptions	Page 7, Lines 179–187	Q statistics (P = 0.141) showed no statistically significant differences, suggesting no heterogeneity (Supplementary Table 3). The graphical representation of sensitivity analysis can be found in Graphic Abstract. After applying the MR-PRESSO outlier test to remove outlier SNPs (Outliers: rs10043720), Both MR-PRESSO global test (P = 0.082) and the MR-Egger intercept analysis (intercept = 0.026, P = 0.082) revealed no significant horizontal pleiotropy. for horizontal pleiotropy. The "leave one out" analysis demonstrated the robustness of our MR analysis, as it was not affected by any individual SNP. No invalid SNPs were detected using the MR-Steiger filtering method.

			The MR- Egger intercept test provided no evidence of potential pleiotropy (all P > 0.05; Supplementary Table 4), while MR-PRESSO showed the presence of pleiotropic SNPs in the GWAS dataset for TNFRSF9 (P = 0.043, Supplementary Table 4). The results of the sensitivity analysis can be found in the supplementary Table 4.
b)	Report results from other sensitivity analyses or additional analyses	Page 7, Lines 174–179	Although the results of other MR analysis methods did not reach statistical significance , they showed consistent trends or directions with the IVW analysis results(MR-Egger: OR [95% CI] = 1.012 [0.758-1.351] , P = 0.935; Weighted median: OR [95% CI] = 1.242 [1.059-1.457], P = 0.008; Weighted mode: OR [95% CI] = 1.084 [0.819-1.433], P = 0.579; Simple mode: OR [95% CI] = 1.097 [0.794-1.517], P = 0.581).
c)	Report any assessment of direction of causal relationship (e.g., bidirectional MR)	Page 6-8, Lines 164–206	Of the 91 inflammatory proteins, thirteen relevant inflammatory proteins that reached a nominal significance (P < 0.05) were screened from the primary analysis, including interleukin-12 subunit beta (IL-12p40) , FGF family members (FGF5, FGF19, FGF23), chemokine regulators (CCL20, CXCL10, MIP-1α), osteoprotegerin (OPG) , tumor necrosis factor (TNF), interferon gamma (IFN-γ), fms-related tyrosine kinase 3 ligand (FLT3L), CUB domain-containing protein 1 (CDCP1), T-cell surface glycoprotein CD5 (CD5), Figure 2 shows IVW analytical findings for these thirteen inflammatory proteins.<br><br>After FDR correction, only genetically predicted IL-12p40 was significantly associated with increased HT risk (IVW: OR [95% CI] = 1.295 [1.172, 1.431] , P = 3.66×10-7 , PFDR = 3.33×10-5) .Although the results of other MR analysis methods did not reach statistical significance , they showed consistent trends or directions with the IVW analysis results(MR-Egger: OR [95% CI] = 1.012 [0.758-1.351] , P = 0.935; Weighted median: OR [95% CI] = 1.242 [1.059-1.457], P = 0.008; Weighted mode: OR [95% CI] = 1.084 [0.819-1.433], P = 0.579; Simple mode: OR [95% CI] = 1.097 [0.794-1.517], P = 0.581). Q statistics (P = 0.141) showed no statistically significant

			differences, suggesting no heterogeneity (Supplementary Table 3). The graphical representation of sensitivity analysis can be found in Graphic Abstract. After applying the MR-PRESSO outlier test to remove outlier SNPs (Outliers: rs10043720), both MR-PRESSO global test ($P = 0.082$) and the MR-Egger intercept analysis (intercept = 0.026, $P = 0.082$) revealed no significant horizontal pleiotropy. for horizontal pleiotropy. The "leave one out" analysis demonstrated the robustness of our MR analysis, as it was not affected by any individual SNP. No invalid SNPs were detected using the MR-Steiger filtering method.<br><br>3.2. Secondary Analyses<br><br>Using 91 inflammatory proteins as the outcome to investigate the possibility of reverse causation, we thoroughly examined 46 SNPs that showed strong associations with HT, using a threshold of $P < 5\times10^{-6}$. Genetic instrument strength was confirmed by F-statistics exceeding the empirical threshold of 10 (range:32.82-38.58), demonstrating methodological robustness against weak instrument bias. Comprehensive SNP characteristics are presented in Supplementary Table 2, while the effect of HT on 91 inflammatory proteins is shown in Figure 3B and Supplementary Table 4.<br><br>Subsequently, the IVW analysis identified four inflammatory casually linked to HT ($P < 0.05$; Figure 4), including CCL19, CCL25, CCL4 and TNFRSF9. For the analyses involving CCL19 and TNFRSF9, 3 and 1 SNPs were not present in the outcome GWAS, respectively, and no proxy SNPs were available, leaving 43 and 45 SNPs for analysis. After FDR correction, no inflammatory proteins were observed as a downstream consequence of HT.
d)	When relevant, report and compare with estimates from non-MR analyses	NA	No non-MR observational analyses were conducted for comparison in this study.

		e) Consider additional plots to visualize results (e.g., leave-one-out analyses)	Page 7, Lines 184–187	The "leave one out" analysis demonstrated the robustness of our MR analysis, as it was not affected by any individual SNP. No invalid SNPs were detected using the MR-Steiger filtering method. The graphical representation of sensitivity analysis can be found in Graphic Abstract.
	**DISCUSSION**			
14	**Key results**	Summarize key results with reference to study objectives	Page 8, Lines 208–216	In this study, we performed MR analyses to explore the association between 91 inflammatory proteins and the risk of HT. Our findings indicate a genetically predicted association between IL-12p40 and HT, providing evidence for a potential causal role of IL-12p40 in HT etiology. Before FDR correction, MR analyses identified 12 inflammatory proteins associated with HT risk. In the reverse-direction analysis, HT was associated with four inflammatory proteins. However, after FDR correction, no causal relationship was identified between these 16 inflammatory proteins and HT. Notably, MR primarily informs etiological relevance, whereas the therapeutic implications of targeting this pathway warrant further investigation.
15	**Limitations**	Discuss limitations of the study, taking into account the validity of the IV assumptions, other sources of potential bias, and imprecision. Discuss both direction and magnitude of any potential bias and any efforts to address them	Page 11, Lines 316–333	However, this study has several limitations. First, due to the limitations of MR analysis, it may not fully address the second and third hypotheses, potentially introducing bias. Second, further experimental studies and well-designed clinical trials in patients with HT are required to validate these findings and assess their potential clinical relevance. Third, this study was limited to participants of European ancestry, which may restrict the generalizability of our findings to populations of other ethnic backgrounds. Fourth, a relaxed SNP selection threshold ($P < 5 \times 10^{-6}$) was used to identify instrumental variables, which may increase the risk of false-positive findings. Therefore, the results should be interpreted with caution. Fifth, the limited number of SNPs and the fact that some confounders are unknown make it possible for unmeasured confounders to bias the results, potentially affecting both HT and inflammatory proteins levels. Finally, the HT phenotype may be clinically

				heterogeneous, encompassing euthyroid HT, overt hypothyroidism, and antibody-positive individuals. Such phenotype misclassification could attenuate the associations between genetic instruments and the true disease status, potentially biasing MR estimates toward the null. Future studies incorporating more precise phenotyping, including antibody titers, thyroid function assessments, or ultrasound features, are warranted to enable stratified analyses and reveal more specific causal relationships.
16	**Interpretation**			
		a) Meaning: Give a cautious overall interpretation of results in the context of their limitations and in comparison with other studies	Page 11, Lines 303–315	This study systematically investigated the associations between Hashimoto's thyroiditis and 91 circulating inflammatory proteins using MR. Our findings are supported by prior literature that has examined both upstream and downstream inflammatory proteins in HT. Clinical trials of treating immune disorders by targeting IL-12p40 have provided satisfactory therapeutic results53. The treatment of HT has remained largely unchanged over the past few decades. Patients may need the option of long-term treatment with lifelong thyroid hormone replacement therapy if they have hypothyroidism. Future studies should aim to better understand the pathogenesis of HT in order to obtain safe, conservative, and definitive treatments that target the cause. Our findings offer further insights into the inflammatory mechanisms underlying Hashimoto's thyroiditis and may inform future studies on therapeutic target development.
		b) Mechanism: Discuss underlying biological mechanisms that could drive a potential causal relationship between the investigated exposure and the outcome, and whether the gene-environment equivalence assumption is reasonable. Use causal language carefully, clarifying that IV estimates may provide causal effects only under certain assumptions	Page 8-11, Lines 217–315	Numerous inflammatory proteins originating from infiltrating lymphocytes have been implicated in the pathogenesis of HT[29]. One study identified significant causal associations between proteins associated with lymphocyte function and the risk of hypothyroidism[12]. Moreover, two other studies found the causal associations between several inflammatory proteins, including IL-12p70, IL-7, and MIP-1β, and both autoimmune thyroid disease and hypothyroidism[13, 14]. Additionally, HT may manifest Th1 and Th17 immune

responses, promoting cell-mediated immunity and triggering apoptosis-driven destruction of thyroid follicular cells. The inflammatory proteins produced in these responses play a non-negligible role [5, 6]. These findings indicate that proteins associated with immune and inflammatory responses may serve as essential contributors to the onset and progression of HT.

IL-12p40 (also referred to as IL-12β) is a shared subunit of IL-12 and IL-23 but is believed to exert distinct biological functions[30]. Activated inflammatory immune cells produce IL-12p40, which performs intricate and diverse functions in various inflammatory microenvironments[31]. Several research findings propose a potential correlation between IL-12p40 and HT. Previous experimental research has demonstrated a notable elevation in plasma levels of IL-12p40 and IL-12p70 in the study group relative to the control group[32]. Patients with IL-12p40 deficiency exhibit undetectable serum concentrations of both IL-12p40 and IL-12p70. Furthermore, animal studies indicate that mice lacking IL-12p40 or IL-18 experience a transition from a Th1 to a Th2 cytokine response, along with a decline in cytotoxic natural killer (NK) cells and cytotoxic T lymphocytes (CTLs) [33]. Mice deficient in IL-12p40 exhibit resistance to disease in various preclinical models of chronic inflammation and autoimmunity[34, 35]. A recent study indicated that IL-12p40 potentially exerts an enhancing effect on Th17 cell differentiation while promoting the polarization of pro-inflammatory macrophages in murine models[36]. Th17 cells, a subpopulation of pro-inflammatory T-lymphocytes identified subsequent to TH1 and TH2 cells, are known to play a significant role in immuno-inflammatory processes[37]. Usually, patients with HT have higher levels of TgAb[38]. Studies have shown that IL-12p40 concentration in unstimulated whole saliva is positively correlated with plasma TgAb concentration[32]. These findings support our study. However, it is important to note that there is heterogeneity in the findings across various studies. Ikeda *et al.* reported no statistically significant correlation between IL-12B gene polymorphisms and thyroiditis[39]. These differences might

stem from study design variations, sample size constraints, and population heterogeneity. Moreover, HT is a multifactorial disease shaped by various genetic and environmental influences. The collective impact of polymorphisms in inflammatory proteins may have a greater influence on the susceptibility and severity of HT, while IL-12B gene polymorphisms may constitute only a fraction of the overall mechanism[40]. Hence, additional research is necessary to further explore the contentious nature of the relationship between IL-12B gene polymorphisms and thyroiditis.

IL-12 is a inflammatory protein composed of two subunits, p40 subunit and p35 subunit[41]. Notably, the absence of the p40 subunit leads to the complete lack of both IL-12 and IL-23. Studies have shown that serum IL-12 levels are elevated in patients with HT when compared to individuals with toxic nodular goiter, Graves' disease (GD), or healthy controls[6]. A separate study utilized IL-12 transgenic mouse models investigated the role of IL-12 in the pathogenesis of HT and its associated immune mechanisms, revealing that the presence of IL-12 in the thyroid leads to thyroiditis[42]. IL-12p40 combines with IL-12p35 to form bioactive IL-12, which can induce CD4+ T lymphocytes to differentiate from the Th0 to the Th1 phenotype [43]. The presence of IL-12 can also activate immune cells, including NKT cells, NK cells, and T cells. The activation of these immune cells may lead to enhanced inflammation and cell-mediated immune responses, further damaging thyroid follicular cells. While we have understood that IL-12 may play a detrimental role in HT, the specific mechanisms and their sequential order still require further in-depth research to ascertain.

IL-23 consists of a distinct p19 subunit combined with the p40 subunit of IL-12[44]. Although the measurement of p40 chain is often interpreted as a measurement of the complete p75 heterodimer, detecting p40 is only a measurement of one subunit of IL-12. Therefore, if p40 is detected, it implies the detection of both IL-12 and IL-23 simultaneously. Antigen producing cells, including macrophages and dendritic cells, produce IL-23, which plays a pivotal role in the differentiation of pathogenic

Th17 lymphocytes[44]. Previous research suggests that a higher Th17 cell count in the blood of HT patients is positively associated with structural damage to the thyroid. IL-23 drives HT pathogenesis by enhancing Th17 cell formation and inducing the release of IL-17[45, 46].

In addition, the accumulation of abnormal reactive oxygen species (ROS) is recognized as a detrimental factor contributing to the development of HT, and autophagy plays a crucial role in mitigating cellular damage and preventing cell death by serving as a significant mechanism for the removal of excessive ROS[47, 48]. Research by Zheng *et al.* demonstrated heightened IL-23 production within thyroid follicular cell (TFC) obtained from HT patients and confirmed that IL-23 can induce ROS accumulation and autophagy inhibition in TFCs[49]. A recent study revealed a noteworthy elevation in serum IL-23 levels in patients diagnosed with HT when compared to both GD patients and healthy individuals[50]. The pathogenesis and pathophysiology of HT involve multiple complex processes, of which immune-mediated inflammation represents only one component. It is worth noting that whether the potential role of IL-23 in HT is associated with IL-12p40 remains to be further explored.

Interestingly, our study suggests a possible link between IFN-γ and a decreased risk of HT. TH1 and TH17-mediated cell immune responses have been implicated in HT development. The IFN-γ, produced by TH1 cells, stimulates antigen-presenting cells and enhances TH1 cell differentiation by upregulating the transcription factor T-bet[51]. However, it impedes the initial differentiation of T cells into TH17 subsets[52]. Notably, studies have demonstrated that a deficiency in IFN-γ can worsen autoimmune inflammation, as observed in experimental autoimmune encephalomyelitis. Hence, further investigation is required to uncover the mechanisms involved and investigate the therapeutic potential of IFN-γ in HT and related autoimmune inflammatory disorders.

		c) Clinical relevance: Discuss whether the results have clinical or public policy relevance, and to what extent they inform effect sizes of possible interventions	Page 11, Lines 304–315	Our findings are supported by prior literature that has examined both upstream and downstream inflammatory proteins in HT. Clinical trials of treating immune disorders by targeting IL-12p40 have provided satisfactory therapeutic results[53]. The treatment of HT has remained largely unchanged over the past few decades. Patients may need the option of long-term treatment with lifelong thyroid hormone replacement therapy if they have hypothyroidism. Future studies should aim to better understand the pathogenesis of HT in order to obtain safe, conservative, and definitive treatments that target the cause. Our findings offer further insights into the inflammatory mechanisms underlying Hashimoto's thyroiditis and may inform future studies on therapeutic target development.
17	**Generalizability**	Discuss the generalizability of the study results (a) to other populations, (b) across other exposure periods/timings, and (c) across other levels of exposure	Page 12, Lines 320–322	This study was limited to participants of European ancestry, which may restrict the generalizability of our findings to populations of other ethnic backgrounds.
	**OTHER INFORMATION**			
18	**Funding**	Describe sources of funding and the role of funders in the present study and, if applicable, sources of funding for the databases and original study or studies on which the present study is based	Page 13, Lines 366–369	This work was supported by the National Natural Science Foundation of China (Grant No. 82003560 by YK； 82170865 by XS), the National Natural Science Foundation of Shandong (Grant No. ZR2020MH340 by YK) and Science and Technology Program of Shaanxi Province (Grant No. 2024SF-YBXM-256 by BY).
19	**Data and data sharing**	Provide the data used to perform all analyses or report where and how the data can be accessed, and reference these sources in the article. Provide the statistical code needed to reproduce the results in the article, or report whether the code is publicly accessible and if so, where	Page 12, Lines 347–349	The data for 91 inflammatory proteins were derived from GWAS Catalog (https://www.ebi.ac.uk/gwas/). The data for HT were derived from IEU Open GWAS (https://gwas.mrcieu.ac.uk/).
20	**Conflicts of Interest**	All authors should declare all potential conflicts of interest	Page 12, Lines 345	The authors declare no conflict of interest.

This checklist is copyrighted by the Equator Network under the Creative Commons Attribution 3.0 Unported (CC BY 3.0) license.

1. Skrivankova VW, Richmond RC, Woolf BAR, Yarmolinsky J, Davies NM, Swanson SA, et al. Strengthening the Reporting of Observational Studies in Epidemiology using Mendelian Randomization (STROBE-MR) Statement. JAMA. 2021;under review.

2. Skrivankova VW, Richmond RC, Woolf BAR, Davies NM, Swanson SA, VanderWeele TJ, et al. Strengthening the Reporting of Observational Studies in Epidemiology using Mendelian Randomisation (STROBE-MR): Explanation and Elaboration. BMJ. 2021;375:n2233.
