# Supplementary material for: Exploring Causal Links Between 91 Circulating Inflammatory Proteins and Hashimoto’s Thyroiditis: A Bidirectional Mendelian Randomization Study
Source: Mediators Inflamm. 2026 Sep 7;2026:6651720. doi: 10.1155/mi/6651720 (PMC13550872; doi:10.1155/mi/6651720)
Supplement: Supplementary file 2 [file MI-2026-6651720-s002.pdf]

Supplementary Table 1. Details of systemic inflammatory proteins predicting SNPs with Hashimoto's Thyroiditis from GWAS (with SNPs reaching *P*< 5×10^{-6})

Inflammatory proteins	Hashimoto's Thyroiditis						
SNP	Effect allele	Other allele	P	Beta	Standard Error	log(OR)	Standard Error
**ADA (F-statistic = 131.91, R2=13.21%)**							
rs10024178	T	C	3.35E-06	-0.093	0.020	-0.001	0.025
rs111478357	T	C	3.39E-06	0.204	0.044	-0.044	0.071
rs112665079	T	C	1.00E-200	0.893	0.026	0.007	0.041
rs115657851	A	G	2.10E-06	0.139	0.029	0.021	0.035
rs117645472	A	T	3.59E-06	0.349	0.075	-0.313	0.209
rs12479560	A	G	3.26E-16	0.120	0.015	-0.031	0.017
rs1608554	A	G	9.50E-07	0.060	0.012	0.021	0.018
rs189104742	A	T	2.41E-06	0.275	0.058	0.026	0.052
rs2527856	T	C	4.09E-06	0.056	0.012	0.026	0.017
rs4749761	A	T	4.07E-06	0.555	0.120	-0.098	0.620
rs541944924	T	C	2.97E-06	0.167	0.036	-0.006	0.037
rs62319741	T	C	6.87E-07	0.128	0.026	0.051	0.033
rs7292320	A	G	4.82E-06	-0.060	0.013	-0.007	0.020
rs75492374	A	G	3.97E-06	0.355	0.077	0.046	0.112
rs80212763	T	C	1.10E-103	0.969	0.045	-0.002	0.077
**ARTN (F-statistic = 22.59, R2=4.14%)**							
rs118115625	T	C	2.55E-06	0.170	0.036	-0.056	0.069
rs1329733	A	G	7.20E-07	-0.067	0.014	-0.005	0.016
rs13303278	A	C	4.01E-06	-0.066	0.014	-0.029	0.017
rs1370215	T	C	4.76E-06	-0.070	0.015	0.003	0.018
rs138311590	A	G	4.07E-06	-0.081	0.018	-0.005	0.023
rs142585779	T	C	1.11E-06	0.212	0.044	0.018	0.045
rs142970662	A	G	1.13E-06	0.311	0.064	0.028	0.087
rs148226005	A	G	2.83E-06	0.211	0.045	0.036	0.340
rs17340161	A	G	3.86E-06	-0.087	0.019	0.031	0.025
rs3003450	T	G	3.97E-06	0.063	0.014	-0.041	0.018
rs4993886	A	T	1.08E-06	-0.079	0.016	0.008	0.054
rs55885546	A	G	3.28E-06	-0.205	0.044	-0.003	0.043
rs569463048	T	G	4.88E-06	-0.269	0.059	0.494	0.330

Supplementary Table 1. Details of systemic inflammatory proteins predicting SNPs with Hashimoto's Thyroiditis from GWAS (with SNPs reaching $P < 5\times10^{-6}$)

Inflammatory proteins	Hashimoto's Thyroiditis						
SNP	Effect allele	Other allele	P	Beta	Standard Error	log(OR)	Standard Error
rs62277657	A	G	5.22E-07	0.083	0.017	0.001	0.022
rs6509909	C	G	5.57E-07	-0.088	0.018	0.028	0.020
rs76672219	A	C	2.24E-06	-0.323	0.068	0.061	0.080
rs77502336	C	G	1.47E-06	0.068	0.014	0.031	0.018
rs9496445	T	C	2.44E-06	0.085	0.018	0.023	0.024
rs9684768	C	G	7.70E-07	0.069	0.014	0.047	0.017
rs9941952	T	C	4.63E-06	0.077	0.017	-0.008	0.020
**AXIN1 (F-statistic = 23.38, R2=1.66%)**							
rs115455827	A	G	2.09E-06	0.145	0.031	-0.001	0.032
rs117097664	A	C	2.84E-06	0.244	0.052	0.040	0.061
rs148317708	A	G	4.47E-07	-0.212	0.042	-0.017	0.082
rs2470622	A	G	3.80E-07	-0.123	0.024	-0.013	0.028
rs2691530	T	C	4.71E-07	-0.067	0.013	0.007	0.017
rs2712427	A	G	2.99E-06	0.063	0.013	-0.006	0.016
rs428324	T	C	2.93E-06	0.069	0.015	0.018	0.017
rs7171268	T	C	2.32E-06	0.155	0.033	0.018	0.044
**Capase 8 (F-statistic = 30.92, R2=3.21%)**							
rs1032417	C	G	1.99E-07	-0.086	0.017	0.063	0.024
rs1034059	A	G	2.65E-06	0.068	0.015	0.038	0.019
rs111764025	C	G	4.21E-06	0.110	0.024	-0.036	0.038
rs11695193	A	T	6.51E-07	-0.081	0.016	0.004	0.027
rs11709039	T	C	3.34E-06	0.078	0.017	-0.023	0.025
rs12073499	A	C	1.16E-06	-0.081	0.017	0.021	0.021
rs137887001	C	G	1.85E-06	0.232	0.049	0.092	0.070
rs140844269	C	G	3.19E-06	-0.362	0.078	0.176	0.086
rs148608719	A	G	3.65E-07	0.347	0.068	-0.006	0.051
rs187405204	T	C	4.23E-06	0.420	0.091	0.075	0.073
rs34154371	T	C	1.78E-06	-0.202	0.042	0.040	0.072
rs56328050	C	G	1.56E-30	-0.199	0.017	-0.014	0.027
rs73306603	T	C	7.01E-07	0.204	0.041	0.120	0.099
rs78449258	A	G	2.94E-06	-0.200	0.043	-0.025	0.090

Supplementary Table 1. Details of systemic inflammatory proteins predicting SNPs with Hashimoto's Thyroiditis from GWAS (with SNPs reaching *P*< 5×10^{-6})

Inflammatory proteins	Hashimoto's Thyroiditis						
SNP	Effect allele	Other allele	P	Beta	Standard Error	log(OR)	Standard Error
**CCL19 (F-statistic = 35.92, R2=5.03%)**							
rs10242459	A	G	2.20E-06	0.073	0.015	-0.005	0.023
rs10496135	C	G	1.34E-06	0.186	0.038	0.075	0.049
rs113539352	C	G	1.15E-06	0.449	0.092	-0.352	0.313
rs11574915	A	C	7.19E-18	-0.146	0.017	0.008	0.024
rs138108512	A	G	1.83E-06	-0.294	0.062	-0.195	0.120
rs1415763	C	G	1.42E-06	0.076	0.016	-0.031	0.020
rs147708384	T	C	2.40E-06	-0.271	0.057	-0.106	0.109
rs28635588	A	G	6.90E-18	-0.178	0.021	0.154	0.081
rs34791102	A	G	2.99E-06	0.124	0.027	0.041	0.050
rs3792790	A	C	4.40E-06	0.055	0.012	-0.022	0.017
rs4554017	T	C	1.03E-06	0.060	0.012	-0.018	0.017
rs4907572	A	G	9.69E-07	-0.067	0.014	-0.004	0.018
rs62292952	T	G	7.53E-37	0.235	0.019	0.007	0.024
rs6486995	A	T	2.43E-07	-0.070	0.014	0.001	0.019
rs6870560	T	C	3.28E-07	0.086	0.017	-0.018	0.023
rs73257451	T	C	1.43E-06	-0.365	0.076	0.073	0.059
rs74764632	A	T	4.47E-06	0.135	0.029	0.034	0.036
rs7595241	C	G	2.24E-06	0.062	0.013	0.045	0.018
rs79086127	T	C	3.20E-06	-0.210	0.045	0.128	0.109
**CCL20 (F-statistic = 26.74, R2=3.83%)**							
rs10207134	T	C	7.18E-15	-0.100	0.013	0.022	0.020
rs10837780	T	C	4.63E-06	0.119	0.026	-0.002	0.035
rs111674022	T	C	4.61E-06	-0.095	0.021	0.039	0.028
rs11242693	T	C	2.38E-06	0.064	0.014	0.006	0.017
rs1129474	T	C	1.80E-06	0.057	0.012	-0.019	0.017
rs11582772	T	C	2.39E-06	0.259	0.055	0.111	0.122
rs117753190	C	G	2.56E-07	-0.185	0.036	0.120	0.080
rs117900576	C	G	1.45E-07	-0.226	0.043	-0.032	0.082
rs13049624	A	G	1.89E-06	-0.132	0.028	0.041	0.033
rs151092565	T	C	4.03E-06	-0.151	0.033	-0.017	0.055

Supplementary Table 1. Details of systemic inflammatory proteins predicting SNPs with Hashimoto's Thyroiditis from GWAS (with SNPs reaching $P < 5\times10^{-6}$)

Inflammatory proteins	Hashimoto's Thyroiditis						
SNP	Effect allele	Other allele	P	Beta	Standard Error	log(OR)	Standard Error
rs2003431	A	T	3.21E-06	-0.160	0.034	0.031	0.043
rs2796032	T	C	5.50E-07	0.064	0.013	0.003	0.018
rs35833676	T	C	1.28E-06	0.080	0.017	-0.037	0.026
rs41302923	A	G	2.82E-06	0.194	0.041	0.053	0.053
rs4981022	A	G	7.02E-07	0.064	0.013	-0.003	0.018
rs5769672	T	G	4.09E-06	-0.118	0.026	0.024	0.050
rs6420556	T	C	3.19E-06	-0.074	0.016	0.025	0.022
rs6498926	A	T	4.29E-06	0.062	0.013	0.007	0.017
rs73072893	A	G	1.09E-06	0.206	0.042	-0.032	0.047
rs742493	T	C	3.11E-15	0.146	0.019	-0.065	0.028
**CCL23 (F-statistic = 366.27, R2=11.44%)**							
rs1001495	T	C	4.83E-06	0.098	0.022	-0.025	0.027
rs11017721	A	G	1.79E-07	0.097	0.019	-0.006	0.029
rs112492	A	G	7.70E-07	-0.069	0.014	-0.038	0.061
rs113959305	T	C	9.50E-07	-0.140	0.029	-0.105	0.050
rs116683909	A	G	8.24E-07	-0.210	0.043	0.050	0.054
rs117639761	A	C	3.85E-15	-0.325	0.041	-0.060	0.060
rs12204524	C	G	9.04E-07	0.116	0.024	-0.016	0.039
rs12949647	A	G	2.69E-06	-0.064	0.014	0.024	0.018
rs143910182	T	C	1.56E-06	0.216	0.045	-0.534	0.426
rs146074782	A	G	1.01E-06	-0.306	0.063	-0.004	0.078
rs16971993	T	C	2.75E-07	-0.129	0.025	0.077	0.041
rs17735284	A	T	4.45E-06	0.093	0.020	-0.020	0.036
rs1926742	A	G	4.39E-06	0.057	0.013	-0.003	0.018
rs2870238	T	C	4.78E-07	0.057	0.011	-0.028	0.017
rs4317138	T	C	4.10E-06	0.059	0.013	0.034	0.018
rs438811	T	C	1.25E-06	0.067	0.014	-0.002	0.019
rs4719850	T	C	3.42E-06	-0.055	0.012	0.012	0.018
rs712048	A	C	1.00E-200	-0.515	0.016	0.026	0.024
rs7225405	T	G	9.00E-15	-0.226	0.029	-0.305	0.244
rs73071153	A	G	2.70E-06	-0.150	0.032	-0.076	0.049

Supplementary Table 1. Details of systemic inflammatory proteins predicting SNPs with Hashimoto's Thyroiditis from GWAS (with SNPs reaching *P*< 5×10⁻⁶)

Inflammatory proteins	Hashimoto's Thyroiditis						
SNP	Effect allele	Other allele	P	Beta	Standard Error	log(OR)	Standard Error
rs73114426	A	G	4.66E-06	0.095	0.021	-0.012	0.027
rs74127049	C	G	4.92E-06	0.060	0.013	0.003	0.018
rs74491711	A	G	4.67E-06	0.127	0.028	0.042	0.034
rs77083410	C	G	7.26E-08	0.224	0.042	-0.048	0.043
rs77354000	A	G	2.32E-06	-0.206	0.044	0.018	0.059
rs77527696	T	C	4.81E-08	-0.254	0.047	-0.053	0.048
**CCL25 (F-statistic = 89.38, R2=15.33%)**							
rs10792444	T	C	1.76E-06	-0.087	0.018	0.025	0.025
rs11028036	A	G	3.06E-06	0.055	0.012	0.021	0.017
rs11140450	A	T	3.65E-06	-0.268	0.058	0.088	0.044
rs111437511	T	C	1.31E-06	0.144	0.030	0.023	0.053
rs116534774	T	C	1.72E-06	0.372	0.078	0.161	0.166
rs12114935	A	G	6.11E-08	0.077	0.014	-0.015	0.019
rs12606983	T	C	5.10E-07	-0.067	0.013	-0.041	0.018
rs143132666	A	C	1.90E-06	-0.193	0.041	-0.093	0.054
rs147851492	A	G	2.17E-06	0.213	0.045	-0.031	0.054
rs149395110	T	C	4.92E-06	-0.278	0.061	-0.029	0.120
rs1705699	T	C	2.52E-06	-0.055	0.012	-0.037	0.017
rs521795	T	C	3.14E-07	-0.062	0.012	0.004	0.017
rs549735081	T	C	1.67E-62	-0.467	0.028	0.030	0.196
rs635634	T	C	1.52E-50	-0.248	0.017	0.003	0.020
rs636479	A	G	4.12E-06	0.055	0.012	-0.011	0.017
rs7137942	A	G	1.67E-06	-0.064	0.013	-0.018	0.017
rs7252764	T	C	3.61E-15	0.232	0.030	-0.034	0.029
rs7296588	A	G	1.66E-23	-0.116	0.012	-0.007	0.017
rs74256540	A	G	3.58E-13	0.124	0.017	-0.005	0.023
rs759817	T	C	1.00E-200	0.392	0.012	0.015	0.017
rs7611785	A	G	2.01E-06	0.094	0.020	-0.014	0.023
rs7806274	T	C	2.74E-06	-0.076	0.016	-0.027	0.021
rs79990760	A	G	4.52E-06	0.219	0.048	-0.056	0.056
rs851850	A	G	1.87E-06	-0.078	0.016	-0.012	0.023

Supplementary Table 1. Details of systemic inflammatory proteins predicting SNPs with Hashimoto's Thyroiditis from GWAS (with SNPs reaching $P < 5\times10^{-6}$)

Inflammatory proteins	Hashimoto's Thyroiditis						
SNP	Effect allele	Other allele	P	Beta	Standard Error	log(OR)	Standard Error
**CCL28 (F-statistic = 25.24, R2=4.12%)**							
rs11078696	T	G	4.92E-07	-0.086	0.017	0.124	0.126
rs112641086	T	C	9.64E-08	-0.106	0.020	-0.001	0.027
rs116782490	A	C	2.49E-06	0.230	0.049	0.044	0.138
rs139902	A	C	1.58E-08	0.068	0.012	0.006	0.017
rs143400203	T	C	3.79E-06	0.181	0.039	0.038	0.048
rs1514964	T	C	1.72E-06	-0.126	0.026	-0.028	0.040
rs2477954	T	C	1.34E-06	-0.058	0.012	-0.008	0.017
rs2631360	A	G	3.10E-06	0.060	0.013	0.014	0.018
rs2866626	C	G	8.06E-07	-0.060	0.012	-0.034	0.018
rs371663005	A	G	4.02E-06	-0.096	0.021	0.065	0.147
rs4866742	T	C	8.71E-09	0.070	0.012	-0.002	0.017
rs546951873	A	G	3.40E-07	0.343	0.067	0.005	0.086
rs6580980	A	G	5.53E-09	-0.069	0.012	0.009	0.018
rs6671688	T	C	4.16E-06	0.053	0.012	0.008	0.017
rs71363004	C	G	2.87E-06	0.218	0.047	0.027	0.103
rs73000929	A	G	5.02E-08	-0.194	0.036	-0.040	0.050
rs76291845	A	G	6.55E-07	0.270	0.054	-0.036	0.072
rs7661151	T	C	4.70E-06	0.053	0.012	0.005	0.017
rs76669782	A	C	2.68E-08	-0.285	0.051	-0.011	0.101
rs77569365	T	G	4.77E-06	0.129	0.028	0.021	0.048
rs78552613	T	C	3.04E-06	-0.184	0.040	0.049	0.059
rs78843608	A	G	1.85E-07	-0.168	0.032	-0.012	0.058
**CCL4 (F-statistic = 102.63, R2=13.20%)**							
rs10990301	A	T	4.88E-06	0.085	0.019	-0.011	0.025
rs111476793	A	G	4.79E-06	0.087	0.019	0.016	0.028
rs112315344	T	G	3.07E-06	0.212	0.046	0.078	0.076
rs113341849	A	G	1.00E-200	0.565	0.018	0.023	0.025
rs1161092	A	G	3.77E-07	-0.064	0.013	-0.018	0.017
rs116193969	A	T	2.31E-06	0.292	0.062	0.010	0.067
rs116309267	C	G	1.37E-06	0.196	0.041	0.002	0.043

Supplementary Table 1. Details of systemic inflammatory proteins predicting SNPs with Hashimoto's Thyroiditis from GWAS (with SNPs reaching $P < 5\times10^{-6}$)

Inflammatory proteins	Hashimoto's Thyroiditis						
SNP	Effect allele	Other allele	P	Beta	Standard Error	log(OR)	Standard Error
rs137896358	T	C	3.85E-07	0.280	0.055	0.018	0.066
rs140505982	T	C	3.24E-08	-0.229	0.042	-0.055	0.087
rs143960014	A	G	2.67E-06	0.195	0.042	-0.039	0.129
rs1634503	A	G	6.72E-08	-0.075	0.014	0.011	0.021
rs17692864	T	C	2.38E-16	0.232	0.028	-0.030	0.041
rs42279	T	C	4.51E-06	0.074	0.016	-0.015	0.022
rs4817032	A	T	4.50E-06	-0.090	0.020	-0.125	0.142
rs56131468	A	G	2.87E-06	0.083	0.018	-0.029	0.021
rs5743614	T	C	3.56E-07	0.073	0.014	0.016	0.024
rs72800563	T	G	2.37E-06	-0.177	0.037	0.120	0.086
rs78044056	A	G	2.33E-06	-0.261	0.055	-0.030	0.094
rs8064426	A	G	2.11E-124	-0.410	0.017	-0.015	0.021
**CD40L (F-statistic = 83.3, R2=9.56%)**							
rs117191103	T	C	4.28E-16	0.355	0.044	0.042	0.052
rs12152184	A	G	1.21E-07	0.109	0.021	-0.043	0.026
rs12500563	T	G	4.76E-06	0.314	0.069	-0.175	0.179
rs12624433	A	G	1.00E-200	-0.419	0.013	0.042	0.019
rs138739860	T	G	3.33E-06	0.163	0.035	-0.513	0.271
rs138897138	A	G	4.50E-07	0.109	0.022	0.032	0.031
rs3967170	A	G	3.46E-06	0.079	0.017	0.040	0.022
rs404842	A	G	1.12E-06	-0.060	0.012	0.001	0.017
rs4278412	C	G	1.67E-06	0.198	0.041	0.030	0.043
rs45456397	A	C	4.89E-07	-0.116	0.023	-0.031	0.031
rs4664334	T	C	4.55E-06	0.054	0.012	0.005	0.017
rs551896428	T	C	3.39E-06	-0.328	0.071	-0.044	0.602
rs7250371	T	C	3.34E-09	0.069	0.012	-0.004	0.018
rs75626299	C	G	3.95E-06	0.146	0.032	0.028	0.048
rs8024256	A	G	3.41E-06	-0.056	0.012	0.009	0.018
rs9526031	A	C	9.64E-08	0.064	0.012	0.017	0.017
rs9993874	A	G	3.22E-06	-0.058	0.013	0.024	0.017
**CD5 (F-statistic = 31.33, R2=4.54%)**							

Supplementary Table 1. Details of systemic inflammatory proteins predicting SNPs with Hashimoto's Thyroiditis from GWAS (with SNPs reaching *P*< 5×10^{-6})

Inflammatory proteins	Hashimoto's Thyroiditis						
SNP	Effect allele	Other allele	P	Beta	Standard Error	log(OR)	Standard Error
rs11622090	T	C	4.35E-06	-0.059	0.013	0.004	0.019
rs117276991	A	G	8.40E-07	-0.126	0.026	0.012	0.029
rs117516652	A	G	6.12E-07	0.237	0.048	-0.054	0.065
rs12416520	A	G	1.89E-06	-0.063	0.013	0.026	0.020
rs1259355	A	G	1.74E-06	0.057	0.012	0.024	0.017
rs138861398	A	C	1.05E-07	-0.080	0.015	-0.062	0.105
rs17345759	A	C	3.68E-06	-0.113	0.025	0.030	0.035
rs17769062	T	C	9.38E-07	0.164	0.033	-0.072	0.051
rs192582121	A	T	2.31E-07	-0.417	0.081	0.045	0.160
rs2237450	T	C	5.06E-07	0.062	0.012	0.002	0.018
rs4073745	A	G	1.85E-07	-0.068	0.013	-0.026	0.019
rs4893	A	G	2.61E-06	0.147	0.031	-0.095	0.045
rs4939490	C	G	1.80E-19	0.108	0.012	0.022	0.018
rs563284470	A	G	2.76E-06	0.183	0.039	-0.389	0.266
rs60562286	A	T	1.79E-06	-0.168	0.035	0.041	0.047
rs61747728	T	C	4.06E-09	0.184	0.031	-0.026	0.035
rs61768480	A	G	2.27E-06	0.070	0.015	-0.061	0.019
rs674379	C	G	2.85E-21	0.119	0.013	-0.055	0.018
rs7242462	A	G	1.44E-10	-0.120	0.019	0.008	0.024
rs72821405	T	C	2.97E-06	-0.101	0.022	-0.024	0.030
**CD6 (F-statistic = 130.32, R2=13.65%)**							
rs11230553	T	C	1.00E-200	-0.507	0.013	0.000	0.021
rs115830154	A	G	8.78E-07	-0.083	0.017	-0.010	0.021
rs12074680	A	G	2.22E-06	-0.186	0.039	0.017	0.057
rs12288280	T	G	2.68E-62	-0.355	0.021	-0.085	0.037
rs143374617	T	C	7.97E-08	0.177	0.033	0.068	0.044
rs35086654	T	C	2.97E-06	-0.242	0.052	0.051	0.150
rs35634144	C	G	3.17E-06	0.175	0.038	-0.092	0.088
rs414633	C	G	9.80E-07	0.066	0.013	0.031	0.019
rs4939458	A	G	4.31E-07	-0.065	0.013	0.015	0.017
rs534268736	T	C	6.32E-07	0.291	0.058	-0.022	0.381

Supplementary Table 1. Details of systemic inflammatory proteins predicting SNPs with Hashimoto's Thyroiditis from GWAS (with SNPs reaching $P < 5\times10^{-6}$)

Inflammatory proteins	Hashimoto's Thyroiditis						
SNP	Effect allele	Other allele	P	Beta	Standard Error	log(OR)	Standard Error
rs60988380	T	C	7.49E-07	0.096	0.019	-0.023	0.025
rs72969761	A	T	1.78E-06	-0.180	0.038	-0.064	0.038
rs74886615	A	T	9.64E-08	0.069	0.013	-0.008	0.017
rs8051133	A	G	4.83E-06	-0.108	0.024	-0.038	0.038
rs841332	A	G	2.84E-06	-0.072	0.015	0.011	0.021
rs9691968	A	C	1.43E-06	0.065	0.013	0.011	0.020
**CDCP1 (F-statistic = 50.82, R2=8.63%)**							
rs10044847	C	G	5.55E-07	-0.078	0.016	-0.006	0.021
rs111476793	A	G	8.76E-07	0.090	0.018	0.016	0.028
rs118116993	A	T	1.19E-06	0.096	0.020	0.034	0.024
rs12290068	A	G	1.40E-23	0.122	0.012	0.029	0.018
rs12928188	A	T	9.09E-07	-0.066	0.013	-0.020	0.021
rs146583535	A	G	2.51E-06	0.456	0.097	0.093	0.048
rs17078000	A	G	1.96E-06	0.061	0.013	-0.054	0.073
rs191929998	A	G	2.15E-06	-0.194	0.041	-0.027	0.064
rs2276862	C	G	1.14E-95	0.303	0.015	-0.001	0.021
rs28535751	T	C	4.55E-06	-0.054	0.012	0.021	0.017
rs34027772	T	C	1.20E-07	0.076	0.014	0.040	0.068
rs35478654	T	C	2.10E-06	-0.148	0.031	-0.022	0.042
rs4804148	T	C	7.35E-07	-0.062	0.013	-0.008	0.018
rs544425471	C	G	3.88E-06	0.388	0.084	-0.073	0.050
rs573808300	A	G	5.59E-07	-0.300	0.060	-0.222	0.117
rs59872649	A	G	2.46E-06	-0.109	0.023	-0.004	0.028
rs61782461	T	C	3.84E-06	-0.218	0.047	0.048	0.103
rs6452820	A	G	3.46E-06	-0.074	0.016	-0.009	0.020
rs672806	A	G	2.00E-12	0.082	0.012	-0.005	0.017
rs6991332	T	C	1.14E-06	-0.058	0.012	-0.023	0.017
rs74811341	T	C	1.79E-06	-0.132	0.028	-0.025	0.038
rs7506429	A	C	3.09E-07	0.074	0.014	-0.012	0.021
rs7618923	T	C	1.18E-30	-0.174	0.015	0.004	0.022
rs9272226	T	C	2.64E-15	-0.093	0.012	-0.054	0.018

Supplementary Table 1. Details of systemic inflammatory proteins predicting SNPs with Hashimoto's Thyroiditis from GWAS (with SNPs reaching *P*< 5×10^{-6})

Inflammatory proteins	Hashimoto's Thyroiditis						
SNP	Effect allele	Other allele	P	Beta	Standard Error	log(OR)	Standard Error
**CS3CL1 (F-statistic = 26.51, R2=4.26%)**							
rs10778675	A	C	5.37E-07	0.079	0.016	-0.001	0.022
rs111472651	C	G	1.57E-06	-0.119	0.025	0.053	0.039
rs114359340	A	G	3.41E-06	-0.188	0.041	0.042	0.078
rs11682107	A	G	8.45E-08	-0.068	0.013	-0.017	0.017
rs117698194	A	G	3.83E-06	-0.200	0.043	0.170	0.074
rs12331618	A	G	3.30E-07	-0.058	0.011	-0.019	0.017
rs13167439	T	C	2.89E-06	-0.090	0.019	0.032	0.025
rs1976172	C	G	2.12E-06	-0.057	0.012	-0.002	0.017
rs2731674	T	G	1.04E-07	-0.068	0.013	-0.012	0.019
rs35026817	T	G	2.12E-07	0.063	0.012	-0.016	0.018
rs3732378	A	G	9.94E-10	0.094	0.015	0.044	0.023
rs530057572	T	G	4.29E-06	-0.226	0.049	-0.615	0.468
rs55896747	T	C	3.71E-06	0.113	0.024	-0.025	0.043
rs56397903	A	G	3.46E-06	0.197	0.042	-0.106	0.041
rs571946532	A	C	2.66E-06	-0.240	0.051	-0.037	0.068
rs635634	T	C	5.29E-12	-0.115	0.017	0.003	0.020
rs6937696	T	C	1.40E-11	-0.081	0.012	0.032	0.026
rs712137	A	C	2.71E-06	0.056	0.012	0.012	0.018
rs73137770	C	G	3.91E-06	0.244	0.053	-0.041	0.077
rs77915710	A	G	2.32E-06	-0.144	0.030	-0.100	0.077
rs8052631	A	G	2.19E-08	-0.151	0.027	0.012	0.040
rs980364	A	G	8.94E-07	0.148	0.030	0.426	0.207
**CST5 (F-statistic = 65.18, R2=14.83%)**							
rs11054069	T	C	6.07E-38	0.167	0.013	-0.024	0.018
rs112961631	T	C	1.46E-32	0.566	0.048	-0.045	0.068
rs12294339	T	C	2.70E-09	0.070	0.012	-0.008	0.018
rs13127297	A	G	3.19E-06	0.055	0.012	0.016	0.017
rs13397003	T	G	6.64E-07	0.070	0.014	-0.023	0.021
rs139918398	T	C	6.05E-07	0.337	0.068	0.020	0.125
rs140505821	A	G	2.13E-06	0.328	0.069	-0.047	0.073

Supplementary Table 1. Details of systemic inflammatory proteins predicting SNPs with Hashimoto's Thyroiditis from GWAS (with SNPs reaching $P< 5\times10^{-6}$)

Inflammatory proteins	Hashimoto's Thyroiditis						
SNP	Effect allele	Other allele	P	Beta	Standard Error	log(OR)	Standard Error
rs141179525	T	C	7.54E-09	-0.258	0.045	-0.034	0.071
rs141192864	T	C	1.84E-06	0.102	0.021	-0.004	0.029
rs150547	A	C	4.12E-06	0.055	0.012	0.025	0.017
rs1536565	A	G	2.45E-06	-0.124	0.026	0.006	0.030
rs17154027	A	G	4.00E-06	-0.056	0.012	0.014	0.017
rs1797042	T	C	1.04E-36	-0.228	0.018	-0.026	0.028
rs188169560	T	C	5.90E-16	-0.310	0.038	-0.211	0.275
rs3889401	A	G	3.60E-06	0.089	0.019	-0.023	0.021
rs4507745	T	C	2.98E-06	0.057	0.012	-0.035	0.017
rs4640452	A	G	1.00E-200	0.355	0.012	-0.025	0.017
rs470841	T	C	1.81E-06	0.057	0.012	0.003	0.017
rs4756151	A	C	4.63E-08	-0.094	0.017	-0.012	0.024
rs4766797	A	G	9.30E-07	-0.057	0.012	-0.023	0.017
rs60586299	T	C	1.06E-06	-0.065	0.013	0.015	0.019
rs6083489	A	G	6.06E-07	0.237	0.047	0.293	0.118
rs62436191	A	G	3.35E-07	0.119	0.023	-0.047	0.041
rs67020211	T	G	6.44E-12	0.079	0.012	-0.004	0.017
rs697699	A	C	3.61E-06	0.110	0.024	-0.005	0.034
rs7275493	T	C	1.53E-06	-0.057	0.012	-0.002	0.018
rs72980022	A	G	1.45E-06	0.061	0.013	-0.006	0.018
rs77121303	A	G	2.25E-06	-0.252	0.053	-0.142	0.091
rs79359257	T	C	2.88E-06	0.073	0.016	0.009	0.022
rs930735	A	G	3.80E-07	0.058	0.011	-0.001	0.017
rs9327707	T	C	5.73E-07	0.059	0.012	0.005	0.017
rs9455923	A	G	3.02E-06	0.054	0.012	-0.011	0.017
rs962720	T	C	2.74E-06	0.056	0.012	0.003	0.016
**CXCL1 (F-statistic =66.74, R2=5.49%)**							
rs10734292	A	G	4.09E-06	0.123	0.027	-0.001	0.033
rs11880837	A	G	4.42E-06	0.058	0.013	-0.046	0.017
rs12770839	A	C	2.71E-07	0.065	0.013	-0.088	0.054
rs1366946	T	C	5.65E-121	-0.313	0.013	-0.015	0.019

Supplementary Table 1. Details of systemic inflammatory proteins predicting SNPs with Hashimoto's Thyroiditis from GWAS (with SNPs reaching $P< 5\times10^{-6}$)

Inflammatory proteins	Hashimoto's Thyroiditis						
SNP	Effect allele	Other allele	P	Beta	Standard Error	log(OR)	Standard Error
rs2122155	A	G	4.26E-06	-0.054	0.012	-0.023	0.016
rs2280441	A	G	3.75E-06	-0.144	0.031	0.011	0.043
rs2835916	C	G	2.60E-06	-0.056	0.012	-0.009	0.017
rs4474574	T	C	2.79E-06	-0.365	0.078	-0.620	0.640
rs62311721	T	C	4.88E-06	-0.186	0.041	0.013	0.038
rs67797811	C	G	4.39E-06	0.082	0.018	-0.118	0.133
rs77914035	A	G	3.72E-07	-0.105	0.021	-0.238	0.150
rs863004	T	C	2.78E-08	0.066	0.012	-0.067	0.073
**CXCL10 (F-statistic = 28.19, R2=3.73%)**							
rs10481754	T	C	4.12E-06	-0.055	0.012	-0.019	0.016
rs1077965	A	G	3.71E-06	-0.055	0.012	0.009	0.017
rs112202639	A	G	1.68E-06	-0.161	0.034	-0.048	0.041
rs12155428	T	C	2.52E-06	-0.177	0.038	-0.104	0.100
rs12174864	A	G	1.51E-06	-0.058	0.012	-0.003	0.017
rs12476448	T	C	3.15E-06	0.054	0.012	0.043	0.017
rs12629593	T	G	1.37E-06	-0.088	0.018	-0.001	0.028
rs12646113	T	C	6.78E-13	0.098	0.014	0.036	0.019
rs143796249	C	G	4.03E-06	-0.286	0.062	-0.042	0.050
rs150519056	T	C	9.77E-07	-0.184	0.038	0.032	0.047
rs1563171	A	G	3.14E-06	0.059	0.013	-0.017	0.018
rs2349689	A	G	1.50E-06	-0.079	0.016	-0.023	0.022
rs4367871	T	C	1.89E-06	-0.057	0.012	-0.017	0.017
rs4804148	T	C	1.31E-06	-0.063	0.013	-0.008	0.018
rs55732300	A	G	2.60E-06	0.118	0.025	-0.070	0.046
rs71534595	T	C	7.99E-07	-0.299	0.061	-0.207	0.642
rs72651343	T	C	6.98E-23	-0.610	0.062	-0.106	0.099
rs72682331	A	G	1.88E-07	0.109	0.021	0.002	0.029
rs741344	A	G	3.92E-06	-0.060	0.013	0.022	0.017
**CXCL11 (F-statistic = 33.83, R2=5.60%)**							
rs10733789	T	C	1.39E-10	-0.082	0.013	-0.014	0.018
rs10898158	A	G	4.83E-07	0.061	0.012	-0.030	0.018

Supplementary Table 1. Details of systemic inflammatory proteins predicting SNPs with Hashimoto's Thyroiditis from GWAS (with SNPs reaching $P< 5×10^{-6}$)

Inflammatory proteins	Hashimoto's Thyroiditis						
SNP	Effect allele	Other allele	P	Beta	Standard Error	log(OR)	Standard Error
rs111787182	A	C	4.19E-06	-0.145	0.031	-0.003	0.037
rs111848253	A	G	3.35E-06	-0.116	0.025	-0.179	0.182
rs112884138	T	C	3.01E-06	-0.098	0.021	0.019	0.039
rs116470923	T	C	3.74E-06	-0.198	0.043	0.009	0.109
rs12433317	A	G	1.95E-06	0.057	0.012	0.024	0.017
rs191536201	A	G	1.18E-06	-0.332	0.068	0.146	0.484
rs2245786	A	G	4.82E-06	-0.060	0.013	-0.009	0.018
rs2894233	A	G	1.96E-06	-0.224	0.047	0.041	0.068
rs4748380	T	C	2.12E-06	0.090	0.019	-0.018	0.028
rs59148364	A	G	1.45E-06	0.182	0.038	-0.058	0.046
rs60141220	A	G	2.49E-06	-0.078	0.017	-0.042	0.023
rs62291421	C	G	2.44E-07	0.267	0.052	-0.056	0.124
rs6827617	A	G	2.42E-48	-0.172	0.012	0.013	0.017
rs7009937	T	C	2.60E-06	0.118	0.025	0.009	0.037
rs7222922	T	C	1.26E-06	-0.088	0.018	0.056	0.027
rs72747175	A	G	2.49E-06	0.154	0.033	-0.017	0.047
rs72925566	A	G	1.44E-07	-0.097	0.019	-0.002	0.021
rs7475335	A	C	1.89E-07	-0.065	0.012	0.005	0.018
rs75612655	T	C	3.16E-14	0.535	0.071	0.708	0.300
rs7750544	T	C	2.44E-06	-0.077	0.016	0.008	0.024
rs78632565	T	C	4.28E-07	0.108	0.021	-0.031	0.026
**CXCL5 (F-statistic = 80.76, R2=8.28%)**							
rs10403201	A	G	2.22E-06	-0.060	0.013	-0.004	0.019
rs10821552	A	C	3.97E-13	-0.113	0.016	-0.004	0.017
rs11734099	A	G	5.00E-07	0.077	0.015	0.009	0.021
rs140828666	A	G	4.70E-06	-0.147	0.032	0.022	0.140
rs146553494	A	G	1.00E-06	0.184	0.038	0.073	0.075
rs2004100	T	C	4.39E-06	0.068	0.015	-0.030	0.020
rs2647400	C	G	1.21E-06	0.063	0.013	0.026	0.017
rs3183927	A	G	3.13E-07	-0.062	0.012	-0.007	0.017
rs425535	T	C	3.52E-172	0.506	0.018	0.042	0.027

Supplementary Table 1. Details of systemic inflammatory proteins predicting SNPs with Hashimoto's Thyroiditis from GWAS (with SNPs reaching $P < 5\times10^{-6}$)

Inflammatory proteins	Hashimoto's Thyroiditis						
SNP	Effect allele	Other allele	P	Beta	Standard Error	log(OR)	Standard Error
rs4279906	T	C	7.15E-07	0.208	0.042	0.025	0.043
rs59312158	A	G	8.01E-07	-0.190	0.039	-0.053	0.063
rs6993770	A	T	4.61E-15	-0.106	0.014	0.015	0.019
rs7636889	A	G	2.07E-08	-0.077	0.014	0.017	0.020
rs77914035	A	G	1.20E-08	-0.117	0.021	-0.238	0.150
rs79649467	T	G	4.50E-10	-0.172	0.028	-0.028	0.030
**CXCL6 (F-statistic = 117.32, R2=9.44%)**							
rs10021230	A	T	1.30E-17	0.118	0.014	0.014	0.019
rs10031141	A	G	1.00E-200	-0.392	0.012	-0.004	0.017
rs11547311	T	C	2.31E-06	0.129	0.027	-0.084	0.053
rs117109857	C	G	4.85E-06	0.190	0.042	-0.072	0.091
rs12075	A	G	1.29E-12	0.082	0.012	-0.019	0.017
rs182381042	A	G	1.63E-06	-0.383	0.080	-0.437	0.278
rs184103539	T	C	4.95E-06	-0.160	0.035	-0.017	0.033
rs34080302	T	G	3.87E-06	0.061	0.013	0.047	0.054
rs4877818	T	G	3.10E-06	0.063	0.013	0.084	0.098
rs4951518	T	G	2.54E-06	-0.215	0.046	-0.660	0.322
rs574432935	A	T	7.07E-07	0.343	0.069	0.104	0.077
rs9371716	T	C	1.92E-06	-0.092	0.019	0.018	0.023
**CXCL9 (F-statistic =29.16, R2=4.43%)**							
rs114033894	T	G	4.22E-06	-0.225	0.049	-0.005	0.043
rs12191307	A	G	1.70E-13	-0.145	0.020	-0.041	0.030
rs142119726	T	C	9.14E-07	0.271	0.055	0.042	0.056
rs145607633	T	C	2.28E-06	0.073	0.015	-0.002	0.023
rs182152471	T	C	1.34E-06	0.186	0.039	-0.010	0.042
rs192119373	T	G	1.59E-06	0.618	0.129	0.095	0.138
rs35435997	A	G	4.76E-06	0.092	0.020	-0.003	0.028
rs4241577	A	C	1.47E-28	-0.142	0.013	0.103	0.112
rs4888966	C	G	4.71E-06	-0.157	0.034	0.034	0.049
rs61875680	A	G	1.61E-06	-0.076	0.016	0.003	0.022
rs62200304	A	C	2.44E-06	-0.115	0.024	0.033	0.042

Supplementary Table 1. Details of systemic inflammatory proteins predicting SNPs with Hashimoto's Thyroiditis from GWAS (with SNPs reaching $P < 5\times10^{-6}$)

Inflammatory proteins	Hashimoto's Thyroiditis						
SNP	Effect allele	Other allele	P	Beta	Standard Error	log(OR)	Standard Error
rs62272916	A	G	2.15E-06	-0.083	0.018	-0.007	0.025
rs656739	A	G	2.33E-06	0.056	0.012	-0.016	0.017
rs73194812	A	G	3.20E-06	0.182	0.039	0.045	0.052
rs73367822	T	C	3.06E-06	-0.059	0.013	-0.064	0.096
rs73606147	A	C	2.22E-06	0.279	0.059	0.059	0.077
rs74956615	A	T	4.72E-06	-0.131	0.029	-0.197	0.046
rs783149	A	C	2.27E-08	-0.081	0.014	-0.010	0.021
rs80348644	T	C	3.21E-06	0.155	0.033	0.029	0.046
rs9366778	A	G	1.43E-07	-0.061	0.012	-0.047	0.018
**DNER (F-statistic = 48.5, R2=5.40%)**							
rs10191831	T	C	2.27E-06	-0.059	0.013	-0.013	0.017
rs10892466	C	G	3.89E-06	0.081	0.018	-0.013	0.026
rs1133851	A	G	2.92E-06	-0.066	0.014	0.008	0.020
rs116179073	T	C	3.84E-06	-0.248	0.054	0.753	0.384
rs117343254	C	G	2.94E-06	-0.128	0.027	0.020	0.033
rs13107325	T	C	4.76E-06	0.107	0.023	-0.105	0.069
rs194746	T	C	2.43E-06	0.053	0.011	-0.001	0.016
rs207671	A	G	3.83E-18	0.102	0.012	0.007	0.017
rs2837988	A	C	1.05E-06	0.058	0.012	0.006	0.018
rs28689968	T	C	6.07E-07	0.092	0.018	0.018	0.023
rs35032874	T	G	1.52E-23	0.132	0.013	0.003	0.018
rs62193248	A	T	3.50E-67	0.213	0.012	0.014	0.019
rs6517655	C	G	4.41E-09	0.076	0.013	-0.015	0.018
rs75685422	T	C	2.29E-06	0.088	0.019	-0.019	0.029
rs9289590	T	C	4.40E-06	-0.065	0.014	-0.066	0.060
rs9967959	T	G	2.32E-06	0.072	0.015	0.011	0.023
**EIF4E-BPI (F-statistic = 34.35, R2=2.66%)**							
rs10792025	T	C	1.73E-06	-0.059	0.012	0.026	0.018
rs10926313	A	G	4.39E-07	-0.088	0.018	0.009	0.022
rs137887001	C	G	4.25E-06	0.224	0.049	0.092	0.070
rs181095999	T	C	2.65E-06	-0.223	0.047	0.040	0.065

Supplementary Table 1. Details of systemic inflammatory proteins predicting SNPs with Hashimoto's Thyroiditis from GWAS (with SNPs reaching $P < 5\times10^{-6}$)

Inflammatory proteins	Hashimoto's Thyroiditis						
SNP	Effect allele	Other allele	P	Beta	Standard Error	log(OR)	Standard Error
rs2731672	T	C	1.11E-09	0.093	0.015	-0.011	0.019
rs2856226	T	C	3.33E-06	-0.100	0.021	0.009	0.031
rs356283	T	G	1.75E-06	0.059	0.012	0.022	0.019
rs3733402	A	G	2.32E-29	-0.131	0.012	0.019	0.016
rs5030072	T	C	5.99E-07	0.059	0.012	0.007	0.017
rs6963617	T	C	1.10E-07	0.075	0.014	-0.007	0.020
rs76686678	T	G	5.62E-07	0.261	0.052	-0.095	0.055
**Eotaxin (F-statistic = 40.64, R2=5.64%)**							
rs114744647	T	C	2.99E-06	0.211	0.045	-0.049	0.066
rs12075	A	G	5.03E-11	0.075	0.011	-0.019	0.017
rs139476527	T	G	3.11E-06	0.283	0.061	-0.074	0.172
rs139597204	A	C	2.30E-06	-0.337	0.071	-0.184	0.133
rs1564995	A	G	3.34E-06	-0.061	0.013	0.001	0.017
rs2112920	C	G	3.29E-07	0.082	0.016	0.004	0.022
rs2201150	T	C	1.75E-10	0.079	0.012	-0.021	0.017
rs2228467	T	C	3.69E-49	-0.332	0.023	-0.014	0.032
rs34536806	T	G	1.01E-06	0.102	0.021	-0.041	0.032
rs35543857	A	G	3.10E-06	-0.114	0.024	-0.041	0.029
rs554698477	A	G	2.56E-06	0.106	0.023	-0.095	0.159
rs6894241	A	C	1.68E-06	-0.066	0.014	0.011	0.019
rs73171545	A	G	4.14E-06	-0.086	0.019	0.049	0.024
rs73209015	A	T	1.36E-06	-0.091	0.019	0.017	0.029
rs757973	A	G	1.58E-22	0.200	0.021	0.023	0.034
rs79432525	T	G	4.85E-06	0.193	0.042	0.127	0.087
rs79684468	T	C	7.16E-07	0.150	0.030	0.046	0.042
rs79722574	T	C	6.78E-15	-0.122	0.016	0.022	0.024
rs9940390	T	C	7.73E-10	-0.082	0.013	0.026	0.018
rs9976282	A	G	1.15E-06	-0.168	0.035	0.069	0.056
**FGF19 (F-statistic =33.98, R2=5.00%)**							
rs10777251	A	G	3.83E-06	-0.077	0.017	0.016	0.020
rs112126444	A	G	3.19E-06	-0.093	0.020	-0.069	0.029

Supplementary Table 1. Details of systemic inflammatory proteins predicting SNPs with Hashimoto's Thyroiditis from GWAS (with SNPs reaching $P< 5\times10^{-6}$)

Inflammatory proteins	Hashimoto's Thyroiditis						
SNP	Effect allele	Other allele	P	Beta	Standard Error	log(OR)	Standard Error
rs11244038	C	G	1.21E-06	0.120	0.025	-0.021	0.024
rs117398067	T	C	4.65E-06	0.258	0.056	-0.057	0.051
rs12110436	T	C	4.67E-06	-0.136	0.030	0.034	0.042
rs12407505	A	G	4.53E-06	-0.161	0.035	0.067	0.056
rs12520287	T	C	4.47E-06	-0.101	0.022	-0.028	0.026
rs13103023	A	G	1.48E-11	0.084	0.012	-0.018	0.017
rs142067135	A	G	3.09E-06	-0.230	0.049	0.222	0.102
rs145274424	T	C	2.23E-06	0.199	0.042	0.005	0.068
rs1671812	T	C	2.54E-06	0.062	0.013	0.009	0.019
rs191522330	T	C	1.89E-06	0.233	0.049	-0.025	0.045
rs2306969	A	G	1.75E-06	0.067	0.014	-0.005	0.021
rs2906655	T	C	3.93E-06	-0.062	0.014	-0.028	0.020
rs35836706	A	G	2.39E-06	0.215	0.046	-0.114	0.055
rs516246	T	C	5.31E-45	-0.166	0.012	0.027	0.018
rs574352299	A	G	8.69E-07	0.237	0.048	0.324	0.316
rs7005978	A	G	9.72E-18	-0.106	0.012	0.014	0.017
rs76708593	T	C	3.98E-06	0.102	0.022	0.072	0.178
rs79110990	A	G	2.88E-06	0.073	0.016	0.010	0.021
rs834971	A	G	1.61E-06	-0.061	0.013	-0.006	0.018
**FGF21 (F-statistic = 30.92, R2=3.21%)**							
rs10495032	T	G	9.77E-07	-0.156	0.032	0.010	0.032
rs12290350	T	C	2.02E-06	-0.069	0.015	0.026	0.022
rs1260326	T	C	1.03E-28	0.132	0.012	0.027	0.017
rs13229619	A	G	6.13E-20	-0.161	0.018	0.013	0.025
rs142603673	T	C	2.35E-07	-0.235	0.046	-0.107	0.091
rs145127946	A	G	1.22E-06	-0.175	0.036	-0.027	0.092
rs188758663	A	G	3.88E-07	-0.380	0.075	0.060	0.127
rs2429473	A	C	3.32E-08	-0.081	0.015	-0.006	0.021
rs4700382	T	C	3.62E-06	-0.063	0.014	0.013	0.019
rs60277384	T	C	3.54E-06	-0.088	0.019	0.008	0.023
rs7012637	A	G	4.61E-07	-0.061	0.012	0.004	0.017

Supplementary Table 1. Details of systemic inflammatory proteins predicting SNPs with Hashimoto's Thyroiditis from GWAS (with SNPs reaching $P < 5\times10^{-6}$)

Inflammatory proteins	Hashimoto's Thyroiditis						
SNP	Effect allele	Other allele	P	Beta	Standard Error	log(OR)	Standard Error
rs72965996	A	G	6.08E-07	-0.222	0.044	0.082	0.060
rs7610704	T	C	2.21E-06	-0.057	0.012	0.003	0.016
rs838131	A	C	6.15E-36	0.163	0.013	0.015	0.017
**FGF23 (F-statistic = 28.01, R2=3.55%)**							
rs11150539	T	C	2.53E-06	-0.056	0.012	-0.019	0.016
rs118177380	A	G	3.18E-06	0.236	0.051	-0.046	0.093
rs11930290	A	T	2.11E-06	-0.185		0.048	0.072
rs12195292	A	G	3.72E-06	-0.077	0.017	0.002	0.024
rs181409232	A	C	3.43E-06	-0.222	0.048	-0.056	0.070
rs33921462	A	G	1.32E-06	-0.065	0.014	0.002	0.017
rs34551523	A	G	9.75E-07	0.170	0.035	0.026	0.049
rs35590112	T	C	1.32E-06	0.080	0.017	-0.134	0.127
rs368215473	A	G	1.77E-06	0.123	0.026	-0.047	0.034
rs370203222	C	G	1.84E-06	0.106	0.022	-0.134	0.081
rs3811621	C	G	6.82E-19	0.116	0.013	-0.060	0.018
rs6127099	A	T	1.30E-11	0.091	0.013	-0.032	0.019
rs671205	T	C	1.93E-07	0.071	0.014	0.013	0.018
rs6753743	T	G	3.65E-07	0.094	0.019	-0.029	0.027
rs75338333	A	G	3.89E-06	-0.109	0.024	-0.013	0.071
rs794007	T	C	1.97E-06	0.069	0.014	0.026	0.021
rs7955866	A	G	7.14E-08	-0.096	0.018	-0.002	0.026
**FGF5 (F-statistic = 73.87, R2=14.97%)**							
rs11120686	T	C	2.40E-06	-0.060	0.013	0.019	0.017
rs112790107	A	G	1.64E-06	0.509	0.106	0.052	0.052
rs11674185	A	G	4.69E-06	-0.059	0.013	-0.004	0.017
rs117253850	T	C	5.67E-07	0.227	0.045	0.029	0.081
rs12005489	T	C	2.78E-06	0.151	0.032	-0.009	0.038
rs1412253	A	G	2.20E-06	-0.091	0.019	0.015	0.027
rs144059387	A	G	2.04E-14	0.454	0.059	0.042	0.079
rs148332905	T	C	2.12E-06	0.288	0.061	0.069	0.072
rs148797749	A	G	2.32E-06	-0.171	0.036	0.068	0.041

Supplementary Table 1. Details of systemic inflammatory proteins predicting SNPs with Hashimoto's Thyroiditis from GWAS (with SNPs reaching $P< 5×10^{-6}$)

Inflammatory proteins	Hashimoto's Thyroiditis						
SNP	Effect allele	Other allele	P	Beta	Standard Error	log(OR)	Standard Error
rs150524893	A	G	3.71E-06	0.255	0.055	0.023	0.065
rs189846846	T	C	3.91E-06	-0.304	0.066	0.001	0.188
rs1902859	T	C	1.00E-200	-0.439	0.013	-0.044	0.017
rs192746810	A	G	3.11E-06	-0.184	0.040	-0.052	0.044
rs305897	T	C	1.95E-06	0.119	0.025	0.035	0.033
rs369482528	A	G	3.51E-06	-0.099	0.021	-0.001	0.067
rs4326078	A	C	1.21E-06	-0.073	0.015	-0.006	0.020
rs4970834	T	C	2.56E-06	-0.073	0.016	-0.007	0.021
rs56373850	A	T	7.01E-14	0.183	0.024	-0.036	0.024
rs56391958	A	G	1.53E-06	-0.120	0.025	-0.017	0.028
rs62298642	A	G	4.66E-08	0.113	0.021	0.077	0.033
rs624605	T	G	3.25E-06	0.062	0.013	-0.005	0.017
rs66613644	T	C	5.56E-07	-0.083	0.017	0.017	0.022
rs75140224	A	G	2.85E-06	0.146	0.031	0.019	0.045
rs77210320	A	T	2.61E-06	0.167	0.036	0.037	0.041
rs9433760	A	G	4.95E-06	0.102	0.022	0.004	0.030
**FLT3L (F-statistic = 30.38, R2=5.81%)**							
rs10164255	T	G	4.33E-06	0.056	0.012	-0.015	0.017
rs11212636	A	G	4.16E-11	-0.077	0.012	0.008	0.017
rs12338549	T	C	4.76E-06	-0.085	0.019	0.043	0.023
rs1379304	T	C	2.05E-06	0.060	0.013	-0.009	0.017
rs141117375	T	C	4.45E-06	-0.152	0.033	0.064	0.067
rs142870956	T	C	1.07E-06	-0.431	0.088	-0.302	0.587
rs144317085	A	T	9.85E-12	0.221	0.032	-0.049	0.037
rs17272847	A	G	8.36E-08	0.118	0.022	0.001	0.026
rs185979634	T	C	1.26E-06	0.148	0.031	0.004	0.066
rs1866051	T	C	8.76E-11	-0.075	0.012	-0.008	0.017
rs192857133	T	C	2.68E-06	0.181	0.039	0.062	0.064
rs218265	T	C	1.93E-07	0.083	0.016	0.032	0.022
rs28533776	A	G	3.48E-07	-0.075	0.015	0.006	0.022
rs55633025	A	G	1.91E-08	0.073	0.013	-0.021	0.017

Supplementary Table 1. Details of systemic inflammatory proteins predicting SNPs with Hashimoto's Thyroiditis from GWAS (with SNPs reaching *P*< 5×10⁻⁶)

Inflammatory proteins	Hashimoto's Thyroiditis						
SNP	Effect allele	Other allele	P	Beta	Standard Error	log(OR)	Standard Error
rs57191857	A	G	1.76E-06	-0.143	0.030	0.066	0.042
rs575588389	A	G	2.50E-06	-0.090	0.019	0.011	0.135
rs61792394	A	G	3.45E-06	-0.157	0.034	0.008	0.045
rs6453962	T	C	1.77E-06	-0.056	0.012	0.041	0.052
rs72681653	A	G	9.84E-07	0.130	0.027	0.030	0.043
rs73000965	A	T	2.55E-06	0.059	0.013	-0.005	0.017
rs74722523	T	C	3.43E-06	0.353	0.076	0.374	0.197
rs7624160	T	G	4.84E-19	-0.106	0.012	0.023	0.017
rs7625643	A	G	2.18E-06	0.061	0.013	-0.042	0.017
rs7726159	A	C	1.34E-20	-0.119	0.013	-0.016	0.018
rs78429945	T	G	3.29E-08	0.088	0.016	-0.052	0.019
rs9381118	T	C	2.27E-06	0.064	0.014	0.012	0.019
**GDNF (F-statistic =52.2, R2=5.84%)**							
rs112594435	T	C	4.27E-06	0.153	0.033	0.002	0.063
rs11740708	A	G	7.56E-86	-0.389	0.020	0.033	0.032
rs138691533	A	G	1.63E-06	0.258	0.054	0.039	0.111
rs144181640	A	T	1.37E-06	0.337	0.070	-0.066	0.076
rs199503547	A	T	2.60E-07	0.113	0.022	-0.091	0.149
rs2093446	C	G	2.63E-06	0.061	0.013	0.003	0.017
rs2194229	A	T	5.34E-21	-0.168	0.018	0.034	0.024
rs2453338	A	T	2.30E-11	0.083	0.012	0.028	0.017
rs35740453	A	G	3.13E-06	-0.072	0.015	-0.025	0.019
rs540456481	T	G	1.80E-06	-0.294	0.062	0.976	0.463
rs55850217	T	C	5.59E-09	0.082	0.014	-0.201	0.105
rs57104839	A	G	3.04E-06	0.092	0.020	0.016	0.030
rs62243355	T	C	5.58E-07	-0.098	0.020	0.020	0.027
rs77671113	A	C	9.45E-09	-0.119	0.021	-0.019	0.031
rs79953230	T	C	4.96E-06	0.192	0.042	-0.005	0.045
rs9303102	T	G	3.71E-06	0.210	0.045	0.058	0.116
**HGF (F-statistic = 28.56, R2=4.04%)**							
rs113903858	A	G	9.22E-07	0.367	0.075	0.044	0.207

Supplementary Table 1. Details of systemic inflammatory proteins predicting SNPs with Hashimoto's Thyroiditis from GWAS (with SNPs reaching $P < 5\times10^{-6}$)

Inflammatory proteins	Hashimoto's Thyroiditis						
SNP	Effect allele	Other allele	P	Beta	Standard Error	log(OR)	Standard Error
rs115009171	A	G	4.21E-06	0.430	0.093	-0.500	0.825
rs138848271	T	C	1.13E-06	-0.405	0.083	-0.017	0.207
rs150498795	A	T	1.68E-06	0.157	0.033	-0.016	0.079
rs1986719	T	C	5.44E-07	-0.195	0.039	-0.057	0.053
rs2281532	T	C	3.79E-06	-0.070	0.015	0.002	0.021
rs2737756	A	G	1.65E-07	0.205	0.039	-0.104	0.068
rs34219417	T	C	4.84E-06	0.099	0.022	-0.011	0.025
rs4290523	T	C	4.84E-06	-0.099	0.022	0.092	0.078
rs5007364	T	C	2.75E-06	0.077	0.016	0.045	0.122
rs57193877	T	C	4.31E-07	0.157	0.031	-0.003	0.057
rs5745687	T	C	9.83E-16	-0.194	0.024	0.012	0.043
rs5745692	C	G	3.26E-06	-0.155	0.033	-0.100	0.053
rs59950280	A	G	1.34E-20	0.122	0.013	0.013	0.019
rs6709159	A	G	7.52E-07	0.056	0.011	-0.014	0.017
rs74130123	T	C	6.79E-07	-0.243	0.049	0.035	0.049
rs7620568	A	G	1.73E-06	-0.086	0.018	0.011	0.022
rs8106345	A	G	3.14E-06	0.059	0.013	0.017	0.017
rs851612	A	G	4.24E-06	-0.118	0.026	0.072	0.044
**IL-10 (F-statistic = 29.42, R2=3.57%)**							
rs10067443	T	C	4.33E-07	-0.075	0.015	0.001	0.022
rs12123181	A	G	4.22E-11	-0.100	0.015	-0.002	0.021
rs138203589	T	G	3.59E-06	0.194	0.042	0.031	0.053
rs148332061	C	G	3.28E-06	0.111	0.024	-0.030	0.025
rs150408090	A	G	1.45E-06	0.367	0.076	0.019	0.052
rs17654493	T	C	2.43E-06	0.295	0.063	0.070	0.085
rs188535859	T	C	1.32E-06	-0.235	0.049	0.041	0.069
rs3135932	A	G	3.39E-25	-0.160	0.015	0.044	0.021
rs3769274	A	G	1.94E-06	0.077	0.016	0.011	0.023
rs385697	T	C	3.10E-06	0.056	0.012	-0.012	0.017
rs389239	A	G	4.39E-06	-0.057	0.013	-0.011	0.017
rs398518	A	G	3.01E-09	-0.129	0.022	-0.024	0.030

Supplementary Table 1. Details of systemic inflammatory proteins predicting SNPs with Hashimoto's Thyroiditis from GWAS (with SNPs reaching $P< 5\times10^{-6}$)

Inflammatory proteins	Hashimoto's Thyroiditis						
SNP	Effect allele	Other allele	P	Beta	Standard Error	log(OR)	Standard Error
rs4655953	A	G	3.02E-06	0.057	0.012	0.009	0.017
rs549056348	A	C	1.10E-06	0.165	0.034	-0.027	0.041
rs66624860	T	C	1.91E-06	0.068	0.014	0.001	0.017
rs72701782	A	G	3.33E-06	0.098	0.021	0.054	0.026
rs79438518	A	T	4.15E-06	-0.285	0.062	-0.653	0.211
**IL-10Rβ (F-statistic = 92.89, R2=11.76%)**							
rs10091841	T	G	2.13E-06	0.064	0.014	-0.011	0.017
rs143313574	T	C	4.24E-06	-0.387	0.084	-0.072	0.054
rs17832137	A	G	1.03E-06	0.168	0.034	0.023	0.038
rs180893431	A	C	2.73E-06	0.406	0.087	0.292	0.175
rs286605	T	G	1.98E-06	0.062	0.013	0.002	0.017
rs3135932	A	G	5.07E-07	-0.084	0.017	0.044	0.021
rs41272625	T	G	4.63E-06	0.082	0.018	0.042	0.028
rs6670799	A	G	7.48E-07	-0.125	0.025	-0.002	0.029
rs74719858	A	G	1.97E-06	0.392	0.082	-0.017	0.040
rs80137559	T	C	4.37E-06	0.235	0.051	0.035	0.053
rs111850948	A	G	3.20E-06	-0.084	0.018	-0.084	0.027
rs112798338	T	C	2.78E-06	0.193	0.041	-0.031	0.053
rs114288486	T	C	3.17E-06	0.145	0.031	0.078	0.061
rs1153572	T	C	4.12E-06	-0.055	0.012	0.028	0.016
rs116315732	T	C	7.60E-07	0.199	0.040	0.052	0.052
rs117883662	C	G	2.19E-06	-0.430	0.091	-0.049	0.051
rs142421172	A	G	1.95E-11	0.208	0.031	-0.023	0.035
rs149156511	A	G	3.27E-06	0.207	0.044	-0.035	0.054
rs192325200	A	G	4.07E-06	-0.233	0.051	-0.015	0.090
rs34361138	A	G	2.50E-06	0.053	0.011	-0.012	0.017
rs55780441	T	C	3.00E-07	0.178	0.035	-0.010	0.029
rs566450266	T	G	4.40E-06	0.065	0.014	0.099	0.070
rs6517157	T	C	1.83E-09	0.151	0.025	-0.035	0.038
rs6584485	T	C	3.93E-06	0.074	0.016	-0.029	0.029
rs75710908	A	G	3.30E-06	0.159	0.034	0.044	0.042

Supplementary Table 1. Details of systemic inflammatory proteins predicting SNPs with Hashimoto's Thyroiditis from GWAS (with SNPs reaching $P < 5×10^{-6}$)

Inflammatory proteins	Hashimoto's Thyroiditis						
SNP	Effect allele	Other allele	P	Beta	Standard Error	log(OR)	Standard Error
rs7574167	A	G	2.38E-06	0.064	0.014	-0.005	0.019
rs7855466	T	C	3.49E-07	0.086	0.017	-0.031	0.019
rs8178484	T	C	1.00E-200	-0.426	0.012	0.015	0.017
rs9685374	A	G	3.99E-06	-0.062	0.013	0.019	0.018
**IL-12p40 (F-statistic = 33.99, R2=5.99%)**							
rs11039216	T	C	4.00E-07	0.059	0.012	-0.003	0.017
rs11130215	A	G	2.36E-11	-0.101	0.015	-0.045	0.019
rs117888068	T	C	3.49E-06	0.219	0.047	0.004	0.061
rs12588969	C	G	1.74E-24	-0.130	0.013	-0.001	0.021
rs12766137	C	G	1.95E-06	0.071	0.015	-0.077	0.094
rs1398590	A	G	5.73E-08	-0.197	0.036	0.022	0.057
rs144850787	T	G	1.66E-08	0.316	0.056	0.022	0.095
rs148549227	T	C	1.82E-06	0.109	0.023	0.055	0.029
rs149993822	T	C	3.93E-07	0.098	0.019	0.089	0.148
rs17056751	A	G	1.87E-07	0.169	0.033	-0.032	0.036
rs17722688	T	C	6.07E-11	-0.086	0.013	-0.035	0.018
rs1950897	T	C	4.54E-10	0.080	0.013	0.055	0.018
rs3130510	A	G	1.58E-16	0.116	0.014	0.071	0.025
rs5014495	T	C	2.82E-06	0.207	0.044	0.010	0.037
rs516978	C	G	3.42E-09	0.072	0.012	0.019	0.017
rs539598	T	C	1.90E-07	-0.067	0.013	-0.018	0.020
rs6750065	T	C	2.78E-06	0.063	0.013	0.060	0.020
rs6968201	T	G	1.72E-07	-0.062	0.012	-0.014	0.017
rs73416208	A	G	1.92E-09	-0.148	0.025	-0.016	0.030
rs76428106	T	C	1.94E-10	-0.341	0.054	-0.196	0.078
rs780679	T	C	5.73E-07	-0.070	0.014	-0.041	0.019
rs8026767	A	G	1.81E-08	-0.074	0.013	-0.029	0.019
rs9965402	A	G	4.94E-06	0.092	0.020	-0.006	0.029
**IL-13 (F-statistic = 23.58, R2=2.84%)**							
rs10446322	A	G	1.24E-06	0.064	0.013	0.012	0.018
rs10893042	C	G	2.87E-06	-0.070	0.015	-0.018	0.017

Supplementary Table 1. Details of systemic inflammatory proteins predicting SNPs with Hashimoto's Thyroiditis from GWAS (with SNPs reaching $P < 5\times10^{-6}$)

Inflammatory proteins	Hashimoto's Thyroiditis						
SNP	Effect allele	Other allele	P	Beta	Standard Error	log(OR)	Standard Error
rs10917637	A	G	3.61E-07	0.069	0.014	-0.011	0.018
rs11251952	A	G	3.36E-06	-0.185	0.040	-0.021	0.030
rs12146346	T	G	2.19E-06	0.073	0.016	0.003	0.054
rs13119159	A	G	2.16E-08	0.074	0.013	0.001	0.016
rs141726773	T	C	2.69E-06	0.209	0.045	-0.021	0.043
rs149979178	A	G	2.63E-06	0.183	0.039	-0.025	0.033
rs183684201	C	G	2.29E-06	-0.297	0.063	-0.023	0.049
rs55924857	T	C	3.33E-07	-0.093	0.018	0.009	0.023
rs59235092	A	G	1.34E-06	0.212	0.044	0.042	0.045
rs76219628	A	G	2.20E-06	0.169	0.036	0.061	0.052
rs9881117	A	G	3.28E-06	-0.066	0.014	-0.038	0.017
**L-15Rα (F-statistic = 30.92, R2=3.21%)**							
rs117428573	A	C	4.67E-06	-0.313	0.068	0.092	0.072
rs117484888	A	G	2.05E-06	0.379	0.080	0.359	0.207
rs12570881	A	T	6.27E-07	-0.087	0.017	0.014	0.021
rs145793996	A	G	3.66E-06	-0.228	0.049	-0.093	0.048
rs192538756	A	T	1.52E-06	0.116	0.024	0.021	0.025
rs2228059	T	G	8.39E-186	0.369	0.013	-0.014	0.016
rs2836464	A	G	2.48E-06	0.080	0.017	0.058	0.023
rs4459616	T	C	1.92E-06	0.140	0.029	0.003	0.033
rs56240706	A	T	1.92E-06	0.092	0.019	0.048	0.131
rs649129	T	C	4.33E-06	-0.073	0.016	0.016	0.019
rs6845172	C	G	1.57E-06	0.078	0.016	-0.011	0.020
rs7898286	T	G	4.34E-48	-0.197	0.014	0.047	0.018
**IL-17A (F-statistic = 22.87, R2=2.66%)**							
rs115193412	T	C	1.32E-06	0.169	0.035	0.086	0.275
rs12599620	T	C	2.08E-06	-0.073	0.015	-0.043	0.018
rs1462017	C	G	1.50E-07	-0.089	0.017	-0.025	0.024
rs17448036	A	G	4.87E-06	-0.059	0.013	-0.010	0.016
rs2429911	T	C	2.02E-06	0.231	0.049	-0.018	0.082
rs28852438	A	G	4.34E-06	-0.068	0.015	0.004	0.018

Supplementary Table 1. Details of systemic inflammatory proteins predicting SNPs with Hashimoto's Thyroiditis from GWAS (with SNPs reaching $P < 5\times10^{-6}$)

Inflammatory proteins	Hashimoto's Thyroiditis						
SNP	Effect allele	Other allele	P	Beta	Standard Error	log(OR)	Standard Error
rs33998516	A	C	1.01E-06	-0.080	0.016	0.032	0.018
rs34557923	T	G	2.23E-06	0.245	0.052	-0.028	0.075
rs41270871	A	T	1.04E-06	0.092	0.019	-0.154	0.066
rs75788494	T	C	3.61E-06	-0.243	0.053	0.093	0.075
rs77529534	T	C	2.57E-06	-0.128	0.027	-0.003	0.026
rs77595989	T	G	4.84E-06	0.170	0.037	-0.042	0.036
rs79982350	C	G	6.60E-07	-0.147	0.030	-0.035	0.038
**IL-17C (F-statistic = 24.89, R2=4.48%)**							
rs1072074	A	G	4.17E-06	-0.065	0.014	-0.041	0.017
rs10870773	A	G	1.82E-06	-0.082	0.017	-0.009	0.019
rs114882743	A	T	2.73E-09	-0.326	0.055	-0.017	0.111
rs11931996	T	C	3.28E-06	-0.122	0.026	-0.024	0.035
rs12092228	T	G	4.00E-06	0.151	0.033	-0.016	0.035
rs12445031	C	G	3.92E-06	-0.090	0.020	-0.046	0.099
rs12863903	T	C	1.93E-06	-0.086	0.018	-0.020	0.022
rs12895022	T	C	3.78E-06	0.109	0.024	0.009	0.029
rs149756404	A	C	1.55E-07	-0.275	0.052	0.399	0.329
rs17700884	T	G	1.16E-14	-0.105	0.014	0.026	0.016
rs1920106	T	C	1.62E-06	0.063	0.013	-0.011	0.017
rs2814092	C	G	3.80E-06	0.062	0.014	-0.003	0.018
rs39779	C	G	2.09E-06	0.108	0.023	0.017	0.025
rs4593271	A	G	2.05E-06	-0.079	0.017	-0.088	0.101
rs554604740	A	G	1.56E-06	0.193	0.040	0.019	0.064
rs59356989	A	G	6.26E-07	-0.088	0.018	0.002	0.022
rs72815428	T	C	4.85E-06	-0.167	0.037	-0.055	0.071
rs75246422	T	C	2.23E-06	-0.095	0.020	0.011	0.024
rs77654242	T	G	3.56E-06	-0.217	0.047	0.040	0.052
rs8015296	C	G	4.37E-06	-0.116	0.025	0.005	0.031
**IL-18 (F-statistic = 42.13, R2=6.82%)**							
rs112338272	A	C	4.27E-06	-0.195	0.043	-0.509	0.313
rs113552426	C	G	1.71E-06	-0.278	0.058	-0.138	0.141

Supplementary Table 1. Details of systemic inflammatory proteins predicting SNPs with Hashimoto's Thyroiditis from GWAS (with SNPs reaching *P*< 5×10^{-6})

Inflammatory proteins	Hashimoto's Thyroiditis						
SNP	Effect allele	Other allele	P	Beta	Standard Error	log(OR)	Standard Error
rs11605127	C	G	4.41E-06	-0.063	0.014	0.029	0.017
rs12201187	A	T	9.65E-07	0.068	0.014	-0.012	0.017
rs12648080	A	G	8.16E-07	0.072	0.015	-0.014	0.022
rs12681624	C	G	2.59E-09	-0.094	0.016	0.029	0.020
rs12711729	T	C	4.47E-06	-0.070	0.015	-0.022	0.018
rs139760701	A	T	4.46E-07	-0.305	0.060	0.035	0.121
rs1403742	A	T	4.82E-06	-0.057	0.012	-0.010	0.017
rs148504635	A	C	1.42E-06	0.253	0.053	0.021	0.072
rs17229943	A	C	2.07E-09	-0.160	0.027	-0.019	0.028
rs2108621	T	C	4.01E-06	0.058	0.013	0.003	0.018
rs2954016	T	C	1.14E-06	-0.076	0.016	-0.008	0.018
rs35495214	T	C	2.34E-06	0.137	0.029	-0.119	0.063
rs385076	T	C	1.68E-51	-0.186	0.012	0.016	0.017
rs4140444	A	G	2.88E-06	0.216	0.046	0.016	0.064
rs567093451	T	G	4.66E-06	0.115	0.025	-0.413	0.154
rs56836641	T	C	6.12E-08	-0.163	0.030	0.015	0.031
rs5744249	A	C	1.53E-51	0.204	0.014	0.022	0.020
rs57954386	C	G	1.45E-06	-0.125	0.026	0.001	0.049
rs60235102	T	C	2.91E-07	0.115	0.023	-0.028	0.028
rs67424072	T	G	6.71E-07	-0.082	0.017	0.004	0.021
rs77994606	A	G	9.46E-07	-0.231	0.047	0.044	0.058
**IL-18R1 (F-statistic = 119.91, R2=18.00%)**							
rs116935210	A	G	3.13E-06	0.239	0.051	0.074	0.036
rs117145942	A	G	3.63E-06	-0.301	0.065	-0.011	0.116
rs117195704	A	G	1.52E-06	-0.151	0.031	0.059	0.055
rs117599131	A	C	2.89E-06	-0.148	0.032	-0.002	0.039
rs1760941	A	C	3.71E-06	0.066	0.014	0.001	0.021
rs2570479	A	C	1.01E-06	0.066	0.014	-0.001	0.017
rs2732865	A	C	3.70E-33	0.364	0.030	0.030	0.061
rs28929474	T	C	2.86E-09	0.264	0.045	0.020	0.059
rs34174304	A	G	9.62E-07	0.073	0.015	0.015	0.022

Supplementary Table 1. Details of systemic inflammatory proteins predicting SNPs with Hashimoto's Thyroiditis from GWAS (with SNPs reaching $P < 5\times10^{-6}$)

Inflammatory proteins	Hashimoto's Thyroiditis						
SNP	Effect allele	Other allele	P	Beta	Standard Error	log(OR)	Standard Error
rs35021151	A	G	1.49E-06	-0.165	0.034	-0.004	0.046
rs536613714	T	G	8.43E-07	0.191	0.039	-0.277	0.295
rs55871806	C	G	1.00E-200	0.530	0.015	0.028	0.020
rs62257659	A	G	1.61E-06	-0.202	0.042	-0.106	0.071
rs73176827	T	C	2.03E-06	-0.105	0.022	0.005	0.036
rs7512350	A	G	4.60E-06	0.092	0.020	-0.008	0.024
rs75366565	T	C	1.92E-175	-0.401	0.014	-0.012	0.020
rs7642750	A	C	2.94E-06	0.094	0.020	-0.026	0.025
rs78035061	A	C	2.87E-06	0.104	0.022	0.022	0.033
rs78199195	A	G	1.07E-06	-0.096	0.020	0.032	0.028
rs78357146	A	G	3.65E-19	-0.338	0.038	0.081	0.089
rs78714574	C	G	2.01E-23	0.473	0.047	0.004	0.051
rs79097546	T	C	8.75E-07	-0.275	0.056	0.082	0.121
rs80317525	A	G	2.49E-06	0.141	0.030	0.035	0.075
**IL-1α (F-statistic = 33.27, R2=4.15%)**							
rs10233998	T	G	4.64E-06	0.131	0.029	0.027	0.039
rs115494443	A	G	2.55E-06	0.266	0.057	-0.002	0.092
rs115602471	T	C	2.33E-06	0.222	0.047	0.008	0.140
rs117537822	T	C	7.17E-07	0.218	0.044	-0.059	0.045
rs11759846	A	G	1.36E-34	-0.326	0.027	-0.038	0.037
rs11878153	A	G	4.42E-07	0.112	0.022	0.005	0.023
rs12047801	T	C	4.67E-06	0.078	0.017	0.020	0.024
rs13195317	T	C	4.40E-09	-0.325	0.055	-0.439	0.438
rs16886503	A	T	3.28E-06	0.244	0.052	0.032	0.050
rs4767214	T	C	1.85E-06	0.062	0.013	0.011	0.016
rs523604	A	G	1.10E-07	0.075	0.014	-0.011	0.017
rs547199103	A	T	9.53E-08	-0.345	0.065	-0.180	0.155
rs61098036	A	G	4.70E-06	-0.086	0.019	-0.029	0.128
rs7138836	A	T	2.10E-06	-0.098	0.021	0.039	0.025
**IL-2 (F-statistic = 22.69, R2=2.98%)**							
rs10996419	T	C	2.38E-06	-0.122	0.026	-0.025	0.025

Supplementary Table 1. Details of systemic inflammatory proteins predicting SNPs with Hashimoto's Thyroiditis from GWAS (with SNPs reaching *P*< 5×10^{-6})

Inflammatory proteins	Hashimoto's Thyroiditis						
SNP	Effect allele	Other allele	P	Beta	Standard Error	log(OR)	Standard Error
rs142641125	T	C	5.73E-07	0.210	0.042	-0.104	0.330
rs144129701	C	G	2.79E-06	-0.270	0.058	0.061	0.060
rs1671226	C	G	5.91E-07	-0.085	0.017	-0.037	0.023
rs201229721	A	C	2.03E-06	-0.147	0.031	-0.087	0.039
rs28515912	A	C	1.22E-06	-0.072	0.015	-0.040	0.017
rs36006983	T	C	1.95E-06	-0.073	0.015	0.016	0.022
rs4703508	T	C	2.91E-06	-0.122	0.026	-0.029	0.047
rs6535212	T	G	3.36E-06	-0.065	0.014	0.020	0.018
rs74852566	T	G	3.52E-06	-0.200	0.043	-0.023	0.062
rs75739108	T	C	4.85E-06	0.174	0.038	-0.004	0.038
rs7638939	A	T	9.20E-07	0.085	0.017	0.021	0.022
rs77832761	A	G	3.44E-06	0.192	0.041	0.068	0.037
rs77853517	T	C	1.37E-06	-0.105	0.022	-0.006	0.022
rs79419497	A	G	1.99E-06	0.202	0.042	-0.119	0.063
**IL-20 (F-statistic =23.02, R2=2.73%)**							
rs111249483	A	G	8.10E-07	0.294	0.060	-0.015	0.115
rs114209791	A	G	3.50E-06	-0.213	0.046	-0.136	0.064
rs114363239	T	C	1.11E-06	-0.237	0.049	-0.058	0.084
rs117977288	T	G	9.96E-07	-0.155	0.032	0.057	0.040
rs12498781	A	G	3.57E-06	0.064	0.014	0.013	0.020
rs138771212	A	G	3.80E-06	-0.360	0.078	0.145	0.107
rs143181662	T	C	4.05E-06	0.072	0.016	-0.015	0.097
rs146918864	T	C	7.29E-07	-0.255	0.052	-0.087	0.044
rs188205021	T	C	2.00E-07	-0.222	0.043	-0.041	0.080
rs200680034	A	G	4.03E-07	-0.360	0.071	0.272	0.134
rs28542561	T	C	2.45E-06	-0.175	0.037	-0.021	0.044
rs72633862	T	G	4.65E-06	-0.172	0.038	-0.032	0.043
rs72659995	T	C	3.95E-06	-0.123	0.027	-0.030	0.033
**IL-20Rβ (F-statistic = 23.41, R2=2.45%)**							
rs113153423	A	G	2.60E-06	0.191	0.041	-0.012	0.062
rs113563938	A	T	2.62E-06	0.318	0.068	0.198	0.129

Supplementary Table 1. Details of systemic inflammatory proteins predicting SNPs with Hashimoto's Thyroiditis from GWAS (with SNPs reaching *P*< 5×10^{-6})

Inflammatory proteins	Hashimoto's Thyroiditis						
SNP	Effect allele	Other allele	P	Beta	Standard Error	log(OR)	Standard Error
rs11987533	A	G	3.34E-09	0.117	0.020	0.028	0.023
rs12138611	C	G	3.34E-06	0.119	0.026	-0.002	0.026
rs143067549	T	C	4.69E-06	-0.417	0.091	0.199	0.177
rs149111746	A	G	3.98E-06	0.236	0.051	0.000	0.060
rs540645152	A	C	1.36E-07	-0.082	0.016	-0.038	0.068
rs544388667	A	G	1.33E-06	0.363	0.075	0.029	0.053
rs59446445	A	G	4.61E-06	0.174	0.038	-0.093	0.059
rs6920753	A	G	4.01E-06	-0.066	0.014	-0.086	0.054
rs72785422	T	C	4.22E-06	0.090	0.020	0.280	0.137
**IL-22Rα1 (F-statistic = 22.53, R2=2.37%)**							
rs117263973	A	G	4.02E-06	0.347	0.075	-0.020	0.110
rs12240324	A	G	2.77E-06	0.220	0.047	0.007	0.037
rs13257574	T	C	3.36E-06	0.271	0.058	0.045	0.087
rs1473089	A	C	1.79E-06	0.268	0.056	0.048	0.068
rs17256492	T	C	3.56E-06	-0.075	0.016	-0.019	0.023
rs3181361	T	C	3.50E-06	0.270	0.058	-0.063	0.087
rs4811167	T	C	2.40E-06	0.063	0.013	0.027	0.017
rs56282088	A	G	8.23E-07	0.282	0.057	-0.044	0.152
rs7158900	A	G	1.04E-06	-0.067	0.014	0.049	0.053
rs75590335	A	G	3.25E-06	0.172	0.037	-0.064	0.041
rs78594373	T	C	3.23E-06	-0.127	0.027	-0.041	0.039
rs79229049	A	T	3.36E-07	-0.210	0.041	0.075	0.064
**IL-24 (F-statistic = 22.51, R2=2.61%)**							
rs11066309	A	G	1.74E-06	0.066	0.014	0.147	0.098
rs11256610	A	G	2.76E-06	0.301	0.064	0.059	0.186
rs116790447	T	C	3.16E-06	0.173	0.037	-0.079	0.046
rs117613902	A	G	4.97E-06	-0.300	0.066	0.056	0.136
rs143986178	A	G	4.02E-06	0.297	0.064	0.041	0.172
rs145295031	A	T	2.20E-06	0.306	0.065	-0.088	0.076
rs167018	A	G	1.36E-06	0.066	0.014	0.010	0.017
rs2341294	A	G	2.51E-06	-0.132	0.028	0.030	0.030

Supplementary Table 1. Details of systemic inflammatory proteins predicting SNPs with Hashimoto's Thyroiditis from GWAS (with SNPs reaching $P < 5\times10^{-6}$)

Inflammatory proteins	Hashimoto's Thyroiditis						
SNP	Effect allele	Other allele	P	Beta	Standard Error	log(OR)	Standard Error
rs36183478	T	C	2.15E-06	0.082	0.017	-0.056	0.103
rs60306270	A	G	3.51E-06	-0.089	0.019	0.008	0.028
rs73169406	A	G	8.22E-08	0.111	0.021	-0.031	0.028
rs7401175	A	T	4.78E-06	0.089	0.020	0.167	0.080
**IL-2 (F-statistic = 22.69, R2=2.98%)**							
rs115890206	A	G	3.81E-06	0.232	0.050	0.007	0.113
rs116007066	A	G	1.90E-06	-0.210	0.044	-0.065	0.056
rs11630708	T	C	2.09E-06	-0.210	0.044	-0.049	0.048
rs117538197	T	C	3.36E-06	0.121	0.026	0.024	0.039
rs13001788	T	C	6.65E-07	0.277	0.056	0.139	0.356
rs141460846	T	C	3.86E-06	-0.247	0.054	-0.054	0.064
rs17549820	T	C	1.00E-06	0.063	0.013	-0.015	0.017
rs2157876	T	G	2.13E-06	-0.113	0.024	0.003	0.026
rs34246740	T	C	3.80E-06	-0.072	0.016	-0.021	0.022
rs9321271	A	C	1.36E-06	-0.157	0.033	-0.054	0.073
rs9921181	A	G	2.20E-06	-0.061	0.013	0.031	0.017
**IL-33 (F-statistic = 22.22, R2=2.48%)**							
rs1013434	T	C	3.61E-06	-0.073	0.016	-0.004	0.020
rs10415966	A	C	3.74E-07	-0.118	0.023	0.008	0.032
rs112495884	A	G	4.47E-06	0.343	0.075	0.121	0.065
rs13336496	A	C	4.01E-06	0.094	0.020	-0.042	0.027
rs17149396	C	G	4.59E-06	0.183	0.040	0.040	0.068
rs35370544	T	G	1.73E-06	0.174	0.036	0.399	0.250
rs62556318	C	G	2.34E-06	0.131	0.028	0.006	0.024
rs66755903	A	G	3.45E-06	-0.075	0.016	0.030	0.019
rs7742625	C	G	3.03E-06	0.069	0.015	-0.015	0.018
rs77792454	T	C	4.82E-06	-0.117	0.026	-0.002	0.027
rs78455659	A	G	3.99E-06	0.126	0.027	0.039	0.026
rs8042883	T	C	4.55E-07	-0.068	0.014	-0.012	0.016
**IL-4 (F-statistic = 22.84, R2=2.30%)**							
rs116963481	A	G	2.16E-06	0.281	0.059	-0.032	0.057

Supplementary Table 1. Details of systemic inflammatory proteins predicting SNPs with Hashimoto's Thyroiditis from GWAS (with SNPs reaching $P < 5\times10^{-6}$)

Inflammatory proteins	Hashimoto's Thyroiditis						
SNP	Effect allele	Other allele	P	Beta	Standard Error	log(OR)	Standard Error
rs11989681	A	G	6.19E-07	0.068	0.014	0.002	0.017
rs12641440	C	G	2.31E-06	-0.118	0.025	-0.024	0.025
rs12900168	A	G	2.47E-06	0.072	0.015	0.071	0.063
rs13130198	A	G	1.16E-06	-0.077	0.016	-0.017	0.020
rs13418255	A	G	4.61E-06	0.070	0.015	0.022	0.017
rs1490743	T	G	1.49E-06	-0.134	0.028	0.049	0.036
rs2502347	T	C	4.01E-06	-0.077	0.017	-0.051	0.020
rs4876407	T	G	3.28E-06	-0.139	0.030	-0.035	0.037
rs7150107	T	C	3.78E-06	-0.113	0.024	0.007	0.115
rs75610895	T	C	1.68E-07	-0.235	0.045	-0.034	0.087
**IL-5 (F-statistic = 22.83, R2=2.25%)**							
rs10988407	A	G	3.28E-06	0.078	0.017	0.001	0.025
rs114829303	T	C	4.20E-06	0.166	0.036	0.006	0.044
rs12592185	T	C	1.27E-06	0.084	0.017	0.005	0.022
rs132210	T	C	1.14E-06	-0.069	0.014	-0.008	0.018
rs141941146	A	G	3.38E-06	0.383	0.083	-0.025	0.053
rs17009223	A	G	7.29E-07	-0.117	0.024	0.034	0.034
rs34639000	A	G	1.95E-07	0.077	0.015	-0.016	0.017
rs4277106	A	G	3.48E-06	-0.275	0.059	0.056	0.076
rs62069649	A	G	4.72E-06	0.221	0.048	-0.087	0.115
rs7142197	A	G	8.88E-07	-0.092	0.019	0.014	0.024
rs7193141	C	G	3.84E-06	0.241	0.052	0.064	0.052
**IL-6 (F-statistic = 45.04, R2=2.49%)**							
rs10234370	T	C	4.59E-06	0.058	0.013	-0.024	0.017
rs10977774	A	C	1.08E-06	-0.342	0.070	0.019	0.057
rs1346147	A	G	1.05E-06	-0.066	0.014	-0.015	0.019
rs17486819	T	G	3.82E-06	0.098	0.021	-0.007	0.024
rs185969953	T	G	5.04E-07	-0.343	0.068	0.109	0.137
rs187416244	T	C	1.71E-06	-0.224	0.047	-0.254	0.156
rs2228145	A	C	1.83E-45	-0.168	0.012	0.008	0.018
rs248536	T	G	2.70E-06	-0.067	0.014	-0.023	0.021

Supplementary Table 1. Details of systemic inflammatory proteins predicting SNPs with Hashimoto's Thyroiditis from GWAS (with SNPs reaching *P*< 5×10^{-6})

Inflammatory proteins	Hashimoto's Thyroiditis						
SNP	Effect allele	Other allele	P	Beta	Standard Error	log(OR)	Standard Error
**IL-7 (F-statistic = 25.26, R2=3.15%)**							
rs10403201	A	G	4.26E-06	-0.058	0.013	-0.004	0.019
rs1050152	T	C	9.84E-07	-0.065	0.013	-0.014	0.018
rs111066336	T	C	1.03E-07	0.075	0.014	0.272	0.102
rs112359206	A	G	5.67E-14	0.169	0.023	-0.146	0.165
rs11742240	T	G	7.86E-07	-0.065	0.013	-0.029	0.018
rs12321566	A	G	5.12E-07	0.070	0.014	0.040	0.018
rs12699881	A	G	8.33E-07	0.068	0.014	-0.012	0.018
rs144728120	T	C	2.23E-06	0.150	0.032	0.030	0.038
rs55849454	A	G	4.54E-06	0.073	0.016	-0.028	0.022
rs56889120	A	G	3.86E-06	0.097	0.021	0.057	0.067
rs570880779	T	C	3.73E-06	-0.234	0.051	0.039	0.134
rs576004227	A	T	3.67E-06	0.160	0.035	0.107	0.229
rs7026771	A	G	3.03E-06	0.069	0.015	-0.011	0.018
rs74131483	A	G	5.52E-07	0.137	0.027	0.035	0.038
rs77336315	A	C	2.24E-06	0.251	0.053	0.221	0.116
rs79072837	T	C	1.19E-06	-0.136	0.028	-0.004	0.045
**IL-8 (F-statistic =26.27, R2=3.17%)**							
rs12075	A	G	8.92E-09	0.067	0.012	-0.019	0.017
rs12188297	A	T	2.81E-07	-0.076	0.015	-0.002	0.020
rs13064825	T	C	3.06E-06	0.056	0.012	-0.006	0.017
rs141979722	T	C	3.27E-06	0.283	0.061	0.122	0.193
rs144815207	T	C	3.02E-07	0.319	0.062	-0.107	0.099
rs145125644	A	G	1.29E-06	0.269	0.056	0.021	0.040
rs1516478	T	C	4.37E-06	0.148	0.032	-0.042	0.070
rs34519991	T	C	3.75E-06	0.056	0.012	-0.019	0.017
rs6446951	A	G	3.87E-15	-0.189	0.024	0.003	0.036
rs6585420	T	C	2.00E-06	0.155	0.033	0.014	0.036
rs6709159	A	G	1.56E-06	0.056	0.012	-0.014	0.017
rs7216538	A	G	2.08E-06	-0.132	0.028	0.008	0.047
rs72633250	A	C	3.67E-06	0.057	0.012	0.001	0.017

Supplementary Table 1. Details of systemic inflammatory proteins predicting SNPs with Hashimoto's Thyroiditis from GWAS (with SNPs reaching $P < 5\times10^{-6}$)

Inflammatory proteins	Hashimoto's Thyroiditis						
SNP	Effect allele	Other allele	P	Beta	Standard Error	log(OR)	Standard Error
rs72886502	T	G	2.98E-07	-0.390	0.076	0.108	0.179
rs8069322	A	G	3.41E-07	0.067	0.013	0.018	0.019
rs906401	T	C	2.54E-06	0.094	0.020	0.022	0.031
rs972492	A	C	3.64E-07	-0.094	0.018	-0.018	0.026
**IFN-γ (F-statistic = 24.41, R2=2.41%)**							
rs140562194	T	C	1.97E-07	0.467	0.090	0.008	0.117
rs146527076	C	G	4.68E-07	0.194	0.038	-0.067	0.041
rs4810686	T	C	9.69E-07	0.067	0.014	-0.032	0.017
rs531747046	T	G	2.81E-06	0.268	0.057	-0.017	0.062
rs61863416	A	G	1.54E-06	0.136	0.028	0.023	0.046
rs62230781	A	G	5.98E-08	0.218	0.040	0.013	0.043
rs6442763	A	C	3.68E-06	0.065	0.014	-0.020	0.017
rs67101138	T	G	1.95E-06	0.146	0.031	-0.042	0.036
rs704	A	G	5.73E-07	-0.064	0.013	0.042	0.017
rs74903999	T	C	8.55E-07	0.349	0.071	0.057	0.078
**LAP TGF-β (F-statistic = 32.81, R2=4.55%)**							
rs10403201	A	G	1.79E-06	-0.060	0.013	-0.004	0.019
rs10508556	T	C	4.48E-06	0.052	0.011	-0.028	0.017
rs114152855	T	C	1.07E-06	-0.299	0.061	0.008	0.087
rs143420877	T	C	1.79E-06	0.145	0.030	-0.006	0.044
rs144834546	T	C	4.17E-07	-0.266	0.053	-0.306	0.121
rs149458216	A	C	1.09E-06	-0.230	0.047	0.021	0.070
rs1800472	A	G	2.55E-43	-0.462	0.034	-0.042	0.047
rs183319799	T	G	2.14E-06	0.219	0.046	0.058	0.058
rs1921885	A	C	1.51E-06	0.127	0.026	0.019	0.055
rs2281119	T	C	4.33E-06	-0.087	0.019	0.046	0.027
rs34536806	T	G	2.48E-06	0.099	0.021	-0.041	0.032
rs34992551	T	C	2.89E-06	-0.093	0.020	-0.015	0.021
rs60821320	A	C	3.04E-06	-0.108	0.023	-0.044	0.036
rs7013321	A	G	5.04E-07	-0.061	0.012	-0.017	0.017
rs7247630	T	C	3.68E-07	0.138	0.027	0.042	0.034

Supplementary Table 1. Details of systemic inflammatory proteins predicting SNPs with Hashimoto's Thyroiditis from GWAS (with SNPs reaching $P < 5\times10^{-6}$)

Inflammatory proteins	Hashimoto's Thyroiditis						
SNP	Effect allele	Other allele	P	Beta	Standard Error	log(OR)	Standard Error
rs73045256	A	G	3.40E-12	-0.192	0.028	-0.049	0.030
rs73112514	C	G	2.12E-06	0.205	0.043	0.381	0.298
rs7570818	A	G	3.50E-06	-0.085	0.018	-0.034	0.031
rs7656674	A	T	2.30E-07	0.063	0.012	0.016	0.017
rs7820135	T	C	1.44E-06	-0.075	0.016	0.019	0.020
**LIF (F-statistic = 22.32, R2=2.53%)**							
rs11127419	T	C	3.82E-06	0.071	0.015	-0.016	0.018
rs11608349	A	T	3.08E-06	-0.308	0.066	-0.062	0.122
rs116794364	C	G	1.77E-06	-0.302	0.063	-0.051	0.074
rs11883375	A	G	2.87E-07	-0.101	0.020	0.032	0.026
rs13264315	A	T	1.66E-06	0.076	0.016	-0.034	0.103
rs145344087	C	G	4.59E-06	0.255	0.056	0.108	0.076
rs234592	A	G	3.02E-06	-0.064	0.014	0.005	0.017
rs332426	A	G	2.53E-06	0.112	0.024	-0.034	0.031
rs7225483	T	G	2.70E-06	0.067	0.014	-0.018	0.016
rs74992559	A	G	2.60E-06	0.173	0.037	0.034	0.062
rs80092539	A	T	3.13E-06	0.099	0.021	-0.007	0.030
rs825264	A	G	2.90E-06	-0.212	0.045	-0.038	0.034
**LIFR (F-statistic = 42.86, R2=6.71%)**							
rs115938660	T	C	3.01E-06	-0.218	0.047	-0.041	0.050
rs117117000	A	G	1.91E-06	0.159	0.033	0.072	0.036
rs12754806	A	G	3.94E-06	0.056	0.012	0.010	0.016
rs141933232	A	G	6.07E-07	-0.272	0.055	-0.210	0.405
rs147006060	A	G	4.28E-06	-0.260	0.057	-0.092	0.054
rs1690678	A	C	2.50E-06	0.090	0.019	-0.016	0.023
rs2289779	T	G	4.09E-09	0.079	0.013	0.014	0.016
rs3963364	A	C	2.80E-06	-0.095	0.020	0.289	0.137
rs41307428	T	C	1.21E-11	0.203	0.030	0.017	0.026
rs4937122	T	G	3.61E-07	0.126	0.025	0.030	0.026
rs6002001	A	G	4.66E-06	0.649	0.142	0.122	1.077
rs62258077	T	C	1.26E-06	-0.063	0.013	0.111	0.099

Supplementary Table 1. Details of systemic inflammatory proteins predicting SNPs with Hashimoto's Thyroiditis from GWAS (with SNPs reaching $P < 5\times10^{-6}$)

Inflammatory proteins	Hashimoto's Thyroiditis						
SNP	Effect allele	Other allele	P	Beta	Standard Error	log(OR)	Standard Error
rs635634	T	C	2.61E-79	-0.289	0.015	0.003	0.020
rs72730728	A	T	2.84E-06	0.141	0.030	0.033	0.037
rs72837682	T	C	3.30E-06	-0.080	0.017	-0.188	0.120
rs73157388	T	C	4.60E-06	-0.131	0.029	0.012	0.027
rs778016	A	G	2.65E-06	-0.068	0.015	-0.012	0.018
rs9313148	C	G	1.91E-06	-0.082	0.017	0.021	0.025
**M-CSF (F-statistic = 33.23, R2=4.06%)**							
rs10042665	C	G	1.76E-06	0.058	0.012	-0.028	0.018
rs115370595	A	G	4.42E-06	-0.228	0.050	-0.006	0.088
rs116443177	T	C	1.75E-06	0.083	0.017	-0.030	0.021
rs11685487	T	C	1.46E-06	0.094	0.020	-0.017	0.025
rs117591817	C	G	4.82E-06	-0.199	0.044	-0.074	0.052
rs142421172	A	G	3.45E-06	0.144	0.031	-0.023	0.035
rs17610659	T	C	3.31E-46	0.168	0.012	0.033	0.017
rs17734417	T	C	3.79E-06	-0.123	0.027	-0.059	0.041
rs2523992	T	G	8.05E-07	-0.075	0.015	-0.088	0.075
rs563284470	A	G	5.93E-08	0.209	0.039	-0.389	0.266
rs7227917	A	G	2.43E-06	-0.087	0.019	0.009	0.024
rs72783071	T	C	1.53E-06	-0.199	0.041	0.055	0.070
rs74849049	T	G	4.88E-06	-0.091	0.020	-0.042	0.027
rs7538029	A	C	2.64E-06	0.078	0.017	-0.021	0.018
rs75869070	A	G	1.47E-06	0.115	0.024	0.038	0.046
rs76421506	A	G	3.64E-06	0.078	0.017	0.001	0.024
rs78652121	C	G	4.81E-06	-0.119	0.026	0.105	0.125
**MCP-1 (F-statistic = 42.74, R2=6.86%)**							
rs117265389	C	G	4.24E-06	0.173	0.038	-0.020	0.051
rs117317477	T	G	4.57E-06	-0.323	0.070	0.047	0.039
rs117510656	T	C	4.16E-06	-0.259	0.056	0.020	0.057
rs11870164	T	C	7.61E-09	0.075	0.013	0.016	0.018
rs12075	A	G	8.39E-51	0.169	0.011	-0.019	0.017
rs12133241	T	G	3.54E-06	0.073	0.016	-0.024	0.023

Supplementary Table 1. Details of systemic inflammatory proteins predicting SNPs with Hashimoto's Thyroiditis from GWAS (with SNPs reaching $P < 5\times10^{-6}$)

Inflammatory proteins	Hashimoto's Thyroiditis						
SNP	Effect allele	Other allele	P	Beta	Standard Error	log(OR)	Standard Error
rs12367122	A	G	3.35E-06	0.074	0.016	-0.012	0.022
rs146186562	T	G	3.96E-06	-0.229	0.050	-0.043	0.049
rs148307902	A	G	2.08E-06	0.255	0.054	-0.180	0.085
rs149604038	C	G	4.21E-07	-0.281	0.056	0.038	0.083
rs181998213	T	C	8.47E-08	0.372	0.070	-0.054	0.042
rs182599222	A	T	3.13E-06	-0.403	0.086	-0.530	0.717
rs1846616	A	T	6.33E-13	0.086	0.012	-0.020	0.018
rs190108694	A	G	4.64E-06	-0.208	0.046	-0.024	0.064
rs207311	T	C	4.32E-06	0.058	0.013	0.007	0.017
rs34292320	T	C	3.32E-07	-0.068	0.013	-0.021	0.019
rs35728689	A	G	5.44E-52	0.300	0.020	0.018	0.029
rs4682862	A	G	2.34E-06	0.058	0.012	-0.019	0.017
rs7619350	A	G	9.32E-07	0.099	0.020	0.002	0.023
rs77128226	T	C	1.08E-06	-0.106	0.022	0.072	0.037
rs77344300	T	C	2.44E-06	-0.138	0.029	0.016	0.039
rs79697698	T	C	9.86E-08	-0.348	0.065	-0.036	0.040
rs9942602	T	C	1.28E-07	0.062	0.012	-0.012	0.017
**MCP-2 (F-statistic = 117.46, R2=23.48%)**							
rs112168535	T	C	8.90E-07	-0.155	0.032	-0.004	0.045
rs11652256	A	G	3.48E-144	-0.905	0.035	-0.023	0.048
rs12075	A	G	4.95E-17	-0.097	0.012	-0.019	0.017
rs12523126	A	T	1.35E-06	0.112	0.023	-0.065	0.035
rs138884871	T	C	3.37E-06	0.220	0.047	0.066	0.068
rs144101664	T	C	1.45E-11	-0.369	0.055	-0.055	0.071
rs144618566	T	C	7.15E-22	0.517	0.054	0.316	0.137
rs145516279	A	G	3.56E-06	0.223	0.048	-0.115	0.063
rs16969443	T	C	5.38E-11	-0.497	0.076	0.058	0.037
rs181726805	A	G	3.75E-06	-0.248	0.054	-0.026	0.053
rs1821142	T	C	4.64E-17	0.401	0.048	-0.190	0.084
rs188234686	C	G	1.73E-12	-0.359	0.051	0.059	0.061
rs34202026	A	T	6.89E-72	0.665	0.037	0.018	0.049

Supplementary Table 1. Details of systemic inflammatory proteins predicting SNPs with Hashimoto's Thyroiditis from GWAS (with SNPs reaching *P*< 5×10^-6^)

Inflammatory proteins	Hashimoto's Thyroiditis						
SNP	Effect allele	Other allele	P	Beta	Standard Error	log(OR)	Standard Error
rs35904471	T	C	3.99E-06	0.076	0.017	0.023	0.018
rs407595	A	C	2.12E-06	0.070	0.015	-0.005	0.019
rs4722985	T	C	2.52E-06	-0.063	0.013	0.031	0.020
rs6685547	A	G	4.22E-07	0.146	0.029	-0.055	0.044
rs72825048	A	G	1.00E-200	-0.812	0.027	-0.086	0.037
rs74350159	A	G	4.19E-06	0.280	0.061	-0.037	0.091
rs7538845	C	G	3.50E-06	0.057	0.012	0.004	0.017
rs75573205	C	G	2.99E-06	0.191	0.041	0.068	0.044
rs75580960	T	C	1.52E-06	-0.090	0.019	-0.036	0.024
rs76263310	A	G	7.74E-11	0.296	0.046	-0.026	0.060
rs78743764	A	C	3.53E-06	-0.223	0.048	-0.125	0.067
rs79567569	A	C	9.16E-07	-0.328	0.067	-0.286	0.154
rs79965992	A	G	2.78E-06	0.109	0.023	0.083	0.177
rs80131163	A	G	8.77E-07	0.196	0.040	0.001	0.036
rs80219280	T	G	2.00E-173	-0.904	0.032	0.035	0.060
rs948962	A	C	4.84E-06	0.054	0.012	0.030	0.017
rs9889517	A	G	4.83E-07	0.061	0.012	0.011	0.017
**MCP-3 (F-statistic = 33.63, R2=5.71%)**							
rs11276535	T	C	3.70E-07	-0.085	0.017	0.011	0.099
rs12075	A	G	4.67E-12	-0.090	0.013	-0.019	0.017
rs12142504	T	C	2.78E-06	0.078	0.017	-0.005	0.056
rs139796674	A	G	2.10E-06	0.233	0.049	-0.036	0.055
rs146174820	A	G	1.13E-06	0.266	0.055	-0.043	0.096
rs147793446	A	G	2.60E-06	0.194	0.041	-0.018	0.062
rs148859911	A	G	4.34E-06	0.151	0.033	0.001	0.039
rs16947053	A	G	3.34E-06	0.226	0.049	-0.144	0.063
rs182239847	T	G	1.75E-06	-0.172	0.036	-0.027	0.029
rs2228467	T	C	2.88E-36	-0.317	0.025	-0.014	0.032
rs28667091	T	G	4.05E-06	-0.061	0.013	-0.010	0.017
rs4146770	T	C	1.56E-08	-0.093	0.017	0.009	0.021
rs481267	A	G	4.99E-07	-0.075	0.015	-0.028	0.017

Supplementary Table 1. Details of systemic inflammatory proteins predicting SNPs with Hashimoto's Thyroiditis from GWAS (with SNPs reaching *P*< $5×10^{-6}$)

Inflammatory proteins	Hashimoto's Thyroiditis						
SNP	Effect allele	Other allele	P	Beta	Standard Error	log(OR)	Standard Error
rs7116054	T	C	2.47E-08	-0.100	0.018	-0.020	0.027
rs7213460	A	G	9.58E-13	0.094	0.013	-0.010	0.017
rs7327202	C	G	5.89E-07	-0.097	0.019	-0.034	0.021
rs74157668	T	C	4.46E-06	0.157	0.034	0.024	0.054
rs78848134	T	C	2.04E-06	0.244	0.051	-0.006	0.073
rs9968876	T	G	2.21E-06	-0.105	0.022	0.032	0.032
**MCP-4 (F-statistic = 65.03, R2=8.50%)**							
rs10140592	A	G	1.01E-06	0.270	0.055	0.017	0.047
rs115511138	A	G	3.21E-06	0.196	0.042	-0.074	0.090
rs12075	A	G	9.89E-62	0.189	0.011	-0.019	0.017
rs144118870	A	G	2.01E-06	0.175	0.037	0.122	0.045
rs181250	T	C	6.99E-07	-0.103	0.021	-0.081	0.087
rs2497351	T	C	4.09E-06	0.067	0.015	0.044	0.021
rs2631360	A	G	3.34E-06	0.061	0.013	0.014	0.018
rs2721961	T	G	1.72E-19	-0.120	0.013	0.008	0.018
rs3136676	A	G	1.60E-54	0.390	0.025	-0.009	0.035
rs3917900	A	G	1.70E-38	0.156	0.012	-0.009	0.018
rs55842014	A	G	1.11E-06	-0.064	0.013	-0.001	0.018
rs62201455	A	C	2.18E-06	-0.214	0.045	0.085	0.040
rs62336911	T	C	2.29E-06	0.157	0.033	0.004	0.048
rs72825017	T	G	1.77E-15	-0.200	0.025	-0.073	0.035
rs74794857	T	C	1.24E-06	0.067	0.014	-0.019	0.017
rs7612912	T	C	1.33E-26	0.128	0.012	-0.010	0.017
rs8112014	A	G	4.49E-06	-0.058	0.013	-0.002	0.019
rs847767	A	G	4.58E-06	0.077	0.017	0.039	0.021
rs926646	T	C	2.47E-06	0.143	0.030	-0.031	0.044
**MIP-1α (F-statistic = 95.62, R2=9.05%)**							
rs1125560	T	C	2.06E-07	-0.064	0.012	-0.001	0.056
rs113448118	A	G	4.88E-06	0.082	0.018	0.014	0.025
rs1143687	T	C	3.04E-07	-0.123	0.024	0.031	0.038
rs1719209	A	G	1.00E-200	0.409	0.014	0.015	0.022

Supplementary Table 1. Details of systemic inflammatory proteins predicting SNPs with Hashimoto's Thyroiditis from GWAS (with SNPs reaching *P*< 5×10^{-6})

Inflammatory proteins	Hashimoto's Thyroiditis						
SNP	Effect allele	Other allele	P	Beta	Standard Error	log(OR)	Standard Error
rs2847249	T	C	4.97E-06	-0.062	0.014	0.016	0.018
rs2853926	A	G	4.84E-06	-0.061	0.013	-0.053	0.023
rs56675286	A	C	2.75E-08	-0.181	0.033	-0.048	0.052
rs5743604	A	G	4.98E-08	-0.074	0.014	-0.022	0.024
rs677925	T	C	2.99E-06	-0.067	0.014	0.057	0.066
rs76522981	T	C	7.53E-09	-0.146	0.025	-0.056	0.034
rs78871521	T	C	1.04E-06	0.181	0.037	0.026	0.050
rs9507462	A	G	2.52E-06	-0.063	0.013	-0.068	0.099
rs9540456	A	G	3.12E-07	0.093	0.018	0.064	0.030
rs9911791	A	G	1.71E-27	0.176	0.016	0.020	0.020
**MMP-1 (F-statistic = 40.56, R2=4.22%)**							
rs10978641	A	T	3.56E-06	0.065	0.014	-0.013	0.020
rs10990535	T	C	1.91E-07	0.070	0.014	-0.004	0.018
rs11673125	T	C	4.10E-06	-0.059	0.013	-0.003	0.019
rs116889819	A	G	4.45E-09	-0.176	0.030	0.004	0.051
rs12141791	A	G	3.90E-11	0.086	0.013	0.018	0.017
rs12367368	T	G	3.67E-06	0.086	0.019	-0.023	0.023
rs141782294	A	G	2.56E-17	0.377	0.045	0.006	0.048
rs188626385	T	C	2.20E-06	-0.251	0.053	0.348	0.202
rs2155053	T	C	4.68E-34	-0.321	0.026	0.063	0.039
rs2229629	A	G	2.80E-06	-0.315	0.067	-0.112	0.062
rs4734879	A	G	1.11E-16	0.114	0.014	0.016	0.019
rs6717478	A	T	4.65E-06	0.155	0.034	0.004	0.043
rs7219205	A	G	2.57E-06	0.057	0.012	0.012	0.017
rs77095316	T	G	2.69E-09	-0.142	0.024	-0.024	0.038
rs873675	T	C	1.34E-07	0.123	0.023	-0.032	0.030
**MMP-10 (F-statistic = 30.92, R2=3.21%)**							
rs112062276	T	G	1.36E-06	0.120	0.025	-0.007	0.030
rs11225354	T	C	5.38E-60	0.296	0.018	-0.002	0.027
rs112327130	C	G	2.51E-06	-0.286	0.061	-0.119	0.135
rs113958571	A	G	4.18E-06	-0.232	0.050	0.035	0.121

Supplementary Table 1. Details of systemic inflammatory proteins predicting SNPs with Hashimoto's Thyroiditis from GWAS (with SNPs reaching *P*< 5×10⁻⁶)

Inflammatory proteins	Hashimoto's Thyroiditis						
SNP	Effect allele	Other allele	P	Beta	Standard Error	log(OR)	Standard Error
rs12567824	A	C	4.76E-06	0.070	0.015	-0.004	0.022
rs1356799	T	G	4.22E-06	-0.069	0.015	0.026	0.020
rs142915220	A	G	4.37E-11	-0.326	0.049	0.126	0.099
rs17099622	A	G	8.12E-19	-0.206	0.023	-0.009	0.032
rs17860955	T	C	2.60E-129	1.137	0.047	-0.016	0.124
rs2584662	A	C	9.91E-07	-0.060	0.012	0.043	0.017
rs3742926	T	C	3.27E-06	-0.091	0.020	0.030	0.024
rs539147801	A	C	3.53E-06	-0.206	0.044	-0.115	0.198
rs55804377	A	G	3.18E-06	-0.219	0.047	0.087	0.041
rs72699188	C	G	2.04E-08	-0.093	0.017	0.026	0.024
rs7966257	C	G	4.20E-06	-0.082	0.018	0.006	0.026
**NKR2B4 (F-statistic = 40.28, R2=5.60%)**							
rs10903115	A	G	4.61E-06	-0.056	0.012	-0.031	0.017
rs10992054	T	C	1.20E-06	-0.371	0.076	0.024	0.110
rs11020961	A	G	1.84E-06	0.077	0.016	-0.006	0.020
rs11220506	A	G	1.79E-06	-0.070	0.015	-0.012	0.019
rs11265488	A	G	1.24E-07	-0.062	0.012	-0.009	0.017
rs11265493	A	G	3.99E-71	0.212	0.012	-0.013	0.017
rs114758826	A	G	8.65E-08	-0.180	0.034	0.006	0.047
rs139924614	A	G	3.33E-06	0.162	0.035	0.023	0.089
rs1434219	T	C	1.68E-06	-0.059	0.012	-0.005	0.017
rs147033439	A	G	5.88E-07	0.205	0.041	-0.022	0.072
rs149458216	A	C	4.97E-06	-0.215	0.047	0.021	0.070
rs1574847	C	G	4.59E-06	-0.064	0.014	-0.025	0.019
rs2554697	A	G	2.88E-06	0.063	0.013	0.009	0.018
rs34826779	T	G	4.99E-06	0.063	0.014	-0.015	0.020
rs3828139	T	C	4.05E-10	0.074	0.012	0.038	0.016
rs4672486	A	G	1.61E-08	0.066	0.012	0.024	0.017
rs56272634	T	C	2.31E-08	0.070	0.013	-0.003	0.018
rs6928352	A	G	5.87E-07	-0.107	0.022	-0.002	0.031
rs72837690	T	C	5.37E-10	-0.078	0.013	0.016	0.017

Supplementary Table 1. Details of systemic inflammatory proteins predicting SNPs with Hashimoto's Thyroiditis from GWAS (with SNPs reaching *P*< 5×10^{-6})

Inflammatory proteins	Hashimoto's Thyroiditis						
SNP	Effect allele	Other allele	P	Beta	Standard Error	log(OR)	Standard Error
rs78142752	T	C	4.15E-06	0.229	0.050	0.073	0.055
**NT-3 (F-statistic = 23.94, R2=3.06%)**							
rs10780690	T	G	3.46E-06	0.056	0.012	-0.004	0.017
rs11225042	A	C	1.64E-06	-0.097	0.020	-0.044	0.023
rs112418830	T	C	2.06E-07	0.137	0.026	-0.014	0.033
rs12517801	A	G	4.50E-06	-0.056	0.012	-0.018	0.017
rs145686993	C	G	4.84E-06	-0.246	0.054	0.052	0.076
rs146532158	T	C	1.46E-06	-0.186	0.039	0.014	0.053
rs17537796	A	C	3.33E-06	-0.257	0.055	0.026	0.065
rs2034246	A	C	1.23E-06	0.058	0.012	0.033	0.017
rs2107473	A	G	2.36E-06	-0.239	0.051	0.030	0.073
rs2846299	A	G	3.22E-06	0.058	0.013	0.001	0.018
rs28735437	C	G	1.06E-12	-0.117	0.016	0.045	0.027
rs3218587	A	G	3.49E-06	0.136	0.029	0.025	0.056
rs376150779	C	G	1.92E-06	-0.082	0.017	0.120	0.123
rs5751454	T	C	4.64E-06	0.182	0.040	-0.063	0.042
rs58399805	A	T	4.98E-07	0.111	0.022	0.006	0.028
rs72652073	C	G	3.48E-06	0.058	0.013	0.008	0.017
rs73166403	T	C	4.22E-06	-0.092	0.020	0.032	0.026
rs79759094	A	G	3.60E-06	-0.116	0.025	0.025	0.034
**NTN (F-statistic = 22.59, R2=3.19%)**							
rs10412462	A	C	2.25E-06	-0.173	0.037	0.017	0.037
rs113580784	A	G	3.80E-06	-0.229	0.050	-0.045	0.040
rs143131035	T	C	4.91E-07	-0.436	0.087	0.064	0.068
rs182274799	A	G	4.46E-07	0.157	0.031	0.061	0.043
rs192427410	T	G	1.92E-06	0.374	0.079	-0.085	0.050
rs29630	T	C	3.25E-06	-0.074	0.016	0.013	0.020
rs4360502	A	G	3.76E-06	-0.065	0.014	-0.026	0.018
rs62120726	A	G	4.88E-06	0.079	0.017	0.022	0.024
rs7202385	A	T	2.02E-06	0.075	0.016	-0.056	0.104
rs76777800	T	C	4.44E-06	0.339	0.074	-0.115	0.189

Supplementary Table 1. Details of systemic inflammatory proteins predicting SNPs with Hashimoto's Thyroiditis from GWAS (with SNPs reaching $P < 5\times10^{-6}$)

Inflammatory proteins	Hashimoto's Thyroiditis						
SNP	Effect allele	Other allele	P	Beta	Standard Error	log(OR)	Standard Error
rs7712604	C	G	5.90E-07	-0.088	0.018	0.017	0.019
rs77216559	T	C	9.44E-07	0.167	0.034	-0.021	0.044
rs8033310	T	C	3.20E-06	0.133	0.029	-0.022	0.033
rs908372	C	G	4.80E-06	-0.062	0.014	0.001	0.017
**OPG (F-statistic = 40.75, R2=6.03%)**							
rs10142466	A	G	1.70E-07	0.057	0.011	0.000	0.016
rs112635299	T	G	1.48E-09	0.262	0.043	0.017	0.059
rs114165349	C	G	1.01E-08	-0.195	0.034	0.014	0.045
rs1230164	C	G	1.98E-07	-0.067	0.013	0.012	0.020
rs138408096	A	G	2.09E-06	-0.201	0.042	-0.139	0.076
rs138557324	T	C	1.91E-06	-0.225	0.047	-0.100	0.127
rs1437633	A	C	7.69E-08	-0.079	0.015	0.004	0.023
rs144471361	T	C	1.04E-06	0.307	0.063	0.078	0.474
rs17600346	T	C	1.54E-07	-0.157	0.030	0.004	0.040
rs192337992	A	G	3.23E-06	-0.156	0.033	0.209	0.249
rs2247769	T	C	1.42E-50	-0.171	0.011	0.022	0.017
rs271665	T	C	1.90E-07	-0.067	0.013	0.022	0.018
rs28454196	C	G	3.97E-06	-0.224	0.049	-0.048	0.050
rs376613127	A	G	2.14E-06	-0.269	0.057	0.091	0.079
rs56009454	T	G	4.59E-06	-0.064	0.014	0.036	0.053
rs61968587	A	G	2.47E-06	-0.267	0.057	0.010	0.109
rs6985207	A	C	4.67E-06	0.052	0.011	-0.019	0.017
rs704	A	G	1.14E-36	-0.140	0.011	0.042	0.017
rs74111611	T	C	7.96E-07	-0.147	0.030	0.049	0.057
rs77507094	A	G	4.64E-06	0.197	0.043	-0.003	0.046
rs9946923	T	C	4.77E-06	0.172	0.038	-0.025	0.081
**OSM (F-statistic = 32.5, R2=3.13%)**							
rs111865607	T	C	1.44E-09	-0.108	0.018	0.127	0.125
rs116928468	A	G	1.69E-06	-0.257	0.054	-0.052	0.036
rs2285179	A	G	5.50E-08	-0.066	0.012	-0.029	0.017
rs2511241	T	C	6.38E-14	0.177	0.024	0.016	0.030

Supplementary Table 1. Details of systemic inflammatory proteins predicting SNPs with Hashimoto's Thyroiditis from GWAS (with SNPs reaching $P < 5\times10^{-6}$)

Inflammatory proteins	Hashimoto's Thyroiditis						
SNP	Effect allele	Other allele	P	Beta	Standard Error	log(OR)	Standard Error
rs28444486	A	G	2.92E-06	0.064	0.014	-0.009	0.019
rs3859189	A	G	4.95E-21	0.110	0.012	0.004	0.017
rs559651	T	C	2.01E-06	-0.059	0.013	0.008	0.017
rs61897429	A	G	6.28E-07	-0.085	0.017	0.002	0.025
rs7007281	A	T	2.00E-06	0.116	0.024	0.033	0.031
rs7102957	T	C	3.46E-06	-0.080	0.017	-0.020	0.021
rs7432752	A	G	1.28E-07	0.088	0.017	0.046	0.073
rs76275710	A	G	3.85E-07	0.267	0.053	-0.042	0.049
rs80303768	A	G	3.35E-06	-0.152	0.033	-0.059	0.077
**PD-L1 (F-statistic = 29.16, R2=3.90%)**							
rs10043030	T	C	1.99E-06	-0.118	0.025	-0.151	0.068
rs113674769	T	G	4.92E-06	-0.114	0.025	0.061	0.187
rs12539643	C	G	2.60E-06	0.191	0.041	-0.037	0.035
rs146328951	A	G	7.02E-07	-0.493	0.099	-0.143	0.203
rs184265227	A	G	5.58E-07	0.096	0.019	-0.162	0.078
rs2283438	T	C	1.52E-06	-0.058	0.012	0.005	0.017
rs35024691	C	G	4.17E-06	-0.101	0.022	0.032	0.028
rs4974444	A	G	3.55E-06	-0.082	0.018	-0.009	0.023
rs60830384	A	T	9.16E-08	0.114	0.021	0.041	0.031
rs62373966	T	G	2.55E-07	0.141	0.027	0.025	0.027
rs6917605	T	C	1.79E-06	0.055	0.012	-0.012	0.016
rs7026097	A	T	1.99E-07	0.083	0.016	-0.275	0.129
rs7125631	T	C	1.96E-06	-0.061	0.013	0.009	0.018
rs7304709	T	C	4.44E-06	0.054	0.012	-0.008	0.017
rs75510105	A	G	5.15E-07	0.193	0.039	0.030	0.075
rs79045618	A	G	5.83E-07	0.162	0.032	-0.036	0.032
rs7945680	T	G	8.75E-08	0.061	0.011	-0.023	0.017
rs822335	T	C	3.77E-27	-0.130	0.012	0.022	0.017
**S100A12 (F-statistic = 32.92, R2=3.95%)**							
rs112956934	T	C	3.67E-06	0.188	0.041	-0.046	0.049
rs112988802	T	C	1.89E-06	-0.140	0.029	0.024	0.053

Supplementary Table 1. Details of systemic inflammatory proteins predicting SNPs with Hashimoto's Thyroiditis from GWAS (with SNPs reaching $P < 5\times10^{-6}$)

Inflammatory proteins	Hashimoto's Thyroiditis						
SNP	Effect allele	Other allele	P	Beta	Standard Error	log(OR)	Standard Error
rs13384799	T	C	2.92E-06	-0.062	0.013	-0.010	0.018
rs138439507	C	G	2.28E-06	0.087	0.018	-0.074	0.081
rs147302029	A	G	1.65E-07	0.181	0.035	-0.017	0.047
rs150015576	A	C	4.51E-06	0.194	0.042	-0.016	0.044
rs174344	A	G	7.97E-07	0.062	0.013	0.013	0.018
rs191808657	A	C	4.22E-06	0.306	0.067	-0.010	0.071
rs2056031	T	C	3.86E-07	-0.060	0.012	-0.029	0.017
rs2425640	A	G	2.14E-06	-0.058	0.012	-0.011	0.017
rs3014874	A	G	2.15E-36	-0.173	0.014	-0.007	0.019
rs3802266	A	G	2.56E-06	0.056	0.012	0.011	0.017
rs55783131	T	C	1.19E-06	0.206	0.043	-0.005	0.051
rs56268787	T	C	4.01E-06	-0.094	0.020	0.027	0.031
rs59538998	C	G	4.13E-06	-0.119	0.026	-0.069	0.032
rs61803119	T	G	1.02E-14	0.150	0.019	0.005	0.029
rs78800546	A	G	2.18E-06	-0.253	0.053	-0.012	0.098
**SCF (F-statistic = 43.67, R2=9.73%)**							
rs10832027	A	G	2.21E-06	-0.057	0.012	0.005	0.017
rs10894669	A	C	2.63E-06	-0.061	0.013	0.022	0.018
rs11126305	A	G	1.02E-06	-0.057	0.012	0.003	0.017
rs11199541	T	C	4.96E-06	0.155	0.034	0.008	0.040
rs11608458	T	C	6.83E-09	-0.071	0.012	0.000	0.018
rs11877350	A	G	4.26E-06	0.054	0.012	0.015	0.017
rs12149545	A	G	1.20E-20	0.115	0.012	0.017	0.018
rs12378537	T	C	1.63E-06	0.105	0.022	-0.020	0.023
rs138854302	T	C	1.16E-11	-0.139	0.021	0.014	0.031
rs139690	A	G	4.22E-06	0.110	0.024	0.044	0.045
rs146276488	A	G	2.68E-06	0.242	0.052	-0.035	0.073
rs151246947	A	G	3.00E-06	0.212	0.045	-0.018	0.041
rs16942887	A	G	3.14E-22	0.166	0.017	0.006	0.024
rs174535	T	C	1.24E-09	-0.074	0.012	0.006	0.017
rs186248365	A	G	2.37E-06	-0.428	0.091	0.045	0.045

Supplementary Table 1. Details of systemic inflammatory proteins predicting SNPs with Hashimoto's Thyroiditis from GWAS (with SNPs reaching $P< 5\times10^{-6}$)

Inflammatory proteins	Hashimoto's Thyroiditis						
SNP	Effect allele	Other allele	P	Beta	Standard Error	log(OR)	Standard Error
rs2024570	T	C	5.85E-09	-0.199	0.034	0.034	0.054
rs2133027	A	G	4.75E-06	0.472	0.103	0.051	0.049
rs2161465	A	T	2.55E-06	-0.072	0.015	0.183	0.115
rs2740488	A	C	3.57E-18	0.116	0.013	-0.003	0.020
rs2980888	T	C	5.94E-08	-0.068	0.013	0.008	0.019
rs34813411	T	C	1.79E-06	-0.162	0.034	-0.033	0.092
rs45617141	T	G	4.38E-08	0.280	0.051	0.218	0.137
rs557933	A	C	2.78E-06	-0.055	0.012	0.004	0.017
rs6073958	T	C	1.16E-87	-0.286	0.014	0.015	0.022
rs705379	A	G	4.87E-11	0.079	0.012	-0.006	0.017
rs71431776	A	C	1.03E-06	-0.094	0.019	-0.002	0.031
rs71644719	A	G	4.40E-06	-0.158	0.035	-0.052	0.040
rs72847212	T	C	1.53E-06	-0.072	0.015	0.007	0.022
rs7664024	T	C	2.69E-06	-0.072	0.015	-0.007	0.025
rs78397068	T	C	2.49E-07	0.120	0.023	-0.037	0.038
rs785900	T	C	1.10E-06	0.058	0.012	0.020	0.017
rs798893	C	G	1.61E-11	0.095	0.014	-0.019	0.018
**SIRT2 (F-statistic = 30.44, R2=2.80%)**							
rs1034059	A	G	1.59E-07	0.076	0.015	0.038	0.019
rs11115639	A	C	4.86E-06	-0.084	0.018	0.024	0.023
rs115455827	A	G	4.02E-06	0.128	0.028	-0.001	0.032
rs116198125	T	C	3.78E-06	-0.152	0.033	-0.040	0.051
rs12152184	A	G	3.49E-06	0.097	0.021	-0.043	0.026
rs12531843	C	G	1.21E-07	-0.129	0.024	0.056	0.030
rs138897138	A	G	1.84E-06	0.104	0.022	0.032	0.031
rs144373891	T	C	7.69E-29	-0.574	0.052	0.044	0.091
rs147383134	T	C	3.02E-06	0.257	0.055	0.059	0.105
rs60459634	A	G	3.45E-06	-0.106	0.023	0.010	0.030
rs72883370	A	G	3.30E-06	-0.343	0.074	0.026	0.069
rs7718478	A	T	3.65E-06	0.064	0.014	0.017	0.020
rs77381020	T	C	2.80E-06	0.086	0.018	-0.013	0.023

Supplementary Table 1. Details of systemic inflammatory proteins predicting SNPs with Hashimoto's Thyroiditis from GWAS (with SNPs reaching $P< 5\times10^{-6}$)

Inflammatory proteins	Hashimoto's Thyroiditis						
SNP	Effect allele	Other allele	P	Beta	Standard Error	log(OR)	Standard Error
**SLAM (F-statistic = 33.87, R2=6.65%)**							
rs10212190	A	T	1.88E-09	0.073	0.012	-0.024	0.017
rs10499604	T	C	3.48E-06	0.058	0.013	-0.010	0.018
rs11065264	T	G	3.73E-06	0.057	0.012	-0.114	0.052
rs111865527	T	C	1.88E-06	0.271	0.057	0.154	0.068
rs112742549	T	C	1.71E-06	0.242	0.051	0.025	0.094
rs12058421	A	C	2.80E-15	-0.126	0.016	0.015	0.023
rs13014154	A	G	6.50E-07	-0.061	0.012	0.005	0.017
rs13147502	A	G	1.62E-06	-0.061	0.013	-0.009	0.017
rs138657007	A	G	6.98E-07	-0.286	0.058	-0.040	0.092
rs147616029	T	C	4.87E-07	-0.064	0.013	0.004	0.017
rs149092077	A	G	8.63E-07	-0.359	0.073	0.248	0.141
rs1609310	T	G	3.46E-06	-0.056	0.012	0.021	0.017
rs17720330	C	G	4.04E-06	-0.198	0.043	0.025	0.064
rs192245879	A	G	3.76E-07	0.333	0.066	-0.015	0.127
rs1972433	A	C	5.73E-07	0.066	0.013	-0.003	0.097
rs2623960	T	C	1.50E-06	0.069	0.014	0.014	0.020
rs2790462	T	C	3.06E-09	-0.075	0.013	-0.001	0.019
rs35271532	A	G	4.51E-06	-0.149	0.032	-0.094	0.048
rs3745163	A	C	3.35E-06	0.067	0.015	-0.022	0.019
rs3806818	A	G	3.82E-06	0.071	0.015	-0.016	0.021
rs56244095	A	C	8.38E-11	0.101	0.016	-0.011	0.019
rs570025519	A	G	7.96E-16	0.132	0.016	-0.003	0.118
rs60094514	T	C	2.45E-42	0.221	0.016	-0.168	0.138
rs73151926	T	C	3.45E-06	0.106	0.023	-0.054	0.038
rs73231041	C	G	3.05E-06	-0.279	0.060	0.154	0.103
rs7745783	T	C	7.44E-07	-0.059	0.012	-0.005	0.016
rs79348253	A	G	7.75E-08	-0.159	0.030	-0.007	0.035
**STAMBP (F-statistic = 23.45, R2=2.03%)**							
rs1034059	A	G	1.64E-06	0.070	0.015	0.038	0.019
rs10426867	A	G	3.36E-06	0.203	0.044	-0.240	0.337

Supplementary Table 1. Details of systemic inflammatory proteins predicting SNPs with Hashimoto's Thyroiditis from GWAS (with SNPs reaching $P < 5\times10^{-6}$)

Inflammatory proteins	Hashimoto's Thyroiditis						
SNP	Effect allele	Other allele	P	Beta	Standard Error	log(OR)	Standard Error
rs111581172	A	G	9.98E-07	-0.172	0.035	-0.072	0.065
rs111872246	A	G	2.41E-07	0.220	0.043	-0.063	0.053
rs116198125	T	C	4.97E-06	-0.148	0.033	-0.040	0.051
rs12152184	A	G	3.83E-06	0.096	0.021	-0.043	0.026
rs12531843	C	G	2.63E-07	-0.125	0.024	0.056	0.030
rs138897138	A	G	6.46E-07	0.109	0.022	0.032	0.031
rs140656901	A	G	2.19E-06	0.129	0.027	0.039	0.202
rs2280441	A	G	3.69E-07	-0.157	0.031	0.011	0.043
rs73327656	A	G	4.44E-06	0.235	0.051	-0.110	0.096
rs75610895	T	C	1.28E-06	-0.191	0.039	-0.034	0.087
**SULT1A1 (F-statistic = 26.48, R2=7.54%)**							
rs10842189	T	C	3.76E-06	-0.080	0.017	-0.013	0.020
rs11074902	A	G	5.73E-07	0.070	0.014	-0.014	0.017
rs117222566	A	G	3.10E-06	0.271	0.058	-0.068	0.083
rs149278	T	C	3.58E-44	-0.188	0.014	0.003	0.096
rs149869672	T	C	3.24E-06	-0.318	0.068	0.023	0.113
rs1698742	T	C	1.78E-06	-0.098	0.021	0.045	0.032
rs2213907	A	G	2.25E-06	-0.150	0.032	0.047	0.046
rs232928	T	C	4.80E-06	0.062	0.014	-0.004	0.017
rs2691531	T	C	1.77E-06	-0.065	0.014	0.077	0.098
rs2712255	A	G	1.79E-06	0.070	0.015	0.004	0.017
rs2731674	T	G	4.75E-08	-0.083	0.015	-0.012	0.019
rs371387526	A	C	9.72E-07	0.105	0.021	-0.019	0.024
rs55793562	A	G	3.43E-06	0.092	0.020	0.065	0.027
rs55939507	A	G	3.72E-06	0.077	0.017	-0.003	0.018
rs66530140	T	C	5.64E-35	-0.168	0.014	-0.013	0.017
rs703595	T	C	1.15E-06	-0.072	0.015	0.007	0.017
rs710446	T	C	1.33E-07	-0.071	0.014	0.007	0.017
rs72643498	A	C	1.69E-06	-0.180	0.038	0.028	0.031
rs72656644	C	G	1.52E-06	0.108	0.022	0.033	0.026
rs76833948	A	T	2.58E-06	0.134	0.029	0.034	0.026

Supplementary Table 1. Details of systemic inflammatory proteins predicting SNPs with Hashimoto's Thyroiditis from GWAS (with SNPs reaching *P*< 5×10^{-6})

Inflammatory proteins	Hashimoto's Thyroiditis						
SNP	Effect allele	Other allele	P	Beta	Standard Error	log(OR)	Standard Error
rs77919742	T	C	5.94E-07	-0.143	0.029	0.030	0.032
rs7812322	T	C	4.08E-06	-0.348	0.076	-0.060	0.128
rs9804965	A	C	9.40E-08	-0.193	0.036	0.005	0.032
**TGF-α (F-statistic = 26.89, R2=2.58%)**							
rs11242125	A	C	4.55E-06	-0.076	0.017	-0.095	0.113
rs12270510	T	C	4.48E-07	0.096	0.019	0.017	0.025
rs149146289	A	G	5.19E-07	0.286	0.057	0.097	0.065
rs1963491	C	G	2.60E-06	0.108	0.023	0.002	0.028
rs34007466	A	G	7.96E-08	-0.268	0.050	-0.145	0.092
rs34630685	C	G	3.35E-06	0.168	0.036	0.124	0.236
rs3859189	A	G	1.64E-08	0.066	0.012	0.004	0.017
rs4934441	A	G	1.60E-06	-0.095	0.020	0.013	0.021
rs72912115	A	T	9.65E-14	0.137	0.018	-0.033	0.027
rs74130123	T	C	3.05E-07	-0.260	0.051	0.035	0.049
rs78464136	A	T	3.19E-06	0.143	0.031	-0.049	0.045
rs78912149	C	G	1.53E-06	-0.269	0.056	0.138	0.058
rs9262670	T	C	9.06E-07	0.072	0.015	0.017	0.022
**TNF-β (F-statistic = 75.14, R2=15.15%)**							
rs112317147	T	C	3.11E-06	-0.144	0.031	0.031	0.029
rs113244922	T	C	3.20E-06	0.232	0.050	0.128	0.084
rs114250919	T	C	4.69E-07	0.247	0.049	-0.076	0.092
rs12743015	C	G	1.94E-06	-0.192	0.040	0.004	0.073
rs12883833	A	G	1.34E-06	-0.125	0.026	-0.006	0.025
rs138471526	T	C	3.31E-06	-0.342	0.074	0.126	0.184
rs139511528	A	G	1.85E-06	-0.249	0.052	-0.092	0.123
rs143019966	T	C	2.54E-06	0.212	0.045	0.075	0.093
rs147423408	A	G	7.48E-07	0.173	0.035	-0.031	0.035
rs16852556	T	C	1.79E-06	0.151	0.032	-0.022	0.062
rs192303297	T	C	4.25E-06	-0.387	0.084	-0.047	0.219
rs2364485	A	C	1.32E-20	0.176	0.019	-0.008	0.022
rs2523882	T	C	1.77E-43	-0.199	0.014	0.020	0.020

Supplementary Table 1. Details of systemic inflammatory proteins predicting SNPs with Hashimoto's Thyroiditis from GWAS (with SNPs reaching *P*< 5×10^{-6})

Inflammatory proteins	Hashimoto's Thyroiditis						
SNP	Effect allele	Other allele	P	Beta	Standard Error	log(OR)	Standard Error
rs28700215	T	C	4.17E-06	-0.063	0.014	0.016	0.016
rs2904602	A	G	1.00E-200	-0.625	0.020	-0.015	0.024
rs537860389	C	G	3.10E-06	-0.229	0.049	0.028	0.033
rs542608643	T	C	2.74E-14	-0.355	0.047	-0.460	0.297
rs55914821	A	G	2.55E-06	0.075	0.016	-0.013	0.018
rs72854560	C	G	8.21E-07	-0.297	0.060	0.034	0.087
rs74363807	T	C	2.71E-06	0.250	0.053	-0.063	0.083
rs7583622	A	G	3.38E-06	0.075	0.016	-0.011	0.019
rs76913603	A	G	4.36E-06	-0.174	0.038	-0.015	0.063
rs77897196	T	C	2.93E-06	-0.192	0.041	0.073	0.057
rs9267798	C	G	4.71E-07	-0.120	0.024	0.002	0.032
**TNF (F-statistic = 23.37, R2=3.97%)**							
rs117427219	A	G	1.13E-06	-0.306	0.063	-0.114	0.161
rs12328100	A	G	5.97E-07	-0.065	0.013	-0.030	0.017
rs12354434	A	G	1.03E-06	-0.121	0.025	-0.024	0.030
rs1266163	A	T	1.90E-06	0.137	0.029	0.046	0.050
rs12975019	C	G	6.14E-07	-0.113	0.023	0.003	0.026
rs1377015	A	G	4.25E-06	-0.065	0.014	-0.017	0.019
rs138684734	A	G	2.44E-06	0.251	0.053	-0.048	0.110
rs141295666	T	C	2.48E-07	0.341	0.066	-0.100	0.108
rs148560093	A	G	3.97E-06	-0.298	0.065	-0.283	0.129
rs333835	A	T	3.88E-07	0.129	0.025	0.038	0.032
rs4835254	T	C	9.90E-07	-0.152	0.031	-0.014	0.039
rs537908127	T	C	1.87E-07	0.246	0.047	-0.140	0.096
rs6138961	C	G	2.19E-06	0.510	0.108	0.583	0.747
rs7621032	T	C	5.96E-07	0.067	0.014	0.021	0.017
rs7806921	T	C	3.73E-06	-0.061	0.013	-0.017	0.017
rs867063	T	C	4.07E-06	0.118	0.026	0.036	0.028
rs9614693	T	C	3.06E-06	0.083	0.018	-0.005	0.023
rs9913313	T	C	4.92E-06	-0.084	0.018	0.007	0.018
**TNFRSF9 (F-statistic = 25.96, R2=6.22%)**							

Supplementary Table 1. Details of systemic inflammatory proteins predicting SNPs with Hashimoto's Thyroiditis from GWAS (with SNPs reaching $P< 5\times10^{-6}$)

Inflammatory proteins	Hashimoto's Thyroiditis						
SNP	Effect allele	Other allele	P	Beta	Standard Error	log(OR)	Standard Error
rs10101219	C	G	2.13E-06	-0.402	0.085	-0.026	0.074
rs10178845	A	G	2.18E-06	-0.068	0.014	-0.043	0.017
rs10744409	A	G	1.03E-06	0.064	0.013	-0.002	0.017
rs11202989	T	C	2.69E-06	-0.100	0.021	0.016	0.021
rs118083884	A	G	8.83E-07	0.328	0.067	-0.119	0.098
rs12227836	A	G	1.77E-06	-0.067	0.014	0.016	0.017
rs12874404	A	G	2.17E-06	-0.140	0.030	-0.078	0.047
rs140214797	T	G	4.28E-06	0.232	0.050	-0.009	0.070
rs143274881	A	T	3.76E-06	-0.333	0.072	0.035	0.058
rs146380867	A	G	4.29E-06	0.374	0.081	0.157	0.142
rs150949259	A	G	3.56E-06	0.375	0.081	0.047	0.072
rs1645331	C	G	2.49E-06	0.097	0.021	-0.303	0.140
rs1776354	A	G	1.41E-23	-0.130	0.013	-0.045	0.017
rs1846520	A	G	2.05E-07	-0.163	0.031	0.037	0.034
rs192538756	A	T	8.96E-07	0.119	0.024	0.021	0.025
rs2579649	A	C	1.15E-06	-0.097	0.020	-0.045	0.023
rs4976685	A	G	6.18E-07	-0.069	0.014	-0.022	0.018
rs57700441	C	G	4.07E-07	-0.116	0.023	-0.017	0.022
rs72850206	A	T	3.10E-07	-0.144	0.028	0.048	0.042
rs72964492	A	C	1.70E-06	0.311	0.065	-0.261	0.150
rs75246422	T	C	1.32E-06	-0.098	0.020	0.011	0.024
rs76428106	T	C	4.72E-06	-0.271	0.059	-0.196	0.078
rs7737635	A	G	4.29E-06	0.092	0.020	-0.011	0.024
rs7858310	A	G	3.30E-06	0.071	0.015	0.007	0.018
rs8005108	C	G	1.04E-06	0.089	0.018	-0.016	0.076
rs9669611	T	C	2.78E-06	0.070	0.015	0.003	0.019
**TNFSF12 (F-statistic = 36.41, R2=7.62%)**							
rs10831650	A	G	3.09E-08	0.069	0.013	0.033	0.018
rs11136339	T	C	5.05E-07	0.061	0.012	0.033	0.017
rs111641307	C	G	4.15E-06	-0.401	0.087	0.208	0.132
rs11738159	T	C	1.24E-06	0.058	0.012	0.017	0.017

Supplementary Table 1. Details of systemic inflammatory proteins predicting SNPs with Hashimoto's Thyroiditis from GWAS (with SNPs reaching $P < 5\times10^{-6}$)

Inflammatory proteins	Hashimoto's Thyroiditis						
SNP	Effect allele	Other allele	P	Beta	Standard Error	log(OR)	Standard Error
rs117550445	C	G	8.64E-07	-0.186	0.038	0.001	0.049
rs12145644	A	T	2.52E-07	-0.073	0.014	0.012	0.019
rs12726054	A	G	1.75E-06	0.109	0.023	0.014	0.026
rs13107325	T	C	8.77E-18	0.203	0.024	-0.105	0.069
rs16879825	A	G	3.69E-06	-0.203	0.044	-0.041	0.031
rs1965399	A	G	2.79E-06	-0.131	0.028	-0.034	0.053
rs201620545	A	G	4.34E-07	-0.085	0.017	-0.049	0.059
rs2163333	A	G	2.71E-07	0.140	0.027	0.006	0.043
rs2738752	A	G	4.62E-06	-0.059	0.013	0.079	0.065
rs34790908	T	C	3.90E-60	0.211	0.013	0.016	0.107
rs3821473	A	G	4.80E-06	-0.062	0.014	0.016	0.019
rs4882710	T	C	3.70E-07	0.067	0.013	-0.001	0.017
rs552941568	A	C	3.22E-06	0.102	0.022	-0.090	0.162
rs56023600	T	C	4.18E-06	-0.086	0.019	-0.020	0.024
rs575752535	T	C	1.57E-06	-0.226	0.047	-0.024	0.315
rs579459	T	C	2.68E-12	0.110	0.016	-0.016	0.020
rs58743186	A	G	3.23E-06	-0.072	0.015	0.122	0.069
rs7019909	T	C	3.36E-06	-0.090	0.019	-0.022	0.024
rs73590361	T	C	3.82E-06	-0.074	0.016	0.031	0.024
rs7503751	A	G	5.76E-09	-0.139	0.024	-0.105	0.039
rs76047473	T	C	4.06E-06	0.132	0.029	-0.068	0.041
rs76133364	A	C	2.77E-07	0.190	0.037	-0.009	0.040
rs78254232	T	C	3.79E-06	-0.211	0.046	-0.065	0.094
rs826688	A	T	2.18E-06	-0.059	0.013	-0.003	0.018
rs9842051	C	G	1.68E-17	0.119	0.014	-0.020	0.022
**TNFSF14 (F-statistic = 36.42, R2=8.37%)**							
rs10211598	T	C	4.56E-06	-0.063	0.014	-0.017	0.017
rs11085155	A	G	4.07E-06	-0.081	0.018	-0.007	0.021
rs11105725	T	C	3.38E-06	-0.068	0.015	-0.004	0.019
rs11569551	A	G	2.74E-15	0.258	0.033	-0.050	0.037
rs115950744	A	G	3.64E-07	-0.257	0.051	-0.111	0.084

Supplementary Table 1. Details of systemic inflammatory proteins predicting SNPs with Hashimoto's Thyroiditis from GWAS (with SNPs reaching *P*< 5×10⁻⁶)

Inflammatory proteins	Hashimoto's Thyroiditis						
SNP	Effect allele	Other allele	P	Beta	Standard Error	log(OR)	Standard Error
rs117442125	A	T	3.35E-06	0.169	0.036	-0.053	0.040
rs117720118	T	G	1.55E-06	-0.146	0.030	-0.002	0.042
rs11950562	A	C	2.73E-09	0.079	0.013	0.011	0.018
rs1461044	T	C	1.53E-06	-0.152	0.032	0.005	0.032
rs147119146	A	C	1.17E-06	-0.242	0.050	-0.017	0.044
rs184708984	T	C	1.12E-08	-0.633	0.111	0.247	0.264
rs2738591	T	C	2.17E-06	-0.063	0.013	-0.020	0.016
rs344560	T	C	7.29E-72	-0.489	0.027	0.014	0.035
rs34925583	C	G	4.39E-06	-0.070	0.015	-0.001	0.020
rs4772260	T	C	4.54E-06	0.062	0.014	-0.021	0.017
rs59801342	A	G	4.07E-06	0.229	0.050	-0.026	0.057
rs74600570	T	C	1.77E-06	-0.226	0.047	-0.054	0.047
rs76688046	T	C	1.59E-06	0.103	0.022	0.110	0.138
rs76778362	C	G	8.51E-07	-0.212	0.043	0.047	0.109
rs7688836	A	G	8.32E-07	-0.082	0.017	-0.006	0.020
rs78336300	A	C	4.60E-06	0.239	0.052	0.006	0.066
rs79233212	A	G	3.16E-06	-0.236	0.051	0.063	0.066
rs79791117	T	C	4.84E-06	-0.234	0.051	-0.105	0.083
rs9295562	T	C	3.89E-06	-0.065	0.014	-0.045	0.018
rs9961731	A	G	4.69E-07	0.105	0.021	0.034	0.133
rs9962377	T	C	1.14E-06	-0.091	0.019	-0.037	0.029
**TRAIL (F-statistic = 68.14, R2=12.40%)**							
rs1065657	A	G	2.84E-08	0.066	0.012	0.005	0.017
rs10745981	A	T	1.92E-06	-0.088	0.019	0.031	0.028
rs111257277	A	G	3.25E-07	0.189	0.037	-0.054	0.052
rs114597610	T	C	4.03E-06	-0.260	0.056	0.112	0.077
rs140862652	A	C	4.10E-07	0.134	0.027	-0.046	0.026
rs141011299	A	G	3.02E-06	-0.502	0.108	0.280	0.240
rs142889372	C	G	2.89E-06	-0.284	0.061	-0.090	0.063
rs145386370	T	G	7.96E-07	0.264	0.054	0.057	0.063
rs146922303	T	C	2.95E-06	0.162	0.035	0.164	0.294

Supplementary Table 1. Details of systemic inflammatory proteins predicting SNPs with Hashimoto's Thyroiditis from GWAS (with SNPs reaching $P < 5\times10^{-6}$)

Inflammatory proteins	Hashimoto's Thyroiditis						
SNP	Effect allele	Other allele	P	Beta	Standard Error	log(OR)	Standard Error
rs16840522	T	C	5.40E-16	-0.119	0.015	0.006	0.025
rs174533	A	G	7.81E-11	0.079	0.012	-0.006	0.017
rs17779404	T	G	1.04E-06	0.067	0.014	-0.009	0.021
rs180941492	A	G	6.71E-07	-0.244	0.049	-0.034	0.093
rs2113173	T	C	1.88E-06	0.069	0.015	0.024	0.019
rs2138380	A	G	1.26E-06	0.088	0.018	-0.037	0.022
rs2418781	T	G	4.33E-06	-0.145	0.032	0.009	0.045
rs28424593	A	T	2.62E-06	-0.131	0.028	0.011	0.035
rs28929474	T	C	3.08E-52	0.669	0.044	0.020	0.059
rs4760	A	G	2.14E-105	0.355	0.016	-0.012	0.023
rs490342	T	G	3.85E-10	0.125	0.020	-0.039	0.036
rs5030044	A	G	1.90E-52	0.285	0.019	-0.039	0.024
rs574044675	A	C	3.69E-48	0.582	0.040	0.011	0.052
rs6969337	T	C	1.61E-06	0.182	0.038	-0.015	0.032
rs77303550	T	C	7.09E-07	-0.072	0.015	-0.061	0.021
rs78956561	A	T	1.71E-06	0.169	0.035	0.001	0.039
rs8178824	T	C	3.42E-12	0.258	0.037	-0.081	0.088
**TRANCE (F-statistic = 44.13, R2=9.10%)**							
rs113835939	A	C	3.15E-06	-0.194	0.042	-0.010	0.043
rs117113213	A	G	4.10E-08	-0.165	0.030	-0.040	0.035
rs11713634	A	G	7.03E-11	-0.098	0.015	0.016	0.017
rs1432791	T	C	3.28E-06	-0.055	0.012	-0.021	0.016
rs150128402	A	G	2.01E-07	0.239	0.046	0.076	0.055
rs150416959	C	G	3.46E-06	0.146	0.032	0.040	0.045
rs17221073	C	G	1.08E-07	0.265	0.050	0.275	0.128
rs181906998	A	T	7.69E-07	-0.104	0.021	0.018	0.019
rs183217699	C	G	4.02E-06	-0.125	0.027	0.077	0.098
rs1839438	T	G	2.12E-06	-0.068	0.014	-0.027	0.101
rs201831835	A	C	1.35E-06	0.138	0.029	-0.033	0.235
rs2468187	T	C	8.62E-43	-0.162	0.012	0.003	0.018
rs2975421	T	G	4.47E-06	-0.078	0.017	-0.009	0.023

Supplementary Table 1. Details of systemic inflammatory proteins predicting SNPs with Hashimoto's Thyroiditis from GWAS (with SNPs reaching $P< 5\times10^{-6}$)

Inflammatory proteins	Hashimoto's Thyroiditis						
SNP	Effect allele	Other allele	P	Beta	Standard Error	log(OR)	Standard Error
rs34221978	A	G	1.21E-06	0.167	0.034	0.006	0.037
rs3801000	A	G	1.31E-07	0.069	0.013	-0.007	0.017
rs4334157	T	C	2.24E-06	-0.168	0.036	0.007	0.057
rs4365726	A	G	2.86E-06	-0.078	0.017	-0.039	0.022
rs4512994	A	C	5.33E-24	0.117	0.012	-0.041	0.017
rs5001566	A	T	4.54E-06	0.122	0.027	0.167	0.218
rs6063153	T	C	4.47E-07	0.064	0.013	-0.017	0.018
rs62501135	A	G	4.13E-25	-0.153	0.015	0.049	0.022
rs6479556	T	G	2.25E-06	0.070	0.015	0.009	0.021
rs6932751	A	G	3.24E-06	0.054	0.012	0.027	0.052
rs704	A	G	8.48E-21	-0.109	0.012	0.042	0.017
rs72781813	T	G	1.10E-06	0.237	0.049	-0.093	0.063
rs75991751	T	G	6.31E-07	-0.269	0.054	-0.097	0.110
rs76488966	T	G	2.64E-06	-0.172	0.037	0.057	0.040
rs77980965	T	C	3.44E-06	-0.298	0.064	0.042	0.085
rs79287178	A	G	6.82E-48	-0.499	0.034	0.008	0.043
**TSLP (F-statistic = 23.05, R2=3.14%)**							
rs112206094	A	T	3.37E-06	-0.093	0.020	-0.035	0.025
rs117095006	A	G	3.45E-06	-0.157	0.034	-0.035	0.048
rs118119134	A	G	2.73E-06	-0.442	0.094	-0.025	0.192
rs12448125	T	G	1.17E-06	0.178	0.037	-0.053	0.043
rs12472785	C	G	1.32E-06	-0.074	0.015	0.008	0.018
rs140886020	A	G	3.98E-06	0.162	0.035	0.123	0.049
rs145111430	A	G	3.31E-06	0.267	0.058	-0.096	0.104
rs185922111	A	G	1.28E-06	0.362	0.075	0.110	0.097
rs190906347	A	C	1.08E-06	0.322	0.066	-0.178	0.132
rs4724126	T	G	2.61E-08	0.085	0.015	0.000	0.018
rs72652144	A	G	2.34E-06	0.163	0.035	-0.011	0.034
rs72811179	T	C	2.70E-06	-0.296	0.063	0.031	0.066
rs7406537	A	G	3.73E-06	-0.064	0.014	-0.024	0.016
rs75129390	A	T	2.57E-06	0.074	0.016	-0.158	0.099

Supplementary Table 1. Details of systemic inflammatory proteins predicting SNPs with Hashimoto's Thyroiditis from GWAS (with SNPs reaching $P< 5\times10^{-6}$)

Inflammatory proteins	Hashimoto's Thyroiditis						
SNP	Effect allele	Other allele	P	Beta	Standard Error	log(OR)	Standard Error
rs7550374	C	G	6.96E-07	-0.093	0.019	-0.010	0.024
**uPA (F-statistic = 39.83, R2=6.52%)**							
rs1074669	A	G	4.12E-06	-0.055	0.012	0.007	0.016
rs10840566	A	G	3.56E-07	0.078	0.015	0.024	0.025
rs10992204	A	G	1.01E-06	0.106	0.022	-0.040	0.025
rs11220462	A	G	2.01E-10	-0.102	0.016	-0.012	0.021
rs13299342	A	G	4.04E-09	0.100	0.017	-0.002	0.020
rs144470316	C	G	2.28E-07	0.340	0.066	0.009	0.123
rs154972	A	G	1.91E-07	-0.063	0.012	0.032	0.019
rs202063542	T	C	4.99E-06	0.277	0.061	-0.557	0.423
rs2227575	A	C	1.93E-16	-0.095	0.012	0.012	0.065
rs333947	A	G	2.26E-06	-0.079	0.017	0.034	0.022
rs3933086	A	G	3.05E-06	0.310	0.066	0.039	0.043
rs4251805	T	C	6.10E-42	-0.422	0.031	0.081	0.046
rs4760	A	G	1.42E-17	0.141	0.017	-0.012	0.023
rs4800787	T	C	5.48E-14	-0.094	0.013	0.023	0.017
rs577588918	C	G	4.43E-06	0.209	0.046	0.087	0.185
rs59772299	T	G	2.43E-06	0.063	0.013	-0.004	0.020
rs62286554	T	C	1.67E-06	-0.091	0.019	-0.017	0.021
rs6805474	A	G	6.33E-08	0.095	0.018	0.035	0.023
rs704	A	G	8.20E-07	0.057	0.012	0.042	0.017
rs72727847	C	G	2.37E-06	0.197	0.042	0.103	0.436
rs7406661	T	C	1.28E-12	-0.111	0.016	-0.171	0.120
rs74607099	A	G	3.00E-06	-0.182	0.039	-0.009	0.047
rs7564243	A	G	7.80E-14	-0.085	0.011	0.009	0.017
**VEFG-A (F-statistic = 66.04, R2=10.74%)**							
rs10822155	A	C	7.93E-13	0.084	0.012	0.016	0.017
rs10840177	C	G	1.51E-06	-0.068	0.014	0.071	0.106
rs114694170	T	C	1.16E-09	-0.163	0.027	0.033	0.037
rs117976551	C	G	1.45E-06	-0.216	0.045	-0.078	0.071
rs11953790	T	C	8.43E-07	-0.184	0.037	0.091	0.084

Supplementary Table 1. Details of systemic inflammatory proteins predicting SNPs with Hashimoto's Thyroiditis from GWAS (with SNPs reaching *P*< 5×10⁻⁶)

Inflammatory proteins	Hashimoto's Thyroiditis						
SNP	Effect allele	Other allele	P	Beta	Standard Error	log(OR)	Standard Error
rs1203834	T	C	3.48E-06	0.117	0.025	0.009	0.030
rs1208181	T	C	1.95E-06	-0.079	0.017	0.241	0.128
rs12784822	A	G	4.93E-06	-0.327	0.072	0.162	0.086
rs138648222	C	G	6.04E-07	0.198	0.040	0.027	0.070
rs144037665	A	C	4.05E-06	-0.278	0.060	0.077	0.093
rs145703410	T	C	4.39E-06	-0.248	0.054	-0.091	0.060
rs145846107	T	C	2.59E-06	0.274	0.058	0.104	0.136
rs16883415	T	C	1.48E-06	-0.220	0.046	0.007	0.070
rs2050256	A	G	1.37E-06	-0.073	0.015	-0.017	0.020
rs34536806	T	G	1.85E-06	0.099	0.021	-0.041	0.032
rs4875579	C	G	2.73E-06	-0.074	0.016	-0.007	0.023
rs61818787	T	C	2.92E-07	-0.076	0.015	-0.044	0.019
rs62640435	T	C	1.27E-07	-0.067	0.013	0.060	0.104
rs6582666	C	G	1.17E-06	-0.129	0.027	-0.040	0.031
rs6993770	A	T	4.06E-20	0.123	0.013	0.015	0.019
rs7688836	A	G	2.31E-06	-0.069	0.015	-0.006	0.020
rs7808444	A	C	4.56E-06	-0.063	0.014	-0.138	0.105
rs9349270	A	C	2.00E-08	0.106	0.019	-0.060	0.027
rs9369434	T	C	1.00E-200	-0.369	0.012	-0.005	0.017
**β-NGF (F-statistic = 24.42, R2=3.97%)**							
rs117164089	T	C	1.61E-06	-0.271	0.057	-0.186	0.140
rs12188775	T	G	9.06E-07	0.121	0.025	-0.016	0.035
rs12259196	A	G	4.03E-07	-0.075	0.015	-0.005	0.023
rs12533747	A	G	4.84E-07	0.139	0.028	-0.006	0.034
rs12539643	C	G	4.74E-06	0.187	0.041	-0.037	0.035
rs13058191	C	G	3.59E-06	-0.216	0.047	0.019	0.065
rs140072192	A	G	4.10E-06	0.277	0.060	-0.098	0.139
rs148834134	T	C	4.87E-06	0.254	0.056	-0.085	0.124
rs17647697	A	C	4.35E-06	-0.162	0.035	-0.060	0.049
rs17689534	A	G	1.76E-06	0.221	0.046	-0.006	0.088
rs182658308	A	T	1.97E-06	-0.206	0.043	-0.118	0.528

Supplementary Table 1. Details of systemic inflammatory proteins predicting SNPs with Hashimoto's Thyroiditis from GWAS (with SNPs reaching *P*< 5×10^{-6})

Inflammatory proteins	Hashimoto's Thyroiditis						
SNP	Effect allele	Other allele	P	Beta	Standard Error	log(OR)	Standard Error
rs1878359	A	G	6.76E-07	0.063	0.013	-0.034	0.018
rs3128517	T	C	5.11E-11	-0.078	0.012	-0.032	0.016
rs34559191	T	C	9.82E-07	0.061	0.012	-0.001	0.018
rs397081	T	C	4.40E-06	0.120	0.026	0.102	0.040
rs4017	T	C	4.21E-06	-0.119	0.026	-0.067	0.051
rs62092633	T	C	1.42E-06	-0.125	0.026	0.033	0.025
rs62494116	T	C	7.81E-07	0.058	0.012	-0.002	0.018
rs6328	A	C	6.46E-11	-0.080	0.012	-0.025	0.017
rs72673086	A	G	3.57E-06	0.058	0.013	-0.012	0.017
rs77493302	T	C	4.01E-06	0.085	0.019	0.056	0.027
rs9479501	T	G	4.41E-06	0.063	0.014	-0.010	0.020

Supplementary Table 2. Details of Hashimoto's Thyroiditis predicting SNPs with inflammatory proteins from IEU Open GWAS (with SNPs reaching $P < 5 \times 10^{-6}$).

Inflammatory proteins						Hashimoto's Thyroiditis	
SNP	Effect allele	Other allele	P value	Beta	Standard Error	log(OR)	Standard Error
**ADA (F-statistic = 38.11, R2=0.44%)**							
rs10424978	A	C	8.97E-07	-0.08	0.017	-0.015	0.012
rs10494077	A	G	9.20E-10	0.12	0.020	0.013	0.014
rs10786679	A	G	1.87E-06	-0.09	0.018	-0.012	0.014
rs111358861	G	A	3.30E-06	1.82	0.391	-0.096	0.107
rs1115587	A	G	1.82E-07	-0.10	0.018	0.023	0.012
rs11211645	G	A	9.33E-08	0.09	0.017	-0.008	0.012
rs113075322	G	C	7.18E-07	-0.14	0.028	0.002	0.033
rs11611029	T	C	1.16E-08	-0.10	0.017	-0.019	0.012
rs117167029	C	G	8.05E-07	2.99	0.606	0.049	0.091
rs117369326	A	G	3.55E-06	0.24	0.051	-0.028	0.029
rs118075239	C	T	3.45E-06	0.38	0.083	0.014	0.047
rs11822254	G	A	3.52E-06	-0.08	0.017	0.005	0.012
rs11889341	T	C	6.64E-11	0.12	0.019	-0.007	0.014
rs1317983	C	T	1.65E-07	0.09	0.018	-0.027	0.013
rs13249705	G	A	3.78E-07	-0.09	0.018	0.000	0.014
rs140111921	A	G	1.34E-06	-0.16	0.034	0.039	0.031
rs150619443	C	G	5.08E-07	0.44	0.088	-0.053	0.054
rs1534430	T	C	9.40E-08	-0.09	0.017	0.002	0.012
rs17490285	G	A	9.26E-07	-0.18	0.038	-0.012	0.019
rs1887414	C	T	1.97E-07	0.12	0.022	-0.008	0.016
rs2111485	G	A	4.19E-06	0.08	0.017	-0.009	0.012
rs229526	C	G	1.93E-06	0.10	0.022	-0.003	0.015
rs2476601	G	A	3.49E-45	-0.33	0.023	0.037	0.019
rs28367132	A	G	1.21E-07	-0.14	0.026	0.003	0.015
rs28450758	A	C	4.17E-06	0.14	0.031	0.007	0.022
rs3184504	C	T	1.40E-25	-0.18	0.017	-0.019	0.013
rs34536443	C	G	2.47E-07	-0.25	0.049	-0.020	0.030
rs34636506	G	A	1.92E-14	-0.13	0.018	-0.003	0.012
rs61830417	G	A	3.59E-06	0.27	0.059	-0.005	0.041
rs62076103	G	A	1.23E-07	-0.19	0.035	0.011	0.024

Supplementary Table 2. Details of Hashimoto's Thyroiditis predicting SNPs with inflammatory proteins from IEU Open GWAS (with SNPs reaching $P < 5 \times 10^{-6}$).

Inflammatory proteins						Hashimoto's Thyroiditis	
SNP	Effect allele	Other allele	P value	Beta	Standard Error	log(OR)	Standard Error
rs6448432	A	G	1.75E-06	0.09	0.019	0.018	0.013
rs6501550	T	C	1.42E-06	-0.18	0.037	-0.005	0.035
rs7030280	T	C	6.44E-31	0.21	0.018	-0.019	0.013
rs72808590	C	T	1.55E-06	-0.37	0.077	-0.008	0.044
rs73165154	G	A	3.76E-06	-0.15	0.033	-0.011	0.022
rs73192661	T	C	1.45E-09	-0.10	0.017	-0.029	0.012
rs7335748	G	A	2.41E-06	0.38	0.080	0.029	0.040
rs766932	T	A	4.96E-07	0.10	0.019	-0.012	0.013
rs7767668	T	C	9.60E-07	-0.09	0.019	0.002	0.012
rs7861040	C	G	9.08E-07	0.08	0.017	-0.011	0.012
rs79219789	C	G	2.84E-06	-0.15	0.033	0.047	0.030
rs8084503	T	C	1.97E-07	0.14	0.027	0.019	0.018
rs847803	G	A	7.91E-07	0.09	0.018	0.008	0.013
rs9271365	G	T	1.44E-39	0.25	0.019	0.004	0.012
rs9357119	T	G	3.65E-14	-0.15	0.020	-0.007	0.013
rs9497965	T	C	1.71E-06	0.08	0.017	-0.006	0.012
**ARTN (F-statistic = 38.11, R2=0.44%)**							
rs10424978	A	C	8.97E-07	-0.08	0.017	0.018	0.014
rs10494077	A	G	9.20E-10	0.12	0.020	-0.006	0.016
rs10786679	A	G	1.87E-06	-0.09	0.018	0.007	0.015
rs111358861	G	A	3.30E-06	1.82	0.391	0.264	0.107
rs1115587	A	G	1.82E-07	-0.10	0.018	0.018	0.014
rs11211645	G	A	9.33E-08	0.09	0.017	0.004	0.013
rs113075322	G	C	7.18E-07	-0.14	0.028	0.083	0.036
rs11611029	T	C	1.16E-08	-0.10	0.017	-0.006	0.014
rs117167029	C	G	8.05E-07	2.99	0.606	0.108	0.111
rs117369326	A	G	3.55E-06	0.24	0.051	-0.058	0.033
rs118075239	C	T	3.45E-06	0.38	0.083	0.000	0.051
rs11822254	G	A	3.52E-06	-0.08	0.017	0.002	0.014
rs11889341	T	C	6.64E-11	0.12	0.019	0.010	0.016
rs1317983	C	T	1.65E-07	0.09	0.018	0.016	0.015

Supplementary Table 2. Details of Hashimoto's Thyroiditis predicting SNPs with inflammatory proteins from IEU Open GWAS (with SNPs reaching $P < 5 \times 10^{-6}$).

Inflammatory proteins				Hashimoto's Thyroiditis			
SNP	Effect allele	Other allele	P value	Beta	Standard Error	log(OR)	Standard Error
rs13249705	G	A	3.78E-07	-0.09	0.018	-0.043	0.016
rs140111921	A	G	1.34E-06	-0.16	0.034	-0.038	0.033
rs150619443	C	G	5.08E-07	0.44	0.088	0.033	0.058
rs1534430	T	C	9.40E-08	-0.09	0.017	0.000	0.014
rs17490285	G	A	9.26E-07	-0.18	0.038	-0.007	0.021
rs1887414	C	T	1.97E-07	0.12	0.022	-0.017	0.018
rs2111485	G	A	4.19E-06	0.08	0.017	0.008	0.013
rs229526	C	G	1.93E-06	0.10	0.022	0.003	0.016
rs2476601	G	A	3.49E-45	-0.33	0.023	0.032	0.021
rs28367132	A	G	1.21E-07	-0.14	0.026	-0.014	0.017
rs28450758	A	C	4.17E-06	0.14	0.031	-0.007	0.025
rs3184504	C	T	1.40E-25	-0.18	0.017	-0.010	0.013
rs34536443	C	G	2.47E-07	-0.25	0.049	-0.006	0.033
rs34636506	G	A	1.92E-14	-0.13	0.018	-0.007	0.014
rs61830417	G	A	3.59E-06	0.27	0.059	-0.063	0.046
rs62076103	G	A	1.23E-07	-0.19	0.035	-0.003	0.026
rs6448432	A	G	1.75E-06	0.09	0.019	0.010	0.014
rs6501550	T	C	1.42E-06	-0.18	0.037	0.032	0.040
rs7030280	T	C	6.44E-31	0.21	0.018	0.004	0.014
rs72808590	C	T	1.55E-06	-0.37	0.077	-0.013	0.048
rs73165154	G	A	3.76E-06	-0.15	0.033	-0.012	0.024
rs73192661	T	C	1.45E-09	-0.10	0.017	0.003	0.014
rs7335748	G	A	2.41E-06	0.38	0.080	0.040	0.046
rs766932	T	A	4.96E-07	0.10	0.019	0.018	0.015
rs7767668	T	C	9.60E-07	-0.09	0.019	-0.009	0.013
rs7861040	C	G	9.08E-07	0.08	0.017	-0.006	0.013
rs79219789	C	G	2.84E-06	-0.15	0.033	0.050	0.035
rs8084503	T	C	1.97E-07	0.14	0.027	-0.002	0.021
rs847803	G	A	7.91E-07	0.09	0.018	0.017	0.014
rs9271365	G	T	1.44E-39	0.25	0.019	-0.016	0.013
rs9357119	T	G	3.65E-14	-0.15	0.020	0.001	0.015

Supplementary Table 2. Details of Hashimoto's Thyroiditis predicting SNPs with inflammatory proteins from IEU Open GWAS (with SNPs reaching $P < 5 \times 10^{-6}$).

Inflammatory proteins				Hashimoto's Thyroiditis			
SNP	Effect allele	Other allele	P value	Beta	Standard Error	log(OR)	Standard Error
rs9497965	T	C	1.71E-06	0.08	0.017	0.002	0.013
**AXIN1 (F-statistic = 38.11, R2=0.44%)**							
rs10424978	A	C	8.97E-07	-0.08	0.017	-0.006	0.014
rs10494077	A	G	9.20E-10	0.12	0.020	0.004	0.016
rs10786679	A	G	1.87E-06	-0.09	0.018	-0.014	0.015
rs111358861	G	A	3.30E-06	1.82	0.391	-0.265	0.106
rs1115587	A	G	1.82E-07	-0.10	0.018	0.017	0.014
rs11211645	G	A	9.33E-08	0.09	0.017	-0.016	0.013
rs113075322	G	C	7.18E-07	-0.14	0.028	-0.040	0.036
rs11611029	T	C	1.16E-08	-0.10	0.017	0.013	0.014
rs117167029	C	G	8.05E-07	2.99	0.606	-0.089	0.111
rs117369326	A	G	3.55E-06	0.24	0.051	-0.025	0.033
rs118075239	C	T	3.45E-06	0.38	0.083	-0.050	0.051
rs11822254	G	A	3.52E-06	-0.08	0.017	-0.011	0.014
rs11889341	T	C	6.64E-11	0.12	0.019	-0.010	0.016
rs1317983	C	T	1.65E-07	0.09	0.018	-0.019	0.015
rs13249705	G	A	3.78E-07	-0.09	0.018	-0.019	0.016
rs140111921	A	G	1.34E-06	-0.16	0.034	-0.005	0.033
rs150619443	C	G	5.08E-07	0.44	0.088	-0.019	0.058
rs1534430	T	C	9.40E-08	-0.09	0.017	0.022	0.014
rs17490285	G	A	9.26E-07	-0.18	0.038	-0.029	0.021
rs1887414	C	T	1.97E-07	0.12	0.022	-0.006	0.019
rs2111485	G	A	4.19E-06	0.08	0.017	0.007	0.013
rs229526	C	G	1.93E-06	0.10	0.022	0.001	0.017
rs2476601	G	A	3.49E-45	-0.33	0.023	0.036	0.021
rs28367132	A	G	1.21E-07	-0.14	0.026	0.003	0.017
rs28450758	A	C	4.17E-06	0.14	0.031	-0.012	0.025
rs3184504	C	T	1.40E-25	-0.18	0.017	-0.013	0.013
rs34536443	C	G	2.47E-07	-0.25	0.049	-0.038	0.034
rs34636506	G	A	1.92E-14	-0.13	0.018	-0.003	0.014
rs61830417	G	A	3.59E-06	0.27	0.059	-0.002	0.046

Supplementary Table 2. Details of Hashimoto's Thyroiditis predicting SNPs with inflammatory proteins from IEU Open GWAS (with SNPs reaching $P < 5 \times 10^{-6}$).

Inflammatory proteins				Hashimoto's Thyroiditis			
SNP	Effect allele	Other allele	P value	Beta	Standard Error	log(OR)	Standard Error
rs62076103	G	A	1.23E-07	-0.19	0.035	-0.007	0.026
rs6448432	A	G	1.75E-06	0.09	0.019	0.005	0.014
rs6501550	T	C	1.42E-06	-0.18	0.037	0.034	0.040
rs7030280	T	C	6.44E-31	0.21	0.018	0.007	0.014
rs72808590	C	T	1.55E-06	-0.37	0.077	0.029	0.048
rs73165154	G	A	3.76E-06	-0.15	0.033	-0.012	0.024
rs73192661	T	C	1.45E-09	-0.10	0.017	-0.025	0.014
rs7335748	G	A	2.41E-06	0.38	0.080	-0.024	0.047
rs766932	T	A	4.96E-07	0.10	0.019	-0.025	0.015
rs7767668	T	C	9.60E-07	-0.09	0.019	-0.016	0.013
rs7861040	C	G	9.08E-07	0.08	0.017	0.000	0.014
rs79219789	C	G	2.84E-06	-0.15	0.033	-0.025	0.035
rs8084503	T	C	1.97E-07	0.14	0.027	0.007	0.021
rs847803	G	A	7.91E-07	0.09	0.018	0.002	0.014
rs9271365	G	T	1.44E-39	0.25	0.019	0.008	0.013
rs9357119	T	G	3.65E-14	-0.15	0.020	-0.010	0.015
rs9497965	T	C	1.71E-06	0.08	0.017	-0.008	0.014
**Caspase 8 (F-statistic = 38.11, R2=0.44%)**							
rs10424978	A	C	8.97E-07	-0.08	0.017	-0.004	0.012
rs10494077	A	G	9.20E-10	0.12	0.020	0.018	0.014
rs10786679	A	G	1.87E-06	-0.09	0.018	-0.015	0.013
rs111358861	G	A	3.30E-06	1.82	0.391	-0.218	0.106
rs1115587	A	G	1.82E-07	-0.10	0.018	0.020	0.012
rs11211645	G	A	9.33E-08	0.09	0.017	-0.022	0.012
rs113075322	G	C	7.18E-07	-0.14	0.028	0.005	0.033
rs11611029	T	C	1.16E-08	-0.10	0.017	0.007	0.012
rs117167029	C	G	8.05E-07	2.99	0.606	0.041	0.088
rs117369326	A	G	3.55E-06	0.24	0.051	-0.021	0.029
rs118075239	C	T	3.45E-06	0.38	0.083	-0.007	0.047
rs11822254	G	A	3.52E-06	-0.08	0.017	-0.009	0.012
rs11889341	T	C	6.64E-11	0.12	0.019	-0.001	0.014

Supplementary Table 2. Details of Hashimoto's Thyroiditis predicting SNPs with inflammatory proteins from IEU Open GWAS (with SNPs reaching $P < 5 \times 10^{-6}$).

Inflammatory proteins				Hashimoto's Thyroiditis			
SNP	Effect allele	Other allele	P value	Beta	Standard Error	log(OR)	Standard Error
rs1317983	C	T	1.65E-07	0.09	0.018	-0.006	0.013
rs13249705	G	A	3.78E-07	-0.09	0.018	0.006	0.014
rs140111921	A	G	1.34E-06	-0.16	0.034	0.006	0.030
rs150619443	C	G	5.08E-07	0.44	0.088	-0.072	0.055
rs1534430	T	C	9.40E-08	-0.09	0.017	0.020	0.012
rs17490285	G	A	9.26E-07	-0.18	0.038	-0.005	0.019
rs1887414	C	T	1.97E-07	0.12	0.022	-0.022	0.017
rs2111485	G	A	4.19E-06	0.08	0.017	0.005	0.012
rs229526	C	G	1.93E-06	0.10	0.022	-0.006	0.015
rs2476601	G	A	3.49E-45	-0.33	0.023	0.027	0.019
rs28367132	A	G	1.21E-07	-0.14	0.026	-0.008	0.015
rs28450758	A	C	4.17E-06	0.14	0.031	-0.011	0.023
rs3184504	C	T	1.40E-25	-0.18	0.017	-0.028	0.013
rs34536443	C	G	2.47E-07	-0.25	0.049	-0.010	0.031
rs34636506	G	A	1.92E-14	-0.13	0.018	0.008	0.012
rs61830417	G	A	3.59E-06	0.27	0.059	0.043	0.042
rs62076103	G	A	1.23E-07	-0.19	0.035	-0.017	0.025
rs6448432	A	G	1.75E-06	0.09	0.019	0.027	0.013
rs6501550	T	C	1.42E-06	-0.18	0.037	0.002	0.034
rs7030280	T	C	6.44E-31	0.21	0.018	-0.007	0.013
rs72808590	C	T	1.55E-06	-0.37	0.077	-0.010	0.045
rs73165154	G	A	3.76E-06	-0.15	0.033	-0.039	0.022
rs73192661	T	C	1.45E-09	-0.10	0.017	-0.026	0.012
rs7335748	G	A	2.41E-06	0.38	0.080	-0.009	0.041
rs766932	T	A	4.96E-07	0.10	0.019	-0.006	0.013
rs7767668	T	C	9.60E-07	-0.09	0.019	0.022	0.012
rs7861040	C	G	9.08E-07	0.08	0.017	-0.016	0.012
rs79219789	C	G	2.84E-06	-0.15	0.033	-0.016	0.031
rs8084503	T	C	1.97E-07	0.14	0.027	0.004	0.018
rs847803	G	A	7.91E-07	0.09	0.018	0.022	0.013
rs9271365	G	T	1.44E-39	0.25	0.019	0.009	0.012

Supplementary Table 2. Details of Hashimoto's Thyroiditis predicting SNPs with inflammatory proteins from IEU Open GWAS (with SNPs reaching $P < 5 \times 10^{-6}$).

Inflammatory proteins				Hashimoto's Thyroiditis			
SNP	Effect allele	Other allele	P value	Beta	Standard Error	log(OR)	Standard Error
rs9357119	T	G	3.65E-14	-0.15	0.020	-0.004	0.013
rs9497965	T	C	1.71E-06	0.08	0.017	-0.012	0.012
**CCL19 (F-statistic = 32.82, R2=0.36%)**							
rs10424978	A	C	8.97E-07	-0.08	0.017	-0.008	0.012
rs10494077	A	G	9.20E-10	0.12	0.020	-0.007	0.014
rs10786679	A	G	1.87E-06	-0.09	0.018	-0.002	0.014
rs111358861	G	A	3.30E-06	1.82	0.391	0.054	0.106
rs1115587	A	G	1.82E-07	-0.10	0.018	-0.019	0.012
rs11211645	G	A	9.33E-08	0.09	0.017	0.000	0.012
rs113075322	G	C	7.18E-07	-0.14	0.028	0.037	0.033
rs11611029	T	C	1.16E-08	-0.10	0.017	-0.007	0.012
rs117167029	C	G	8.05E-07	2.99	0.606	0.021	0.090
rs117369326	A	G	3.55E-06	0.24	0.051	-0.032	0.029
rs118075239	C	T	3.45E-06	0.38	0.083	0.023	0.048
rs11822254	G	A	3.52E-06	-0.08	0.017	-0.019	0.012
rs11889341	T	C	6.64E-11	0.12	0.019	0.023	0.014
rs1317983	C	T	1.65E-07	0.09	0.018	-0.005	0.013
rs13249705	G	A	3.78E-07	-0.09	0.018	-0.002	0.014
rs140111921	A	G	1.34E-06	-0.16	0.034	-0.036	0.031
rs150619443	C	G	5.08E-07	0.44	0.088	0.028	0.055
rs1534430	T	C	9.40E-08	-0.09	0.017	-0.017	0.012
rs17490285	G	A	9.26E-07	-0.18	0.038	-0.019	0.019
rs1887414	C	T	1.97E-07	0.12	0.022	-0.039	0.017
rs2111485	G	A	4.19E-06	0.08	0.017	0.007	0.012
rs229526	C	G	1.93E-06	0.10	0.022	-0.005	0.015
rs2476601	G	A	3.49E-45	-0.33	0.023	-0.048	0.020
rs28367132	A	G	1.21E-07	-0.14	0.026	0.009	0.015
rs28450758	A	C	4.17E-06	0.14	0.031	0.028	0.023
rs34536443	C	G	2.47E-07	-0.25	0.049	-0.065	0.031
rs61830417	G	A	3.59E-06	0.27	0.059	-0.002	0.042
rs62076103	G	A	1.23E-07	-0.19	0.035	0.031	0.025

Supplementary Table 2. Details of Hashimoto's Thyroiditis predicting SNPs with inflammatory proteins from IEU Open GWAS (with SNPs reaching $P < 5 \times 10^{-6}$).

Inflammatory proteins						Hashimoto's Thyroiditis	
SNP	Effect allele	Other allele	P value	Beta	Standard Error	log(OR)	Standard Error
rs6448432	A	G	1.75E-06	0.09	0.019	0.006	0.013
rs6501550	T	C	1.42E-06	-0.18	0.037	-0.019	0.035
rs7030280	T	C	6.44E-31	0.21	0.018	-0.009	0.013
rs72808590	C	T	1.55E-06	-0.37	0.077	-0.027	0.045
rs73165154	G	A	3.76E-06	-0.15	0.033	-0.036	0.022
rs73192661	T	C	1.45E-09	-0.10	0.017	-0.010	0.012
rs7335748	G	A	2.41E-06	0.38	0.080	-0.011	0.041
rs766932	T	A	4.96E-07	0.10	0.019	0.001	0.013
rs7767668	T	C	9.60E-07	-0.09	0.019	-0.003	0.012
rs7861040	C	G	9.08E-07	0.08	0.017	0.014	0.012
rs79219789	C	G	2.84E-06	-0.15	0.033	0.008	0.031
rs8084503	T	C	1.97E-07	0.14	0.027	0.017	0.019
rs847803	G	A	7.91E-07	0.09	0.018	0.027	0.013
rs9357119	T	G	3.65E-14	-0.15	0.020	-0.026	0.013
rs9497965	T	C	1.71E-06	0.08	0.017	-0.012	0.012
**CCL20 (F-statistic = 38.11, R2=0.44%)**							
rs10424978	A	C	8.97E-07	-0.08	0.017	-0.007	0.012
rs10494077	A	G	9.20E-10	0.12	0.020	0.000	0.014
rs10786679	A	G	1.87E-06	-0.09	0.018	-0.017	0.014
rs111358861	G	A	3.30E-06	1.82	0.391	0.090	0.107
rs1115587	A	G	1.82E-07	-0.10	0.018	0.007	0.012
rs11211645	G	A	9.33E-08	0.09	0.017	0.000	0.012
rs113075322	G	C	7.18E-07	-0.14	0.028	0.010	0.033
rs11611029	T	C	1.16E-08	-0.10	0.017	0.004	0.012
rs117167029	C	G	8.05E-07	2.99	0.606	-0.152	0.090
rs117369326	A	G	3.55E-06	0.24	0.051	-0.044	0.029
rs118075239	C	T	3.45E-06	0.38	0.083	0.020	0.047
rs11822254	G	A	3.52E-06	-0.08	0.017	0.014	0.012
rs11889341	T	C	6.64E-11	0.12	0.019	0.001	0.014
rs1317983	C	T	1.65E-07	0.09	0.018	0.008	0.013
rs13249705	G	A	3.78E-07	-0.09	0.018	0.018	0.014

Supplementary Table 2. Details of Hashimoto's Thyroiditis predicting SNPs with inflammatory proteins from IEU Open GWAS (with SNPs reaching $P < 5 \times 10^{-6}$).

Inflammatory proteins				Hashimoto's Thyroiditis			
SNP	Effect allele	Other allele	P value	Beta	Standard Error	log(OR)	Standard Error
rs140111921	A	G	1.34E-06	-0.16	0.034	-0.023	0.030
rs150619443	C	G	5.08E-07	0.44	0.088	0.026	0.055
rs1534430	T	C	9.40E-08	-0.09	0.017	0.033	0.012
rs17490285	G	A	9.26E-07	-0.18	0.038	-0.016	0.019
rs1887414	C	T	1.97E-07	0.12	0.022	-0.022	0.017
rs2111485	G	A	4.19E-06	0.08	0.017	-0.003	0.012
rs229526	C	G	1.93E-06	0.10	0.022	0.008	0.015
rs2476601	G	A	3.49E-45	-0.33	0.023	-0.033	0.019
rs28367132	A	G	1.21E-07	-0.14	0.026	-0.017	0.015
rs28450758	A	C	4.17E-06	0.14	0.031	-0.011	0.023
rs3184504	C	T	1.40E-25	-0.18	0.017	-0.002	0.013
rs34536443	C	G	2.47E-07	-0.25	0.049	-0.054	0.030
rs34636506	G	A	1.92E-14	-0.13	0.018	-0.011	0.012
rs61830417	G	A	3.59E-06	0.27	0.059	0.016	0.042
rs62076103	G	A	1.23E-07	-0.19	0.035	-0.004	0.025
rs6448432	A	G	1.75E-06	0.09	0.019	-0.004	0.013
rs6501550	T	C	1.42E-06	-0.18	0.037	0.041	0.034
rs7030280	T	C	6.44E-31	0.21	0.018	-0.019	0.013
rs72808590	C	T	1.55E-06	-0.37	0.077	-0.069	0.045
rs73165154	G	A	3.76E-06	-0.15	0.033	0.057	0.022
rs73192661	T	C	1.45E-09	-0.10	0.017	0.006	0.012
rs7335748	G	A	2.41E-06	0.38	0.080	0.014	0.041
rs766932	T	A	4.96E-07	0.10	0.019	0.002	0.013
rs7767668	T	C	9.60E-07	-0.09	0.019	0.013	0.012
rs7861040	C	G	9.08E-07	0.08	0.017	-0.011	0.012
rs79219789	C	G	2.84E-06	-0.15	0.033	0.080	0.031
rs8084503	T	C	1.97E-07	0.14	0.027	0.015	0.018
rs847803	G	A	7.91E-07	0.09	0.018	-0.004	0.013
rs9271365	G	T	1.44E-39	0.25	0.019	-0.020	0.012
rs9357119	T	G	3.65E-14	-0.15	0.020	0.013	0.013
rs9497965	T	C	1.71E-06	0.08	0.017	-0.003	0.012

Supplementary Table 2. Details of Hashimoto's Thyroiditis predicting SNPs with inflammatory proteins from IEU Open GWAS (with SNPs reaching *P* < 5 x 10^{-6}).

Inflammatory proteins				Hashimoto's Thyroiditis			
SNP	Effect allele	Other allele	P value	Beta	Standard Error	log(OR)	Standard Error
**CCL23 (F-statistic = 38.11, R2=0.44%)**							
rs10424978	A	C	8.97E-07	-0.08	0.017	-0.024	0.012
rs10494077	A	G	9.20E-10	0.12	0.020	-0.006	0.014
rs10786679	A	G	1.87E-06	-0.09	0.018	-0.013	0.013
rs111358861	G	A	3.30E-06	1.82	0.391	-0.037	0.107
rs1115587	A	G	1.82E-07	-0.10	0.018	0.021	0.012
rs11211645	G	A	9.33E-08	0.09	0.017	0.006	0.012
rs113075322	G	C	7.18E-07	-0.14	0.028	-0.018	0.033
rs11611029	T	C	1.16E-08	-0.10	0.017	-0.006	0.012
rs117167029	C	G	8.05E-07	2.99	0.606	0.006	0.089
rs117369326	A	G	3.55E-06	0.24	0.051	-0.010	0.029
rs118075239	C	T	3.45E-06	0.38	0.083	0.060	0.047
rs11822254	G	A	3.52E-06	-0.08	0.017	0.010	0.012
rs11889341	T	C	6.64E-11	0.12	0.019	0.006	0.014
rs1317983	C	T	1.65E-07	0.09	0.018	0.031	0.013
rs13249705	G	A	3.78E-07	-0.09	0.018	-0.001	0.014
rs140111921	A	G	1.34E-06	-0.16	0.034	0.050	0.030
rs150619443	C	G	5.08E-07	0.44	0.088	-0.060	0.054
rs1534430	T	C	9.40E-08	-0.09	0.017	-0.021	0.012
rs17490285	G	A	9.26E-07	-0.18	0.038	0.001	0.019
rs1887414	C	T	1.97E-07	0.12	0.022	-0.002	0.016
rs2111485	G	A	4.19E-06	0.08	0.017	-0.014	0.012
rs229526	C	G	1.93E-06	0.10	0.022	-0.037	0.015
rs2476601	G	A	3.49E-45	-0.33	0.023	-0.004	0.019
rs28367132	A	G	1.21E-07	-0.14	0.026	0.010	0.015
rs28450758	A	C	4.17E-06	0.14	0.031	0.008	0.022
rs3184504	C	T	1.40E-25	-0.18	0.017	-0.026	0.013
rs34536443	C	G	2.47E-07	-0.25	0.049	0.032	0.030
rs34636506	G	A	1.92E-14	-0.13	0.018	-0.010	0.012
rs61830417	G	A	3.59E-06	0.27	0.059	-0.003	0.041
rs62076103	G	A	1.23E-07	-0.19	0.035	-0.019	0.024

Supplementary Table 2. Details of Hashimoto's Thyroiditis predicting SNPs with inflammatory proteins from IEU Open GWAS (with SNPs reaching $P < 5 \times 10^{-6}$).

Inflammatory proteins				Hashimoto's Thyroiditis			
SNP	Effect allele	Other allele	P value	Beta	Standard Error	log(OR)	Standard Error
rs6448432	A	G	1.75E-06	0.09	0.019	0.015	0.013
rs6501550	T	C	1.42E-06	-0.18	0.037	0.021	0.034
rs7030280	T	C	6.44E-31	0.21	0.018	-0.014	0.013
rs72808590	C	T	1.55E-06	-0.37	0.077	-0.052	0.044
rs73165154	G	A	3.76E-06	-0.15	0.033	-0.002	0.022
rs73192661	T	C	1.45E-09	-0.10	0.017	0.015	0.012
rs7335748	G	A	2.41E-06	0.38	0.080	-0.002	0.041
rs766932	T	A	4.96E-07	0.10	0.019	0.010	0.013
rs7767668	T	C	9.60E-07	-0.09	0.019	0.002	0.012
rs7861040	C	G	9.08E-07	0.08	0.017	0.014	0.012
rs79219789	C	G	2.84E-06	-0.15	0.033	-0.018	0.031
rs8084503	T	C	1.97E-07	0.14	0.027	0.000	0.018
rs847803	G	A	7.91E-07	0.09	0.018	-0.019	0.013
rs9271365	G	T	1.44E-39	0.25	0.019	0.019	0.012
rs9357119	T	G	3.65E-14	-0.15	0.020	-0.006	0.013
rs9497965	T	C	1.71E-06	0.08	0.017	0.008	0.012
**CCL25 (F-statistic = 38.11, R2=0.44%)**							
rs10424978	A	C	8.97E-07	-0.08	0.017	-0.011	0.012
rs10494077	A	G	9.20E-10	0.12	0.020	-0.005	0.014
rs10786679	A	G	1.87E-06	-0.09	0.018	-0.009	0.013
rs111358861	G	A	3.30E-06	1.82	0.391	0.129	0.107
rs1115587	A	G	1.82E-07	-0.10	0.018	0.015	0.012
rs11211645	G	A	9.33E-08	0.09	0.017	0.007	0.012
rs113075322	G	C	7.18E-07	-0.14	0.028	-0.056	0.033
rs11611029	T	C	1.16E-08	-0.10	0.017	0.009	0.012
rs117167029	C	G	8.05E-07	2.99	0.606	0.129	0.091
rs117369326	A	G	3.55E-06	0.24	0.051	0.022	0.029
rs118075239	C	T	3.45E-06	0.38	0.083	0.056	0.047
rs11822254	G	A	3.52E-06	-0.08	0.017	0.011	0.012
rs11889341	T	C	6.64E-11	0.12	0.019	0.009	0.014
rs1317983	C	T	1.65E-07	0.09	0.018	0.017	0.013

Supplementary Table 2. Details of Hashimoto's Thyroiditis predicting SNPs with inflammatory proteins from IEU Open GWAS (with SNPs reaching $P < 5 \times 10^{-6}$).

Inflammatory proteins				Hashimoto's Thyroiditis			
SNP	Effect allele	Other allele	P value	Beta	Standard Error	log(OR)	Standard Error
rs13249705	G	A	3.78E-07	-0.09	0.018	-0.016	0.014
rs140111921	A	G	1.34E-06	-0.16	0.034	-0.050	0.030
rs150619443	C	G	5.08E-07	0.44	0.088	0.034	0.054
rs1534430	T	C	9.40E-08	-0.09	0.017	-0.010	0.012
rs17490285	G	A	9.26E-07	-0.18	0.038	-0.013	0.019
rs1887414	C	T	1.97E-07	0.12	0.022	0.020	0.016
rs2111485	G	A	4.19E-06	0.08	0.017	0.013	0.012
rs229526	C	G	1.93E-06	0.10	0.022	-0.001	0.015
rs2476601	G	A	3.49E-45	-0.33	0.023	-0.003	0.019
rs28367132	A	G	1.21E-07	-0.14	0.026	-0.023	0.015
rs28450758	A	C	4.17E-06	0.14	0.031	0.003	0.022
rs3184504	C	T	1.40E-25	-0.18	0.017	-0.013	0.013
rs34536443	C	G	2.47E-07	-0.25	0.049	0.012	0.030
rs34636506	G	A	1.92E-14	-0.13	0.018	-0.025	0.012
rs61830417	G	A	3.59E-06	0.27	0.059	-0.012	0.041
rs62076103	G	A	1.23E-07	-0.19	0.035	-0.013	0.024
rs6448432	A	G	1.75E-06	0.09	0.019	0.000	0.013
rs6501550	T	C	1.42E-06	-0.18	0.037	-0.046	0.034
rs7030280	T	C	6.44E-31	0.21	0.018	-0.016	0.012
rs72808590	C	T	1.55E-06	-0.37	0.077	-0.013	0.044
rs73165154	G	A	3.76E-06	-0.15	0.033	-0.017	0.022
rs73192661	T	C	1.45E-09	-0.10	0.017	-0.002	0.012
rs7335748	G	A	2.41E-06	0.38	0.080	-0.013	0.041
rs766932	T	A	4.96E-07	0.10	0.019	-0.027	0.013
rs7767668	T	C	9.60E-07	-0.09	0.019	-0.027	0.012
rs7861040	C	G	9.08E-07	0.08	0.017	-0.014	0.012
rs79219789	C	G	2.84E-06	-0.15	0.033	0.007	0.031
rs8084503	T	C	1.97E-07	0.14	0.027	0.024	0.018
rs847803	G	A	7.91E-07	0.09	0.018	0.017	0.013
rs9271365	G	T	1.44E-39	0.25	0.019	-0.004	0.012
rs9357119	T	G	3.65E-14	-0.15	0.020	-0.032	0.013

Supplementary Table 2. Details of Hashimoto's Thyroiditis predicting SNPs with inflammatory proteins from IEU Open GWAS (with SNPs reaching $P < 5 \times 10^{-6}$).

Inflammatory proteins				Hashimoto's Thyroiditis			
SNP	Effect allele	Other allele	P value	Beta	Standard Error	log(OR)	Standard Error
rs9497965	T	C	1.71E-06	0.08	0.017	-0.018	0.012
**CCL28 (F-statistic = 38.11, R2=0.44%)**							
rs10424978	A	C	8.97E-07	-0.08	0.017	-0.013	0.012
rs10494077	A	G	9.20E-10	0.12	0.020	0.013	0.013
rs10786679	A	G	1.87E-06	-0.09	0.018	0.010	0.013
rs111358861	G	A	3.30E-06	1.82	0.391	0.005	0.106
rs1115587	A	G	1.82E-07	-0.10	0.018	0.011	0.012
rs11211645	G	A	9.33E-08	0.09	0.017	-0.018	0.011
rs113075322	G	C	7.18E-07	-0.14	0.028	-0.030	0.032
rs11611029	T	C	1.16E-08	-0.10	0.017	-0.002	0.012
rs117167029	C	G	8.05E-07	2.99	0.606	-0.157	0.087
rs117369326	A	G	3.55E-06	0.24	0.051	0.020	0.028
rs118075239	C	T	3.45E-06	0.38	0.083	0.049	0.046
rs11822254	G	A	3.52E-06	-0.08	0.017	0.019	0.012
rs11889341	T	C	6.64E-11	0.12	0.019	-0.014	0.013
rs1317983	C	T	1.65E-07	0.09	0.018	0.031	0.013
rs13249705	G	A	3.78E-07	-0.09	0.018	-0.011	0.014
rs140111921	A	G	1.34E-06	-0.16	0.034	-0.021	0.030
rs150619443	C	G	5.08E-07	0.44	0.088	-0.033	0.053
rs1534430	T	C	9.40E-08	-0.09	0.017	-0.002	0.012
rs17490285	G	A	9.26E-07	-0.18	0.038	0.029	0.018
rs1887414	C	T	1.97E-07	0.12	0.022	-0.029	0.016
rs2111485	G	A	4.19E-06	0.08	0.017	-0.006	0.011
rs229526	C	G	1.93E-06	0.10	0.022	-0.006	0.014
rs2476601	G	A	3.49E-45	-0.33	0.023	0.021	0.018
rs28367132	A	G	1.21E-07	-0.14	0.026	-0.028	0.015
rs28450758	A	C	4.17E-06	0.14	0.031	-0.038	0.022
rs3184504	C	T	1.40E-25	-0.18	0.017	-0.032	0.013
rs34536443	C	G	2.47E-07	-0.25	0.049	-0.031	0.029
rs34636506	G	A	1.92E-14	-0.13	0.018	-0.010	0.012
rs61830417	G	A	3.59E-06	0.27	0.059	-0.011	0.040

Supplementary Table 2. Details of Hashimoto's Thyroiditis predicting SNPs with inflammatory proteins from IEU Open GWAS (with SNPs reaching $P < 5 \times 10^{-6}$).

Inflammatory proteins				Hashimoto's Thyroiditis			
SNP	Effect allele	Other allele	P value	Beta	Standard Error	log(OR)	Standard Error
rs62076103	G	A	1.23E-07	-0.19	0.035	0.000	0.024
rs6448432	A	G	1.75E-06	0.09	0.019	-0.017	0.012
rs6501550	T	C	1.42E-06	-0.18	0.037	0.069	0.033
rs7030280	T	C	6.44E-31	0.21	0.018	-0.008	0.012
rs72808590	C	T	1.55E-06	-0.37	0.077	-0.003	0.043
rs73165154	G	A	3.76E-06	-0.15	0.033	0.004	0.021
rs73192661	T	C	1.45E-09	-0.10	0.017	-0.007	0.012
rs7335748	G	A	2.41E-06	0.38	0.080	-0.003	0.040
rs766932	T	A	4.96E-07	0.10	0.019	-0.003	0.013
rs7767668	T	C	9.60E-07	-0.09	0.019	0.003	0.011
rs7861040	C	G	9.08E-07	0.08	0.017	-0.019	0.012
rs79219789	C	G	2.84E-06	-0.15	0.033	0.017	0.030
rs8084503	T	C	1.97E-07	0.14	0.027	0.001	0.018
rs847803	G	A	7.91E-07	0.09	0.018	-0.007	0.012
rs9271365	G	T	1.44E-39	0.25	0.019	-0.002	0.011
rs9357119	T	G	3.65E-14	-0.15	0.020	0.004	0.013
rs9497965	T	C	1.71E-06	0.08	0.017	-0.010	0.012
**CCL4 (F-statistic = 38.11, R2=0.44%)**							
rs10424978	A	C	8.97E-07	-0.08	0.017	-0.028	0.012
rs10494077	A	G	9.20E-10	0.12	0.020	0.001	0.014
rs10786679	A	G	1.87E-06	-0.09	0.018	-0.014	0.014
rs111358861	G	A	3.30E-06	1.82	0.391	0.070	0.107
rs1115587	A	G	1.82E-07	-0.10	0.018	0.001	0.012
rs11211645	G	A	9.33E-08	0.09	0.017	-0.028	0.012
rs113075322	G	C	7.18E-07	-0.14	0.028	-0.008	0.033
rs11611029	T	C	1.16E-08	-0.10	0.017	0.002	0.012
rs117167029	C	G	8.05E-07	2.99	0.606	0.096	0.089
rs117369326	A	G	3.55E-06	0.24	0.051	0.027	0.029
rs118075239	C	T	3.45E-06	0.38	0.083	0.021	0.047
rs11822254	G	A	3.52E-06	-0.08	0.017	-0.005	0.012
rs11889341	T	C	6.64E-11	0.12	0.019	0.015	0.014

Supplementary Table 2. Details of Hashimoto's Thyroiditis predicting SNPs with inflammatory proteins from IEU Open GWAS (with SNPs reaching $P < 5 \times 10^{-6}$).

Inflammatory proteins				Hashimoto's Thyroiditis			
SNP	Effect allele	Other allele	P value	Beta	Standard Error	log(OR)	Standard Error
rs1317983	C	T	1.65E-07	0.09	0.018	0.001	0.013
rs13249705	G	A	3.78E-07	-0.09	0.018	0.013	0.014
rs140111921	A	G	1.34E-06	-0.16	0.034	0.012	0.031
rs150619443	C	G	5.08E-07	0.44	0.088	0.002	0.055
rs1534430	T	C	9.40E-08	-0.09	0.017	0.010	0.012
rs17490285	G	A	9.26E-07	-0.18	0.038	0.015	0.019
rs1887414	C	T	1.97E-07	0.12	0.022	-0.002	0.017
rs2111485	G	A	4.19E-06	0.08	0.017	-0.026	0.012
rs229526	C	G	1.93E-06	0.10	0.022	-0.008	0.015
rs2476601	G	A	3.49E-45	-0.33	0.023	0.008	0.019
rs28367132	A	G	1.21E-07	-0.14	0.026	-0.020	0.015
rs28450758	A	C	4.17E-06	0.14	0.031	-0.018	0.023
rs3184504	C	T	1.40E-25	-0.18	0.017	-0.038	0.013
rs34536443	C	G	2.47E-07	-0.25	0.049	-0.001	0.031
rs34636506	G	A	1.92E-14	-0.13	0.018	-0.011	0.012
rs61830417	G	A	3.59E-06	0.27	0.059	0.029	0.042
rs62076103	G	A	1.23E-07	-0.19	0.035	0.010	0.025
rs6448432	A	G	1.75E-06	0.09	0.019	0.022	0.013
rs6501550	T	C	1.42E-06	-0.18	0.037	-0.012	0.035
rs7030280	T	C	6.44E-31	0.21	0.018	0.009	0.013
rs72808590	C	T	1.55E-06	-0.37	0.077	-0.074	0.045
rs73165154	G	A	3.76E-06	-0.15	0.033	-0.011	0.022
rs73192661	T	C	1.45E-09	-0.10	0.017	-0.019	0.012
rs7335748	G	A	2.41E-06	0.38	0.080	0.019	0.041
rs766932	T	A	4.96E-07	0.10	0.019	0.017	0.013
rs7767668	T	C	9.60E-07	-0.09	0.019	-0.010	0.012
rs7861040	C	G	9.08E-07	0.08	0.017	-0.010	0.012
rs79219789	C	G	2.84E-06	-0.15	0.033	0.064	0.031
rs8084503	T	C	1.97E-07	0.14	0.027	0.017	0.019
rs847803	G	A	7.91E-07	0.09	0.018	0.010	0.013
rs9271365	G	T	1.44E-39	0.25	0.019	0.010	0.012

Supplementary Table 2. Details of Hashimoto's Thyroiditis predicting SNPs with inflammatory proteins from IEU Open GWAS (with SNPs reaching $P < 5 \times 10^{-6}$).

Inflammatory proteins				Hashimoto's Thyroiditis			
SNP	Effect allele	Other allele	P value	Beta	Standard Error	log(OR)	Standard Error
rs9357119	T	G	3.65E-14	-0.15	0.020	-0.009	0.013
rs9497965	T	C	1.71E-06	0.08	0.017	-0.012	0.012
**CD40LR (F-statistic = 38.11, R2=0.44%)**							
rs10424978	A	C	8.97E-07	-0.08	0.017	-0.017	0.012
rs10494077	A	G	9.20E-10	0.12	0.020	-0.003	0.014
rs10786679	A	G	1.87E-06	-0.09	0.018	0.001	0.013
rs111358861	G	A	3.30E-06	1.82	0.391	-0.233	0.106
rs1115587	A	G	1.82E-07	-0.10	0.018	0.009	0.012
rs11211645	G	A	9.33E-08	0.09	0.017	-0.029	0.012
rs113075322	G	C	7.18E-07	-0.14	0.028	-0.012	0.033
rs11611029	T	C	1.16E-08	-0.10	0.017	0.019	0.012
rs117167029	C	G	8.05E-07	2.99	0.606	-0.002	0.088
rs117369326	A	G	3.55E-06	0.24	0.051	0.004	0.029
rs118075239	C	T	3.45E-06	0.38	0.083	0.033	0.046
rs11822254	G	A	3.52E-06	-0.08	0.017	-0.016	0.012
rs11889341	T	C	6.64E-11	0.12	0.019	-0.006	0.013
rs1317983	C	T	1.65E-07	0.09	0.018	-0.011	0.013
rs13249705	G	A	3.78E-07	-0.09	0.018	-0.023	0.014
rs140111921	A	G	1.34E-06	-0.16	0.034	0.011	0.030
rs150619443	C	G	5.08E-07	0.44	0.088	-0.035	0.053
rs1534430	T	C	9.40E-08	-0.09	0.017	-0.003	0.012
rs17490285	G	A	9.26E-07	-0.18	0.038	0.004	0.019
rs1887414	C	T	1.97E-07	0.12	0.022	-0.016	0.016
rs2111485	G	A	4.19E-06	0.08	0.017	-0.001	0.011
rs229526	C	G	1.93E-06	0.10	0.022	0.004	0.014
rs2476601	G	A	3.49E-45	-0.33	0.023	0.023	0.018
rs28367132	A	G	1.21E-07	-0.14	0.026	0.012	0.015
rs28450758	A	C	4.17E-06	0.14	0.031	-0.004	0.022
rs3184504	C	T	1.40E-25	-0.18	0.017	-0.031	0.013
rs34536443	C	G	2.47E-07	-0.25	0.049	0.009	0.030
rs34636506	G	A	1.92E-14	-0.13	0.018	0.001	0.012

Supplementary Table 2. Details of Hashimoto's Thyroiditis predicting SNPs with inflammatory proteins from IEU Open GWAS (with SNPs reaching $P < 5 \times 10^{-6}$).

Inflammatory proteins				Hashimoto's Thyroiditis			
SNP	Effect allele	Other allele	P value	Beta	Standard Error	log(OR)	Standard Error
rs61830417	G	A	3.59E-06	0.27	0.059	-0.006	0.041
rs62076103	G	A	1.23E-07	-0.19	0.035	0.015	0.024
rs6448432	A	G	1.75E-06	0.09	0.019	0.004	0.012
rs6501550	T	C	1.42E-06	-0.18	0.037	0.047	0.034
rs7030280	T	C	6.44E-31	0.21	0.018	-0.007	0.012
rs72808590	C	T	1.55E-06	-0.37	0.077	-0.028	0.043
rs73165154	G	A	3.76E-06	-0.15	0.033	-0.018	0.022
rs73192661	T	C	1.45E-09	-0.10	0.017	-0.012	0.012
rs7335748	G	A	2.41E-06	0.38	0.080	-0.006	0.040
rs766932	T	A	4.96E-07	0.10	0.019	-0.015	0.013
rs7767668	T	C	9.60E-07	-0.09	0.019	-0.006	0.012
rs7861040	C	G	9.08E-07	0.08	0.017	0.021	0.012
rs79219789	C	G	2.84E-06	-0.15	0.033	-0.010	0.030
rs8084503	T	C	1.97E-07	0.14	0.027	0.016	0.018
rs847803	G	A	7.91E-07	0.09	0.018	0.025	0.013
rs9271365	G	T	1.44E-39	0.25	0.019	0.015	0.011
rs9357119	T	G	3.65E-14	-0.15	0.020	-0.005	0.013
rs9497965	T	C	1.71E-06	0.08	0.017	-0.014	0.012
**CD5 (F-statistic = 36.02, R2=0.40%)**							
rs10424978	A	C	8.97E-07	-0.08	0.017	-0.027	0.012
rs10494077	A	G	9.20E-10	0.12	0.020	0.006	0.014
rs10786679	A	G	1.87E-06	-0.09	0.018	0.009	0.013
rs111358861	G	A	3.30E-06	1.82	0.391	-0.170	0.106
rs1115587	A	G	1.82E-07	-0.10	0.018	0.003	0.012
rs11211645	G	A	9.33E-08	0.09	0.017	-0.008	0.012
rs113075322	G	C	7.18E-07	-0.14	0.028	-0.004	0.033
rs11611029	T	C	1.16E-08	-0.10	0.017	0.010	0.012
rs117167029	C	G	8.05E-07	2.99	0.606	-0.104	0.088
rs117369326	A	G	3.55E-06	0.24	0.051	-0.001	0.029
rs118075239	C	T	3.45E-06	0.38	0.083	0.090	0.047
rs11822254	G	A	3.52E-06	-0.08	0.017	0.008	0.012

Supplementary Table 2. Details of Hashimoto's Thyroiditis predicting SNPs with inflammatory proteins from IEU Open GWAS (with SNPs reaching $P < 5 \times 10^{-6}$).

Inflammatory proteins				Hashimoto's Thyroiditis			
SNP	Effect allele	Other allele	P value	Beta	Standard Error	log(OR)	Standard Error
rs11889341	T	C	6.64E-11	0.12	0.019	-0.003	0.014
rs1317983	C	T	1.65E-07	0.09	0.018	0.015	0.013
rs13249705	G	A	3.78E-07	-0.09	0.018	-0.020	0.014
rs140111921	A	G	1.34E-06	-0.16	0.034	0.030	0.031
rs150619443	C	G	5.08E-07	0.44	0.088	-0.075	0.054
rs1534430	T	C	9.40E-08	-0.09	0.017	-0.008	0.012
rs17490285	G	A	9.26E-07	-0.18	0.038	0.006	0.019
rs1887414	C	T	1.97E-07	0.12	0.022	-0.016	0.016
rs2111485	G	A	4.19E-06	0.08	0.017	-0.019	0.012
rs229526	C	G	1.93E-06	0.10	0.022	-0.005	0.015
rs2476601	G	A	3.49E-45	-0.33	0.023	-0.050	0.019
rs28367132	A	G	1.21E-07	-0.14	0.026	0.011	0.015
rs28450758	A	C	4.17E-06	0.14	0.031	-0.007	0.022
rs34536443	C	G	2.47E-07	-0.25	0.049	0.005	0.030
rs61830417	G	A	3.59E-06	0.27	0.059	0.017	0.041
rs62076103	G	A	1.23E-07	-0.19	0.035	-0.002	0.024
rs6448432	A	G	1.75E-06	0.09	0.019	0.012	0.013
rs6501550	T	C	1.42E-06	-0.18	0.037	0.022	0.035
rs7030280	T	C	6.44E-31	0.21	0.018	0.000	0.013
rs72808590	C	T	1.55E-06	-0.37	0.077	-0.083	0.044
rs73165154	G	A	3.76E-06	-0.15	0.033	0.011	0.022
rs73192661	T	C	1.45E-09	-0.10	0.017	0.003	0.012
rs7335748	G	A	2.41E-06	0.38	0.080	0.012	0.040
rs766932	T	A	4.96E-07	0.10	0.019	0.007	0.013
rs7767668	T	C	9.60E-07	-0.09	0.019	-0.016	0.012
rs7861040	C	G	9.08E-07	0.08	0.017	0.007	0.012
rs79219789	C	G	2.84E-06	-0.15	0.033	0.005	0.030
rs8084503	T	C	1.97E-07	0.14	0.027	0.028	0.018
rs847803	G	A	7.91E-07	0.09	0.018	0.009	0.013
rs9271365	G	T	1.44E-39	0.25	0.019	-0.037	0.012
rs9357119	T	G	3.65E-14	-0.15	0.020	-0.037	0.013

Supplementary Table 2. Details of Hashimoto's Thyroiditis predicting SNPs with inflammatory proteins from IEU Open GWAS (with SNPs reaching $P < 5 \times 10^{-6}$).

Inflammatory proteins				Hashimoto's Thyroiditis			
SNP	Effect allele	Other allele	P value	Beta	Standard Error	log(OR)	Standard Error
rs9497965	T	C	1.71E-06	0.08	0.017	-0.008	0.012
**CD6 (F-statistic = 36.52, R2=0.42%)**							
rs10424978	A	C	8.97E-07	-0.08	0.017	-0.015	0.012
rs10494077	A	G	9.20E-10	0.12	0.020	0.017	0.014
rs10786679	A	G	1.87E-06	-0.09	0.018	-0.008	0.013
rs111358861	G	A	3.30E-06	1.82	0.391	-0.191	0.107
rs1115587	A	G	1.82E-07	-0.10	0.018	0.013	0.012
rs11211645	G	A	9.33E-08	0.09	0.017	-0.010	0.012
rs113075322	G	C	7.18E-07	-0.14	0.028	0.006	0.033
rs11611029	T	C	1.16E-08	-0.10	0.017	-0.002	0.012
rs117167029	C	G	8.05E-07	2.99	0.606	-0.106	0.088
rs117369326	A	G	3.55E-06	0.24	0.051	0.017	0.029
rs118075239	C	T	3.45E-06	0.38	0.083	0.052	0.047
rs11822254	G	A	3.52E-06	-0.08	0.017	0.013	0.012
rs11889341	T	C	6.64E-11	0.12	0.019	-0.006	0.014
rs1317983	C	T	1.65E-07	0.09	0.018	0.002	0.013
rs13249705	G	A	3.78E-07	-0.09	0.018	-0.017	0.014
rs140111921	A	G	1.34E-06	-0.16	0.034	0.007	0.031
rs150619443	C	G	5.08E-07	0.44	0.088	-0.036	0.054
rs1534430	T	C	9.40E-08	-0.09	0.017	-0.007	0.012
rs17490285	G	A	9.26E-07	-0.18	0.038	0.017	0.019
rs1887414	C	T	1.97E-07	0.12	0.022	-0.022	0.017
rs2111485	G	A	4.19E-06	0.08	0.017	-0.025	0.012
rs229526	C	G	1.93E-06	0.10	0.022	0.033	0.015
rs2476601	G	A	3.49E-45	-0.33	0.023	0.015	0.019
rs28367132	A	G	1.21E-07	-0.14	0.026	-0.019	0.015
rs28450758	A	C	4.17E-06	0.14	0.031	-0.008	0.022
rs34536443	C	G	2.47E-07	-0.25	0.049	-0.057	0.030
rs34636506	G	A	1.92E-14	-0.13	0.018	-0.038	0.012
rs61830417	G	A	3.59E-06	0.27	0.059	0.002	0.042
rs62076103	G	A	1.23E-07	-0.19	0.035	0.015	0.024

Supplementary Table 2. Details of Hashimoto's Thyroiditis predicting SNPs with inflammatory proteins from IEU Open GWAS (with SNPs reaching $P < 5 \times 10^{-6}$).

Inflammatory proteins						Hashimoto's Thyroiditis	
SNP	Effect allele	Other allele	P value	Beta	Standard Error	log(OR)	Standard Error
rs6448432	A	G	1.75E-06	0.09	0.019	0.010	0.013
rs6501550	T	C	1.42E-06	-0.18	0.037	-0.013	0.034
rs7030280	T	C	6.44E-31	0.21	0.018	-0.012	0.013
rs72808590	C	T	1.55E-06	-0.37	0.077	-0.052	0.044
rs73165154	G	A	3.76E-06	-0.15	0.033	0.030	0.022
rs73192661	T	C	1.45E-09	-0.10	0.017	-0.005	0.012
rs7335748	G	A	2.41E-06	0.38	0.080	0.054	0.041
rs766932	T	A	4.96E-07	0.10	0.019	0.018	0.013
rs7767668	T	C	9.60E-07	-0.09	0.019	-0.017	0.012
rs7861040	C	G	9.08E-07	0.08	0.017	-0.019	0.012
rs79219789	C	G	2.84E-06	-0.15	0.033	0.047	0.031
rs8084503	T	C	1.97E-07	0.14	0.027	0.025	0.018
rs847803	G	A	7.91E-07	0.09	0.018	-0.005	0.013
rs9271365	G	T	1.44E-39	0.25	0.019	-0.003	0.012
rs9357119	T	G	3.65E-14	-0.15	0.020	-0.023	0.013
rs9497965	T	C	1.71E-06	0.08	0.017	-0.020	0.012
**CDCP1 (F-statistic = 36.52, R2=0.42%)**							
rs10424978	A	C	8.97E-07	-0.08	0.017	-0.012	0.012
rs10494077	A	G	9.20E-10	0.12	0.020	0.000	0.014
rs10786679	A	G	1.87E-06	-0.09	0.018	-0.007	0.013
rs111358861	G	A	3.30E-06	1.82	0.391	-0.020	0.106
rs1115587	A	G	1.82E-07	-0.10	0.018	0.005	0.012
rs11211645	G	A	9.33E-08	0.09	0.017	-0.015	0.012
rs113075322	G	C	7.18E-07	-0.14	0.028	0.055	0.032
rs11611029	T	C	1.16E-08	-0.10	0.017	-0.005	0.012
rs117167029	C	G	8.05E-07	2.99	0.606	-0.075	0.087
rs117369326	A	G	3.55E-06	0.24	0.051	-0.028	0.028
rs118075239	C	T	3.45E-06	0.38	0.083	0.082	0.045
rs11822254	G	A	3.52E-06	-0.08	0.017	0.006	0.012
rs11889341	T	C	6.64E-11	0.12	0.019	0.021	0.013
rs1317983	C	T	1.65E-07	0.09	0.018	0.023	0.013

Supplementary Table 2. Details of Hashimoto's Thyroiditis predicting SNPs with inflammatory proteins from IEU Open GWAS (with SNPs reaching *P* < 5 x 10^{-6}).

Inflammatory proteins				Hashimoto's Thyroiditis			
SNP	Effect allele	Other allele	P value	Beta	Standard Error	log(OR)	Standard Error
rs13249705	G	A	3.78E-07	-0.09	0.018	0.002	0.014
rs140111921	A	G	1.34E-06	-0.16	0.034	-0.025	0.029
rs150619443	C	G	5.08E-07	0.44	0.088	-0.024	0.053
rs1534430	T	C	9.40E-08	-0.09	0.017	0.001	0.012
rs17490285	G	A	9.26E-07	-0.18	0.038	-0.007	0.018
rs1887414	C	T	1.97E-07	0.12	0.022	0.011	0.016
rs2111485	G	A	4.19E-06	0.08	0.017	0.023	0.011
rs229526	C	G	1.93E-06	0.10	0.022	0.017	0.014
rs2476601	G	A	3.49E-45	-0.33	0.023	0.034	0.019
rs28367132	A	G	1.21E-07	-0.14	0.026	0.015	0.015
rs28450758	A	C	4.17E-06	0.14	0.031	-0.030	0.022
rs34536443	C	G	2.47E-07	-0.25	0.049	-0.053	0.030
rs34636506	G	A	1.92E-14	-0.13	0.018	-0.009	0.012
rs61830417	G	A	3.59E-06	0.27	0.059	0.123	0.040
rs62076103	G	A	1.23E-07	-0.19	0.035	-0.045	0.024
rs6448432	A	G	1.75E-06	0.09	0.019	0.004	0.013
rs6501550	T	C	1.42E-06	-0.18	0.037	0.054	0.033
rs7030280	T	C	6.44E-31	0.21	0.018	0.000	0.012
rs72808590	C	T	1.55E-06	-0.37	0.077	-0.081	0.043
rs73165154	G	A	3.76E-06	-0.15	0.033	0.030	0.021
rs73192661	T	C	1.45E-09	-0.10	0.017	0.011	0.012
rs7335748	G	A	2.41E-06	0.38	0.080	0.008	0.040
rs766932	T	A	4.96E-07	0.10	0.019	-0.027	0.013
rs7767668	T	C	9.60E-07	-0.09	0.019	0.007	0.012
rs7861040	C	G	9.08E-07	0.08	0.017	-0.020	0.012
rs79219789	C	G	2.84E-06	-0.15	0.033	0.080	0.030
rs8084503	T	C	1.97E-07	0.14	0.027	0.025	0.018
rs847803	G	A	7.91E-07	0.09	0.018	-0.011	0.012
rs9271365	G	T	1.44E-39	0.25	0.019	-0.008	0.011
rs9357119	T	G	3.65E-14	-0.15	0.020	-0.007	0.013
rs9497965	T	C	1.71E-06	0.08	0.017	-0.002	0.012

Supplementary Table 2. Details of Hashimoto's Thyroiditis predicting SNPs with inflammatory proteins from IEU Open GWAS (with SNPs reaching $P < 5 \times 10^{-6}$).

Inflammatory proteins				Hashimoto's Thyroiditis			
SNP	Effect allele	Other allele	P value	Beta	Standard Error	log(OR)	Standard Error
**CST5 (F-statistic = 38.11, R2=0.44%)**							
rs10424978	A	C	8.97E-07	-0.08	0.017	-0.026	0.012
rs10494077	A	G	9.20E-10	0.12	0.020	-0.002	0.014
rs10786679	A	G	1.87E-06	-0.09	0.018	0.009	0.013
rs111358861	G	A	3.30E-06	1.82	0.391	0.041	0.107
rs1115587	A	G	1.82E-07	-0.10	0.018	-0.005	0.012
rs11211645	G	A	9.33E-08	0.09	0.017	0.005	0.012
rs113075322	G	C	7.18E-07	-0.14	0.028	0.004	0.033
rs11611029	T	C	1.16E-08	-0.10	0.017	-0.003	0.012
rs117167029	C	G	8.05E-07	2.99	0.606	-0.087	0.088
rs117369326	A	G	3.55E-06	0.24	0.051	0.011	0.029
rs118075239	C	T	3.45E-06	0.38	0.083	0.038	0.047
rs11822254	G	A	3.52E-06	-0.08	0.017	-0.012	0.012
rs11889341	T	C	6.64E-11	0.12	0.019	-0.005	0.014
rs1317983	C	T	1.65E-07	0.09	0.018	0.018	0.013
rs13249705	G	A	3.78E-07	-0.09	0.018	-0.011	0.014
rs140111921	A	G	1.34E-06	-0.16	0.034	-0.034	0.030
rs150619443	C	G	5.08E-07	0.44	0.088	-0.045	0.054
rs1534430	T	C	9.40E-08	-0.09	0.017	-0.019	0.012
rs17490285	G	A	9.26E-07	-0.18	0.038	0.033	0.019
rs1887414	C	T	1.97E-07	0.12	0.022	-0.016	0.016
rs2111485	G	A	4.19E-06	0.08	0.017	0.010	0.012
rs229526	C	G	1.93E-06	0.10	0.022	0.017	0.014
rs2476601	G	A	3.49E-45	-0.33	0.023	-0.008	0.019
rs28367132	A	G	1.21E-07	-0.14	0.026	-0.007	0.015
rs28450758	A	C	4.17E-06	0.14	0.031	-0.008	0.022
rs3184504	C	T	1.40E-25	-0.18	0.017	-0.010	0.013
rs34536443	C	G	2.47E-07	-0.25	0.049	0.016	0.030
rs34636506	G	A	1.92E-14	-0.13	0.018	-0.013	0.012
rs61830417	G	A	3.59E-06	0.27	0.059	0.043	0.041
rs62076103	G	A	1.23E-07	-0.19	0.035	0.003	0.024

Supplementary Table 2. Details of Hashimoto's Thyroiditis predicting SNPs with inflammatory proteins from IEU Open GWAS (with SNPs reaching $P < 5 \times 10^{-6}$).

Inflammatory proteins				Hashimoto's Thyroiditis			
SNP	Effect allele	Other allele	P value	Beta	Standard Error	log(OR)	Standard Error
rs6448432	A	G	1.75E-06	0.09	0.019	-0.010	0.013
rs6501550	T	C	1.42E-06	-0.18	0.037	0.081	0.034
rs7030280	T	C	6.44E-31	0.21	0.018	-0.002	0.012
rs72808590	C	T	1.55E-06	-0.37	0.077	-0.011	0.044
rs73165154	G	A	3.76E-06	-0.15	0.033	0.007	0.022
rs73192661	T	C	1.45E-09	-0.10	0.017	0.007	0.012
rs7335748	G	A	2.41E-06	0.38	0.080	0.025	0.040
rs766932	T	A	4.96E-07	0.10	0.019	-0.001	0.013
rs7767668	T	C	9.60E-07	-0.09	0.019	-0.003	0.012
rs7861040	C	G	9.08E-07	0.08	0.017	-0.006	0.012
rs79219789	C	G	2.84E-06	-0.15	0.033	-0.077	0.030
rs8084503	T	C	1.97E-07	0.14	0.027	-0.016	0.018
rs847803	G	A	7.91E-07	0.09	0.018	0.009	0.013
rs9271365	G	T	1.44E-39	0.25	0.019	-0.007	0.012
rs9357119	T	G	3.65E-14	-0.15	0.020	-0.013	0.013
rs9497965	T	C	1.71E-06	0.08	0.017	-0.008	0.012
**CX3CL1 (F-statistic = 37.68, R2=0.43%)**							
rs10424978	A	C	8.97E-07	-0.08	0.017	-0.020	0.012
rs10494077	A	G	9.20E-10	0.12	0.020	-0.007	0.014
rs10786679	A	G	1.87E-06	-0.09	0.018	-0.009	0.013
rs111358861	G	A	3.30E-06	1.82	0.391	-0.001	0.107
rs1115587	A	G	1.82E-07	-0.10	0.018	-0.010	0.012
rs11211645	G	A	9.33E-08	0.09	0.017	0.002	0.012
rs113075322	G	C	7.18E-07	-0.14	0.028	-0.006	0.033
rs11611029	T	C	1.16E-08	-0.10	0.017	-0.015	0.012
rs117167029	C	G	8.05E-07	2.99	0.606	0.042	0.090
rs117369326	A	G	3.55E-06	0.24	0.051	-0.012	0.029
rs118075239	C	T	3.45E-06	0.38	0.083	0.059	0.047
rs11822254	G	A	3.52E-06	-0.08	0.017	0.010	0.012
rs11889341	T	C	6.64E-11	0.12	0.019	-0.009	0.014
rs1317983	C	T	1.65E-07	0.09	0.018	0.022	0.013

Supplementary Table 2. Details of Hashimoto's Thyroiditis predicting SNPs with inflammatory proteins from IEU Open GWAS (with SNPs reaching *P* < 5 x 10^{-6}).

Inflammatory proteins				Hashimoto's Thyroiditis			
SNP	Effect allele	Other allele	P value	Beta	Standard Error	log(OR)	Standard Error
rs13249705	G	A	3.78E-07	-0.09	0.018	0.004	0.014
rs140111921	A	G	1.34E-06	-0.16	0.034	-0.019	0.030
rs150619443	C	G	5.08E-07	0.44	0.088	-0.044	0.054
rs1534430	T	C	9.40E-08	-0.09	0.017	-0.016	0.012
rs17490285	G	A	9.26E-07	-0.18	0.038	0.024	0.019
rs1887414	C	T	1.97E-07	0.12	0.022	-0.021	0.016
rs2111485	G	A	4.19E-06	0.08	0.017	0.004	0.012
rs229526	C	G	1.93E-06	0.10	0.022	-0.016	0.015
rs2476601	G	A	3.49E-45	-0.33	0.023	0.007	0.019
rs28367132	A	G	1.21E-07	-0.14	0.026	0.010	0.015
rs28450758	A	C	4.17E-06	0.14	0.031	0.000	0.022
rs3184504	C	T	1.40E-25	-0.18	0.017	-0.005	0.013
rs34536443	C	G	2.47E-07	-0.25	0.049	-0.007	0.030
rs34636506	G	A	1.92E-14	-0.13	0.018	0.008	0.012
rs61830417	G	A	3.59E-06	0.27	0.059	-0.011	0.041
rs62076103	G	A	1.23E-07	-0.19	0.035	-0.023	0.024
rs6448432	A	G	1.75E-06	0.09	0.019	-0.007	0.013
rs6501550	T	C	1.42E-06	-0.18	0.037	0.019	0.034
rs7030280	T	C	6.44E-31	0.21	0.018	-0.006	0.012
rs72808590	C	T	1.55E-06	-0.37	0.077	-0.017	0.044
rs73165154	G	A	3.76E-06	-0.15	0.033	-0.004	0.022
rs73192661	T	C	1.45E-09	-0.10	0.017	-0.004	0.012
rs7335748	G	A	2.41E-06	0.38	0.080	-0.046	0.040
rs766932	T	A	4.96E-07	0.10	0.019	0.005	0.013
rs7767668	T	C	9.60E-07	-0.09	0.019	-0.009	0.012
rs7861040	C	G	9.08E-07	0.08	0.017	0.014	0.012
rs79219789	C	G	2.84E-06	-0.15	0.033	-0.009	0.030
rs8084503	T	C	1.97E-07	0.14	0.027	0.018	0.018
rs847803	G	A	7.91E-07	0.09	0.018	0.009	0.013
rs9271365	G	T	1.44E-39	0.25	0.019	0.002	0.012
rs9497965	T	C	1.71E-06	0.08	0.017	0.002	0.012

Supplementary Table 2. Details of Hashimoto's Thyroiditis predicting SNPs with inflammatory proteins from IEU Open GWAS (with SNPs reaching $P < 5 \times 10^{-6}$).

Inflammatory proteins				Hashimoto's Thyroiditis			
SNP	Effect allele	Other allele	P value	Beta	Standard Error	log(OR)	Standard Error
**CXCL1 (F-statistic = 38.11, R2=0.44%)**							
rs10424978	A	C	8.97E-07	-0.08	0.017	-0.009	0.012
rs10494077	A	G	9.20E-10	0.12	0.020	-0.010	0.014
rs10786679	A	G	1.87E-06	-0.09	0.018	-0.002	0.014
rs111358861	G	A	3.30E-06	1.82	0.391	-0.145	0.106
rs1115587	A	G	1.82E-07	-0.10	0.018	0.021	0.012
rs11211645	G	A	9.33E-08	0.09	0.017	-0.030	0.012
rs113075322	G	C	7.18E-07	-0.14	0.028	-0.022	0.033
rs11611029	T	C	1.16E-08	-0.10	0.017	0.011	0.012
rs117167029	C	G	8.05E-07	2.99	0.606	-0.115	0.088
rs117369326	A	G	3.55E-06	0.24	0.051	-0.014	0.029
rs118075239	C	T	3.45E-06	0.38	0.083	-0.071	0.047
rs11822254	G	A	3.52E-06	-0.08	0.017	0.002	0.012
rs11889341	T	C	6.64E-11	0.12	0.019	0.003	0.014
rs1317983	C	T	1.65E-07	0.09	0.018	0.010	0.013
rs13249705	G	A	3.78E-07	-0.09	0.018	-0.003	0.014
rs140111921	A	G	1.34E-06	-0.16	0.034	0.014	0.031
rs150619443	C	G	5.08E-07	0.44	0.088	0.012	0.054
rs1534430	T	C	9.40E-08	-0.09	0.017	0.023	0.012
rs17490285	G	A	9.26E-07	-0.18	0.038	-0.009	0.019
rs1887414	C	T	1.97E-07	0.12	0.022	-0.004	0.017
rs2111485	G	A	4.19E-06	0.08	0.017	-0.002	0.012
rs229526	C	G	1.93E-06	0.10	0.022	-0.011	0.015
rs2476601	G	A	3.49E-45	-0.33	0.023	0.014	0.019
rs28367132	A	G	1.21E-07	-0.14	0.026	-0.020	0.015
rs28450758	A	C	4.17E-06	0.14	0.031	0.023	0.023
rs3184504	C	T	1.40E-25	-0.18	0.017	-0.021	0.013
rs34536443	C	G	2.47E-07	-0.25	0.049	0.020	0.030
rs34636506	G	A	1.92E-14	-0.13	0.018	0.009	0.012
rs61830417	G	A	3.59E-06	0.27	0.059	-0.056	0.041
rs62076103	G	A	1.23E-07	-0.19	0.035	-0.015	0.025

Supplementary Table 2. Details of Hashimoto's Thyroiditis predicting SNPs with inflammatory proteins from IEU Open GWAS (with SNPs reaching $P < 5 \times 10^{-6}$).

Inflammatory proteins				Hashimoto's Thyroiditis			
SNP	Effect allele	Other allele	P value	Beta	Standard Error	log(OR)	Standard Error
rs6448432	A	G	1.75E-06	0.09	0.019	0.026	0.013
rs6501550	T	C	1.42E-06	-0.18	0.037	0.001	0.035
rs7030280	T	C	6.44E-31	0.21	0.018	-0.004	0.013
rs72808590	C	T	1.55E-06	-0.37	0.077	-0.045	0.045
rs73165154	G	A	3.76E-06	-0.15	0.033	0.008	0.022
rs73192661	T	C	1.45E-09	-0.10	0.017	-0.020	0.012
rs7335748	G	A	2.41E-06	0.38	0.080	0.029	0.041
rs766932	T	A	4.96E-07	0.10	0.019	-0.020	0.013
rs7767668	T	C	9.60E-07	-0.09	0.019	-0.023	0.012
rs7861040	C	G	9.08E-07	0.08	0.017	-0.008	0.012
rs79219789	C	G	2.84E-06	-0.15	0.033	-0.022	0.031
rs8084503	T	C	1.97E-07	0.14	0.027	-0.011	0.018
rs847803	G	A	7.91E-07	0.09	0.018	0.008	0.013
rs9271365	G	T	1.44E-39	0.25	0.019	0.021	0.012
rs9357119	T	G	3.65E-14	-0.15	0.020	0.013	0.013
rs9497965	T	C	1.71E-06	0.08	0.017	-0.004	0.012
**CXCL10 (F-statistic = 36.74, R2=0.41%)**							
rs10424978	A	C	8.97E-07	-0.08	0.017	-0.019	0.012
rs10494077	A	G	9.20E-10	0.12	0.020	-0.007	0.014
rs10786679	A	G	1.87E-06	-0.09	0.018	-0.021	0.013
rs111358861	G	A	3.30E-06	1.82	0.391	0.057	0.106
rs1115587	A	G	1.82E-07	-0.10	0.018	0.016	0.012
rs11211645	G	A	9.33E-08	0.09	0.017	-0.027	0.012
rs113075322	G	C	7.18E-07	-0.14	0.028	-0.009	0.033
rs11611029	T	C	1.16E-08	-0.10	0.017	-0.003	0.012
rs117167029	C	G	8.05E-07	2.99	0.606	0.051	0.088
rs117369326	A	G	3.55E-06	0.24	0.051	0.022	0.029
rs118075239	C	T	3.45E-06	0.38	0.083	-0.001	0.047
rs11822254	G	A	3.52E-06	-0.08	0.017	0.009	0.012
rs11889341	T	C	6.64E-11	0.12	0.019	0.028	0.014
rs1317983	C	T	1.65E-07	0.09	0.018	0.027	0.013

Supplementary Table 2. Details of Hashimoto's Thyroiditis predicting SNPs with inflammatory proteins from IEU Open GWAS (with SNPs reaching $P < 5 \times 10^{-6}$).

Inflammatory proteins				Hashimoto's Thyroiditis			
SNP	Effect allele	Other allele	P value	Beta	Standard Error	log(OR)	Standard Error
rs13249705	G	A	3.78E-07	-0.09	0.018	-0.006	0.014
rs140111921	A	G	1.34E-06	-0.16	0.034	-0.004	0.030
rs150619443	C	G	5.08E-07	0.44	0.088	-0.017	0.055
rs1534430	T	C	9.40E-08	-0.09	0.017	-0.002	0.012
rs17490285	G	A	9.26E-07	-0.18	0.038	-0.030	0.019
rs1887414	C	T	1.97E-07	0.12	0.022	-0.017	0.017
rs2111485	G	A	4.19E-06	0.08	0.017	0.031	0.012
rs229526	C	G	1.93E-06	0.10	0.022	-0.013	0.015
rs2476601	G	A	3.49E-45	-0.33	0.023	0.018	0.019
rs28367132	A	G	1.21E-07	-0.14	0.026	-0.011	0.015
rs28450758	A	C	4.17E-06	0.14	0.031	0.001	0.023
rs34636506	G	A	1.92E-14	-0.13	0.018	-0.025	0.012
rs61830417	G	A	3.59E-06	0.27	0.059	0.012	0.042
rs62076103	G	A	1.23E-07	-0.19	0.035	-0.024	0.025
rs6448432	A	G	1.75E-06	0.09	0.019	0.021	0.013
rs6501550	T	C	1.42E-06	-0.18	0.037	0.053	0.034
rs7030280	T	C	6.44E-31	0.21	0.018	0.001	0.013
rs72808590	C	T	1.55E-06	-0.37	0.077	-0.038	0.045
rs73165154	G	A	3.76E-06	-0.15	0.033	-0.014	0.022
rs73192661	T	C	1.45E-09	-0.10	0.017	0.023	0.012
rs7335748	G	A	2.41E-06	0.38	0.080	0.009	0.041
rs766932	T	A	4.96E-07	0.10	0.019	-0.023	0.013
rs7767668	T	C	9.60E-07	-0.09	0.019	-0.018	0.012
rs7861040	C	G	9.08E-07	0.08	0.017	-0.019	0.012
rs79219789	C	G	2.84E-06	-0.15	0.033	0.025	0.031
rs8084503	T	C	1.97E-07	0.14	0.027	-0.017	0.018
rs847803	G	A	7.91E-07	0.09	0.018	-0.010	0.013
rs9271365	G	T	1.44E-39	0.25	0.019	-0.006	0.012
rs9357119	T	G	3.65E-14	-0.15	0.020	-0.014	0.013
rs9497965	T	C	1.71E-06	0.08	0.017	-0.006	0.012
**CXCL11 (F-statistic = 36.52, R2=0.42%)**							

Supplementary Table 2. Details of Hashimoto's Thyroiditis predicting SNPs with inflammatory proteins from IEU Open GWAS (with SNPs reaching $P < 5 \times 10^{-6}$).

Inflammatory proteins				Hashimoto's Thyroiditis			
SNP	Effect allele	Other allele	P value	Beta	Standard Error	log(OR)	Standard Error
rs10424978	A	C	8.97E-07	-0.08	0.017	-0.024	0.012
rs10494077	A	G	9.20E-10	0.12	0.020	0.001	0.014
rs10786679	A	G	1.87E-06	-0.09	0.018	0.017	0.013
rs111358861	G	A	3.30E-06	1.82	0.391	-0.161	0.107
rs1115587	A	G	1.82E-07	-0.10	0.018	0.017	0.012
rs11211645	G	A	9.33E-08	0.09	0.017	-0.039	0.012
rs113075322	G	C	7.18E-07	-0.14	0.028	-0.009	0.033
rs11611029	T	C	1.16E-08	-0.10	0.017	-0.003	0.012
rs117167029	C	G	8.05E-07	2.99	0.606	-0.044	0.089
rs117369326	A	G	3.55E-06	0.24	0.051	0.006	0.029
rs118075239	C	T	3.45E-06	0.38	0.083	-0.014	0.048
rs11822254	G	A	3.52E-06	-0.08	0.017	-0.001	0.012
rs11889341	T	C	6.64E-11	0.12	0.019	0.006	0.014
rs1317983	C	T	1.65E-07	0.09	0.018	-0.003	0.013
rs13249705	G	A	3.78E-07	-0.09	0.018	-0.015	0.014
rs140111921	A	G	1.34E-06	-0.16	0.034	0.040	0.031
rs150619443	C	G	5.08E-07	0.44	0.088	-0.039	0.055
rs1534430	T	C	9.40E-08	-0.09	0.017	0.005	0.012
rs17490285	G	A	9.26E-07	-0.18	0.038	0.000	0.019
rs1887414	C	T	1.97E-07	0.12	0.022	-0.016	0.017
rs2111485	G	A	4.19E-06	0.08	0.017	0.023	0.012
rs229526	C	G	1.93E-06	0.10	0.022	-0.015	0.015
rs2476601	G	A	3.49E-45	-0.33	0.023	-0.008	0.019
rs28367132	A	G	1.21E-07	-0.14	0.026	-0.030	0.015
rs28450758	A	C	4.17E-06	0.14	0.031	-0.030	0.023
rs34536443	C	G	2.47E-07	-0.25	0.049	-0.087	0.030
rs34636506	G	A	1.92E-14	-0.13	0.018	-0.023	0.012
rs61830417	G	A	3.59E-06	0.27	0.059	-0.008	0.042
rs62076103	G	A	1.23E-07	-0.19	0.035	-0.021	0.025
rs6448432	A	G	1.75E-06	0.09	0.019	0.003	0.013
rs6501550	T	C	1.42E-06	-0.18	0.037	0.039	0.035

Supplementary Table 2. Details of Hashimoto's Thyroiditis predicting SNPs with inflammatory proteins from IEU Open GWAS (with SNPs reaching $P < 5 \times 10^{-6}$).

Inflammatory proteins				Hashimoto's Thyroiditis			
SNP	Effect allele	Other allele	P value	Beta	Standard Error	log(OR)	Standard Error
rs7030280	T	C	6.44E-31	0.21	0.018	0.006	0.013
rs72808590	C	T	1.55E-06	-0.37	0.077	-0.029	0.045
rs73165154	G	A	3.76E-06	-0.15	0.033	-0.008	0.022
rs73192661	T	C	1.45E-09	-0.10	0.017	-0.004	0.012
rs7335748	G	A	2.41E-06	0.38	0.080	0.001	0.041
rs766932	T	A	4.96E-07	0.10	0.019	-0.008	0.013
rs7767668	T	C	9.60E-07	-0.09	0.019	-0.014	0.012
rs7861040	C	G	9.08E-07	0.08	0.017	-0.011	0.012
rs79219789	C	G	2.84E-06	-0.15	0.033	0.030	0.031
rs8084503	T	C	1.97E-07	0.14	0.027	-0.011	0.018
rs847803	G	A	7.91E-07	0.09	0.018	-0.010	0.013
rs9271365	G	T	1.44E-39	0.25	0.019	0.000	0.012
rs9357119	T	G	3.65E-14	-0.15	0.020	0.001	0.013
rs9497965	T	C	1.71E-06	0.08	0.017	-0.009	0.012
**CXCL5 (F-statistic = 38.11, R2=0.44%)**							
rs10424978	A	C	8.97E-07	-0.08	0.017	-0.005	0.012
rs10494077	A	G	9.20E-10	0.12	0.020	-0.001	0.014
rs10786679	A	G	1.87E-06	-0.09	0.018	-0.017	0.014
rs111358861	G	A	3.30E-06	1.82	0.391	-0.055	0.106
rs1115587	A	G	1.82E-07	-0.10	0.018	0.014	0.012
rs11211645	G	A	9.33E-08	0.09	0.017	-0.044	0.012
rs113075322	G	C	7.18E-07	-0.14	0.028	-0.013	0.033
rs11611029	T	C	1.16E-08	-0.10	0.017	0.013	0.012
rs117167029	C	G	8.05E-07	2.99	0.606	-0.133	0.089
rs117369326	A	G	3.55E-06	0.24	0.051	-0.017	0.029
rs118075239	C	T	3.45E-06	0.38	0.083	-0.031	0.047
rs11822254	G	A	3.52E-06	-0.08	0.017	-0.017	0.012
rs11889341	T	C	6.64E-11	0.12	0.019	-0.009	0.014
rs1317983	C	T	1.65E-07	0.09	0.018	-0.001	0.013
rs13249705	G	A	3.78E-07	-0.09	0.018	-0.015	0.014
rs140111921	A	G	1.34E-06	-0.16	0.034	0.023	0.031

Supplementary Table 2. Details of Hashimoto's Thyroiditis predicting SNPs with inflammatory proteins from IEU Open GWAS (with SNPs reaching $P < 5 \times 10^{-6}$).

Inflammatory proteins				Hashimoto's Thyroiditis			
SNP	Effect allele	Other allele	P value	Beta	Standard Error	log(OR)	Standard Error
rs150619443	C	G	5.08E-07	0.44	0.088	0.016	0.054
rs1534430	T	C	9.40E-08	-0.09	0.017	0.010	0.012
rs17490285	G	A	9.26E-07	-0.18	0.038	0.001	0.019
rs1887414	C	T	1.97E-07	0.12	0.022	-0.010	0.017
rs2111485	G	A	4.19E-06	0.08	0.017	0.008	0.012
rs229526	C	G	1.93E-06	0.10	0.022	0.009	0.015
rs2476601	G	A	3.49E-45	-0.33	0.023	0.032	0.019
rs28367132	A	G	1.21E-07	-0.14	0.026	-0.013	0.015
rs28450758	A	C	4.17E-06	0.14	0.031	0.002	0.023
rs3184504	C	T	1.40E-25	-0.18	0.017	-0.015	0.013
rs34536443	C	G	2.47E-07	-0.25	0.049	0.011	0.031
rs34636506	G	A	1.92E-14	-0.13	0.018	0.012	0.012
rs61830417	G	A	3.59E-06	0.27	0.059	-0.064	0.042
rs62076103	G	A	1.23E-07	-0.19	0.035	-0.007	0.025
rs6448432	A	G	1.75E-06	0.09	0.019	0.008	0.013
rs6501550	T	C	1.42E-06	-0.18	0.037	-0.032	0.035
rs7030280	T	C	6.44E-31	0.21	0.018	0.002	0.013
rs72808590	C	T	1.55E-06	-0.37	0.077	-0.025	0.045
rs73165154	G	A	3.76E-06	-0.15	0.033	-0.005	0.022
rs73192661	T	C	1.45E-09	-0.10	0.017	-0.013	0.012
rs7335748	G	A	2.41E-06	0.38	0.080	0.030	0.041
rs766932	T	A	4.96E-07	0.10	0.019	-0.003	0.013
rs7767668	T	C	9.60E-07	-0.09	0.019	-0.006	0.012
rs7861040	C	G	9.08E-07	0.08	0.017	-0.009	0.012
rs79219789	C	G	2.84E-06	-0.15	0.033	0.009	0.031
rs8084503	T	C	1.97E-07	0.14	0.027	0.025	0.019
rs847803	G	A	7.91E-07	0.09	0.018	0.002	0.013
rs9271365	G	T	1.44E-39	0.25	0.019	-0.003	0.012
rs9357119	T	G	3.65E-14	-0.15	0.020	0.012	0.013
rs9497965	T	C	1.71E-06	0.08	0.017	-0.001	0.012
**CXCL6 (F-statistic = 36.52, R2=0.44%)**							

Supplementary Table 2. Details of Hashimoto's Thyroiditis predicting SNPs with inflammatory proteins from IEU Open GWAS (with SNPs reaching $P < 5 \times 10^{-6}$).

Inflammatory proteins				Hashimoto's Thyroiditis			
SNP	Effect allele	Other allele	P value	Beta	Standard Error	log(OR)	Standard Error
rs10424978	A	C	8.97E-07	-0.08	0.017	-0.017	0.012
rs10494077	A	G	9.20E-10	0.12	0.020	0.010	0.014
rs10786679	A	G	1.87E-06	-0.09	0.018	-0.001	0.014
rs111358861	G	A	3.30E-06	1.82	0.391	-0.131	0.107
rs1115587	A	G	1.82E-07	-0.10	0.018	0.005	0.012
rs11211645	G	A	9.33E-08	0.09	0.017	-0.030	0.012
rs113075322	G	C	7.18E-07	-0.14	0.028	0.025	0.033
rs11611029	T	C	1.16E-08	-0.10	0.017	0.011	0.012
rs117167029	C	G	8.05E-07	2.99	0.606	-0.097	0.090
rs117369326	A	G	3.55E-06	0.24	0.051	0.031	0.029
rs118075239	C	T	3.45E-06	0.38	0.083	-0.053	0.048
rs11822254	G	A	3.52E-06	-0.08	0.017	-0.002	0.012
rs11889341	T	C	6.64E-11	0.12	0.019	-0.008	0.014
rs1317983	C	T	1.65E-07	0.09	0.018	-0.001	0.013
rs13249705	G	A	3.78E-07	-0.09	0.018	-0.012	0.014
rs140111921	A	G	1.34E-06	-0.16	0.034	0.024	0.031
rs150619443	C	G	5.08E-07	0.44	0.088	0.011	0.054
rs1534430	T	C	9.40E-08	-0.09	0.017	0.007	0.012
rs17490285	G	A	9.26E-07	-0.18	0.038	-0.012	0.019
rs1887414	C	T	1.97E-07	0.12	0.022	-0.006	0.017
rs2111485	G	A	4.19E-06	0.08	0.017	-0.012	0.012
rs229526	C	G	1.93E-06	0.10	0.022	0.016	0.015
rs2476601	G	A	3.49E-45	-0.33	0.023	0.021	0.019
rs28367132	A	G	1.21E-07	-0.14	0.026	-0.023	0.015
rs28450758	A	C	4.17E-06	0.14	0.031	-0.009	0.023
rs34536443	C	G	2.47E-07	-0.25	0.049	0.015	0.030
rs34636506	G	A	1.92E-14	-0.13	0.018	0.004	0.012
rs61830417	G	A	3.59E-06	0.27	0.059	-0.033	0.042
rs62076103	G	A	1.23E-07	-0.19	0.035	-0.026	0.025
rs6448432	A	G	1.75E-06	0.09	0.019	-0.001	0.013
rs6501550	T	C	1.42E-06	-0.18	0.037	-0.016	0.035

Supplementary Table 2. Details of Hashimoto's Thyroiditis predicting SNPs with inflammatory proteins from IEU Open GWAS (with SNPs reaching $P < 5 \times 10^{-6}$).

Inflammatory proteins						Hashimoto's Thyroiditis	
SNP	Effect allele	Other allele	P value	Beta	Standard Error	log(OR)	Standard Error
rs7030280	T	C	6.44E-31	0.21	0.018	-0.002	0.013
rs72808590	C	T	1.55E-06	-0.37	0.077	-0.011	0.045
rs73165154	G	A	3.76E-06	-0.15	0.033	-0.002	0.022
rs73192661	T	C	1.45E-09	-0.10	0.017	-0.011	0.012
rs7335748	G	A	2.41E-06	0.38	0.080	0.008	0.041
rs766932	T	A	4.96E-07	0.10	0.019	-0.001	0.013
rs7767668	T	C	9.60E-07	-0.09	0.019	-0.015	0.012
rs7861040	C	G	9.08E-07	0.08	0.017	-0.006	0.012
rs79219789	C	G	2.84E-06	-0.15	0.033	-0.003	0.031
rs8084503	T	C	1.97E-07	0.14	0.027	-0.006	0.018
rs847803	G	A	7.91E-07	0.09	0.018	0.010	0.013
rs9271365	G	T	1.44E-39	0.25	0.019	0.010	0.012
rs9357119	T	G	3.65E-14	-0.15	0.020	-0.002	0.013
rs9497965	T	C	1.71E-06	0.08	0.017	-0.003	0.012
**CXCL9 (F-statistic = 36.24, R2=0.39%)**							
rs10424978	A	C	8.97E-07	-0.08	0.017	-0.027	0.012
rs10494077	A	G	9.20E-10	0.12	0.020	0.004	0.014
rs10786679	A	G	1.87E-06	-0.09	0.018	-0.020	0.013
rs111358861	G	A	3.30E-06	1.82	0.391	0.031	0.106
rs1115587	A	G	1.82E-07	-0.10	0.018	0.005	0.012
rs11211645	G	A	9.33E-08	0.09	0.017	-0.011	0.012
rs113075322	G	C	7.18E-07	-0.14	0.028	-0.006	0.032
rs11611029	T	C	1.16E-08	-0.10	0.017	0.000	0.012
rs117167029	C	G	8.05E-07	2.99	0.606	-0.010	0.087
rs117369326	A	G	3.55E-06	0.24	0.051	0.030	0.028
rs118075239	C	T	3.45E-06	0.38	0.083	0.027	0.046
rs11822254	G	A	3.52E-06	-0.08	0.017	0.011	0.012
rs11889341	T	C	6.64E-11	0.12	0.019	0.037	0.014
rs1317983	C	T	1.65E-07	0.09	0.018	0.012	0.012
rs13249705	G	A	3.78E-07	-0.09	0.018	0.010	0.014
rs140111921	A	G	1.34E-06	-0.16	0.034	0.037	0.029

Supplementary Table 2. Details of Hashimoto's Thyroiditis predicting SNPs with inflammatory proteins from IEU Open GWAS (with SNPs reaching $P < 5 \times 10^{-6}$).

Inflammatory proteins				Hashimoto's Thyroiditis			
SNP	Effect allele	Other allele	P value	Beta	Standard Error	log(OR)	Standard Error
rs150619443	C	G	5.08E-07	0.44	0.088	0.018	0.053
rs1534430	T	C	9.40E-08	-0.09	0.017	-0.013	0.012
rs17490285	G	A	9.26E-07	-0.18	0.038	0.005	0.018
rs1887414	C	T	1.97E-07	0.12	0.022	-0.002	0.016
rs2111485	G	A	4.19E-06	0.08	0.017	0.024	0.011
rs229526	C	G	1.93E-06	0.10	0.022	-0.009	0.014
rs2476601	G	A	3.49E-45	-0.33	0.023	-0.024	0.019
rs28367132	A	G	1.21E-07	-0.14	0.026	-0.010	0.015
rs28450758	A	C	4.17E-06	0.14	0.031	0.019	0.022
rs61830417	G	A	3.59E-06	0.27	0.059	0.006	0.040
rs62076103	G	A	1.23E-07	-0.19	0.035	-0.046	0.024
rs6448432	A	G	1.75E-06	0.09	0.019	0.013	0.013
rs6501550	T	C	1.42E-06	-0.18	0.037	0.076	0.033
rs7030280	T	C	6.44E-31	0.21	0.018	-0.009	0.012
rs72808590	C	T	1.55E-06	-0.37	0.077	-0.082	0.044
rs73165154	G	A	3.76E-06	-0.15	0.033	-0.003	0.021
rs73192661	T	C	1.45E-09	-0.10	0.017	0.000	0.012
rs7335748	G	A	2.41E-06	0.38	0.080	0.027	0.040
rs766932	T	A	4.96E-07	0.10	0.019	-0.014	0.013
rs7767668	T	C	9.60E-07	-0.09	0.019	-0.025	0.012
rs7861040	C	G	9.08E-07	0.08	0.017	-0.006	0.012
rs79219789	C	G	2.84E-06	-0.15	0.033	0.005	0.030
rs8084503	T	C	1.97E-07	0.14	0.027	-0.012	0.018
rs847803	G	A	7.91E-07	0.09	0.018	-0.016	0.012
rs9271365	G	T	1.44E-39	0.25	0.019	-0.023	0.012
rs9357119	T	G	3.65E-14	-0.15	0.020	-0.012	0.013
rs9497965	T	C	1.71E-06	0.08	0.017	0.000	0.012
**DNER (F-statistic = 38.23, R2=0.43%)**							
rs10424978	A	C	8.97E-07	-0.08	0.017	-0.001	0.012
rs10494077	A	G	9.20E-10	0.12	0.020	0.017	0.014
rs10786679	A	G	1.87E-06	-0.09	0.018	0.003	0.013

Supplementary Table 2. Details of Hashimoto's Thyroiditis predicting SNPs with inflammatory proteins from IEU Open GWAS (with SNPs reaching $P < 5 \times 10^{-6}$).

Inflammatory proteins				Hashimoto's Thyroiditis			
SNP	Effect allele	Other allele	P value	Beta	Standard Error	log(OR)	Standard Error
rs111358861	G	A	3.30E-06	1.82	0.391	0.163	0.107
rs1115587	A	G	1.82E-07	-0.10	0.018	0.023	0.012
rs11211645	G	A	9.33E-08	0.09	0.017	0.012	0.012
rs113075322	G	C	7.18E-07	-0.14	0.028	-0.019	0.033
rs117167029	C	G	8.05E-07	2.99	0.606	-0.015	0.086
rs117369326	A	G	3.55E-06	0.24	0.051	0.016	0.028
rs118075239	C	T	3.45E-06	0.38	0.083	0.069	0.046
rs11822254	G	A	3.52E-06	-0.08	0.017	-0.007	0.012
rs11889341	T	C	6.64E-11	0.12	0.019	-0.008	0.014
rs1317983	C	T	1.65E-07	0.09	0.018	-0.003	0.013
rs13249705	G	A	3.78E-07	-0.09	0.018	-0.029	0.014
rs140111921	A	G	1.34E-06	-0.16	0.034	0.008	0.030
rs150619443	C	G	5.08E-07	0.44	0.088	-0.054	0.053
rs1534430	T	C	9.40E-08	-0.09	0.017	-0.010	0.012
rs17490285	G	A	9.26E-07	-0.18	0.038	0.014	0.019
rs1887414	C	T	1.97E-07	0.12	0.022	0.012	0.016
rs2111485	G	A	4.19E-06	0.08	0.017	0.003	0.011
rs229526	C	G	1.93E-06	0.10	0.022	0.013	0.014
rs2476601	G	A	3.49E-45	-0.33	0.023	-0.008	0.019
rs28367132	A	G	1.21E-07	-0.14	0.026	-0.017	0.015
rs28450758	A	C	4.17E-06	0.14	0.031	-0.028	0.022
rs3184504	C	T	1.40E-25	-0.18	0.017	-0.015	0.013
rs34536443	C	G	2.47E-07	-0.25	0.049	0.046	0.030
rs34636506	G	A	1.92E-14	-0.13	0.018	0.022	0.012
rs61830417	G	A	3.59E-06	0.27	0.059	-0.085	0.040
rs62076103	G	A	1.23E-07	-0.19	0.035	-0.040	0.024
rs6448432	A	G	1.75E-06	0.09	0.019	0.005	0.013
rs6501550	T	C	1.42E-06	-0.18	0.037	-0.041	0.034
rs7030280	T	C	6.44E-31	0.21	0.018	-0.025	0.012
rs72808590	C	T	1.55E-06	-0.37	0.077	0.040	0.044
rs73165154	G	A	3.76E-06	-0.15	0.033	0.002	0.022

Supplementary Table 2. Details of Hashimoto's Thyroiditis predicting SNPs with inflammatory proteins from IEU Open GWAS (with SNPs reaching $P < 5 \times 10^{-6}$).

Inflammatory proteins				Hashimoto's Thyroiditis			
SNP	Effect allele	Other allele	P value	Beta	Standard Error	log(OR)	Standard Error
rs73192661	T	C	1.45E-09	-0.10	0.017	-0.003	0.012
rs7335748	G	A	2.41E-06	0.38	0.080	0.015	0.040
rs766932	T	A	4.96E-07	0.10	0.019	-0.003	0.013
rs7767668	T	C	9.60E-07	-0.09	0.019	-0.005	0.012
rs7861040	C	G	9.08E-07	0.08	0.017	-0.001	0.012
rs79219789	C	G	2.84E-06	-0.15	0.033	0.026	0.030
rs8084503	T	C	1.97E-07	0.14	0.027	0.023	0.018
rs847803	G	A	7.91E-07	0.09	0.018	-0.001	0.013
rs9271365	G	T	1.44E-39	0.25	0.019	0.011	0.011
rs9357119	T	G	3.65E-14	-0.15	0.020	-0.004	0.013
rs9497965	T	C	1.71E-06	0.08	0.017	-0.022	0.012
**EIF4E-BPI (F-statistic = 38.11, R2=0.44%)**							
rs10424978	A	C	8.97E-07	-0.08	0.017	0.002	0.012
rs10494077	A	G	9.20E-10	0.12	0.020	0.006	0.014
rs10786679	A	G	1.87E-06	-0.09	0.018	-0.011	0.014
rs111358861	G	A	3.30E-06	1.82	0.391	-0.150	0.106
rs1115587	A	G	1.82E-07	-0.10	0.018	0.008	0.012
rs11211645	G	A	9.33E-08	0.09	0.017	-0.018	0.012
rs113075322	G	C	7.18E-07	-0.14	0.028	-0.011	0.033
rs11611029	T	C	1.16E-08	-0.10	0.017	0.003	0.012
rs117167029	C	G	8.05E-07	2.99	0.606	0.029	0.090
rs117369326	A	G	3.55E-06	0.24	0.051	-0.006	0.030
rs118075239	C	T	3.45E-06	0.38	0.083	0.006	0.047
rs11822254	G	A	3.52E-06	-0.08	0.017	-0.013	0.012
rs11889341	T	C	6.64E-11	0.12	0.019	-0.010	0.014
rs1317983	C	T	1.65E-07	0.09	0.018	0.013	0.013
rs13249705	G	A	3.78E-07	-0.09	0.018	-0.001	0.014
rs140111921	A	G	1.34E-06	-0.16	0.034	0.016	0.031
rs150619443	C	G	5.08E-07	0.44	0.088	-0.080	0.055
rs1534430	T	C	9.40E-08	-0.09	0.017	0.016	0.012
rs17490285	G	A	9.26E-07	-0.18	0.038	-0.021	0.019

Supplementary Table 2. Details of Hashimoto's Thyroiditis predicting SNPs with inflammatory proteins from IEU Open GWAS (with SNPs reaching $P < 5 \times 10^{-6}$).

Inflammatory proteins				Hashimoto's Thyroiditis			
SNP	Effect allele	Other allele	P value	Beta	Standard Error	log(OR)	Standard Error
rs1887414	C	T	1.97E-07	0.12	0.022	-0.001	0.017
rs2111485	G	A	4.19E-06	0.08	0.017	0.007	0.012
rs229526	C	G	1.93E-06	0.10	0.022	0.008	0.015
rs2476601	G	A	3.49E-45	-0.33	0.023	-0.002	0.020
rs28367132	A	G	1.21E-07	-0.14	0.026	-0.015	0.015
rs28450758	A	C	4.17E-06	0.14	0.031	-0.040	0.023
rs3184504	C	T	1.40E-25	-0.18	0.017	-0.026	0.013
rs34536443	C	G	2.47E-07	-0.25	0.049	-0.040	0.031
rs34636506	G	A	1.92E-14	-0.13	0.018	0.009	0.012
rs61830417	G	A	3.59E-06	0.27	0.059	0.012	0.042
rs62076103	G	A	1.23E-07	-0.19	0.035	0.005	0.025
rs6448432	A	G	1.75E-06	0.09	0.019	0.022	0.013
rs6501550	T	C	1.42E-06	-0.18	0.037	-0.009	0.035
rs7030280	T	C	6.44E-31	0.21	0.018	0.003	0.013
rs72808590	C	T	1.55E-06	-0.37	0.077	-0.014	0.045
rs73165154	G	A	3.76E-06	-0.15	0.033	-0.010	0.022
rs73192661	T	C	1.45E-09	-0.10	0.017	-0.015	0.012
rs7335748	G	A	2.41E-06	0.38	0.080	0.025	0.041
rs766932	T	A	4.96E-07	0.10	0.019	-0.013	0.013
rs7767668	T	C	9.60E-07	-0.09	0.019	0.000	0.012
rs7861040	C	G	9.08E-07	0.08	0.017	-0.015	0.012
rs79219789	C	G	2.84E-06	-0.15	0.033	-0.011	0.031
rs8084503	T	C	1.97E-07	0.14	0.027	0.011	0.019
rs847803	G	A	7.91E-07	0.09	0.018	0.012	0.013
rs9271365	G	T	1.44E-39	0.25	0.019	0.003	0.012
rs9357119	T	G	3.65E-14	-0.15	0.020	-0.021	0.013
rs9497965	T	C	1.71E-06	0.08	0.017	0.008	0.012
**Eotaxin (F-statistic = 38.11, R2=0.44%)**							
rs10424978	A	C	8.97E-07	-0.08	0.017	-0.007	0.012
rs10494077	A	G	9.20E-10	0.12	0.020	0.011	0.014
rs10786679	A	G	1.87E-06	-0.09	0.018	0.000	0.013

Supplementary Table 2. Details of Hashimoto's Thyroiditis predicting SNPs with inflammatory proteins from IEU Open GWAS (with SNPs reaching $P < 5 \times 10^{-6}$).

Inflammatory proteins				Hashimoto's Thyroiditis			
SNP	Effect allele	Other allele	P value	Beta	Standard Error	log(OR)	Standard Error
rs111358861	G	A	3.30E-06	1.82	0.391	0.073	0.107
rs1115587	A	G	1.82E-07	-0.10	0.018	0.018	0.012
rs11211645	G	A	9.33E-08	0.09	0.017	-0.009	0.012
rs113075322	G	C	7.18E-07	-0.14	0.028	0.012	0.033
rs11611029	T	C	1.16E-08	-0.10	0.017	0.013	0.012
rs117167029	C	G	8.05E-07	2.99	0.606	0.007	0.089
rs117369326	A	G	3.55E-06	0.24	0.051	-0.024	0.029
rs118075239	C	T	3.45E-06	0.38	0.083	0.052	0.047
rs11822254	G	A	3.52E-06	-0.08	0.017	0.020	0.012
rs11889341	T	C	6.64E-11	0.12	0.019	-0.008	0.014
rs1317983	C	T	1.65E-07	0.09	0.018	0.001	0.013
rs13249705	G	A	3.78E-07	-0.09	0.018	0.001	0.014
rs140111921	A	G	1.34E-06	-0.16	0.034	-0.038	0.030
rs150619443	C	G	5.08E-07	0.44	0.088	-0.019	0.054
rs1534430	T	C	9.40E-08	-0.09	0.017	0.010	0.012
rs17490285	G	A	9.26E-07	-0.18	0.038	0.016	0.019
rs1887414	C	T	1.97E-07	0.12	0.022	-0.019	0.016
rs2111485	G	A	4.19E-06	0.08	0.017	0.001	0.012
rs229526	C	G	1.93E-06	0.10	0.022	0.020	0.015
rs2476601	G	A	3.49E-45	-0.33	0.023	0.013	0.019
rs28367132	A	G	1.21E-07	-0.14	0.026	-0.034	0.015
rs28450758	A	C	4.17E-06	0.14	0.031	0.006	0.022
rs3184504	C	T	1.40E-25	-0.18	0.017	-0.041	0.013
rs34536443	C	G	2.47E-07	-0.25	0.049	-0.021	0.030
rs34636506	G	A	1.92E-14	-0.13	0.018	-0.010	0.012
rs61830417	G	A	3.59E-06	0.27	0.059	-0.009	0.041
rs62076103	G	A	1.23E-07	-0.19	0.035	-0.028	0.024
rs6448432	A	G	1.75E-06	0.09	0.019	0.002	0.013
rs6501550	T	C	1.42E-06	-0.18	0.037	0.013	0.034
rs7030280	T	C	6.44E-31	0.21	0.018	-0.022	0.012
rs72808590	C	T	1.55E-06	-0.37	0.077	-0.069	0.044

Supplementary Table 2. Details of Hashimoto's Thyroiditis predicting SNPs with inflammatory proteins from IEU Open GWAS (with SNPs reaching $P < 5 \times 10^{-6}$).

Inflammatory proteins						Hashimoto's Thyroiditis	
SNP	Effect allele	Other allele	P value	Beta	Standard Error	log(OR)	Standard Error
rs73165154	G	A	3.76E-06	-0.15	0.033	-0.011	0.022
rs73192661	T	C	1.45E-09	-0.10	0.017	-0.001	0.012
rs7335748	G	A	2.41E-06	0.38	0.080	-0.030	0.041
rs766932	T	A	4.96E-07	0.10	0.019	-0.013	0.013
rs7767668	T	C	9.60E-07	-0.09	0.019	-0.008	0.012
rs7861040	C	G	9.08E-07	0.08	0.017	-0.015	0.012
rs79219789	C	G	2.84E-06	-0.15	0.033	0.010	0.031
rs8084503	T	C	1.97E-07	0.14	0.027	0.010	0.018
rs847803	G	A	7.91E-07	0.09	0.018	0.014	0.013
rs9271365	G	T	1.44E-39	0.25	0.019	-0.004	0.012
rs9357119	T	G	3.65E-14	-0.15	0.020	-0.028	0.013
rs9497965	T	C	1.71E-06	0.08	0.017	-0.007	0.012
**FGF19 (F-statistic = 38.11, R2=0.44%)**							
rs10424978	A	C	8.97E-07	-0.08	0.017	-0.015	0.012
rs10494077	A	G	9.20E-10	0.12	0.020	-0.005	0.014
rs10786679	A	G	1.87E-06	-0.09	0.018	0.011	0.014
rs111358861	G	A	3.30E-06	1.82	0.391	0.029	0.107
rs1115587	A	G	1.82E-07	-0.10	0.018	0.006	0.012
rs11211645	G	A	9.33E-08	0.09	0.017	0.016	0.012
rs113075322	G	C	7.18E-07	-0.14	0.028	-0.034	0.033
rs11611029	T	C	1.16E-08	-0.10	0.017	0.002	0.012
rs117167029	C	G	8.05E-07	2.99	0.606	-0.121	0.090
rs117369326	A	G	3.55E-06	0.24	0.051	0.027	0.029
rs118075239	C	T	3.45E-06	0.38	0.083	0.008	0.048
rs11822254	G	A	3.52E-06	-0.08	0.017	-0.001	0.012
rs11889341	T	C	6.64E-11	0.12	0.019	0.002	0.014
rs1317983	C	T	1.65E-07	0.09	0.018	-0.011	0.013
rs13249705	G	A	3.78E-07	-0.09	0.018	-0.006	0.014
rs140111921	A	G	1.34E-06	-0.16	0.034	-0.031	0.030
rs150619443	C	G	5.08E-07	0.44	0.088	-0.035	0.055
rs1534430	T	C	9.40E-08	-0.09	0.017	0.000	0.012

Supplementary Table 2. Details of Hashimoto's Thyroiditis predicting SNPs with inflammatory proteins from IEU Open GWAS (with SNPs reaching $P < 5 \times 10^{-6}$).

Inflammatory proteins						Hashimoto's Thyroiditis	
SNP	Effect allele	Other allele	P value	Beta	Standard Error	log(OR)	Standard Error
rs17490285	G	A	9.26E-07	-0.18	0.038	0.004	0.019
rs1887414	C	T	1.97E-07	0.12	0.022	0.003	0.017
rs2111485	G	A	4.19E-06	0.08	0.017	-0.010	0.012
rs229526	C	G	1.93E-06	0.10	0.022	-0.002	0.015
rs2476601	G	A	3.49E-45	-0.33	0.023	-0.005	0.020
rs28367132	A	G	1.21E-07	-0.14	0.026	0.007	0.015
rs28450758	A	C	4.17E-06	0.14	0.031	-0.014	0.023
rs3184504	C	T	1.40E-25	-0.18	0.017	0.003	0.013
rs34536443	C	G	2.47E-07	-0.25	0.049	0.053	0.031
rs34636506	G	A	1.92E-14	-0.13	0.018	-0.007	0.012
rs61830417	G	A	3.59E-06	0.27	0.059	0.016	0.042
rs62076103	G	A	1.23E-07	-0.19	0.035	-0.036	0.025
rs6448432	A	G	1.75E-06	0.09	0.019	-0.021	0.013
rs6501550	T	C	1.42E-06	-0.18	0.037	0.009	0.035
rs7030280	T	C	6.44E-31	0.21	0.018	-0.010	0.013
rs72808590	C	T	1.55E-06	-0.37	0.077	-0.002	0.045
rs73165154	G	A	3.76E-06	-0.15	0.033	-0.038	0.022
rs73192661	T	C	1.45E-09	-0.10	0.017	-0.011	0.012
rs7335748	G	A	2.41E-06	0.38	0.080	0.002	0.041
rs766932	T	A	4.96E-07	0.10	0.019	0.006	0.013
rs7767668	T	C	9.60E-07	-0.09	0.019	-0.021	0.012
rs7861040	C	G	9.08E-07	0.08	0.017	-0.009	0.012
rs79219789	C	G	2.84E-06	-0.15	0.033	-0.078	0.031
rs8084503	T	C	1.97E-07	0.14	0.027	0.013	0.019
rs847803	G	A	7.91E-07	0.09	0.018	-0.018	0.013
rs9271365	G	T	1.44E-39	0.25	0.019	-0.001	0.012
rs9357119	T	G	3.65E-14	-0.15	0.020	-0.002	0.013
rs9497965	T	C	1.71E-06	0.08	0.017	0.002	0.012
**FGF21 (F-statistic = 38.11, R2=0.44%)**							
rs10424978	A	C	8.97E-07	-0.08	0.017	-0.014	0.012
rs10494077	A	G	9.20E-10	0.12	0.020	0.002	0.014

Supplementary Table 2. Details of Hashimoto's Thyroiditis predicting SNPs with inflammatory proteins from IEU Open GWAS (with SNPs reaching $P < 5 \times 10^{-6}$).

Inflammatory proteins				Hashimoto's Thyroiditis			
SNP	Effect allele	Other allele	P value	Beta	Standard Error	log(OR)	Standard Error
rs10786679	A	G	1.87E-06	-0.09	0.018	0.019	0.014
rs111358861	G	A	3.30E-06	1.82	0.391	0.250	0.107
rs1115587	A	G	1.82E-07	-0.10	0.018	0.014	0.012
rs11211645	G	A	9.33E-08	0.09	0.017	0.007	0.012
rs113075322	G	C	7.18E-07	-0.14	0.028	-0.002	0.033
rs11611029	T	C	1.16E-08	-0.10	0.017	0.005	0.012
rs117167029	C	G	8.05E-07	2.99	0.606	-0.079	0.091
rs117369326	A	G	3.55E-06	0.24	0.051	0.012	0.029
rs118075239	C	T	3.45E-06	0.38	0.083	-0.020	0.048
rs11822254	G	A	3.52E-06	-0.08	0.017	-0.002	0.012
rs11889341	T	C	6.64E-11	0.12	0.019	-0.039	0.014
rs1317983	C	T	1.65E-07	0.09	0.018	-0.012	0.013
rs13249705	G	A	3.78E-07	-0.09	0.018	0.000	0.014
rs140111921	A	G	1.34E-06	-0.16	0.034	-0.039	0.030
rs150619443	C	G	5.08E-07	0.44	0.088	-0.061	0.055
rs1534430	T	C	9.40E-08	-0.09	0.017	0.013	0.012
rs17490285	G	A	9.26E-07	-0.18	0.038	0.001	0.019
rs1887414	C	T	1.97E-07	0.12	0.022	-0.020	0.017
rs2111485	G	A	4.19E-06	0.08	0.017	-0.003	0.012
rs229526	C	G	1.93E-06	0.10	0.022	0.024	0.015
rs2476601	G	A	3.49E-45	-0.33	0.023	0.020	0.020
rs28367132	A	G	1.21E-07	-0.14	0.026	-0.015	0.015
rs28450758	A	C	4.17E-06	0.14	0.031	-0.001	0.023
rs3184504	C	T	1.40E-25	-0.18	0.017	0.009	0.013
rs34536443	C	G	2.47E-07	-0.25	0.049	0.040	0.031
rs34636506	G	A	1.92E-14	-0.13	0.018	0.010	0.012
rs61830417	G	A	3.59E-06	0.27	0.059	0.016	0.042
rs62076103	G	A	1.23E-07	-0.19	0.035	0.024	0.025
rs6448432	A	G	1.75E-06	0.09	0.019	0.006	0.013
rs6501550	T	C	1.42E-06	-0.18	0.037	-0.027	0.035
rs7030280	T	C	6.44E-31	0.21	0.018	-0.024	0.013

Supplementary Table 2. Details of Hashimoto's Thyroiditis predicting SNPs with inflammatory proteins from IEU Open GWAS (with SNPs reaching $P < 5 \times 10^{-6}$).

Inflammatory proteins				Hashimoto's Thyroiditis			
SNP	Effect allele	Other allele	P value	Beta	Standard Error	log(OR)	Standard Error
rs72808590	C	T	1.55E-06	-0.37	0.077	-0.057	0.045
rs73165154	G	A	3.76E-06	-0.15	0.033	0.012	0.022
rs73192661	T	C	1.45E-09	-0.10	0.017	-0.016	0.012
rs7335748	G	A	2.41E-06	0.38	0.080	0.007	0.041
rs766932	T	A	4.96E-07	0.10	0.019	0.033	0.013
rs7767668	T	C	9.60E-07	-0.09	0.019	-0.011	0.012
rs7861040	C	G	9.08E-07	0.08	0.017	0.001	0.012
rs79219789	C	G	2.84E-06	-0.15	0.033	0.012	0.031
rs8084503	T	C	1.97E-07	0.14	0.027	0.018	0.019
rs847803	G	A	7.91E-07	0.09	0.018	-0.019	0.013
rs9271365	G	T	1.44E-39	0.25	0.019	-0.013	0.012
rs9357119	T	G	3.65E-14	-0.15	0.020	0.006	0.013
rs9497965	T	C	1.71E-06	0.08	0.017	0.004	0.012
**FGF23 (F-statistic = 38.11, R2=0.44%)**							
rs10424978	A	C	8.97E-07	-0.08	0.017	-0.017	0.012
rs10494077	A	G	9.20E-10	0.12	0.020	0.003	0.014
rs10786679	A	G	1.87E-06	-0.09	0.018	0.001	0.013
rs111358861	G	A	3.30E-06	1.82	0.391	0.163	0.107
rs1115587	A	G	1.82E-07	-0.10	0.018	0.012	0.012
rs11211645	G	A	9.33E-08	0.09	0.017	-0.016	0.012
rs113075322	G	C	7.18E-07	-0.14	0.028	-0.020	0.033
rs11611029	T	C	1.16E-08	-0.10	0.017	0.012	0.012
rs117167029	C	G	8.05E-07	2.99	0.606	0.051	0.088
rs117369326	A	G	3.55E-06	0.24	0.051	-0.038	0.029
rs118075239	C	T	3.45E-06	0.38	0.083	0.063	0.046
rs11822254	G	A	3.52E-06	-0.08	0.017	-0.006	0.012
rs11889341	T	C	6.64E-11	0.12	0.019	-0.009	0.013
rs1317983	C	T	1.65E-07	0.09	0.018	0.012	0.013
rs13249705	G	A	3.78E-07	-0.09	0.018	-0.024	0.014
rs140111921	A	G	1.34E-06	-0.16	0.034	-0.015	0.030
rs150619443	C	G	5.08E-07	0.44	0.088	0.030	0.053

Supplementary Table 2. Details of Hashimoto's Thyroiditis predicting SNPs with inflammatory proteins from IEU Open GWAS (with SNPs reaching $P < 5 \times 10^{-6}$).

Inflammatory proteins				Hashimoto's Thyroiditis			
SNP	Effect allele	Other allele	P value	Beta	Standard Error	log(OR)	Standard Error
rs1534430	T	C	9.40E-08	-0.09	0.017	-0.031	0.012
rs17490285	G	A	9.26E-07	-0.18	0.038	-0.040	0.019
rs1887414	C	T	1.97E-07	0.12	0.022	0.009	0.016
rs2111485	G	A	4.19E-06	0.08	0.017	0.002	0.011
rs229526	C	G	1.93E-06	0.10	0.022	-0.007	0.014
rs2476601	G	A	3.49E-45	-0.33	0.023	0.038	0.018
rs28367132	A	G	1.21E-07	-0.14	0.026	-0.003	0.015
rs28450758	A	C	4.17E-06	0.14	0.031	-0.005	0.022
rs3184504	C	T	1.40E-25	-0.18	0.017	-0.009	0.013
rs34536443	C	G	2.47E-07	-0.25	0.049	-0.020	0.029
rs34636506	G	A	1.92E-14	-0.13	0.018	0.014	0.012
rs61830417	G	A	3.59E-06	0.27	0.059	-0.014	0.040
rs62076103	G	A	1.23E-07	-0.19	0.035	0.028	0.024
rs6448432	A	G	1.75E-06	0.09	0.019	-0.010	0.012
rs6501550	T	C	1.42E-06	-0.18	0.037	0.009	0.034
rs7030280	T	C	6.44E-31	0.21	0.018	-0.027	0.013
rs72808590	C	T	1.55E-06	-0.37	0.077	0.004	0.043
rs73165154	G	A	3.76E-06	-0.15	0.033	-0.017	0.022
rs73192661	T	C	1.45E-09	-0.10	0.017	0.016	0.012
rs7335748	G	A	2.41E-06	0.38	0.080	-0.016	0.040
rs766932	T	A	4.96E-07	0.10	0.019	0.007	0.013
rs7767668	T	C	9.60E-07	-0.09	0.019	-0.005	0.012
rs7861040	C	G	9.08E-07	0.08	0.017	-0.005	0.012
rs79219789	C	G	2.84E-06	-0.15	0.033	0.005	0.030
rs8084503	T	C	1.97E-07	0.14	0.027	0.006	0.018
rs847803	G	A	7.91E-07	0.09	0.018	0.029	0.013
rs9271365	G	T	1.44E-39	0.25	0.019	0.039	0.011
rs9357119	T	G	3.65E-14	-0.15	0.020	-0.001	0.013
rs9497965	T	C	1.71E-06	0.08	0.017	0.002	0.012
**FGF5 (F-statistic = 38.11, R2=0.44%)**							
rs10424978	A	C	8.97E-07	-0.08	0.017	-0.009	0.014

Supplementary Table 2. Details of Hashimoto's Thyroiditis predicting SNPs with inflammatory proteins from IEU Open GWAS (with SNPs reaching $P < 5 \times 10^{-6}$).

Inflammatory proteins				Hashimoto's Thyroiditis			
SNP	Effect allele	Other allele	P value	Beta	Standard Error	log(OR)	Standard Error
rs10494077	A	G	9.20E-10	0.12	0.020	0.013	0.015
rs10786679	A	G	1.87E-06	-0.09	0.018	0.004	0.015
rs111358861	G	A	3.30E-06	1.82	0.391	0.058	0.106
rs1115587	A	G	1.82E-07	-0.10	0.018	0.033	0.014
rs11211645	G	A	9.33E-08	0.09	0.017	-0.010	0.013
rs113075322	G	C	7.18E-07	-0.14	0.028	-0.054	0.036
rs11611029	T	C	1.16E-08	-0.10	0.017	-0.004	0.014
rs117167029	C	G	8.05E-07	2.99	0.606	0.020	0.111
rs117369326	A	G	3.55E-06	0.24	0.051	-0.004	0.032
rs118075239	C	T	3.45E-06	0.38	0.083	0.048	0.050
rs11822254	G	A	3.52E-06	-0.08	0.017	0.017	0.013
rs11889341	T	C	6.64E-11	0.12	0.019	-0.013	0.015
rs1317983	C	T	1.65E-07	0.09	0.018	0.008	0.014
rs13249705	G	A	3.78E-07	-0.09	0.018	-0.023	0.016
rs140111921	A	G	1.34E-06	-0.16	0.034	-0.012	0.033
rs150619443	C	G	5.08E-07	0.44	0.088	-0.003	0.056
rs1534430	T	C	9.40E-08	-0.09	0.017	0.005	0.014
rs17490285	G	A	9.26E-07	-0.18	0.038	0.002	0.021
rs1887414	C	T	1.97E-07	0.12	0.022	0.009	0.018
rs2111485	G	A	4.19E-06	0.08	0.017	-0.005	0.013
rs229526	C	G	1.93E-06	0.10	0.022	0.010	0.016
rs2476601	G	A	3.49E-45	-0.33	0.023	0.024	0.020
rs28367132	A	G	1.21E-07	-0.14	0.026	-0.017	0.016
rs28450758	A	C	4.17E-06	0.14	0.031	-0.036	0.024
rs3184504	C	T	1.40E-25	-0.18	0.017	0.013	0.012
rs34536443	C	G	2.47E-07	-0.25	0.049	0.040	0.032
rs34636506	G	A	1.92E-14	-0.13	0.018	0.004	0.013
rs61830417	G	A	3.59E-06	0.27	0.059	-0.042	0.044
rs62076103	G	A	1.23E-07	-0.19	0.035	0.012	0.025
rs6448432	A	G	1.75E-06	0.09	0.019	0.010	0.014
rs6501550	T	C	1.42E-06	-0.18	0.037	-0.007	0.039

Supplementary Table 2. Details of Hashimoto's Thyroiditis predicting SNPs with inflammatory proteins from IEU Open GWAS (with SNPs reaching $P < 5 \times 10^{-6}$).

Inflammatory proteins				Hashimoto's Thyroiditis			
SNP	Effect allele	Other allele	P value	Beta	Standard Error	log(OR)	Standard Error
rs7030280	T	C	6.44E-31	0.21	0.018	-0.014	0.014
rs72808590	C	T	1.55E-06	-0.37	0.077	0.033	0.046
rs73165154	G	A	3.76E-06	-0.15	0.033	0.002	0.024
rs73192661	T	C	1.45E-09	-0.10	0.017	0.003	0.014
rs7335748	G	A	2.41E-06	0.38	0.080	0.027	0.045
rs766932	T	A	4.96E-07	0.10	0.019	0.002	0.014
rs7767668	T	C	9.60E-07	-0.09	0.019	0.005	0.013
rs7861040	C	G	9.08E-07	0.08	0.017	-0.004	0.013
rs79219789	C	G	2.84E-06	-0.15	0.033	-0.032	0.034
rs8084503	T	C	1.97E-07	0.14	0.027	-0.007	0.020
rs847803	G	A	7.91E-07	0.09	0.018	-0.013	0.014
rs9271365	G	T	1.44E-39	0.25	0.019	-0.003	0.013
rs9357119	T	G	3.65E-14	-0.15	0.020	0.011	0.014
rs9497965	T	C	1.71E-06	0.08	0.017	-0.009	0.013
**FLT3L (F-statistic = 38.11, R2=0.44%)**							
rs10424978	A	C	8.97E-07	-0.08	0.017	-0.017	0.012
rs10494077	A	G	9.20E-10	0.12	0.020	0.007	0.014
rs10786679	A	G	1.87E-06	-0.09	0.018	0.027	0.013
rs111358861	G	A	3.30E-06	1.82	0.391	0.121	0.107
rs1115587	A	G	1.82E-07	-0.10	0.018	0.003	0.012
rs11211645	G	A	9.33E-08	0.09	0.017	0.003	0.012
rs113075322	G	C	7.18E-07	-0.14	0.028	0.003	0.033
rs11611029	T	C	1.16E-08	-0.10	0.017	-0.014	0.012
rs117167029	C	G	8.05E-07	2.99	0.606	-0.059	0.090
rs117369326	A	G	3.55E-06	0.24	0.051	-0.017	0.029
rs118075239	C	T	3.45E-06	0.38	0.083	-0.075	0.047
rs11822254	G	A	3.52E-06	-0.08	0.017	-0.001	0.012
rs11889341	T	C	6.64E-11	0.12	0.019	0.003	0.014
rs1317983	C	T	1.65E-07	0.09	0.018	-0.009	0.013
rs13249705	G	A	3.78E-07	-0.09	0.018	0.009	0.014
rs140111921	A	G	1.34E-06	-0.16	0.034	-0.031	0.030

Supplementary Table 2. Details of Hashimoto's Thyroiditis predicting SNPs with inflammatory proteins from IEU Open GWAS (with SNPs reaching $P < 5 \times 10^{-6}$).

Inflammatory proteins				Hashimoto's Thyroiditis			
SNP	Effect allele	Other allele	P value	Beta	Standard Error	log(OR)	Standard Error
rs150619443	C	G	5.08E-07	0.44	0.088	-0.025	0.054
rs1534430	T	C	9.40E-08	-0.09	0.017	-0.007	0.012
rs17490285	G	A	9.26E-07	-0.18	0.038	0.001	0.019
rs1887414	C	T	1.97E-07	0.12	0.022	0.004	0.017
rs2111485	G	A	4.19E-06	0.08	0.017	-0.006	0.012
rs229526	C	G	1.93E-06	0.10	0.022	0.021	0.015
rs2476601	G	A	3.49E-45	-0.33	0.023	0.005	0.019
rs28367132	A	G	1.21E-07	-0.14	0.026	-0.037	0.015
rs28450758	A	C	4.17E-06	0.14	0.031	-0.008	0.022
rs3184504	C	T	1.40E-25	-0.18	0.017	0.016	0.013
rs34536443	C	G	2.47E-07	-0.25	0.049	0.001	0.030
rs34636506	G	A	1.92E-14	-0.13	0.018	0.027	0.012
rs61830417	G	A	3.59E-06	0.27	0.059	-0.040	0.041
rs62076103	G	A	1.23E-07	-0.19	0.035	-0.042	0.024
rs6448432	A	G	1.75E-06	0.09	0.019	0.027	0.013
rs6501550	T	C	1.42E-06	-0.18	0.037	-0.004	0.034
rs7030280	T	C	6.44E-31	0.21	0.018	-0.013	0.013
rs72808590	C	T	1.55E-06	-0.37	0.077	-0.036	0.044
rs73165154	G	A	3.76E-06	-0.15	0.033	0.022	0.022
rs73192661	T	C	1.45E-09	-0.10	0.017	0.006	0.012
rs7335748	G	A	2.41E-06	0.38	0.080	-0.018	0.041
rs766932	T	A	4.96E-07	0.10	0.019	-0.001	0.013
rs7767668	T	C	9.60E-07	-0.09	0.019	-0.008	0.012
rs7861040	C	G	9.08E-07	0.08	0.017	-0.026	0.012
rs79219789	C	G	2.84E-06	-0.15	0.033	0.042	0.031
rs8084503	T	C	1.97E-07	0.14	0.027	0.003	0.018
rs847803	G	A	7.91E-07	0.09	0.018	-0.006	0.013
rs9271365	G	T	1.44E-39	0.25	0.019	0.001	0.012
rs9357119	T	G	3.65E-14	-0.15	0.020	-0.001	0.013
rs9497965	T	C	1.71E-06	0.08	0.017	0.006	0.012
**GDNF (F-statistic = 38.11, R2=0.44%)**							

Supplementary Table 2. Details of Hashimoto's Thyroiditis predicting SNPs with inflammatory proteins from IEU Open GWAS (with SNPs reaching $P < 5 \times 10^{-6}$).

Inflammatory proteins				Hashimoto's Thyroiditis			
SNP	Effect allele	Other allele	P value	Beta	Standard Error	log(OR)	Standard Error
rs10424978	A	C	8.97E-07	-0.08	0.017	-0.025	0.012
rs10494077	A	G	9.20E-10	0.12	0.020	0.025	0.014
rs10786679	A	G	1.87E-06	-0.09	0.018	0.004	0.013
rs111358861	G	A	3.30E-06	1.82	0.391	0.108	0.106
rs1115587	A	G	1.82E-07	-0.10	0.018	0.011	0.012
rs11211645	G	A	9.33E-08	0.09	0.017	-0.003	0.012
rs113075322	G	C	7.18E-07	-0.14	0.028	-0.042	0.033
rs11611029	T	C	1.16E-08	-0.10	0.017	0.010	0.012
rs117167029	C	G	8.05E-07	2.99	0.606	-0.073	0.091
rs117369326	A	G	3.55E-06	0.24	0.051	-0.010	0.029
rs118075239	C	T	3.45E-06	0.38	0.083	0.080	0.047
rs11822254	G	A	3.52E-06	-0.08	0.017	0.010	0.012
rs11889341	T	C	6.64E-11	0.12	0.019	0.019	0.014
rs1317983	C	T	1.65E-07	0.09	0.018	0.012	0.013
rs13249705	G	A	3.78E-07	-0.09	0.018	-0.005	0.014
rs140111921	A	G	1.34E-06	-0.16	0.034	-0.019	0.030
rs150619443	C	G	5.08E-07	0.44	0.088	0.076	0.053
rs1534430	T	C	9.40E-08	-0.09	0.017	-0.013	0.012
rs17490285	G	A	9.26E-07	-0.18	0.038	-0.018	0.019
rs1887414	C	T	1.97E-07	0.12	0.022	-0.019	0.016
rs2111485	G	A	4.19E-06	0.08	0.017	-0.003	0.012
rs229526	C	G	1.93E-06	0.10	0.022	-0.001	0.014
rs2476601	G	A	3.49E-45	-0.33	0.023	0.016	0.019
rs28367132	A	G	1.21E-07	-0.14	0.026	0.002	0.015
rs28450758	A	C	4.17E-06	0.14	0.031	-0.007	0.022
rs3184504	C	T	1.40E-25	-0.18	0.017	-0.031	0.013
rs34536443	C	G	2.47E-07	-0.25	0.049	0.002	0.030
rs34636506	G	A	1.92E-14	-0.13	0.018	0.016	0.012
rs61830417	G	A	3.59E-06	0.27	0.059	-0.028	0.041
rs62076103	G	A	1.23E-07	-0.19	0.035	-0.015	0.024
rs6448432	A	G	1.75E-06	0.09	0.019	0.000	0.013

Supplementary Table 2. Details of Hashimoto's Thyroiditis predicting SNPs with inflammatory proteins from IEU Open GWAS (with SNPs reaching *P* < 5 x 10^{-6}).

Inflammatory proteins				Hashimoto's Thyroiditis			
SNP	Effect allele	Other allele	P value	Beta	Standard Error	log(OR)	Standard Error
rs6501550	T	C	1.42E-06	-0.18	0.037	0.041	0.035
rs7030280	T	C	6.44E-31	0.21	0.018	0.000	0.013
rs72808590	C	T	1.55E-06	-0.37	0.077	-0.053	0.044
rs73165154	G	A	3.76E-06	-0.15	0.033	0.023	0.022
rs73192661	T	C	1.45E-09	-0.10	0.017	0.000	0.012
rs7335748	G	A	2.41E-06	0.38	0.080	-0.005	0.040
rs766932	T	A	4.96E-07	0.10	0.019	0.004	0.013
rs7767668	T	C	9.60E-07	-0.09	0.019	0.006	0.012
rs7861040	C	G	9.08E-07	0.08	0.017	0.004	0.012
rs79219789	C	G	2.84E-06	-0.15	0.033	-0.030	0.030
rs8084503	T	C	1.97E-07	0.14	0.027	0.007	0.018
rs847803	G	A	7.91E-07	0.09	0.018	-0.009	0.013
rs9271365	G	T	1.44E-39	0.25	0.019	-0.001	0.012
rs9357119	T	G	3.65E-14	-0.15	0.020	0.012	0.013
rs9497965	T	C	1.71E-06	0.08	0.017	0.013	0.012
**HGF (F-statistic = 36.52, R2=0.42%)**							
rs10424978	A	C	8.97E-07	-0.08	0.017	-0.016	0.012
rs10494077	A	G	9.20E-10	0.12	0.020	0.015	0.014
rs10786679	A	G	1.87E-06	-0.09	0.018	-0.005	0.013
rs111358861	G	A	3.30E-06	1.82	0.391	-0.036	0.107
rs1115587	A	G	1.82E-07	-0.10	0.018	0.025	0.012
rs11211645	G	A	9.33E-08	0.09	0.017	-0.012	0.012
rs113075322	G	C	7.18E-07	-0.14	0.028	0.009	0.033
rs11611029	T	C	1.16E-08	-0.10	0.017	-0.003	0.012
rs117167029	C	G	8.05E-07	2.99	0.606	0.025	0.092
rs117369326	A	G	3.55E-06	0.24	0.051	-0.011	0.029
rs118075239	C	T	3.45E-06	0.38	0.083	-0.014	0.046
rs11822254	G	A	3.52E-06	-0.08	0.017	-0.016	0.012
rs11889341	T	C	6.64E-11	0.12	0.019	-0.004	0.014
rs1317983	C	T	1.65E-07	0.09	0.018	0.022	0.013
rs13249705	G	A	3.78E-07	-0.09	0.018	0.025	0.014

Supplementary Table 2. Details of Hashimoto's Thyroiditis predicting SNPs with inflammatory proteins from IEU Open GWAS (with SNPs reaching $P < 5 \times 10^{-6}$).

Inflammatory proteins				Hashimoto's Thyroiditis			
SNP	Effect allele	Other allele	P value	Beta	Standard Error	log(OR)	Standard Error
rs140111921	A	G	1.34E-06	-0.16	0.034	-0.033	0.030
rs150619443	C	G	5.08E-07	0.44	0.088	-0.014	0.054
rs1534430	T	C	9.40E-08	-0.09	0.017	0.012	0.012
rs17490285	G	A	9.26E-07	-0.18	0.038	-0.019	0.019
rs1887414	C	T	1.97E-07	0.12	0.022	-0.010	0.016
rs2111485	G	A	4.19E-06	0.08	0.017	0.017	0.012
rs229526	C	G	1.93E-06	0.10	0.022	0.018	0.014
rs2476601	G	A	3.49E-45	-0.33	0.023	0.046	0.019
rs28367132	A	G	1.21E-07	-0.14	0.026	-0.026	0.015
rs28450758	A	C	4.17E-06	0.14	0.031	-0.044	0.022
rs34536443	C	G	2.47E-07	-0.25	0.049	0.009	0.030
rs34636506	G	A	1.92E-14	-0.13	0.018	0.000	0.012
rs61830417	G	A	3.59E-06	0.27	0.059	0.037	0.041
rs62076103	G	A	1.23E-07	-0.19	0.035	0.001	0.024
rs6448432	A	G	1.75E-06	0.09	0.019	-0.005	0.013
rs6501550	T	C	1.42E-06	-0.18	0.037	0.014	0.034
rs7030280	T	C	6.44E-31	0.21	0.018	-0.017	0.012
rs72808590	C	T	1.55E-06	-0.37	0.077	-0.100	0.044
rs73165154	G	A	3.76E-06	-0.15	0.033	-0.014	0.022
rs73192661	T	C	1.45E-09	-0.10	0.017	-0.003	0.012
rs7335748	G	A	2.41E-06	0.38	0.080	0.005	0.041
rs766932	T	A	4.96E-07	0.10	0.019	-0.001	0.013
rs7767668	T	C	9.60E-07	-0.09	0.019	0.003	0.012
rs7861040	C	G	9.08E-07	0.08	0.017	-0.006	0.012
rs79219789	C	G	2.84E-06	-0.15	0.033	0.037	0.030
rs8084503	T	C	1.97E-07	0.14	0.027	0.020	0.018
rs847803	G	A	7.91E-07	0.09	0.018	0.008	0.013
rs9271365	G	T	1.44E-39	0.25	0.019	-0.006	0.012
rs9357119	T	G	3.65E-14	-0.15	0.020	0.012	0.013
rs9497965	T	C	1.71E-06	0.08	0.017	-0.006	0.012
**IL-10 (F-statistic = 38.11, R2=0.44%)**							

Supplementary Table 2. Details of Hashimoto's Thyroiditis predicting SNPs with inflammatory proteins from IEU Open GWAS (with SNPs reaching *P* < 5 x 10^{-6}).

Inflammatory proteins				Hashimoto's Thyroiditis			
SNP	Effect allele	Other allele	P value	Beta	Standard Error	log(OR)	Standard Error
rs10424978	A	C	8.97E-07	-0.08	0.017	-0.034	0.012
rs10494077	A	G	9.20E-10	0.12	0.020	0.019	0.014
rs10786679	A	G	1.87E-06	-0.09	0.018	-0.021	0.014
rs111358861	G	A	3.30E-06	1.82	0.391	-0.103	0.107
rs1115587	A	G	1.82E-07	-0.10	0.018	0.003	0.012
rs11211645	G	A	9.33E-08	0.09	0.017	-0.018	0.012
rs113075322	G	C	7.18E-07	-0.14	0.028	-0.001	0.033
rs11611029	T	C	1.16E-08	-0.10	0.017	-0.003	0.012
rs117167029	C	G	8.05E-07	2.99	0.606	0.000	0.090
rs117369326	A	G	3.55E-06	0.24	0.051	-0.026	0.029
rs118075239	C	T	3.45E-06	0.38	0.083	0.060	0.047
rs11822254	G	A	3.52E-06	-0.08	0.017	0.016	0.012
rs11889341	T	C	6.64E-11	0.12	0.019	0.018	0.014
rs1317983	C	T	1.65E-07	0.09	0.018	-0.001	0.013
rs13249705	G	A	3.78E-07	-0.09	0.018	0.015	0.014
rs140111921	A	G	1.34E-06	-0.16	0.034	0.022	0.031
rs150619443	C	G	5.08E-07	0.44	0.088	-0.028	0.054
rs1534430	T	C	9.40E-08	-0.09	0.017	-0.010	0.012
rs17490285	G	A	9.26E-07	-0.18	0.038	-0.001	0.019
rs1887414	C	T	1.97E-07	0.12	0.022	-0.011	0.017
rs2111485	G	A	4.19E-06	0.08	0.017	-0.006	0.012
rs229526	C	G	1.93E-06	0.10	0.022	0.015	0.015
rs2476601	G	A	3.49E-45	-0.33	0.023	-0.049	0.019
rs28367132	A	G	1.21E-07	-0.14	0.026	-0.012	0.015
rs28450758	A	C	4.17E-06	0.14	0.031	-0.008	0.023
rs3184504	C	T	1.40E-25	-0.18	0.017	-0.042	0.013
rs34536443	C	G	2.47E-07	-0.25	0.049	0.038	0.030
rs34636506	G	A	1.92E-14	-0.13	0.018	-0.029	0.012
rs61830417	G	A	3.59E-06	0.27	0.059	0.081	0.042
rs62076103	G	A	1.23E-07	-0.19	0.035	-0.010	0.025
rs6448432	A	G	1.75E-06	0.09	0.019	-0.005	0.013

Supplementary Table 2. Details of Hashimoto's Thyroiditis predicting SNPs with inflammatory proteins from IEU Open GWAS (with SNPs reaching $P < 5 \times 10^{-6}$).

Inflammatory proteins				Hashimoto's Thyroiditis			
SNP	Effect allele	Other allele	P value	Beta	Standard Error	log(OR)	Standard Error
rs6501550	T	C	1.42E-06	-0.18	0.037	0.043	0.035
rs7030280	T	C	6.44E-31	0.21	0.018	-0.013	0.013
rs72808590	C	T	1.55E-06	-0.37	0.077	-0.036	0.045
rs73165154	G	A	3.76E-06	-0.15	0.033	0.031	0.022
rs73192661	T	C	1.45E-09	-0.10	0.017	-0.010	0.012
rs7335748	G	A	2.41E-06	0.38	0.080	0.010	0.041
rs766932	T	A	4.96E-07	0.10	0.019	-0.018	0.013
rs7767668	T	C	9.60E-07	-0.09	0.019	-0.035	0.012
rs7861040	C	G	9.08E-07	0.08	0.017	-0.013	0.012
rs79219789	C	G	2.84E-06	-0.15	0.033	-0.013	0.031
rs8084503	T	C	1.97E-07	0.14	0.027	-0.001	0.018
rs847803	G	A	7.91E-07	0.09	0.018	0.018	0.013
rs9271365	G	T	1.44E-39	0.25	0.019	-0.005	0.012
rs9357119	T	G	3.65E-14	-0.15	0.020	-0.029	0.013
rs9497965	T	C	1.71E-06	0.08	0.017	-0.003	0.012
**IL-10Rα (F-statistic = 38.11, R2=0.44%)**							
rs10424978	A	C	8.97E-07	-0.08	0.017	-0.007	0.014
rs10494077	A	G	9.20E-10	0.12	0.020	0.035	0.016
rs10786679	A	G	1.87E-06	-0.09	0.018	-0.011	0.015
rs111358861	G	A	3.30E-06	1.82	0.391	0.015	0.107
rs1115587	A	G	1.82E-07	-0.10	0.018	0.007	0.014
rs11211645	G	A	9.33E-08	0.09	0.017	-0.011	0.013
rs113075322	G	C	7.18E-07	-0.14	0.028	0.034	0.036
rs11611029	T	C	1.16E-08	-0.10	0.017	-0.014	0.014
rs117167029	C	G	8.05E-07	2.99	0.606	-0.183	0.111
rs117369326	A	G	3.55E-06	0.24	0.051	-0.002	0.033
rs118075239	C	T	3.45E-06	0.38	0.083	0.024	0.051
rs11822254	G	A	3.52E-06	-0.08	0.017	-0.002	0.014
rs11889341	T	C	6.64E-11	0.12	0.019	0.001	0.016
rs1317983	C	T	1.65E-07	0.09	0.018	0.012	0.015
rs13249705	G	A	3.78E-07	-0.09	0.018	-0.024	0.016

Supplementary Table 2. Details of Hashimoto's Thyroiditis predicting SNPs with inflammatory proteins from IEU Open GWAS (with SNPs reaching $P < 5 \times 10^{-6}$).

Inflammatory proteins				Hashimoto's Thyroiditis			
SNP	Effect allele	Other allele	P value	Beta	Standard Error	log(OR)	Standard Error
rs140111921	A	G	1.34E-06	-0.16	0.034	-0.027	0.033
rs150619443	C	G	5.08E-07	0.44	0.088	-0.002	0.058
rs1534430	T	C	9.40E-08	-0.09	0.017	-0.022	0.014
rs17490285	G	A	9.26E-07	-0.18	0.038	-0.013	0.021
rs1887414	C	T	1.97E-07	0.12	0.022	-0.013	0.018
rs2111485	G	A	4.19E-06	0.08	0.017	0.003	0.013
rs229526	C	G	1.93E-06	0.10	0.022	0.026	0.016
rs2476601	G	A	3.49E-45	-0.33	0.023	0.033	0.021
rs28367132	A	G	1.21E-07	-0.14	0.026	-0.008	0.017
rs28450758	A	C	4.17E-06	0.14	0.031	0.060	0.025
rs3184504	C	T	1.40E-25	-0.18	0.017	-0.023	0.013
rs34536443	C	G	2.47E-07	-0.25	0.049	-0.026	0.034
rs34636506	G	A	1.92E-14	-0.13	0.018	-0.011	0.014
rs61830417	G	A	3.59E-06	0.27	0.059	0.101	0.046
rs62076103	G	A	1.23E-07	-0.19	0.035	-0.037	0.026
rs6448432	A	G	1.75E-06	0.09	0.019	0.001	0.014
rs6501550	T	C	1.42E-06	-0.18	0.037	-0.041	0.040
rs7030280	T	C	6.44E-31	0.21	0.018	-0.017	0.014
rs72808590	C	T	1.55E-06	-0.37	0.077	0.018	0.048
rs73165154	G	A	3.76E-06	-0.15	0.033	0.042	0.024
rs73192661	T	C	1.45E-09	-0.10	0.017	-0.014	0.014
rs7335748	G	A	2.41E-06	0.38	0.080	0.017	0.046
rs766932	T	A	4.96E-07	0.10	0.019	-0.010	0.015
rs7767668	T	C	9.60E-07	-0.09	0.019	0.007	0.013
rs7861040	C	G	9.08E-07	0.08	0.017	-0.004	0.013
rs79219789	C	G	2.84E-06	-0.15	0.033	-0.001	0.035
rs8084503	T	C	1.97E-07	0.14	0.027	-0.037	0.021
rs847803	G	A	7.91E-07	0.09	0.018	0.009	0.014
rs9271365	G	T	1.44E-39	0.25	0.019	-0.004	0.013
rs9357119	T	G	3.65E-14	-0.15	0.020	0.017	0.015
rs9497965	T	C	1.71E-06	0.08	0.017	-0.024	0.013

Supplementary Table 2. Details of Hashimoto's Thyroiditis predicting SNPs with inflammatory proteins from IEU Open GWAS (with SNPs reaching $P < 5 \times 10^{-6}$).

Inflammatory proteins				Hashimoto's Thyroiditis			
SNP	Effect allele	Other allele	P value	Beta	Standard Error	log(OR)	Standard Error
**IL-10Rβ (F-statistic = 38.11, R2=0.44%)**							
rs10424978	A	C	8.97E-07	-0.08	0.017	-0.023	0.012
rs10494077	A	G	9.20E-10	0.12	0.020	0.007	0.014
rs10786679	A	G	1.87E-06	-0.09	0.018	0.000	0.013
rs111358861	G	A	3.30E-06	1.82	0.391	-0.055	0.106
rs1115587	A	G	1.82E-07	-0.10	0.018	-0.012	0.012
rs11211645	G	A	9.33E-08	0.09	0.017	0.002	0.012
rs113075322	G	C	7.18E-07	-0.14	0.028	0.015	0.033
rs11611029	T	C	1.16E-08	-0.10	0.017	-0.001	0.012
rs117167029	C	G	8.05E-07	2.99	0.606	-0.127	0.091
rs117369326	A	G	3.55E-06	0.24	0.051	0.010	0.029
rs118075239	C	T	3.45E-06	0.38	0.083	0.122	0.047
rs11822254	G	A	3.52E-06	-0.08	0.017	-0.006	0.012
rs11889341	T	C	6.64E-11	0.12	0.019	-0.012	0.014
rs1317983	C	T	1.65E-07	0.09	0.018	0.012	0.013
rs13249705	G	A	3.78E-07	-0.09	0.018	-0.008	0.014
rs140111921	A	G	1.34E-06	-0.16	0.034	-0.041	0.031
rs150619443	C	G	5.08E-07	0.44	0.088	-0.044	0.053
rs1534430	T	C	9.40E-08	-0.09	0.017	-0.016	0.012
rs17490285	G	A	9.26E-07	-0.18	0.038	-0.024	0.019
rs1887414	C	T	1.97E-07	0.12	0.022	-0.026	0.016
rs2111485	G	A	4.19E-06	0.08	0.017	-0.012	0.011
rs229526	C	G	1.93E-06	0.10	0.022	0.011	0.014
rs2476601	G	A	3.49E-45	-0.33	0.023	0.005	0.018
rs28367132	A	G	1.21E-07	-0.14	0.026	0.003	0.015
rs28450758	A	C	4.17E-06	0.14	0.031	-0.047	0.022
rs3184504	C	T	1.40E-25	-0.18	0.017	-0.007	0.013
rs34536443	C	G	2.47E-07	-0.25	0.049	0.042	0.030
rs34636506	G	A	1.92E-14	-0.13	0.018	0.002	0.012
rs61830417	G	A	3.59E-06	0.27	0.059	0.003	0.041
rs62076103	G	A	1.23E-07	-0.19	0.035	-0.017	0.024

Supplementary Table 2. Details of Hashimoto's Thyroiditis predicting SNPs with inflammatory proteins from IEU Open GWAS (with SNPs reaching $P < 5 \times 10^{-6}$).

Inflammatory proteins				Hashimoto's Thyroiditis			
SNP	Effect allele	Other allele	P value	Beta	Standard Error	log(OR)	Standard Error
rs6448432	A	G	1.75E-06	0.09	0.019	0.005	0.012
rs6501550	T	C	1.42E-06	-0.18	0.037	0.043	0.034
rs7030280	T	C	6.44E-31	0.21	0.018	-0.019	0.013
rs72808590	C	T	1.55E-06	-0.37	0.077	-0.080	0.044
rs73165154	G	A	3.76E-06	-0.15	0.033	0.011	0.022
rs73192661	T	C	1.45E-09	-0.10	0.017	-0.004	0.012
rs7335748	G	A	2.41E-06	0.38	0.080	-0.048	0.040
rs766932	T	A	4.96E-07	0.10	0.019	0.002	0.013
rs7767668	T	C	9.60E-07	-0.09	0.019	-0.006	0.012
rs7861040	C	G	9.08E-07	0.08	0.017	-0.003	0.012
rs79219789	C	G	2.84E-06	-0.15	0.033	-0.025	0.030
rs8084503	T	C	1.97E-07	0.14	0.027	0.034	0.018
rs847803	G	A	7.91E-07	0.09	0.018	0.002	0.013
rs9271365	G	T	1.44E-39	0.25	0.019	0.020	0.011
rs9357119	T	G	3.65E-14	-0.15	0.020	0.007	0.013
rs9497965	T	C	1.71E-06	0.08	0.017	-0.016	0.012
**IL-12p40 (F-statistic = 38.61, R2=0.38%)**							
rs10424978	A	C	8.97E-07	-0.08	0.017	-0.023	0.012
rs10494077	A	G	9.20E-10	0.12	0.020	0.019	0.014
rs10786679	A	G	1.87E-06	-0.09	0.018	-0.012	0.013
rs111358861	G	A	3.30E-06	1.82	0.391	-0.248	0.106
rs1115587	A	G	1.82E-07	-0.10	0.018	-0.019	0.012
rs11211645	G	A	9.33E-08	0.09	0.017	-0.008	0.012
rs113075322	G	C	7.18E-07	-0.14	0.028	0.031	0.033
rs11611029	T	C	1.16E-08	-0.10	0.017	-0.030	0.012
rs117167029	C	G	8.05E-07	2.99	0.606	-0.027	0.088
rs117369326	A	G	3.55E-06	0.24	0.051	0.003	0.029
rs118075239	C	T	3.45E-06	0.38	0.083	0.050	0.047
rs11822254	G	A	3.52E-06	-0.08	0.017	-0.009	0.012
rs11889341	T	C	6.64E-11	0.12	0.019	0.005	0.014
rs1317983	C	T	1.65E-07	0.09	0.018	-0.002	0.013

Supplementary Table 2. Details of Hashimoto's Thyroiditis predicting SNPs with inflammatory proteins from IEU Open GWAS (with SNPs reaching *P* < 5 x 10^{-6}).

Inflammatory proteins				Hashimoto's Thyroiditis			
SNP	Effect allele	Other allele	P value	Beta	Standard Error	log(OR)	Standard Error
rs13249705	G	A	3.78E-07	-0.09	0.018	0.005	0.014
rs140111921	A	G	1.34E-06	-0.16	0.034	0.002	0.031
rs150619443	C	G	5.08E-07	0.44	0.088	-0.007	0.055
rs1534430	T	C	9.40E-08	-0.09	0.017	0.004	0.012
rs17490285	G	A	9.26E-07	-0.18	0.038	0.010	0.019
rs1887414	C	T	1.97E-07	0.12	0.022	-0.028	0.017
rs2111485	G	A	4.19E-06	0.08	0.017	-0.009	0.012
rs229526	C	G	1.93E-06	0.10	0.022	0.016	0.015
rs2476601	G	A	3.49E-45	-0.33	0.023	-0.036	0.019
rs28367132	A	G	1.21E-07	-0.14	0.026	-0.017	0.015
rs28450758	A	C	4.17E-06	0.14	0.031	0.033	0.023
rs34536443	C	G	2.47E-07	-0.25	0.049	-0.032	0.030
rs34636506	G	A	1.92E-14	-0.13	0.018	-0.039	0.012
rs61830417	G	A	3.59E-06	0.27	0.059	0.014	0.042
rs62076103	G	A	1.23E-07	-0.19	0.035	-0.025	0.025
rs6448432	A	G	1.75E-06	0.09	0.019	0.004	0.013
rs6501550	T	C	1.42E-06	-0.18	0.037	0.041	0.034
rs7030280	T	C	6.44E-31	0.21	0.018	-0.020	0.013
rs72808590	C	T	1.55E-06	-0.37	0.077	-0.037	0.045
rs73165154	G	A	3.76E-06	-0.15	0.033	-0.006	0.022
rs7335748	G	A	2.41E-06	0.38	0.080	0.047	0.041
rs766932	T	A	4.96E-07	0.10	0.019	-0.007	0.013
rs7861040	C	G	9.08E-07	0.08	0.017	0.004	0.012
rs79219789	C	G	2.84E-06	-0.15	0.033	0.000	0.031
rs8084503	T	C	1.97E-07	0.14	0.027	-0.018	0.018
rs9271365	G	T	1.44E-39	0.25	0.019	0.010	0.012
rs9497965	T	C	1.71E-06	0.08	0.017	0.001	0.012
**IL-13 (F-statistic = 38.11, R2=0.44%)**							
rs10424978	A	C	8.97E-07	-0.08	0.017	-0.002	0.014
rs10494077	A	G	9.20E-10	0.12	0.020	0.016	0.016
rs10786679	A	G	1.87E-06	-0.09	0.018	-0.003	0.015

Supplementary Table 2. Details of Hashimoto's Thyroiditis predicting SNPs with inflammatory proteins from IEU Open GWAS (with SNPs reaching $P < 5 \times 10^{-6}$).

Inflammatory proteins				Hashimoto's Thyroiditis			
SNP	Effect allele	Other allele	P value	Beta	Standard Error	log(OR)	Standard Error
rs111358861	G	A	3.30E-06	1.82	0.391	0.076	0.107
rs1115587	A	G	1.82E-07	-0.10	0.018	0.025	0.014
rs11211645	G	A	9.33E-08	0.09	0.017	0.003	0.013
rs113075322	G	C	7.18E-07	-0.14	0.028	-0.034	0.036
rs11611029	T	C	1.16E-08	-0.10	0.017	-0.004	0.014
rs117167029	C	G	8.05E-07	2.99	0.606	0.015	0.111
rs117369326	A	G	3.55E-06	0.24	0.051	-0.039	0.033
rs118075239	C	T	3.45E-06	0.38	0.083	0.128	0.051
rs11822254	G	A	3.52E-06	-0.08	0.017	0.002	0.014
rs11889341	T	C	6.64E-11	0.12	0.019	0.015	0.016
rs1317983	C	T	1.65E-07	0.09	0.018	0.002	0.015
rs13249705	G	A	3.78E-07	-0.09	0.018	0.006	0.016
rs140111921	A	G	1.34E-06	-0.16	0.034	-0.008	0.033
rs150619443	C	G	5.08E-07	0.44	0.088	-0.016	0.058
rs1534430	T	C	9.40E-08	-0.09	0.017	-0.017	0.014
rs17490285	G	A	9.26E-07	-0.18	0.038	0.048	0.021
rs1887414	C	T	1.97E-07	0.12	0.022	-0.008	0.018
rs2111485	G	A	4.19E-06	0.08	0.017	0.010	0.013
rs229526	C	G	1.93E-06	0.10	0.022	-0.016	0.016
rs2476601	G	A	3.49E-45	-0.33	0.023	0.050	0.021
rs28367132	A	G	1.21E-07	-0.14	0.026	-0.021	0.017
rs28450758	A	C	4.17E-06	0.14	0.031	-0.004	0.025
rs3184504	C	T	1.40E-25	-0.18	0.017	-0.005	0.013
rs34536443	C	G	2.47E-07	-0.25	0.049	0.048	0.034
rs34636506	G	A	1.92E-14	-0.13	0.018	-0.012	0.014
rs61830417	G	A	3.59E-06	0.27	0.059	0.042	0.046
rs62076103	G	A	1.23E-07	-0.19	0.035	-0.001	0.026
rs6448432	A	G	1.75E-06	0.09	0.019	0.002	0.014
rs6501550	T	C	1.42E-06	-0.18	0.037	0.029	0.040
rs7030280	T	C	6.44E-31	0.21	0.018	-0.029	0.014
rs72808590	C	T	1.55E-06	-0.37	0.077	0.036	0.048

Supplementary Table 2. Details of Hashimoto's Thyroiditis predicting SNPs with inflammatory proteins from IEU Open GWAS (with SNPs reaching $P < 5 \times 10^{-6}$).

Inflammatory proteins				Hashimoto's Thyroiditis			
SNP	Effect allele	Other allele	P value	Beta	Standard Error	log(OR)	Standard Error
rs73165154	G	A	3.76E-06	-0.15	0.033	0.000	0.024
rs73192661	T	C	1.45E-09	-0.10	0.017	-0.003	0.014
rs7335748	G	A	2.41E-06	0.38	0.080	0.086	0.046
rs766932	T	A	4.96E-07	0.10	0.019	-0.009	0.015
rs7767668	T	C	9.60E-07	-0.09	0.019	0.002	0.013
rs7861040	C	G	9.08E-07	0.08	0.017	-0.007	0.013
rs79219789	C	G	2.84E-06	-0.15	0.033	0.015	0.035
rs8084503	T	C	1.97E-07	0.14	0.027	-0.026	0.021
rs847803	G	A	7.91E-07	0.09	0.018	0.019	0.014
rs9271365	G	T	1.44E-39	0.25	0.019	0.008	0.013
rs9357119	T	G	3.65E-14	-0.15	0.020	0.029	0.015
rs9497965	T	C	1.71E-06	0.08	0.017	-0.014	0.013
**IL-15Rα (F-statistic = 36.52, R2=0.42%)**							
rs10424978	A	C	8.97E-07	-0.08	0.017	0.014	0.014
rs10494077	A	G	9.20E-10	0.12	0.020	-0.023	0.015
rs10786679	A	G	1.87E-06	-0.09	0.018	-0.018	0.015
rs111358861	G	A	3.30E-06	1.82	0.391	-0.089	0.106
rs1115587	A	G	1.82E-07	-0.10	0.018	0.027	0.014
rs11211645	G	A	9.33E-08	0.09	0.017	-0.003	0.013
rs113075322	G	C	7.18E-07	-0.14	0.028	-0.025	0.036
rs11611029	T	C	1.16E-08	-0.10	0.017	-0.007	0.014
rs117167029	C	G	8.05E-07	2.99	0.606	-0.004	0.111
rs117369326	A	G	3.55E-06	0.24	0.051	-0.015	0.032
rs118075239	C	T	3.45E-06	0.38	0.083	0.016	0.050
rs11822254	G	A	3.52E-06	-0.08	0.017	0.009	0.013
rs11889341	T	C	6.64E-11	0.12	0.019	0.019	0.015
rs1317983	C	T	1.65E-07	0.09	0.018	0.017	0.014
rs13249705	G	A	3.78E-07	-0.09	0.018	-0.010	0.016
rs140111921	A	G	1.34E-06	-0.16	0.034	0.034	0.033
rs150619443	C	G	5.08E-07	0.44	0.088	0.009	0.056
rs1534430	T	C	9.40E-08	-0.09	0.017	0.006	0.014

Supplementary Table 2. Details of Hashimoto's Thyroiditis predicting SNPs with inflammatory proteins from IEU Open GWAS (with SNPs reaching $P < 5 \times 10^{-6}$).

Inflammatory proteins				Hashimoto's Thyroiditis			
SNP	Effect allele	Other allele	P value	Beta	Standard Error	log(OR)	Standard Error
rs17490285	G	A	9.26E-07	-0.18	0.038	-0.003	0.021
rs1887414	C	T	1.97E-07	0.12	0.022	-0.031	0.018
rs2111485	G	A	4.19E-06	0.08	0.017	0.004	0.013
rs229526	C	G	1.93E-06	0.10	0.022	-0.019	0.016
rs2476601	G	A	3.49E-45	-0.33	0.023	-0.005	0.020
rs28367132	A	G	1.21E-07	-0.14	0.026	-0.016	0.017
rs28450758	A	C	4.17E-06	0.14	0.031	0.015	0.024
rs34536443	C	G	2.47E-07	-0.25	0.049	-0.037	0.033
rs34636506	G	A	1.92E-14	-0.13	0.018	-0.009	0.013
rs61830417	G	A	3.59E-06	0.27	0.059	0.069	0.044
rs62076103	G	A	1.23E-07	-0.19	0.035	-0.058	0.025
rs6448432	A	G	1.75E-06	0.09	0.019	0.013	0.014
rs6501550	T	C	1.42E-06	-0.18	0.037	0.046	0.039
rs7030280	T	C	6.44E-31	0.21	0.018	-0.008	0.014
rs72808590	C	T	1.55E-06	-0.37	0.077	-0.050	0.047
rs73165154	G	A	3.76E-06	-0.15	0.033	0.014	0.024
rs73192661	T	C	1.45E-09	-0.10	0.017	0.014	0.014
rs7335748	G	A	2.41E-06	0.38	0.080	0.029	0.045
rs766932	T	A	4.96E-07	0.10	0.019	0.001	0.014
rs7767668	T	C	9.60E-07	-0.09	0.019	-0.002	0.013
rs7861040	C	G	9.08E-07	0.08	0.017	-0.018	0.013
rs79219789	C	G	2.84E-06	-0.15	0.033	0.035	0.034
rs8084503	T	C	1.97E-07	0.14	0.027	-0.021	0.020
rs847803	G	A	7.91E-07	0.09	0.018	0.021	0.014
rs9271365	G	T	1.44E-39	0.25	0.019	0.041	0.013
rs9357119	T	G	3.65E-14	-0.15	0.020	-0.012	0.014
rs9497965	T	C	1.71E-06	0.08	0.017	-0.006	0.013
**IL-17A (F-statistic = 35.97, R2=0.41%)**							
rs10424978	A	C	8.97E-07	-0.08	0.017	0.015	0.014
rs10494077	A	G	9.20E-10	0.12	0.020	-0.005	0.016
rs10786679	A	G	1.87E-06	-0.09	0.018	-0.032	0.015

Supplementary Table 2. Details of Hashimoto's Thyroiditis predicting SNPs with inflammatory proteins from IEU Open GWAS (with SNPs reaching *P* < 5 x 10^{-6}).

Inflammatory proteins				Hashimoto's Thyroiditis			
SNP	Effect allele	Other allele	P value	Beta	Standard Error	log(OR)	Standard Error
rs111358861	G	A	3.30E-06	1.82	0.391	0.156	0.106
rs1115587	A	G	1.82E-07	-0.10	0.018	0.042	0.014
rs11211645	G	A	9.33E-08	0.09	0.017	-0.026	0.013
rs113075322	G	C	7.18E-07	-0.14	0.028	-0.020	0.036
rs11611029	T	C	1.16E-08	-0.10	0.017	0.022	0.014
rs117167029	C	G	8.05E-07	2.99	0.606	-0.075	0.111
rs117369326	A	G	3.55E-06	0.24	0.051	-0.038	0.033
rs118075239	C	T	3.45E-06	0.38	0.083	-0.016	0.051
rs11822254	G	A	3.52E-06	-0.08	0.017	-0.004	0.014
rs11889341	T	C	6.64E-11	0.12	0.019	0.031	0.016
rs1317983	C	T	1.65E-07	0.09	0.018	-0.006	0.015
rs13249705	G	A	3.78E-07	-0.09	0.018	0.000	0.016
rs140111921	A	G	1.34E-06	-0.16	0.034	-0.049	0.033
rs150619443	C	G	5.08E-07	0.44	0.088	0.047	0.058
rs1534430	T	C	9.40E-08	-0.09	0.017	0.016	0.014
rs17490285	G	A	9.26E-07	-0.18	0.038	0.016	0.021
rs1887414	C	T	1.97E-07	0.12	0.022	0.005	0.018
rs2111485	G	A	4.19E-06	0.08	0.017	0.009	0.013
rs229526	C	G	1.93E-06	0.10	0.022	-0.036	0.016
rs2476601	G	A	3.49E-45	-0.33	0.023	0.039	0.021
rs28367132	A	G	1.21E-07	-0.14	0.026	-0.008	0.017
rs28450758	A	C	4.17E-06	0.14	0.031	-0.013	0.025
rs3184504	C	T	1.40E-25	-0.18	0.017	-0.024	0.013
rs34536443	C	G	2.47E-07	-0.25	0.049	-0.004	0.034
rs34636506	G	A	1.92E-14	-0.13	0.018	-0.038	0.014
rs61830417	G	A	3.59E-06	0.27	0.059	0.039	0.046
rs62076103	G	A	1.23E-07	-0.19	0.035	0.020	0.026
rs6448432	A	G	1.75E-06	0.09	0.019	-0.005	0.014
rs6501550	T	C	1.42E-06	-0.18	0.037	0.007	0.040
rs72808590	C	T	1.55E-06	-0.37	0.077	-0.048	0.048
rs73165154	G	A	3.76E-06	-0.15	0.033	-0.033	0.024

Supplementary Table 2. Details of Hashimoto's Thyroiditis predicting SNPs with inflammatory proteins from IEU Open GWAS (with SNPs reaching $P < 5 \times 10^{-6}$).

Inflammatory proteins				Hashimoto's Thyroiditis			
SNP	Effect allele	Other allele	P value	Beta	Standard Error	log(OR)	Standard Error
rs73192661	T	C	1.45E-09	-0.10	0.017	-0.012	0.014
rs7335748	G	A	2.41E-06	0.38	0.080	0.016	0.046
rs766932	T	A	4.96E-07	0.10	0.019	-0.001	0.015
rs7767668	T	C	9.60E-07	-0.09	0.019	0.013	0.013
rs7861040	C	G	9.08E-07	0.08	0.017	0.007	0.013
rs79219789	C	G	2.84E-06	-0.15	0.033	0.049	0.035
rs8084503	T	C	1.97E-07	0.14	0.027	0.013	0.021
rs847803	G	A	7.91E-07	0.09	0.018	-0.023	0.014
rs9271365	G	T	1.44E-39	0.25	0.019	0.026	0.013
rs9357119	T	G	3.65E-14	-0.15	0.020	0.023	0.015
rs9497965	T	C	1.71E-06	0.08	0.017	-0.025	0.013
**IL-17C (F-statistic = 38.11, R2=0.44%)**							
rs10424978	A	C	8.97E-07	-0.08	0.017	-0.015	0.014
rs10494077	A	G	9.20E-10	0.12	0.020	0.020	0.015
rs10786679	A	G	1.87E-06	-0.09	0.018	0.004	0.015
rs111358861	G	A	3.30E-06	1.82	0.391	0.176	0.106
rs1115587	A	G	1.82E-07	-0.10	0.018	0.017	0.014
rs11211645	G	A	9.33E-08	0.09	0.017	-0.016	0.013
rs113075322	G	C	7.18E-07	-0.14	0.028	-0.023	0.036
rs11611029	T	C	1.16E-08	-0.10	0.017	0.011	0.014
rs117167029	C	G	8.05E-07	2.99	0.606	0.089	0.111
rs117369326	A	G	3.55E-06	0.24	0.051	-0.016	0.033
rs118075239	C	T	3.45E-06	0.38	0.083	0.105	0.051
rs11822254	G	A	3.52E-06	-0.08	0.017	0.001	0.014
rs11889341	T	C	6.64E-11	0.12	0.019	0.003	0.016
rs1317983	C	T	1.65E-07	0.09	0.018	0.005	0.014
rs13249705	G	A	3.78E-07	-0.09	0.018	-0.006	0.016
rs140111921	A	G	1.34E-06	-0.16	0.034	0.017	0.033
rs150619443	C	G	5.08E-07	0.44	0.088	0.085	0.058
rs1534430	T	C	9.40E-08	-0.09	0.017	-0.020	0.014
rs17490285	G	A	9.26E-07	-0.18	0.038	0.016	0.021

Supplementary Table 2. Details of Hashimoto's Thyroiditis predicting SNPs with inflammatory proteins from IEU Open GWAS (with SNPs reaching $P < 5 \times 10^{-6}$).

Inflammatory proteins						Hashimoto's Thyroiditis	
SNP	Effect allele	Other allele	P value	Beta	Standard Error	log(OR)	Standard Error
rs1887414	C	T	1.97E-07	0.12	0.022	-0.006	0.018
rs2111485	G	A	4.19E-06	0.08	0.017	0.008	0.013
rs229526	C	G	1.93E-06	0.10	0.022	-0.008	0.016
rs2476601	G	A	3.49E-45	-0.33	0.023	0.006	0.021
rs28367132	A	G	1.21E-07	-0.14	0.026	-0.018	0.017
rs28450758	A	C	4.17E-06	0.14	0.031	-0.014	0.025
rs3184504	C	T	1.40E-25	-0.18	0.017	0.031	0.013
rs34536443	C	G	2.47E-07	-0.25	0.049	0.019	0.034
rs34636506	G	A	1.92E-14	-0.13	0.018	-0.003	0.014
rs61830417	G	A	3.59E-06	0.27	0.059	0.025	0.045
rs62076103	G	A	1.23E-07	-0.19	0.035	0.021	0.026
rs6448432	A	G	1.75E-06	0.09	0.019	0.003	0.014
rs6501550	T	C	1.42E-06	-0.18	0.037	-0.051	0.040
rs7030280	T	C	6.44E-31	0.21	0.018	-0.023	0.014
rs72808590	C	T	1.55E-06	-0.37	0.077	-0.071	0.048
rs73165154	G	A	3.76E-06	-0.15	0.033	-0.001	0.024
rs73192661	T	C	1.45E-09	-0.10	0.017	0.015	0.014
rs7335748	G	A	2.41E-06	0.38	0.080	0.025	0.046
rs766932	T	A	4.96E-07	0.10	0.019	0.024	0.015
rs7767668	T	C	9.60E-07	-0.09	0.019	0.010	0.013
rs7861040	C	G	9.08E-07	0.08	0.017	0.006	0.013
rs79219789	C	G	2.84E-06	-0.15	0.033	0.003	0.035
rs8084503	T	C	1.97E-07	0.14	0.027	-0.001	0.021
rs847803	G	A	7.91E-07	0.09	0.018	0.018	0.014
rs9271365	G	T	1.44E-39	0.25	0.019	0.022	0.013
rs9357119	T	G	3.65E-14	-0.15	0.020	0.015	0.015
rs9497965	T	C	1.71E-06	0.08	0.017	-0.016	0.013
**IL-18 (F-statistic = 36.52, R2=0.42%)**							
rs10424978	A	C	8.97E-07	-0.08	0.017	-0.009	0.012
rs10494077	A	G	9.20E-10	0.12	0.020	0.003	0.014
rs10786679	A	G	1.87E-06	-0.09	0.018	-0.016	0.014

Supplementary Table 2. Details of Hashimoto's Thyroiditis predicting SNPs with inflammatory proteins from IEU Open GWAS (with SNPs reaching $P < 5 \times 10^{-6}$).

Inflammatory proteins				Hashimoto's Thyroiditis			
SNP	Effect allele	Other allele	P value	Beta	Standard Error	log(OR)	Standard Error
rs111358861	G	A	3.30E-06	1.82	0.391	-0.261	0.106
rs1115587	A	G	1.82E-07	-0.10	0.018	0.014	0.012
rs11211645	G	A	9.33E-08	0.09	0.017	-0.008	0.012
rs113075322	G	C	7.18E-07	-0.14	0.028	0.022	0.033
rs11611029	T	C	1.16E-08	-0.10	0.017	-0.005	0.012
rs117167029	C	G	8.05E-07	2.99	0.606	0.054	0.090
rs117369326	A	G	3.55E-06	0.24	0.051	-0.013	0.029
rs118075239	C	T	3.45E-06	0.38	0.083	0.091	0.047
rs11822254	G	A	3.52E-06	-0.08	0.017	-0.009	0.012
rs11889341	T	C	6.64E-11	0.12	0.019	0.005	0.014
rs1317983	C	T	1.65E-07	0.09	0.018	0.033	0.013
rs13249705	G	A	3.78E-07	-0.09	0.018	0.004	0.014
rs140111921	A	G	1.34E-06	-0.16	0.034	-0.026	0.031
rs150619443	C	G	5.08E-07	0.44	0.088	-0.031	0.054
rs1534430	T	C	9.40E-08	-0.09	0.017	0.025	0.012
rs17490285	G	A	9.26E-07	-0.18	0.038	0.004	0.019
rs1887414	C	T	1.97E-07	0.12	0.022	-0.016	0.017
rs2111485	G	A	4.19E-06	0.08	0.017	-0.007	0.012
rs229526	C	G	1.93E-06	0.10	0.022	-0.019	0.015
rs2476601	G	A	3.49E-45	-0.33	0.023	-0.026	0.019
rs28367132	A	G	1.21E-07	-0.14	0.026	0.005	0.015
rs28450758	A	C	4.17E-06	0.14	0.031	-0.029	0.022
rs34536443	C	G	2.47E-07	-0.25	0.049	0.004	0.030
rs34636506	G	A	1.92E-14	-0.13	0.018	0.012	0.012
rs61830417	G	A	3.59E-06	0.27	0.059	0.108	0.041
rs62076103	G	A	1.23E-07	-0.19	0.035	0.004	0.024
rs6448432	A	G	1.75E-06	0.09	0.019	0.039	0.013
rs6501550	T	C	1.42E-06	-0.18	0.037	0.028	0.035
rs7030280	T	C	6.44E-31	0.21	0.018	-0.014	0.013
rs72808590	C	T	1.55E-06	-0.37	0.077	-0.067	0.045
rs73165154	G	A	3.76E-06	-0.15	0.033	-0.017	0.022

Supplementary Table 2. Details of Hashimoto's Thyroiditis predicting SNPs with inflammatory proteins from IEU Open GWAS (with SNPs reaching $P < 5 \times 10^{-6}$).

Inflammatory proteins				Hashimoto's Thyroiditis			
SNP	Effect allele	Other allele	P value	Beta	Standard Error	log(OR)	Standard Error
rs73192661	T	C	1.45E-09	-0.10	0.017	-0.006	0.012
rs7335748	G	A	2.41E-06	0.38	0.080	-0.023	0.041
rs766932	T	A	4.96E-07	0.10	0.019	-0.001	0.013
rs7767668	T	C	9.60E-07	-0.09	0.019	-0.007	0.012
rs7861040	C	G	9.08E-07	0.08	0.017	-0.009	0.012
rs79219789	C	G	2.84E-06	-0.15	0.033	0.014	0.031
rs8084503	T	C	1.97E-07	0.14	0.027	0.002	0.018
rs847803	G	A	7.91E-07	0.09	0.018	0.010	0.013
rs9271365	G	T	1.44E-39	0.25	0.019	-0.022	0.012
rs9357119	T	G	3.65E-14	-0.15	0.020	-0.022	0.013
rs9497965	T	C	1.71E-06	0.08	0.017	-0.011	0.012
**IL-18R1 (F-statistic = 38.11, R2=0.44%)**							
rs10424978	A	C	8.97E-07	-0.08	0.017	-0.029	0.012
rs10494077	A	G	9.20E-10	0.12	0.020	0.024	0.014
rs10786679	A	G	1.87E-06	-0.09	0.018	0.000	0.014
rs111358861	G	A	3.30E-06	1.82	0.391	-0.100	0.107
rs1115587	A	G	1.82E-07	-0.10	0.018	0.013	0.012
rs11211645	G	A	9.33E-08	0.09	0.017	-0.009	0.012
rs113075322	G	C	7.18E-07	-0.14	0.028	0.021	0.033
rs11611029	T	C	1.16E-08	-0.10	0.017	0.010	0.012
rs117167029	C	G	8.05E-07	2.99	0.606	-0.047	0.090
rs117369326	A	G	3.55E-06	0.24	0.051	0.003	0.029
rs118075239	C	T	3.45E-06	0.38	0.083	-0.013	0.048
rs11822254	G	A	3.52E-06	-0.08	0.017	-0.014	0.012
rs11889341	T	C	6.64E-11	0.12	0.019	-0.015	0.014
rs1317983	C	T	1.65E-07	0.09	0.018	0.010	0.013
rs13249705	G	A	3.78E-07	-0.09	0.018	-0.016	0.014
rs140111921	A	G	1.34E-06	-0.16	0.034	-0.030	0.031
rs150619443	C	G	5.08E-07	0.44	0.088	0.019	0.054
rs1534430	T	C	9.40E-08	-0.09	0.017	0.001	0.012
rs17490285	G	A	9.26E-07	-0.18	0.038	-0.022	0.019

Supplementary Table 2. Details of Hashimoto's Thyroiditis predicting SNPs with inflammatory proteins from IEU Open GWAS (with SNPs reaching $P < 5 \times 10^{-6}$).

Inflammatory proteins				Hashimoto's Thyroiditis			
SNP	Effect allele	Other allele	P value	Beta	Standard Error	log(OR)	Standard Error
rs1887414	C	T	1.97E-07	0.12	0.022	-0.029	0.017
rs2111485	G	A	4.19E-06	0.08	0.017	0.018	0.012
rs229526	C	G	1.93E-06	0.10	0.022	0.002	0.015
rs2476601	G	A	3.49E-45	-0.33	0.023	0.023	0.019
rs28367132	A	G	1.21E-07	-0.14	0.026	-0.001	0.015
rs28450758	A	C	4.17E-06	0.14	0.031	-0.008	0.022
rs3184504	C	T	1.40E-25	-0.18	0.017	-0.040	0.013
rs34536443	C	G	2.47E-07	-0.25	0.049	-0.018	0.030
rs34636506	G	A	1.92E-14	-0.13	0.018	0.007	0.012
rs61830417	G	A	3.59E-06	0.27	0.059	0.005	0.042
rs62076103	G	A	1.23E-07	-0.19	0.035	-0.010	0.024
rs6448432	A	G	1.75E-06	0.09	0.019	0.000	0.013
rs6501550	T	C	1.42E-06	-0.18	0.037	0.004	0.035
rs7030280	T	C	6.44E-31	0.21	0.018	-0.017	0.013
rs72808590	C	T	1.55E-06	-0.37	0.077	-0.054	0.044
rs73165154	G	A	3.76E-06	-0.15	0.033	0.035	0.022
rs73192661	T	C	1.45E-09	-0.10	0.017	0.009	0.012
rs7335748	G	A	2.41E-06	0.38	0.080	-0.040	0.041
rs766932	T	A	4.96E-07	0.10	0.019	-0.018	0.013
rs7767668	T	C	9.60E-07	-0.09	0.019	0.011	0.012
rs7861040	C	G	9.08E-07	0.08	0.017	-0.006	0.012
rs79219789	C	G	2.84E-06	-0.15	0.033	0.044	0.031
rs8084503	T	C	1.97E-07	0.14	0.027	-0.004	0.018
rs847803	G	A	7.91E-07	0.09	0.018	0.018	0.013
rs9271365	G	T	1.44E-39	0.25	0.019	0.010	0.012
rs9357119	T	G	3.65E-14	-0.15	0.020	0.014	0.013
rs9497965	T	C	1.71E-06	0.08	0.017	0.010	0.012
**IL-1α (F-statistic = 35.09, R2=0.40%)**							
rs10424978	A	C	8.97E-07	-0.08	0.017	-0.010	0.014
rs10494077	A	G	9.20E-10	0.12	0.020	0.022	0.015
rs10786679	A	G	1.87E-06	-0.09	0.018	-0.009	0.015

Supplementary Table 2. Details of Hashimoto's Thyroiditis predicting SNPs with inflammatory proteins from IEU Open GWAS (with SNPs reaching $P < 5 \times 10^{-6}$).

Inflammatory proteins				Hashimoto's Thyroiditis			
SNP	Effect allele	Other allele	P value	Beta	Standard Error	log(OR)	Standard Error
rs111358861	G	A	3.30E-06	1.82	0.391	-0.014	0.107
rs1115587	A	G	1.82E-07	-0.10	0.018	0.026	0.014
rs11211645	G	A	9.33E-08	0.09	0.017	-0.005	0.013
rs113075322	G	C	7.18E-07	-0.14	0.028	-0.044	0.036
rs11611029	T	C	1.16E-08	-0.10	0.017	0.002	0.014
rs117167029	C	G	8.05E-07	2.99	0.606	0.020	0.111
rs117369326	A	G	3.55E-06	0.24	0.051	-0.045	0.033
rs118075239	C	T	3.45E-06	0.38	0.083	0.019	0.051
rs11822254	G	A	3.52E-06	-0.08	0.017	-0.002	0.014
rs11889341	T	C	6.64E-11	0.12	0.019	0.008	0.016
rs1317983	C	T	1.65E-07	0.09	0.018	-0.001	0.015
rs13249705	G	A	3.78E-07	-0.09	0.018	-0.030	0.016
rs140111921	A	G	1.34E-06	-0.16	0.034	-0.034	0.033
rs150619443	C	G	5.08E-07	0.44	0.088	0.045	0.058
rs1534430	T	C	9.40E-08	-0.09	0.017	-0.003	0.014
rs17490285	G	A	9.26E-07	-0.18	0.038	-0.007	0.021
rs1887414	C	T	1.97E-07	0.12	0.022	-0.012	0.018
rs2111485	G	A	4.19E-06	0.08	0.017	0.004	0.013
rs229526	C	G	1.93E-06	0.10	0.022	-0.006	0.016
rs2476601	G	A	3.49E-45	-0.33	0.023	-0.002	0.021
rs28367132	A	G	1.21E-07	-0.14	0.026	-0.007	0.017
rs28450758	A	C	4.17E-06	0.14	0.031	-0.018	0.025
rs3184504	C	T	1.40E-25	-0.18	0.017	0.018	0.013
rs34536443	C	G	2.47E-07	-0.25	0.049	-0.013	0.033
rs34636506	G	A	1.92E-14	-0.13	0.018	-0.009	0.014
rs61830417	G	A	3.59E-06	0.27	0.059	-0.021	0.046
rs62076103	G	A	1.23E-07	-0.19	0.035	-0.012	0.026
rs6448432	A	G	1.75E-06	0.09	0.019	-0.016	0.014
rs6501550	T	C	1.42E-06	-0.18	0.037	-0.020	0.040
rs7030280	T	C	6.44E-31	0.21	0.018	-0.003	0.014
rs72808590	C	T	1.55E-06	-0.37	0.077	0.066	0.048

Supplementary Table 2. Details of Hashimoto's Thyroiditis predicting SNPs with inflammatory proteins from IEU Open GWAS (with SNPs reaching $P < 5 \times 10^{-6}$).

Inflammatory proteins				Hashimoto's Thyroiditis			
SNP	Effect allele	Other allele	P value	Beta	Standard Error	log(OR)	Standard Error
rs73165154	G	A	3.76E-06	-0.15	0.033	-0.016	0.024
rs73192661	T	C	1.45E-09	-0.10	0.017	-0.013	0.014
rs7335748	G	A	2.41E-06	0.38	0.080	0.020	0.046
rs766932	T	A	4.96E-07	0.10	0.019	0.034	0.015
rs7767668	T	C	9.60E-07	-0.09	0.019	-0.005	0.013
rs7861040	C	G	9.08E-07	0.08	0.017	-0.019	0.013
rs79219789	C	G	2.84E-06	-0.15	0.033	-0.046	0.035
rs8084503	T	C	1.97E-07	0.14	0.027	-0.015	0.021
rs847803	G	A	7.91E-07	0.09	0.018	0.015	0.014
rs9357119	T	G	3.65E-14	-0.15	0.020	-0.002	0.015
rs9497965	T	C	1.71E-06	0.08	0.017	-0.009	0.013
**IL-2 (F-statistic = 38.11, R2=0.44%)**							
rs10424978	A	C	8.97E-07	-0.08	0.017	-0.014	0.014
rs10494077	A	G	9.20E-10	0.12	0.020	0.016	0.015
rs10786679	A	G	1.87E-06	-0.09	0.018	0.024	0.015
rs111358861	G	A	3.30E-06	1.82	0.391	-0.111	0.107
rs1115587	A	G	1.82E-07	-0.10	0.018	0.006	0.014
rs11211645	G	A	9.33E-08	0.09	0.017	-0.013	0.013
rs113075322	G	C	7.18E-07	-0.14	0.028	-0.077	0.036
rs11611029	T	C	1.16E-08	-0.10	0.017	-0.022	0.014
rs117167029	C	G	8.05E-07	2.99	0.606	0.034	0.111
rs117369326	A	G	3.55E-06	0.24	0.051	0.007	0.032
rs118075239	C	T	3.45E-06	0.38	0.083	-0.026	0.051
rs11822254	G	A	3.52E-06	-0.08	0.017	-0.005	0.014
rs11889341	T	C	6.64E-11	0.12	0.019	0.020	0.015
rs1317983	C	T	1.65E-07	0.09	0.018	-0.001	0.015
rs13249705	G	A	3.78E-07	-0.09	0.018	-0.002	0.016
rs140111921	A	G	1.34E-06	-0.16	0.034	-0.027	0.033
rs150619443	C	G	5.08E-07	0.44	0.088	0.005	0.058
rs1534430	T	C	9.40E-08	-0.09	0.017	0.009	0.014
rs17490285	G	A	9.26E-07	-0.18	0.038	-0.019	0.021

Supplementary Table 2. Details of Hashimoto's Thyroiditis predicting SNPs with inflammatory proteins from IEU Open GWAS (with SNPs reaching $P < 5 \times 10^{-6}$).

Inflammatory proteins				Hashimoto's Thyroiditis			
SNP	Effect allele	Other allele	P value	Beta	Standard Error	log(OR)	Standard Error
rs1887414	C	T	1.97E-07	0.12	0.022	-0.009	0.018
rs2111485	G	A	4.19E-06	0.08	0.017	-0.013	0.013
rs229526	C	G	1.93E-06	0.10	0.022	0.011	0.016
rs2476601	G	A	3.49E-45	-0.33	0.023	0.003	0.021
rs28367132	A	G	1.21E-07	-0.14	0.026	-0.016	0.017
rs28450758	A	C	4.17E-06	0.14	0.031	0.024	0.025
rs3184504	C	T	1.40E-25	-0.18	0.017	-0.025	0.013
rs34536443	C	G	2.47E-07	-0.25	0.049	-0.059	0.033
rs34636506	G	A	1.92E-14	-0.13	0.018	-0.010	0.014
rs61830417	G	A	3.59E-06	0.27	0.059	-0.020	0.045
rs62076103	G	A	1.23E-07	-0.19	0.035	-0.010	0.026
rs6448432	A	G	1.75E-06	0.09	0.019	-0.007	0.014
rs6501550	T	C	1.42E-06	-0.18	0.037	0.050	0.040
rs7030280	T	C	6.44E-31	0.21	0.018	-0.003	0.014
rs72808590	C	T	1.55E-06	-0.37	0.077	0.065	0.048
rs73165154	G	A	3.76E-06	-0.15	0.033	-0.038	0.024
rs73192661	T	C	1.45E-09	-0.10	0.017	-0.014	0.014
rs7335748	G	A	2.41E-06	0.38	0.080	0.058	0.046
rs766932	T	A	4.96E-07	0.10	0.019	0.014	0.014
rs7767668	T	C	9.60E-07	-0.09	0.019	0.013	0.013
rs7861040	C	G	9.08E-07	0.08	0.017	-0.001	0.013
rs79219789	C	G	2.84E-06	-0.15	0.033	0.019	0.035
rs8084503	T	C	1.97E-07	0.14	0.027	-0.017	0.021
rs847803	G	A	7.91E-07	0.09	0.018	0.000	0.014
rs9271365	G	T	1.44E-39	0.25	0.019	-0.016	0.013
rs9357119	T	G	3.65E-14	-0.15	0.020	0.019	0.015
rs9497965	T	C	1.71E-06	0.08	0.017	-0.018	0.013
rs10424978	A	C	8.97E-07	-0.08	0.017	0.001	0.014
rs10494077	A	G	9.20E-10	0.12	0.020	0.017	0.015
rs10786679	A	G	1.87E-06	-0.09	0.018	0.010	0.015
rs111358861	G	A	3.30E-06	1.82	0.391	0.207	0.107

Supplementary Table 2. Details of Hashimoto's Thyroiditis predicting SNPs with inflammatory proteins from IEU Open GWAS (with SNPs reaching $P < 5 \times 10^{-6}$).

Inflammatory proteins				Hashimoto's Thyroiditis			
SNP	Effect allele	Other allele	P value	Beta	Standard Error	log(OR)	Standard Error
rs1115587	A	G	1.82E-07	-0.10	0.018	0.017	0.014
rs11211645	G	A	9.33E-08	0.09	0.017	-0.022	0.013
rs113075322	G	C	7.18E-07	-0.14	0.028	0.000	0.036
rs11611029	T	C	1.16E-08	-0.10	0.017	-0.002	0.014
rs117167029	C	G	8.05E-07	2.99	0.606	-0.069	0.111
rs117369326	A	G	3.55E-06	0.24	0.051	0.006	0.032
rs118075239	C	T	3.45E-06	0.38	0.083	0.073	0.051
rs11822254	G	A	3.52E-06	-0.08	0.017	-0.010	0.014
rs11889341	T	C	6.64E-11	0.12	0.019	-0.007	0.015
rs1317983	C	T	1.65E-07	0.09	0.018	-0.012	0.015
rs13249705	G	A	3.78E-07	-0.09	0.018	-0.008	0.016
rs140111921	A	G	1.34E-06	-0.16	0.034	-0.051	0.033
rs150619443	C	G	5.08E-07	0.44	0.088	-0.057	0.057
rs1534430	T	C	9.40E-08	-0.09	0.017	0.000	0.014
rs17490285	G	A	9.26E-07	-0.18	0.038	-0.009	0.021
rs1887414	C	T	1.97E-07	0.12	0.022	-0.012	0.018
rs2111485	G	A	4.19E-06	0.08	0.017	0.015	0.013
rs229526	C	G	1.93E-06	0.10	0.022	-0.014	0.016
rs2476601	G	A	3.49E-45	-0.33	0.023	0.020	0.020
rs28367132	A	G	1.21E-07	-0.14	0.026	-0.018	0.017
rs28450758	A	C	4.17E-06	0.14	0.031	-0.004	0.024
rs3184504	C	T	1.40E-25	-0.18	0.017	-0.009	0.013
rs34536443	C	G	2.47E-07	-0.25	0.049	0.004	0.033
rs34636506	G	A	1.92E-14	-0.13	0.018	0.000	0.013
rs61830417	G	A	3.59E-06	0.27	0.059	-0.009	0.045
rs62076103	G	A	1.23E-07	-0.19	0.035	0.002	0.026
rs6448432	A	G	1.75E-06	0.09	0.019	-0.015	0.014
rs6501550	T	C	1.42E-06	-0.18	0.037	0.012	0.040
rs7030280	T	C	6.44E-31	0.21	0.018	-0.003	0.014
rs72808590	C	T	1.55E-06	-0.37	0.077	0.060	0.047
rs73165154	G	A	3.76E-06	-0.15	0.033	-0.013	0.024

Supplementary Table 2. Details of Hashimoto's Thyroiditis predicting SNPs with inflammatory proteins from IEU Open GWAS (with SNPs reaching $P < 5 \times 10^{-6}$).

Inflammatory proteins				Hashimoto's Thyroiditis			
SNP	Effect allele	Other allele	P value	Beta	Standard Error	log(OR)	Standard Error
rs73192661	T	C	1.45E-09	-0.10	0.017	-0.006	0.014
rs7335748	G	A	2.41E-06	0.38	0.080	0.041	0.045
rs766932	T	A	4.96E-07	0.10	0.019	-0.006	0.014
rs7767668	T	C	9.60E-07	-0.09	0.019	0.003	0.013
rs7861040	C	G	9.08E-07	0.08	0.017	-0.010	0.013
rs79219789	C	G	2.84E-06	-0.15	0.033	-0.010	0.034
rs8084503	T	C	1.97E-07	0.14	0.027	-0.009	0.020
rs847803	G	A	7.91E-07	0.09	0.018	0.014	0.014
rs9271365	G	T	1.44E-39	0.25	0.019	0.003	0.013
rs9357119	T	G	3.65E-14	-0.15	0.020	0.005	0.015
rs9497965	T	C	1.71E-06	0.08	0.017	-0.004	0.013
**IL-20Rα (F-statistic = 38.11, R2=0.44%)**							
rs10424978	A	C	8.97E-07	-0.08	0.017	0.020	0.014
rs10494077	A	G	9.20E-10	0.12	0.020	0.018	0.016
rs10786679	A	G	1.87E-06	-0.09	0.018	-0.002	0.015
rs111358861	G	A	3.30E-06	1.82	0.391	0.166	0.107
rs1115587	A	G	1.82E-07	-0.10	0.018	0.006	0.014
rs11211645	G	A	9.33E-08	0.09	0.017	-0.001	0.013
rs113075322	G	C	7.18E-07	-0.14	0.028	-0.043	0.036
rs11611029	T	C	1.16E-08	-0.10	0.017	0.006	0.014
rs117167029	C	G	8.05E-07	2.99	0.606	0.077	0.111
rs117369326	A	G	3.55E-06	0.24	0.051	0.024	0.033
rs118075239	C	T	3.45E-06	0.38	0.083	-0.028	0.051
rs11822254	G	A	3.52E-06	-0.08	0.017	0.005	0.014
rs11889341	T	C	6.64E-11	0.12	0.019	0.023	0.016
rs1317983	C	T	1.65E-07	0.09	0.018	-0.003	0.015
rs13249705	G	A	3.78E-07	-0.09	0.018	0.012	0.016
rs140111921	A	G	1.34E-06	-0.16	0.034	-0.011	0.033
rs150619443	C	G	5.08E-07	0.44	0.088	0.008	0.058
rs1534430	T	C	9.40E-08	-0.09	0.017	-0.003	0.014
rs17490285	G	A	9.26E-07	-0.18	0.038	-0.004	0.021

Supplementary Table 2. Details of Hashimoto's Thyroiditis predicting SNPs with inflammatory proteins from IEU Open GWAS (with SNPs reaching $P < 5 \times 10^{-6}$).

Inflammatory proteins				Hashimoto's Thyroiditis			
SNP	Effect allele	Other allele	P value	Beta	Standard Error	log(OR)	Standard Error
rs1887414	C	T	1.97E-07	0.12	0.022	-0.034	0.019
rs2111485	G	A	4.19E-06	0.08	0.017	-0.009	0.013
rs229526	C	G	1.93E-06	0.10	0.022	0.002	0.016
rs2476601	G	A	3.49E-45	-0.33	0.023	0.031	0.021
rs28367132	A	G	1.21E-07	-0.14	0.026	-0.021	0.017
rs28450758	A	C	4.17E-06	0.14	0.031	-0.023	0.025
rs3184504	C	T	1.40E-25	-0.18	0.017	-0.029	0.013
rs34536443	C	G	2.47E-07	-0.25	0.049	-0.046	0.034
rs34636506	G	A	1.92E-14	-0.13	0.018	-0.011	0.014
rs61830417	G	A	3.59E-06	0.27	0.059	0.008	0.046
rs62076103	G	A	1.23E-07	-0.19	0.035	-0.072	0.026
rs6448432	A	G	1.75E-06	0.09	0.019	0.004	0.014
rs6501550	T	C	1.42E-06	-0.18	0.037	-0.047	0.040
rs7030280	T	C	6.44E-31	0.21	0.018	0.004	0.014
rs72808590	C	T	1.55E-06	-0.37	0.077	0.051	0.048
rs73165154	G	A	3.76E-06	-0.15	0.033	-0.016	0.024
rs73192661	T	C	1.45E-09	-0.10	0.017	-0.019	0.014
rs7335748	G	A	2.41E-06	0.38	0.080	0.032	0.046
rs766932	T	A	4.96E-07	0.10	0.019	0.025	0.015
rs7767668	T	C	9.60E-07	-0.09	0.019	-0.003	0.013
rs7861040	C	G	9.08E-07	0.08	0.017	-0.006	0.013
rs79219789	C	G	2.84E-06	-0.15	0.033	0.020	0.035
rs8084503	T	C	1.97E-07	0.14	0.027	-0.018	0.021
rs847803	G	A	7.91E-07	0.09	0.018	-0.004	0.014
rs9271365	G	T	1.44E-39	0.25	0.019	-0.004	0.013
rs9357119	T	G	3.65E-14	-0.15	0.020	0.009	0.015
rs9497965	T	C	1.71E-06	0.08	0.017	-0.045	0.013
**IL-22Rα1 (F-statistic = 38.11, R2=0.44%)**							
rs10424978	A	C	8.97E-07	-0.08	0.017	-0.005	0.014
rs10494077	A	G	9.20E-10	0.12	0.020	0.012	0.016
rs10786679	A	G	1.87E-06	-0.09	0.018	-0.016	0.015

Supplementary Table 2. Details of Hashimoto's Thyroiditis predicting SNPs with inflammatory proteins from IEU Open GWAS (with SNPs reaching $P < 5 \times 10^{-6}$).

Inflammatory proteins				Hashimoto's Thyroiditis			
SNP	Effect allele	Other allele	P value	Beta	Standard Error	log(OR)	Standard Error
rs111358861	G	A	3.30E-06	1.82	0.391	0.195	0.107
rs1115587	A	G	1.82E-07	-0.10	0.018	0.001	0.014
rs11211645	G	A	9.33E-08	0.09	0.017	0.008	0.014
rs113075322	G	C	7.18E-07	-0.14	0.028	0.020	0.036
rs11611029	T	C	1.16E-08	-0.10	0.017	0.024	0.014
rs117167029	C	G	8.05E-07	2.99	0.606	0.026	0.111
rs117369326	A	G	3.55E-06	0.24	0.051	-0.011	0.033
rs118075239	C	T	3.45E-06	0.38	0.083	0.023	0.051
rs11822254	G	A	3.52E-06	-0.08	0.017	0.006	0.014
rs11889341	T	C	6.64E-11	0.12	0.019	-0.006	0.016
rs1317983	C	T	1.65E-07	0.09	0.018	0.016	0.015
rs13249705	G	A	3.78E-07	-0.09	0.018	-0.007	0.016
rs140111921	A	G	1.34E-06	-0.16	0.034	-0.044	0.033
rs150619443	C	G	5.08E-07	0.44	0.088	-0.017	0.059
rs1534430	T	C	9.40E-08	-0.09	0.017	-0.003	0.014
rs17490285	G	A	9.26E-07	-0.18	0.038	-0.025	0.021
rs1887414	C	T	1.97E-07	0.12	0.022	-0.007	0.019
rs2111485	G	A	4.19E-06	0.08	0.017	-0.005	0.013
rs229526	C	G	1.93E-06	0.10	0.022	-0.004	0.017
rs2476601	G	A	3.49E-45	-0.33	0.023	-0.001	0.022
rs28367132	A	G	1.21E-07	-0.14	0.026	0.019	0.017
rs28450758	A	C	4.17E-06	0.14	0.031	-0.029	0.025
rs3184504	C	T	1.40E-25	-0.18	0.017	0.007	0.013
rs34536443	C	G	2.47E-07	-0.25	0.049	-0.034	0.034
rs34636506	G	A	1.92E-14	-0.13	0.018	-0.012	0.014
rs61830417	G	A	3.59E-06	0.27	0.059	0.030	0.046
rs62076103	G	A	1.23E-07	-0.19	0.035	-0.004	0.027
rs6448432	A	G	1.75E-06	0.09	0.019	-0.006	0.015
rs6501550	T	C	1.42E-06	-0.18	0.037	0.084	0.040
rs7030280	T	C	6.44E-31	0.21	0.018	0.024	0.014
rs72808590	C	T	1.55E-06	-0.37	0.077	0.063	0.049

Supplementary Table 2. Details of Hashimoto's Thyroiditis predicting SNPs with inflammatory proteins from IEU Open GWAS (with SNPs reaching $P < 5 \times 10^{-6}$).

Inflammatory proteins				Hashimoto's Thyroiditis			
SNP	Effect allele	Other allele	P value	Beta	Standard Error	log(OR)	Standard Error
rs73165154	G	A	3.76E-06	-0.15	0.033	0.042	0.024
rs73192661	T	C	1.45E-09	-0.10	0.017	-0.018	0.014
rs7335748	G	A	2.41E-06	0.38	0.080	0.029	0.047
rs766932	T	A	4.96E-07	0.10	0.019	0.011	0.015
rs7767668	T	C	9.60E-07	-0.09	0.019	0.018	0.014
rs7861040	C	G	9.08E-07	0.08	0.017	0.014	0.014
rs79219789	C	G	2.84E-06	-0.15	0.033	0.032	0.036
rs8084503	T	C	1.97E-07	0.14	0.027	0.026	0.021
rs847803	G	A	7.91E-07	0.09	0.018	0.019	0.014
rs9271365	G	T	1.44E-39	0.25	0.019	-0.006	0.014
rs9357119	T	G	3.65E-14	-0.15	0.020	0.010	0.015
rs9497965	T	C	1.71E-06	0.08	0.017	-0.022	0.014
**IL-24 (F-statistic = 38.11, R2=0.44%)**							
rs10424978	A	C	8.97E-07	-0.08	0.017	0.000	0.014
rs10494077	A	G	9.20E-10	0.12	0.020	-0.008	0.016
rs10786679	A	G	1.87E-06	-0.09	0.018	-0.025	0.015
rs111358861	G	A	3.30E-06	1.82	0.391	0.152	0.107
rs1115587	A	G	1.82E-07	-0.10	0.018	0.022	0.014
rs11211645	G	A	9.33E-08	0.09	0.017	0.013	0.014
rs113075322	G	C	7.18E-07	-0.14	0.028	0.064	0.036
rs11611029	T	C	1.16E-08	-0.10	0.017	-0.001	0.014
rs117167029	C	G	8.05E-07	2.99	0.606	-0.302	0.111
rs117369326	A	G	3.55E-06	0.24	0.051	-0.012	0.033
rs118075239	C	T	3.45E-06	0.38	0.083	0.007	0.051
rs11822254	G	A	3.52E-06	-0.08	0.017	0.001	0.014
rs11889341	T	C	6.64E-11	0.12	0.019	0.010	0.016
rs1317983	C	T	1.65E-07	0.09	0.018	0.001	0.015
rs13249705	G	A	3.78E-07	-0.09	0.018	-0.010	0.016
rs140111921	A	G	1.34E-06	-0.16	0.034	-0.028	0.033
rs150619443	C	G	5.08E-07	0.44	0.088	-0.038	0.059
rs1534430	T	C	9.40E-08	-0.09	0.017	0.015	0.014

Supplementary Table 2. Details of Hashimoto's Thyroiditis predicting SNPs with inflammatory proteins from IEU Open GWAS (with SNPs reaching $P < 5 \times 10^{-6}$).

Inflammatory proteins						Hashimoto's Thyroiditis	
SNP	Effect allele	Other allele	P value	Beta	Standard Error	log(OR)	Standard Error
rs17490285	G	A	9.26E-07	-0.18	0.038	0.043	0.021
rs1887414	C	T	1.97E-07	0.12	0.022	-0.031	0.019
rs2111485	G	A	4.19E-06	0.08	0.017	0.019	0.013
rs229526	C	G	1.93E-06	0.10	0.022	0.001	0.017
rs2476601	G	A	3.49E-45	-0.33	0.023	0.002	0.022
rs28367132	A	G	1.21E-07	-0.14	0.026	0.014	0.017
rs28450758	A	C	4.17E-06	0.14	0.031	-0.015	0.025
rs3184504	C	T	1.40E-25	-0.18	0.017	-0.045	0.013
rs34536443	C	G	2.47E-07	-0.25	0.049	-0.016	0.034
rs34636506	G	A	1.92E-14	-0.13	0.018	-0.020	0.014
rs61830417	G	A	3.59E-06	0.27	0.059	-0.017	0.046
rs62076103	G	A	1.23E-07	-0.19	0.035	-0.005	0.027
rs6448432	A	G	1.75E-06	0.09	0.019	0.020	0.015
rs6501550	T	C	1.42E-06	-0.18	0.037	-0.029	0.040
rs7030280	T	C	6.44E-31	0.21	0.018	-0.013	0.014
rs72808590	C	T	1.55E-06	-0.37	0.077	0.025	0.049
rs73165154	G	A	3.76E-06	-0.15	0.033	0.049	0.024
rs73192661	T	C	1.45E-09	-0.10	0.017	0.003	0.014
rs7335748	G	A	2.41E-06	0.38	0.080	0.026	0.047
rs766932	T	A	4.96E-07	0.10	0.019	-0.014	0.015
rs7767668	T	C	9.60E-07	-0.09	0.019	0.009	0.013
rs7861040	C	G	9.08E-07	0.08	0.017	-0.009	0.014
rs79219789	C	G	2.84E-06	-0.15	0.033	0.009	0.036
rs8084503	T	C	1.97E-07	0.14	0.027	-0.035	0.021
rs847803	G	A	7.91E-07	0.09	0.018	0.008	0.014
rs9271365	G	T	1.44E-39	0.25	0.019	0.011	0.013
rs9357119	T	G	3.65E-14	-0.15	0.020	-0.010	0.015
rs9497965	T	C	1.71E-06	0.08	0.017	0.009	0.014
**IL-2Rβ (F-statistic = 36.52, R2=0.42%)**							
rs10424978	A	C	8.97E-07	-0.08	0.017	-0.019	0.014
rs10494077	A	G	9.20E-10	0.12	0.020	0.016	0.015

Supplementary Table 2. Details of Hashimoto's Thyroiditis predicting SNPs with inflammatory proteins from IEU Open GWAS (with SNPs reaching $P < 5 \times 10^{-6}$).

Inflammatory proteins				Hashimoto's Thyroiditis			
SNP	Effect allele	Other allele	P value	Beta	Standard Error	log(OR)	Standard Error
rs10786679	A	G	1.87E-06	-0.09	0.018	0.003	0.015
rs111358861	G	A	3.30E-06	1.82	0.391	0.145	0.107
rs1115587	A	G	1.82E-07	-0.10	0.018	0.020	0.014
rs11211645	G	A	9.33E-08	0.09	0.017	-0.006	0.013
rs113075322	G	C	7.18E-07	-0.14	0.028	-0.073	0.036
rs11611029	T	C	1.16E-08	-0.10	0.017	0.015	0.014
rs117167029	C	G	8.05E-07	2.99	0.606	-0.040	0.111
rs117369326	A	G	3.55E-06	0.24	0.051	-0.030	0.032
rs118075239	C	T	3.45E-06	0.38	0.083	-0.089	0.051
rs11822254	G	A	3.52E-06	-0.08	0.017	-0.001	0.014
rs11889341	T	C	6.64E-11	0.12	0.019	-0.004	0.015
rs1317983	C	T	1.65E-07	0.09	0.018	0.001	0.015
rs13249705	G	A	3.78E-07	-0.09	0.018	-0.042	0.016
rs140111921	A	G	1.34E-06	-0.16	0.034	0.012	0.033
rs150619443	C	G	5.08E-07	0.44	0.088	-0.031	0.057
rs1534430	T	C	9.40E-08	-0.09	0.017	0.018	0.014
rs17490285	G	A	9.26E-07	-0.18	0.038	0.015	0.021
rs1887414	C	T	1.97E-07	0.12	0.022	-0.012	0.018
rs2111485	G	A	4.19E-06	0.08	0.017	0.004	0.013
rs229526	C	G	1.93E-06	0.10	0.022	-0.019	0.016
rs2476601	G	A	3.49E-45	-0.33	0.023	0.010	0.021
rs28367132	A	G	1.21E-07	-0.14	0.026	-0.016	0.017
rs28450758	A	C	4.17E-06	0.14	0.031	-0.002	0.025
rs34536443	C	G	2.47E-07	-0.25	0.049	-0.055	0.033
rs34636506	G	A	1.92E-14	-0.13	0.018	0.006	0.014
rs61830417	G	A	3.59E-06	0.27	0.059	0.095	0.045
rs62076103	G	A	1.23E-07	-0.19	0.035	-0.009	0.026
rs6448432	A	G	1.75E-06	0.09	0.019	0.001	0.014
rs6501550	T	C	1.42E-06	-0.18	0.037	-0.027	0.040
rs7030280	T	C	6.44E-31	0.21	0.018	-0.011	0.014
rs72808590	C	T	1.55E-06	-0.37	0.077	0.130	0.048

Supplementary Table 2. Details of Hashimoto's Thyroiditis predicting SNPs with inflammatory proteins from IEU Open GWAS (with SNPs reaching $P < 5 \times 10^{-6}$).

Inflammatory proteins				Hashimoto's Thyroiditis			
SNP	Effect allele	Other allele	P value	Beta	Standard Error	log(OR)	Standard Error
rs73165154	G	A	3.76E-06	-0.15	0.033	0.013	0.024
rs73192661	T	C	1.45E-09	-0.10	0.017	0.009	0.014
rs7335748	G	A	2.41E-06	0.38	0.080	-0.015	0.046
rs766932	T	A	4.96E-07	0.10	0.019	-0.018	0.014
rs7767668	T	C	9.60E-07	-0.09	0.019	-0.003	0.013
rs7861040	C	G	9.08E-07	0.08	0.017	-0.009	0.013
rs79219789	C	G	2.84E-06	-0.15	0.033	0.034	0.035
rs8084503	T	C	1.97E-07	0.14	0.027	0.002	0.021
rs847803	G	A	7.91E-07	0.09	0.018	0.010	0.014
rs9271365	G	T	1.44E-39	0.25	0.019	-0.012	0.013
rs9357119	T	G	3.65E-14	-0.15	0.020	0.001	0.015
rs9497965	T	C	1.71E-06	0.08	0.017	-0.006	0.013
**IL-33 (F-statistic = 38.11, R2=0.44%)**							
rs10424978	A	C	8.97E-07	-0.08	0.017	0.015	0.014
rs10494077	A	G	9.20E-10	0.12	0.020	-0.021	0.015
rs10786679	A	G	1.87E-06	-0.09	0.018	-0.041	0.015
rs111358861	G	A	3.30E-06	1.82	0.391	-0.170	0.107
rs1115587	A	G	1.82E-07	-0.10	0.018	0.047	0.014
rs11211645	G	A	9.33E-08	0.09	0.017	-0.004	0.013
rs113075322	G	C	7.18E-07	-0.14	0.028	-0.048	0.036
rs11611029	T	C	1.16E-08	-0.10	0.017	-0.003	0.014
rs117167029	C	G	8.05E-07	2.99	0.606	-0.081	0.111
rs117369326	A	G	3.55E-06	0.24	0.051	0.017	0.032
rs118075239	C	T	3.45E-06	0.38	0.083	-0.079	0.051
rs11822254	G	A	3.52E-06	-0.08	0.017	0.010	0.014
rs11889341	T	C	6.64E-11	0.12	0.019	0.023	0.015
rs1317983	C	T	1.65E-07	0.09	0.018	-0.007	0.015
rs13249705	G	A	3.78E-07	-0.09	0.018	0.012	0.016
rs140111921	A	G	1.34E-06	-0.16	0.034	-0.020	0.033
rs150619443	C	G	5.08E-07	0.44	0.088	0.016	0.057
rs1534430	T	C	9.40E-08	-0.09	0.017	-0.003	0.014

Supplementary Table 2. Details of Hashimoto's Thyroiditis predicting SNPs with inflammatory proteins from IEU Open GWAS (with SNPs reaching $P < 5 \times 10^{-6}$).

Inflammatory proteins				Hashimoto's Thyroiditis			
SNP	Effect allele	Other allele	P value	Beta	Standard Error	log(OR)	Standard Error
rs17490285	G	A	9.26E-07	-0.18	0.038	-0.006	0.021
rs1887414	C	T	1.97E-07	0.12	0.022	-0.044	0.018
rs2111485	G	A	4.19E-06	0.08	0.017	-0.030	0.013
rs229526	C	G	1.93E-06	0.10	0.022	-0.013	0.016
rs2476601	G	A	3.49E-45	-0.33	0.023	-0.020	0.021
rs28367132	A	G	1.21E-07	-0.14	0.026	0.002	0.017
rs28450758	A	C	4.17E-06	0.14	0.031	0.010	0.025
rs3184504	C	T	1.40E-25	-0.18	0.017	-0.015	0.013
rs34536443	C	G	2.47E-07	-0.25	0.049	-0.048	0.033
rs34636506	G	A	1.92E-14	-0.13	0.018	0.002	0.014
rs61830417	G	A	3.59E-06	0.27	0.059	0.013	0.045
rs62076103	G	A	1.23E-07	-0.19	0.035	-0.030	0.026
rs6448432	A	G	1.75E-06	0.09	0.019	-0.004	0.014
rs6501550	T	C	1.42E-06	-0.18	0.037	-0.019	0.040
rs7030280	T	C	6.44E-31	0.21	0.018	-0.014	0.014
rs72808590	C	T	1.55E-06	-0.37	0.077	0.035	0.048
rs73165154	G	A	3.76E-06	-0.15	0.033	-0.017	0.024
rs73192661	T	C	1.45E-09	-0.10	0.017	-0.019	0.014
rs7335748	G	A	2.41E-06	0.38	0.080	-0.009	0.046
rs766932	T	A	4.96E-07	0.10	0.019	0.010	0.014
rs7767668	T	C	9.60E-07	-0.09	0.019	0.000	0.013
rs7861040	C	G	9.08E-07	0.08	0.017	-0.005	0.013
rs79219789	C	G	2.84E-06	-0.15	0.033	0.024	0.035
rs8084503	T	C	1.97E-07	0.14	0.027	-0.009	0.021
rs847803	G	A	7.91E-07	0.09	0.018	0.002	0.014
rs9271365	G	T	1.44E-39	0.25	0.019	-0.004	0.013
rs9357119	T	G	3.65E-14	-0.15	0.020	-0.020	0.015
rs9497965	T	C	1.71E-06	0.08	0.017	-0.005	0.013
**IL-4 (F-statistic = 38.11, R2=0.44%)**							
rs10424978	A	C	8.97E-07	-0.08	0.017	-0.004	0.014
rs10494077	A	G	9.20E-10	0.12	0.020	0.016	0.016

Supplementary Table 2. Details of Hashimoto's Thyroiditis predicting SNPs with inflammatory proteins from IEU Open GWAS (with SNPs reaching $P < 5 \times 10^{-6}$).

Inflammatory proteins				Hashimoto's Thyroiditis			
SNP	Effect allele	Other allele	P value	Beta	Standard Error	log(OR)	Standard Error
rs10786679	A	G	1.87E-06	-0.09	0.018	-0.013	0.015
rs111358861	G	A	3.30E-06	1.82	0.391	-0.036	0.107
rs1115587	A	G	1.82E-07	-0.10	0.018	0.032	0.014
rs11211645	G	A	9.33E-08	0.09	0.017	0.014	0.013
rs113075322	G	C	7.18E-07	-0.14	0.028	0.047	0.036
rs11611029	T	C	1.16E-08	-0.10	0.017	-0.037	0.014
rs117167029	C	G	8.05E-07	2.99	0.606	-0.001	0.111
rs117369326	A	G	3.55E-06	0.24	0.051	-0.003	0.033
rs118075239	C	T	3.45E-06	0.38	0.083	0.050	0.051
rs11822254	G	A	3.52E-06	-0.08	0.017	0.012	0.014
rs11889341	T	C	6.64E-11	0.12	0.019	0.015	0.016
rs1317983	C	T	1.65E-07	0.09	0.018	0.014	0.015
rs13249705	G	A	3.78E-07	-0.09	0.018	-0.037	0.016
rs140111921	A	G	1.34E-06	-0.16	0.034	-0.025	0.033
rs150619443	C	G	5.08E-07	0.44	0.088	-0.015	0.059
rs1534430	T	C	9.40E-08	-0.09	0.017	0.002	0.014
rs17490285	G	A	9.26E-07	-0.18	0.038	0.019	0.021
rs1887414	C	T	1.97E-07	0.12	0.022	-0.028	0.019
rs2111485	G	A	4.19E-06	0.08	0.017	0.018	0.013
rs229526	C	G	1.93E-06	0.10	0.022	0.002	0.017
rs2476601	G	A	3.49E-45	-0.33	0.023	0.025	0.022
rs28367132	A	G	1.21E-07	-0.14	0.026	0.006	0.017
rs28450758	A	C	4.17E-06	0.14	0.031	0.008	0.025
rs3184504	C	T	1.40E-25	-0.18	0.017	-0.027	0.013
rs34536443	C	G	2.47E-07	-0.25	0.049	-0.044	0.034
rs34636506	G	A	1.92E-14	-0.13	0.018	-0.026	0.014
rs61830417	G	A	3.59E-06	0.27	0.059	0.077	0.046
rs62076103	G	A	1.23E-07	-0.19	0.035	-0.041	0.027
rs6448432	A	G	1.75E-06	0.09	0.019	-0.012	0.015
rs6501550	T	C	1.42E-06	-0.18	0.037	-0.050	0.040
rs7030280	T	C	6.44E-31	0.21	0.018	0.007	0.014

Supplementary Table 2. Details of Hashimoto's Thyroiditis predicting SNPs with inflammatory proteins from IEU Open GWAS (with SNPs reaching $P < 5 \times 10^{-6}$).

Inflammatory proteins				Hashimoto's Thyroiditis			
SNP	Effect allele	Other allele	P value	Beta	Standard Error	log(OR)	Standard Error
rs72808590	C	T	1.55E-06	-0.37	0.077	-0.016	0.049
rs73165154	G	A	3.76E-06	-0.15	0.033	-0.032	0.024
rs73192661	T	C	1.45E-09	-0.10	0.017	0.012	0.014
rs7335748	G	A	2.41E-06	0.38	0.080	-0.004	0.047
rs766932	T	A	4.96E-07	0.10	0.019	0.009	0.015
rs7767668	T	C	9.60E-07	-0.09	0.019	-0.003	0.013
rs7861040	C	G	9.08E-07	0.08	0.017	0.013	0.014
rs79219789	C	G	2.84E-06	-0.15	0.033	-0.029	0.036
rs8084503	T	C	1.97E-07	0.14	0.027	-0.018	0.021
rs847803	G	A	7.91E-07	0.09	0.018	0.024	0.014
rs9271365	G	T	1.44E-39	0.25	0.019	-0.004	0.013
rs9357119	T	G	3.65E-14	-0.15	0.020	-0.004	0.015
rs9497965	T	C	1.71E-06	0.08	0.017	0.005	0.014
**IL-5 (F-statistic = 38.11, R2=0.44%)**							
rs10424978	A	C	8.97E-07	-0.08	0.017	0.024	0.014
rs10494077	A	G	9.20E-10	0.12	0.020	0.019	0.016
rs10786679	A	G	1.87E-06	-0.09	0.018	0.001	0.015
rs111358861	G	A	3.30E-06	1.82	0.391	-0.135	0.107
rs1115587	A	G	1.82E-07	-0.10	0.018	0.013	0.014
rs11211645	G	A	9.33E-08	0.09	0.017	0.025	0.013
rs113075322	G	C	7.18E-07	-0.14	0.028	-0.025	0.036
rs11611029	T	C	1.16E-08	-0.10	0.017	0.013	0.014
rs117167029	C	G	8.05E-07	2.99	0.606	-0.002	0.111
rs117369326	A	G	3.55E-06	0.24	0.051	0.010	0.033
rs118075239	C	T	3.45E-06	0.38	0.083	0.000	0.051
rs11822254	G	A	3.52E-06	-0.08	0.017	-0.017	0.014
rs11889341	T	C	6.64E-11	0.12	0.019	0.000	0.016
rs1317983	C	T	1.65E-07	0.09	0.018	-0.011	0.015
rs13249705	G	A	3.78E-07	-0.09	0.018	0.025	0.016
rs140111921	A	G	1.34E-06	-0.16	0.034	-0.018	0.033
rs150619443	C	G	5.08E-07	0.44	0.088	0.032	0.059

Supplementary Table 2. Details of Hashimoto's Thyroiditis predicting SNPs with inflammatory proteins from IEU Open GWAS (with SNPs reaching $P < 5 \times 10^{-6}$).

Inflammatory proteins				Hashimoto's Thyroiditis			
SNP	Effect allele	Other allele	P value	Beta	Standard Error	log(OR)	Standard Error
rs1534430	T	C	9.40E-08	-0.09	0.017	0.015	0.014
rs17490285	G	A	9.26E-07	-0.18	0.038	-0.001	0.021
rs1887414	C	T	1.97E-07	0.12	0.022	0.005	0.019
rs2111485	G	A	4.19E-06	0.08	0.017	0.002	0.013
rs229526	C	G	1.93E-06	0.10	0.022	-0.004	0.017
rs2476601	G	A	3.49E-45	-0.33	0.023	0.069	0.022
rs28367132	A	G	1.21E-07	-0.14	0.026	-0.009	0.017
rs28450758	A	C	4.17E-06	0.14	0.031	0.007	0.025
rs3184504	C	T	1.40E-25	-0.18	0.017	0.013	0.013
rs34536443	C	G	2.47E-07	-0.25	0.049	-0.015	0.034
rs34636506	G	A	1.92E-14	-0.13	0.018	-0.012	0.014
rs61830417	G	A	3.59E-06	0.27	0.059	0.080	0.046
rs62076103	G	A	1.23E-07	-0.19	0.035	-0.022	0.027
rs6448432	A	G	1.75E-06	0.09	0.019	-0.004	0.015
rs6501550	T	C	1.42E-06	-0.18	0.037	0.009	0.040
rs7030280	T	C	6.44E-31	0.21	0.018	-0.006	0.014
rs72808590	C	T	1.55E-06	-0.37	0.077	-0.085	0.049
rs73165154	G	A	3.76E-06	-0.15	0.033	-0.009	0.024
rs73192661	T	C	1.45E-09	-0.10	0.017	-0.015	0.014
rs7335748	G	A	2.41E-06	0.38	0.080	-0.056	0.047
rs766932	T	A	4.96E-07	0.10	0.019	-0.001	0.015
rs7767668	T	C	9.60E-07	-0.09	0.019	0.000	0.013
rs7861040	C	G	9.08E-07	0.08	0.017	-0.014	0.014
rs79219789	C	G	2.84E-06	-0.15	0.033	-0.026	0.036
rs8084503	T	C	1.97E-07	0.14	0.027	-0.019	0.021
rs847803	G	A	7.91E-07	0.09	0.018	0.010	0.014
rs9271365	G	T	1.44E-39	0.25	0.019	0.035	0.013
rs9357119	T	G	3.65E-14	-0.15	0.020	0.016	0.015
rs9497965	T	C	1.71E-06	0.08	0.017	-0.017	0.014
**IL-6 (F-statistic = 38.11, R2=0.44%)**							
rs10424978	A	C	8.97E-07	-0.08	0.017	-0.009	0.012

Supplementary Table 2. Details of Hashimoto's Thyroiditis predicting SNPs with inflammatory proteins from IEU Open GWAS (with SNPs reaching $P < 5 \times 10^{-6}$).

Inflammatory proteins				Hashimoto's Thyroiditis			
SNP	Effect allele	Other allele	P value	Beta	Standard Error	log(OR)	Standard Error
rs10494077	A	G	9.20E-10	0.12	0.020	0.004	0.014
rs10786679	A	G	1.87E-06	-0.09	0.018	0.017	0.013
rs111358861	G	A	3.30E-06	1.82	0.391	-0.033	0.106
rs1115587	A	G	1.82E-07	-0.10	0.018	0.001	0.012
rs11211645	G	A	9.33E-08	0.09	0.017	-0.001	0.012
rs113075322	G	C	7.18E-07	-0.14	0.028	0.000	0.033
rs11611029	T	C	1.16E-08	-0.10	0.017	0.014	0.012
rs117167029	C	G	8.05E-07	2.99	0.606	-0.013	0.088
rs117369326	A	G	3.55E-06	0.24	0.051	-0.001	0.029
rs118075239	C	T	3.45E-06	0.38	0.083	-0.023	0.047
rs11822254	G	A	3.52E-06	-0.08	0.017	-0.005	0.012
rs11889341	T	C	6.64E-11	0.12	0.019	0.014	0.014
rs1317983	C	T	1.65E-07	0.09	0.018	0.008	0.013
rs13249705	G	A	3.78E-07	-0.09	0.018	-0.002	0.014
rs140111921	A	G	1.34E-06	-0.16	0.034	-0.014	0.030
rs150619443	C	G	5.08E-07	0.44	0.088	-0.071	0.055
rs1534430	T	C	9.40E-08	-0.09	0.017	0.020	0.012
rs17490285	G	A	9.26E-07	-0.18	0.038	-0.017	0.019
rs1887414	C	T	1.97E-07	0.12	0.022	0.010	0.017
rs2111485	G	A	4.19E-06	0.08	0.017	0.011	0.012
rs229526	C	G	1.93E-06	0.10	0.022	0.005	0.015
rs2476601	G	A	3.49E-45	-0.33	0.023	0.011	0.019
rs28367132	A	G	1.21E-07	-0.14	0.026	-0.020	0.015
rs28450758	A	C	4.17E-06	0.14	0.031	0.013	0.023
rs3184504	C	T	1.40E-25	-0.18	0.017	0.011	0.013
rs34536443	C	G	2.47E-07	-0.25	0.049	-0.024	0.031
rs34636506	G	A	1.92E-14	-0.13	0.018	-0.003	0.012
rs61830417	G	A	3.59E-06	0.27	0.059	0.086	0.042
rs62076103	G	A	1.23E-07	-0.19	0.035	-0.003	0.025
rs6448432	A	G	1.75E-06	0.09	0.019	0.008	0.013
rs6501550	T	C	1.42E-06	-0.18	0.037	0.023	0.034

Supplementary Table 2. Details of Hashimoto's Thyroiditis predicting SNPs with inflammatory proteins from IEU Open GWAS (with SNPs reaching $P < 5 \times 10^{-6}$).

Inflammatory proteins				Hashimoto's Thyroiditis			
SNP	Effect allele	Other allele	P value	Beta	Standard Error	log(OR)	Standard Error
rs7030280	T	C	6.44E-31	0.21	0.018	-0.002	0.013
rs72808590	C	T	1.55E-06	-0.37	0.077	-0.021	0.045
rs73165154	G	A	3.76E-06	-0.15	0.033	-0.009	0.022
rs73192661	T	C	1.45E-09	-0.10	0.017	0.017	0.012
rs7335748	G	A	2.41E-06	0.38	0.080	0.007	0.041
rs766932	T	A	4.96E-07	0.10	0.019	0.007	0.013
rs7767668	T	C	9.60E-07	-0.09	0.019	0.001	0.012
rs7861040	C	G	9.08E-07	0.08	0.017	0.002	0.012
rs79219789	C	G	2.84E-06	-0.15	0.033	0.031	0.031
rs8084503	T	C	1.97E-07	0.14	0.027	0.016	0.018
rs847803	G	A	7.91E-07	0.09	0.018	-0.014	0.013
rs9271365	G	T	1.44E-39	0.25	0.019	0.008	0.012
rs9357119	T	G	3.65E-14	-0.15	0.020	0.011	0.013
rs9497965	T	C	1.71E-06	0.08	0.017	-0.013	0.012
**IL-7 (F-statistic = 38.11, R2=0.44%)**							
rs10424978	A	C	8.97E-07	-0.08	0.017	-0.023	0.012
rs10494077	A	G	9.20E-10	0.12	0.020	-0.007	0.014
rs10786679	A	G	1.87E-06	-0.09	0.018	-0.005	0.014
rs111358861	G	A	3.30E-06	1.82	0.391	-0.139	0.107
rs1115587	A	G	1.82E-07	-0.10	0.018	0.017	0.012
rs11211645	G	A	9.33E-08	0.09	0.017	-0.023	0.012
rs113075322	G	C	7.18E-07	-0.14	0.028	0.008	0.033
rs11611029	T	C	1.16E-08	-0.10	0.017	0.002	0.012
rs117167029	C	G	8.05E-07	2.99	0.606	0.039	0.091
rs117369326	A	G	3.55E-06	0.24	0.051	-0.015	0.029
rs118075239	C	T	3.45E-06	0.38	0.083	-0.051	0.047
rs11822254	G	A	3.52E-06	-0.08	0.017	0.005	0.012
rs11889341	T	C	6.64E-11	0.12	0.019	-0.003	0.014
rs1317983	C	T	1.65E-07	0.09	0.018	0.004	0.013
rs13249705	G	A	3.78E-07	-0.09	0.018	-0.011	0.014
rs140111921	A	G	1.34E-06	-0.16	0.034	0.000	0.031

Supplementary Table 2. Details of Hashimoto's Thyroiditis predicting SNPs with inflammatory proteins from IEU Open GWAS (with SNPs reaching $P < 5 \times 10^{-6}$).

Inflammatory proteins				Hashimoto's Thyroiditis			
SNP	Effect allele	Other allele	P value	Beta	Standard Error	log(OR)	Standard Error
rs150619443	C	G	5.08E-07	0.44	0.088	0.048	0.054
rs1534430	T	C	9.40E-08	-0.09	0.017	0.009	0.012
rs17490285	G	A	9.26E-07	-0.18	0.038	-0.006	0.019
rs1887414	C	T	1.97E-07	0.12	0.022	-0.020	0.017
rs2111485	G	A	4.19E-06	0.08	0.017	-0.006	0.012
rs229526	C	G	1.93E-06	0.10	0.022	0.010	0.015
rs2476601	G	A	3.49E-45	-0.33	0.023	0.022	0.019
rs28367132	A	G	1.21E-07	-0.14	0.026	-0.028	0.015
rs28450758	A	C	4.17E-06	0.14	0.031	-0.031	0.023
rs3184504	C	T	1.40E-25	-0.18	0.017	-0.007	0.013
rs34536443	C	G	2.47E-07	-0.25	0.049	0.003	0.030
rs34636506	G	A	1.92E-14	-0.13	0.018	0.008	0.012
rs61830417	G	A	3.59E-06	0.27	0.059	0.035	0.042
rs62076103	G	A	1.23E-07	-0.19	0.035	0.003	0.025
rs6448432	A	G	1.75E-06	0.09	0.019	0.018	0.013
rs6501550	T	C	1.42E-06	-0.18	0.037	0.033	0.035
rs7030280	T	C	6.44E-31	0.21	0.018	0.000	0.013
rs72808590	C	T	1.55E-06	-0.37	0.077	0.020	0.045
rs73165154	G	A	3.76E-06	-0.15	0.033	0.003	0.022
rs73192661	T	C	1.45E-09	-0.10	0.017	-0.015	0.012
rs7335748	G	A	2.41E-06	0.38	0.080	-0.002	0.041
rs766932	T	A	4.96E-07	0.10	0.019	-0.002	0.013
rs7767668	T	C	9.60E-07	-0.09	0.019	0.004	0.012
rs7861040	C	G	9.08E-07	0.08	0.017	-0.006	0.012
rs79219789	C	G	2.84E-06	-0.15	0.033	0.015	0.031
rs8084503	T	C	1.97E-07	0.14	0.027	-0.014	0.018
rs847803	G	A	7.91E-07	0.09	0.018	0.019	0.013
rs9271365	G	T	1.44E-39	0.25	0.019	0.005	0.012
rs9357119	T	G	3.65E-14	-0.15	0.020	0.005	0.013
rs9497965	T	C	1.71E-06	0.08	0.017	0.005	0.012
**IL-8 (F-statistic = 38.45, R2=0.44%)**							

Supplementary Table 2. Details of Hashimoto's Thyroiditis predicting SNPs with inflammatory proteins from IEU Open GWAS (with SNPs reaching $P < 5 \times 10^{-6}$).

Inflammatory proteins				Hashimoto's Thyroiditis			
SNP	Effect allele	Other allele	P value	Beta	Standard Error	log(OR)	Standard Error
rs10424978	A	C	8.97E-07	-0.08	0.017	-0.005	0.012
rs10494077	A	G	9.20E-10	0.12	0.020	0.000	0.014
rs111358861	G	A	3.30E-06	1.82	0.391	-0.048	0.107
rs1115587	A	G	1.82E-07	-0.10	0.018	0.012	0.012
rs11211645	G	A	9.33E-08	0.09	0.017	-0.025	0.012
rs113075322	G	C	7.18E-07	-0.14	0.028	0.002	0.033
rs11611029	T	C	1.16E-08	-0.10	0.017	0.006	0.012
rs117167029	C	G	8.05E-07	2.99	0.606	-0.022	0.090
rs117369326	A	G	3.55E-06	0.24	0.051	0.026	0.029
rs118075239	C	T	3.45E-06	0.38	0.083	0.072	0.047
rs11822254	G	A	3.52E-06	-0.08	0.017	0.013	0.012
rs11889341	T	C	6.64E-11	0.12	0.019	-0.014	0.014
rs1317983	C	T	1.65E-07	0.09	0.018	-0.014	0.013
rs13249705	G	A	3.78E-07	-0.09	0.018	-0.001	0.014
rs140111921	A	G	1.34E-06	-0.16	0.034	-0.068	0.030
rs150619443	C	G	5.08E-07	0.44	0.088	-0.020	0.054
rs1534430	T	C	9.40E-08	-0.09	0.017	0.037	0.012
rs17490285	G	A	9.26E-07	-0.18	0.038	-0.009	0.019
rs1887414	C	T	1.97E-07	0.12	0.022	-0.027	0.017
rs2111485	G	A	4.19E-06	0.08	0.017	-0.008	0.012
rs229526	C	G	1.93E-06	0.10	0.022	0.003	0.015
rs2476601	G	A	3.49E-45	-0.33	0.023	-0.007	0.019
rs28367132	A	G	1.21E-07	-0.14	0.026	-0.027	0.015
rs28450758	A	C	4.17E-06	0.14	0.031	-0.046	0.023
rs3184504	C	T	1.40E-25	-0.18	0.017	-0.001	0.013
rs34536443	C	G	2.47E-07	-0.25	0.049	-0.012	0.031
rs34636506	G	A	1.92E-14	-0.13	0.018	-0.003	0.012
rs61830417	G	A	3.59E-06	0.27	0.059	0.075	0.041
rs62076103	G	A	1.23E-07	-0.19	0.035	-0.002	0.025
rs6448432	A	G	1.75E-06	0.09	0.019	0.026	0.013
rs6501550	T	C	1.42E-06	-0.18	0.037	0.039	0.034

Supplementary Table 2. Details of Hashimoto's Thyroiditis predicting SNPs with inflammatory proteins from IEU Open GWAS (with SNPs reaching $P < 5 \times 10^{-6}$).

Inflammatory proteins				Hashimoto's Thyroiditis			
SNP	Effect allele	Other allele	P value	Beta	Standard Error	log(OR)	Standard Error
rs7030280	T	C	6.44E-31	0.21	0.018	0.002	0.013
rs72808590	C	T	1.55E-06	-0.37	0.077	-0.044	0.045
rs73165154	G	A	3.76E-06	-0.15	0.033	-0.039	0.022
rs73192661	T	C	1.45E-09	-0.10	0.017	-0.016	0.012
rs7335748	G	A	2.41E-06	0.38	0.080	0.059	0.041
rs766932	T	A	4.96E-07	0.10	0.019	-0.011	0.013
rs7767668	T	C	9.60E-07	-0.09	0.019	0.013	0.012
rs7861040	C	G	9.08E-07	0.08	0.017	-0.008	0.012
rs79219789	C	G	2.84E-06	-0.15	0.033	0.021	0.031
rs8084503	T	C	1.97E-07	0.14	0.027	0.008	0.018
rs847803	G	A	7.91E-07	0.09	0.018	0.022	0.013
rs9271365	G	T	1.44E-39	0.25	0.019	-0.006	0.012
rs9357119	T	G	3.65E-14	-0.15	0.020	-0.013	0.013
rs9497965	T	C	1.71E-06	0.08	0.017	-0.010	0.012
**INF-γ (F-statistic = 38.11, R2=0.44%)**							
rs10424978	A	C	8.97E-07	-0.08	0.017	0.000	0.014
rs10494077	A	G	9.20E-10	0.12	0.020	0.001	0.015
rs10786679	A	G	1.87E-06	-0.09	0.018	-0.010	0.015
rs111358861	G	A	3.30E-06	1.82	0.391	0.071	0.107
rs1115587	A	G	1.82E-07	-0.10	0.018	0.031	0.014
rs11211645	G	A	9.33E-08	0.09	0.017	-0.017	0.013
rs113075322	G	C	7.18E-07	-0.14	0.028	-0.074	0.036
rs11611029	T	C	1.16E-08	-0.10	0.017	0.012	0.014
rs117167029	C	G	8.05E-07	2.99	0.606	-0.023	0.111
rs117369326	A	G	3.55E-06	0.24	0.051	0.027	0.032
rs118075239	C	T	3.45E-06	0.38	0.083	0.102	0.050
rs11822254	G	A	3.52E-06	-0.08	0.017	0.003	0.014
rs11889341	T	C	6.64E-11	0.12	0.019	0.026	0.015
rs1317983	C	T	1.65E-07	0.09	0.018	-0.010	0.015
rs13249705	G	A	3.78E-07	-0.09	0.018	-0.003	0.016
rs140111921	A	G	1.34E-06	-0.16	0.034	0.000	0.033

Supplementary Table 2. Details of Hashimoto's Thyroiditis predicting SNPs with inflammatory proteins from IEU Open GWAS (with SNPs reaching $P < 5 \times 10^{-6}$).

Inflammatory proteins						Hashimoto's Thyroiditis	
SNP	Effect allele	Other allele	P value	Beta	Standard Error	log(OR)	Standard Error
rs150619443	C	G	5.08E-07	0.44	0.088	0.041	0.057
rs1534430	T	C	9.40E-08	-0.09	0.017	0.009	0.014
rs17490285	G	A	9.26E-07	-0.18	0.038	0.016	0.021
rs1887414	C	T	1.97E-07	0.12	0.022	-0.013	0.018
rs2111485	G	A	4.19E-06	0.08	0.017	-0.012	0.013
rs229526	C	G	1.93E-06	0.10	0.022	-0.007	0.016
rs2476601	G	A	3.49E-45	-0.33	0.023	0.033	0.020
rs28367132	A	G	1.21E-07	-0.14	0.026	-0.011	0.017
rs28450758	A	C	4.17E-06	0.14	0.031	-0.046	0.024
rs3184504	C	T	1.40E-25	-0.18	0.017	-0.045	0.013
rs34536443	C	G	2.47E-07	-0.25	0.049	0.002	0.033
rs34636506	G	A	1.92E-14	-0.13	0.018	-0.019	0.013
rs61830417	G	A	3.59E-06	0.27	0.059	0.020	0.045
rs62076103	G	A	1.23E-07	-0.19	0.035	0.032	0.026
rs6448432	A	G	1.75E-06	0.09	0.019	0.003	0.014
rs6501550	T	C	1.42E-06	-0.18	0.037	0.050	0.040
rs7030280	T	C	6.44E-31	0.21	0.018	0.023	0.014
rs72808590	C	T	1.55E-06	-0.37	0.077	0.023	0.047
rs73165154	G	A	3.76E-06	-0.15	0.033	0.002	0.024
rs73192661	T	C	1.45E-09	-0.10	0.017	0.005	0.014
rs7335748	G	A	2.41E-06	0.38	0.080	0.017	0.045
rs766932	T	A	4.96E-07	0.10	0.019	0.005	0.014
rs7767668	T	C	9.60E-07	-0.09	0.019	-0.011	0.013
rs7861040	C	G	9.08E-07	0.08	0.017	0.001	0.013
rs79219789	C	G	2.84E-06	-0.15	0.033	-0.017	0.034
rs8084503	T	C	1.97E-07	0.14	0.027	0.005	0.020
rs847803	G	A	7.91E-07	0.09	0.018	0.003	0.014
rs9271365	G	T	1.44E-39	0.25	0.019	-0.002	0.013
rs9357119	T	G	3.65E-14	-0.15	0.020	0.003	0.015
rs9497965	T	C	1.71E-06	0.08	0.017	-0.011	0.013
**LAP TGF-β1 (F-statistic = 38.11, R2=0.44%)**							

Supplementary Table 2. Details of Hashimoto's Thyroiditis predicting SNPs with inflammatory proteins from IEU Open GWAS (with SNPs reaching $P < 5 \times 10^{-6}$).

Inflammatory proteins				Hashimoto's Thyroiditis			
SNP	Effect allele	Other allele	P value	Beta	Standard Error	log(OR)	Standard Error
rs10424978	A	C	8.97E-07	-0.08	0.017	-0.025	0.012
rs10494077	A	G	9.20E-10	0.12	0.020	-0.005	0.014
rs10786679	A	G	1.87E-06	-0.09	0.018	-0.006	0.013
rs111358861	G	A	3.30E-06	1.82	0.391	-0.086	0.107
rs1115587	A	G	1.82E-07	-0.10	0.018	0.017	0.012
rs11211645	G	A	9.33E-08	0.09	0.017	-0.027	0.012
rs113075322	G	C	7.18E-07	-0.14	0.028	0.000	0.033
rs11611029	T	C	1.16E-08	-0.10	0.017	-0.007	0.012
rs117167029	C	G	8.05E-07	2.99	0.606	-0.080	0.090
rs117369326	A	G	3.55E-06	0.24	0.051	0.026	0.029
rs118075239	C	T	3.45E-06	0.38	0.083	0.025	0.047
rs11822254	G	A	3.52E-06	-0.08	0.017	0.000	0.012
rs11889341	T	C	6.64E-11	0.12	0.019	-0.022	0.014
rs1317983	C	T	1.65E-07	0.09	0.018	0.001	0.013
rs13249705	G	A	3.78E-07	-0.09	0.018	0.007	0.014
rs140111921	A	G	1.34E-06	-0.16	0.034	0.000	0.030
rs150619443	C	G	5.08E-07	0.44	0.088	-0.053	0.054
rs1534430	T	C	9.40E-08	-0.09	0.017	0.000	0.012
rs17490285	G	A	9.26E-07	-0.18	0.038	-0.006	0.019
rs1887414	C	T	1.97E-07	0.12	0.022	-0.014	0.016
rs2111485	G	A	4.19E-06	0.08	0.017	-0.005	0.012
rs229526	C	G	1.93E-06	0.10	0.022	0.005	0.015
rs2476601	G	A	3.49E-45	-0.33	0.023	0.015	0.019
rs28367132	A	G	1.21E-07	-0.14	0.026	-0.026	0.015
rs28450758	A	C	4.17E-06	0.14	0.031	-0.023	0.022
rs3184504	C	T	1.40E-25	-0.18	0.017	-0.032	0.013
rs34536443	C	G	2.47E-07	-0.25	0.049	0.002	0.030
rs34636506	G	A	1.92E-14	-0.13	0.018	0.013	0.012
rs61830417	G	A	3.59E-06	0.27	0.059	0.007	0.041
rs62076103	G	A	1.23E-07	-0.19	0.035	-0.023	0.024
rs6448432	A	G	1.75E-06	0.09	0.019	0.013	0.013

Supplementary Table 2. Details of Hashimoto's Thyroiditis predicting SNPs with inflammatory proteins from IEU Open GWAS (with SNPs reaching $P < 5 \times 10^{-6}$).

Inflammatory proteins				Hashimoto's Thyroiditis			
SNP	Effect allele	Other allele	P value	Beta	Standard Error	log(OR)	Standard Error
rs6501550	T	C	1.42E-06	-0.18	0.037	0.069	0.035
rs7030280	T	C	6.44E-31	0.21	0.018	-0.010	0.013
rs72808590	C	T	1.55E-06	-0.37	0.077	-0.060	0.044
rs73165154	G	A	3.76E-06	-0.15	0.033	0.011	0.022
rs73192661	T	C	1.45E-09	-0.10	0.017	-0.024	0.012
rs7335748	G	A	2.41E-06	0.38	0.080	-0.014	0.041
rs766932	T	A	4.96E-07	0.10	0.019	-0.006	0.013
rs7767668	T	C	9.60E-07	-0.09	0.019	-0.014	0.012
rs7861040	C	G	9.08E-07	0.08	0.017	-0.001	0.012
rs79219789	C	G	2.84E-06	-0.15	0.033	0.006	0.031
rs8084503	T	C	1.97E-07	0.14	0.027	0.002	0.018
rs847803	G	A	7.91E-07	0.09	0.018	0.012	0.013
rs9271365	G	T	1.44E-39	0.25	0.019	0.016	0.012
rs9357119	T	G	3.65E-14	-0.15	0.020	-0.001	0.013
rs9497965	T	C	1.71E-06	0.08	0.017	-0.016	0.012
**LIF (F-statistic = 38.11, R2=0.44%)**							
rs10424978	A	C	8.97E-07	-0.08	0.017	0.015	0.014
rs10494077	A	G	9.20E-10	0.12	0.020	0.013	0.015
rs10786679	A	G	1.87E-06	-0.09	0.018	-0.012	0.015
rs111358861	G	A	3.30E-06	1.82	0.391	-0.036	0.107
rs1115587	A	G	1.82E-07	-0.10	0.018	0.015	0.014
rs11211645	G	A	9.33E-08	0.09	0.017	0.011	0.013
rs113075322	G	C	7.18E-07	-0.14	0.028	0.012	0.036
rs11611029	T	C	1.16E-08	-0.10	0.017	0.001	0.014
rs117167029	C	G	8.05E-07	2.99	0.606	0.091	0.111
rs117369326	A	G	3.55E-06	0.24	0.051	-0.041	0.033
rs118075239	C	T	3.45E-06	0.38	0.083	0.067	0.051
rs11822254	G	A	3.52E-06	-0.08	0.017	0.001	0.014
rs11889341	T	C	6.64E-11	0.12	0.019	-0.010	0.015
rs1317983	C	T	1.65E-07	0.09	0.018	-0.001	0.015
rs13249705	G	A	3.78E-07	-0.09	0.018	0.006	0.016

Supplementary Table 2. Details of Hashimoto's Thyroiditis predicting SNPs with inflammatory proteins from IEU Open GWAS (with SNPs reaching *P* < 5 x 10^{-6}).

Inflammatory proteins				Hashimoto's Thyroiditis			
SNP	Effect allele	Other allele	P value	Beta	Standard Error	log(OR)	Standard Error
rs140111921	A	G	1.34E-06	-0.16	0.034	-0.008	0.033
rs150619443	C	G	5.08E-07	0.44	0.088	-0.040	0.058
rs1534430	T	C	9.40E-08	-0.09	0.017	0.000	0.014
rs17490285	G	A	9.26E-07	-0.18	0.038	0.017	0.021
rs1887414	C	T	1.97E-07	0.12	0.022	0.001	0.018
rs2111485	G	A	4.19E-06	0.08	0.017	0.003	0.013
rs229526	C	G	1.93E-06	0.10	0.022	0.005	0.016
rs2476601	G	A	3.49E-45	-0.33	0.023	0.030	0.021
rs28367132	A	G	1.21E-07	-0.14	0.026	-0.015	0.017
rs28450758	A	C	4.17E-06	0.14	0.031	-0.018	0.025
rs3184504	C	T	1.40E-25	-0.18	0.017	-0.032	0.013
rs34536443	C	G	2.47E-07	-0.25	0.049	0.012	0.033
rs34636506	G	A	1.92E-14	-0.13	0.018	-0.027	0.014
rs61830417	G	A	3.59E-06	0.27	0.059	0.038	0.045
rs62076103	G	A	1.23E-07	-0.19	0.035	-0.025	0.026
rs6448432	A	G	1.75E-06	0.09	0.019	0.007	0.014
rs6501550	T	C	1.42E-06	-0.18	0.037	0.012	0.040
rs7030280	T	C	6.44E-31	0.21	0.018	0.007	0.014
rs72808590	C	T	1.55E-06	-0.37	0.077	0.043	0.048
rs73165154	G	A	3.76E-06	-0.15	0.033	-0.040	0.024
rs73192661	T	C	1.45E-09	-0.10	0.017	-0.002	0.014
rs7335748	G	A	2.41E-06	0.38	0.080	0.052	0.046
rs766932	T	A	4.96E-07	0.10	0.019	0.007	0.015
rs7767668	T	C	9.60E-07	-0.09	0.019	0.013	0.013
rs7861040	C	G	9.08E-07	0.08	0.017	0.017	0.013
rs79219789	C	G	2.84E-06	-0.15	0.033	0.023	0.035
rs8084503	T	C	1.97E-07	0.14	0.027	-0.008	0.021
rs847803	G	A	7.91E-07	0.09	0.018	0.021	0.014
rs9271365	G	T	1.44E-39	0.25	0.019	-0.021	0.013
rs9357119	T	G	3.65E-14	-0.15	0.020	0.002	0.015
rs9497965	T	C	1.71E-06	0.08	0.017	-0.009	0.013

Supplementary Table 2. Details of Hashimoto's Thyroiditis predicting SNPs with inflammatory proteins from IEU Open GWAS (with SNPs reaching *P* < 5 x 10^{-6}).

Inflammatory proteins				Hashimoto's Thyroiditis			
SNP	Effect allele	Other allele	P value	Beta	Standard Error	log(OR)	Standard Error
**LIFR (F-statistic = 38.11, R2=0.44%)**							
rs10424978	A	C	8.97E-07	-0.08	0.017	0.008	0.014
rs10494077	A	G	9.20E-10	0.12	0.020	-0.001	0.015
rs10786679	A	G	1.87E-06	-0.09	0.018	-0.020	0.015
rs111358861	G	A	3.30E-06	1.82	0.391	0.097	0.107
rs1115587	A	G	1.82E-07	-0.10	0.018	0.003	0.014
rs11211645	G	A	9.33E-08	0.09	0.017	0.022	0.012
rs113075322	G	C	7.18E-07	-0.14	0.028	0.018	0.036
rs11611029	T	C	1.16E-08	-0.10	0.017	-0.021	0.014
rs117167029	C	G	8.05E-07	2.99	0.606	0.159	0.111
rs117369326	A	G	3.55E-06	0.24	0.051	0.023	0.031
rs118075239	C	T	3.45E-06	0.38	0.083	0.011	0.050
rs11822254	G	A	3.52E-06	-0.08	0.017	0.007	0.013
rs11889341	T	C	6.64E-11	0.12	0.019	0.008	0.015
rs1317983	C	T	1.65E-07	0.09	0.018	0.021	0.015
rs13249705	G	A	3.78E-07	-0.09	0.018	0.002	0.016
rs140111921	A	G	1.34E-06	-0.16	0.034	-0.036	0.033
rs150619443	C	G	5.08E-07	0.44	0.088	0.016	0.055
rs1534430	T	C	9.40E-08	-0.09	0.017	0.009	0.014
rs17490285	G	A	9.26E-07	-0.18	0.038	0.011	0.021
rs1887414	C	T	1.97E-07	0.12	0.022	-0.001	0.017
rs2111485	G	A	4.19E-06	0.08	0.017	0.000	0.012
rs229526	C	G	1.93E-06	0.10	0.022	0.031	0.015
rs2476601	G	A	3.49E-45	-0.33	0.023	0.010	0.019
rs28367132	A	G	1.21E-07	-0.14	0.026	-0.007	0.016
rs28450758	A	C	4.17E-06	0.14	0.031	-0.047	0.023
rs3184504	C	T	1.40E-25	-0.18	0.017	-0.026	0.012
rs34536443	C	G	2.47E-07	-0.25	0.049	-0.001	0.032
rs34636506	G	A	1.92E-14	-0.13	0.018	0.016	0.013
rs61830417	G	A	3.59E-06	0.27	0.059	-0.022	0.044
rs62076103	G	A	1.23E-07	-0.19	0.035	0.035	0.025

Supplementary Table 2. Details of Hashimoto's Thyroiditis predicting SNPs with inflammatory proteins from IEU Open GWAS (with SNPs reaching $P < 5 \times 10^{-6}$).

Inflammatory proteins						Hashimoto's Thyroiditis	
SNP	Effect allele	Other allele	P value	Beta	Standard Error	log(OR)	Standard Error
rs6448432	A	G	1.75E-06	0.09	0.019	-0.015	0.013
rs6501550	T	C	1.42E-06	-0.18	0.037	-0.027	0.040
rs7030280	T	C	6.44E-31	0.21	0.018	-0.034	0.014
rs72808590	C	T	1.55E-06	-0.37	0.077	-0.014	0.046
rs73165154	G	A	3.76E-06	-0.15	0.033	0.018	0.024
rs73192661	T	C	1.45E-09	-0.10	0.017	0.012	0.014
rs7335748	G	A	2.41E-06	0.38	0.080	0.032	0.044
rs766932	T	A	4.96E-07	0.10	0.019	0.001	0.014
rs7767668	T	C	9.60E-07	-0.09	0.019	0.015	0.012
rs7861040	C	G	9.08E-07	0.08	0.017	-0.014	0.013
rs79219789	C	G	2.84E-06	-0.15	0.033	0.031	0.033
rs8084503	T	C	1.97E-07	0.14	0.027	0.013	0.020
rs847803	G	A	7.91E-07	0.09	0.018	-0.019	0.014
rs9271365	G	T	1.44E-39	0.25	0.019	0.021	0.012
rs9357119	T	G	3.65E-14	-0.15	0.020	0.012	0.014
rs9497965	T	C	1.71E-06	0.08	0.017	0.007	0.013
**M-CSF1 (F-statistic = 38.11, R2=0.44%)**							
rs10424978	A	C	8.97E-07	-0.08	0.017	-0.014	0.012
rs10494077	A	G	9.20E-10	0.12	0.020	0.006	0.014
rs10786679	A	G	1.87E-06	-0.09	0.018	0.016	0.013
rs111358861	G	A	3.30E-06	1.82	0.391	0.065	0.106
rs1115587	A	G	1.82E-07	-0.10	0.018	-0.002	0.012
rs11211645	G	A	9.33E-08	0.09	0.017	0.002	0.012
rs113075322	G	C	7.18E-07	-0.14	0.028	-0.015	0.033
rs11611029	T	C	1.16E-08	-0.10	0.017	0.003	0.012
rs117167029	C	G	8.05E-07	2.99	0.606	0.007	0.090
rs117369326	A	G	3.55E-06	0.24	0.051	-0.021	0.029
rs118075239	C	T	3.45E-06	0.38	0.083	0.075	0.046
rs11822254	G	A	3.52E-06	-0.08	0.017	0.005	0.012
rs11889341	T	C	6.64E-11	0.12	0.019	0.008	0.014
rs1317983	C	T	1.65E-07	0.09	0.018	0.017	0.013

Supplementary Table 2. Details of Hashimoto's Thyroiditis predicting SNPs with inflammatory proteins from IEU Open GWAS (with SNPs reaching $P < 5 \times 10^{-6}$).

Inflammatory proteins				Hashimoto's Thyroiditis			
SNP	Effect allele	Other allele	P value	Beta	Standard Error	log(OR)	Standard Error
rs13249705	G	A	3.78E-07	-0.09	0.018	-0.010	0.014
rs140111921	A	G	1.34E-06	-0.16	0.034	-0.006	0.030
rs150619443	C	G	5.08E-07	0.44	0.088	-0.058	0.053
rs1534430	T	C	9.40E-08	-0.09	0.017	-0.005	0.012
rs17490285	G	A	9.26E-07	-0.18	0.038	0.002	0.019
rs1887414	C	T	1.97E-07	0.12	0.022	-0.016	0.016
rs2111485	G	A	4.19E-06	0.08	0.017	-0.002	0.011
rs229526	C	G	1.93E-06	0.10	0.022	-0.004	0.014
rs2476601	G	A	3.49E-45	-0.33	0.023	0.021	0.018
rs28367132	A	G	1.21E-07	-0.14	0.026	-0.003	0.015
rs28450758	A	C	4.17E-06	0.14	0.031	0.007	0.022
rs3184504	C	T	1.40E-25	-0.18	0.017	-0.037	0.013
rs34536443	C	G	2.47E-07	-0.25	0.049	-0.001	0.030
rs34636506	G	A	1.92E-14	-0.13	0.018	0.007	0.012
rs61830417	G	A	3.59E-06	0.27	0.059	0.003	0.041
rs62076103	G	A	1.23E-07	-0.19	0.035	-0.014	0.024
rs6448432	A	G	1.75E-06	0.09	0.019	-0.005	0.012
rs6501550	T	C	1.42E-06	-0.18	0.037	0.013	0.034
rs7030280	T	C	6.44E-31	0.21	0.018	-0.031	0.013
rs72808590	C	T	1.55E-06	-0.37	0.077	-0.085	0.043
rs73165154	G	A	3.76E-06	-0.15	0.033	-0.001	0.022
rs73192661	T	C	1.45E-09	-0.10	0.017	0.019	0.012
rs7335748	G	A	2.41E-06	0.38	0.080	-0.056	0.040
rs766932	T	A	4.96E-07	0.10	0.019	0.005	0.013
rs7767668	T	C	9.60E-07	-0.09	0.019	-0.021	0.012
rs7861040	C	G	9.08E-07	0.08	0.017	-0.004	0.012
rs79219789	C	G	2.84E-06	-0.15	0.033	0.020	0.030
rs8084503	T	C	1.97E-07	0.14	0.027	0.020	0.018
rs847803	G	A	7.91E-07	0.09	0.018	0.013	0.013
rs9271365	G	T	1.44E-39	0.25	0.019	0.018	0.011
rs9357119	T	G	3.65E-14	-0.15	0.020	-0.011	0.013

Supplementary Table 2. Details of Hashimoto's Thyroiditis predicting SNPs with inflammatory proteins from IEU Open GWAS (with SNPs reaching $P < 5 \times 10^{-6}$).

Inflammatory proteins				Hashimoto's Thyroiditis			
SNP	Effect allele	Other allele	P value	Beta	Standard Error	log(OR)	Standard Error
rs9497965	T	C	1.71E-06	0.08	0.017	-0.004	0.012
**MCP-1 (F-statistic = 38.11, R2=0.44%)**							
rs10424978	A	C	8.97E-07	-0.08	0.017	-0.013	0.012
rs10494077	A	G	9.20E-10	0.12	0.020	-0.007	0.014
rs10786679	A	G	1.87E-06	-0.09	0.018	0.011	0.013
rs111358861	G	A	3.30E-06	1.82	0.391	0.008	0.107
rs1115587	A	G	1.82E-07	-0.10	0.018	-0.010	0.012
rs11211645	G	A	9.33E-08	0.09	0.017	0.000	0.012
rs113075322	G	C	7.18E-07	-0.14	0.028	0.014	0.033
rs11611029	T	C	1.16E-08	-0.10	0.017	-0.004	0.012
rs117167029	C	G	8.05E-07	2.99	0.606	0.081	0.091
rs117369326	A	G	3.55E-06	0.24	0.051	0.023	0.029
rs118075239	C	T	3.45E-06	0.38	0.083	0.063	0.047
rs11822254	G	A	3.52E-06	-0.08	0.017	0.007	0.012
rs11889341	T	C	6.64E-11	0.12	0.019	-0.015	0.014
rs1317983	C	T	1.65E-07	0.09	0.018	0.012	0.013
rs13249705	G	A	3.78E-07	-0.09	0.018	0.007	0.014
rs140111921	A	G	1.34E-06	-0.16	0.034	0.005	0.030
rs150619443	C	G	5.08E-07	0.44	0.088	0.013	0.054
rs1534430	T	C	9.40E-08	-0.09	0.017	0.014	0.012
rs17490285	G	A	9.26E-07	-0.18	0.038	0.021	0.019
rs1887414	C	T	1.97E-07	0.12	0.022	-0.021	0.017
rs2111485	G	A	4.19E-06	0.08	0.017	-0.006	0.012
rs229526	C	G	1.93E-06	0.10	0.022	0.016	0.015
rs2476601	G	A	3.49E-45	-0.33	0.023	-0.021	0.019
rs28367132	A	G	1.21E-07	-0.14	0.026	-0.022	0.015
rs28450758	A	C	4.17E-06	0.14	0.031	-0.003	0.022
rs3184504	C	T	1.40E-25	-0.18	0.017	0.015	0.013
rs34536443	C	G	2.47E-07	-0.25	0.049	-0.038	0.030
rs34636506	G	A	1.92E-14	-0.13	0.018	-0.001	0.012
rs61830417	G	A	3.59E-06	0.27	0.059	-0.011	0.041

Supplementary Table 2. Details of Hashimoto's Thyroiditis predicting SNPs with inflammatory proteins from IEU Open GWAS (with SNPs reaching $P < 5 \times 10^{-6}$).

Inflammatory proteins				Hashimoto's Thyroiditis			
SNP	Effect allele	Other allele	P value	Beta	Standard Error	log(OR)	Standard Error
rs62076103	G	A	1.23E-07	-0.19	0.035	-0.029	0.024
rs6448432	A	G	1.75E-06	0.09	0.019	-0.003	0.013
rs6501550	T	C	1.42E-06	-0.18	0.037	0.032	0.034
rs7030280	T	C	6.44E-31	0.21	0.018	-0.011	0.013
rs72808590	C	T	1.55E-06	-0.37	0.077	-0.048	0.044
rs73165154	G	A	3.76E-06	-0.15	0.033	0.001	0.022
rs73192661	T	C	1.45E-09	-0.10	0.017	-0.033	0.012
rs7335748	G	A	2.41E-06	0.38	0.080	0.002	0.041
rs766932	T	A	4.96E-07	0.10	0.019	0.008	0.013
rs7767668	T	C	9.60E-07	-0.09	0.019	-0.002	0.012
rs7861040	C	G	9.08E-07	0.08	0.017	-0.022	0.012
rs79219789	C	G	2.84E-06	-0.15	0.033	0.002	0.031
rs8084503	T	C	1.97E-07	0.14	0.027	0.008	0.018
rs847803	G	A	7.91E-07	0.09	0.018	0.026	0.013
rs9271365	G	T	1.44E-39	0.25	0.019	-0.004	0.012
rs9357119	T	G	3.65E-14	-0.15	0.020	-0.014	0.013
rs9497965	T	C	1.71E-06	0.08	0.017	-0.006	0.012
**MCP-2 (F-statistic = 38.11, R2=0.44%)**							
rs10424978	A	C	8.97E-07	-0.08	0.017	-0.018	0.012
rs10494077	A	G	9.20E-10	0.12	0.020	-0.017	0.014
rs10786679	A	G	1.87E-06	-0.09	0.018	0.024	0.014
rs111358861	G	A	3.30E-06	1.82	0.391	-0.063	0.107
rs1115587	A	G	1.82E-07	-0.10	0.018	0.019	0.012
rs11211645	G	A	9.33E-08	0.09	0.017	-0.014	0.012
rs113075322	G	C	7.18E-07	-0.14	0.028	-0.008	0.033
rs11611029	T	C	1.16E-08	-0.10	0.017	0.003	0.012
rs117167029	C	G	8.05E-07	2.99	0.606	-0.100	0.090
rs117369326	A	G	3.55E-06	0.24	0.051	-0.015	0.029
rs118075239	C	T	3.45E-06	0.38	0.083	-0.032	0.048
rs11822254	G	A	3.52E-06	-0.08	0.017	0.005	0.012
rs11889341	T	C	6.64E-11	0.12	0.019	0.008	0.014

Supplementary Table 2. Details of Hashimoto's Thyroiditis predicting SNPs with inflammatory proteins from IEU Open GWAS (with SNPs reaching $P < 5 \times 10^{-6}$).

Inflammatory proteins				Hashimoto's Thyroiditis			
SNP	Effect allele	Other allele	P value	Beta	Standard Error	log(OR)	Standard Error
rs1317983	C	T	1.65E-07	0.09	0.018	0.020	0.013
rs13249705	G	A	3.78E-07	-0.09	0.018	-0.009	0.014
rs140111921	A	G	1.34E-06	-0.16	0.034	0.062	0.031
rs150619443	C	G	5.08E-07	0.44	0.088	0.065	0.055
rs1534430	T	C	9.40E-08	-0.09	0.017	0.004	0.012
rs17490285	G	A	9.26E-07	-0.18	0.038	-0.025	0.019
rs1887414	C	T	1.97E-07	0.12	0.022	0.012	0.017
rs2111485	G	A	4.19E-06	0.08	0.017	0.019	0.012
rs229526	C	G	1.93E-06	0.10	0.022	-0.002	0.015
rs2476601	G	A	3.49E-45	-0.33	0.023	0.021	0.019
rs28367132	A	G	1.21E-07	-0.14	0.026	-0.023	0.015
rs28450758	A	C	4.17E-06	0.14	0.031	-0.021	0.023
rs3184504	C	T	1.40E-25	-0.18	0.017	-0.019	0.013
rs34536443	C	G	2.47E-07	-0.25	0.049	-0.049	0.030
rs34636506	G	A	1.92E-14	-0.13	0.018	0.000	0.012
rs61830417	G	A	3.59E-06	0.27	0.059	-0.010	0.042
rs62076103	G	A	1.23E-07	-0.19	0.035	-0.012	0.025
rs6448432	A	G	1.75E-06	0.09	0.019	0.000	0.013
rs6501550	T	C	1.42E-06	-0.18	0.037	0.017	0.035
rs7030280	T	C	6.44E-31	0.21	0.018	0.012	0.013
rs72808590	C	T	1.55E-06	-0.37	0.077	-0.010	0.045
rs73165154	G	A	3.76E-06	-0.15	0.033	-0.022	0.022
rs73192661	T	C	1.45E-09	-0.10	0.017	-0.002	0.012
rs7335748	G	A	2.41E-06	0.38	0.080	-0.038	0.041
rs766932	T	A	4.96E-07	0.10	0.019	0.009	0.013
rs7767668	T	C	9.60E-07	-0.09	0.019	0.012	0.012
rs7861040	C	G	9.08E-07	0.08	0.017	-0.022	0.012
rs79219789	C	G	2.84E-06	-0.15	0.033	-0.032	0.031
rs8084503	T	C	1.97E-07	0.14	0.027	0.002	0.018
rs847803	G	A	7.91E-07	0.09	0.018	0.012	0.013
rs9271365	G	T	1.44E-39	0.25	0.019	-0.037	0.012

Supplementary Table 2. Details of Hashimoto's Thyroiditis predicting SNPs with inflammatory proteins from IEU Open GWAS (with SNPs reaching $P < 5 \times 10^{-6}$).

Inflammatory proteins				Hashimoto's Thyroiditis			
SNP	Effect allele	Other allele	P value	Beta	Standard Error	log(OR)	Standard Error
rs9357119	T	G	3.65E-14	-0.15	0.020	0.003	0.013
rs9497965	T	C	1.71E-06	0.08	0.017	0.001	0.012
**MCP-3 (F-statistic = 38.11, R2=0.44%)**							
rs10424978	A	C	8.97E-07	-0.08	0.017	-0.010	0.014
rs10494077	A	G	9.20E-10	0.12	0.020	-0.004	0.016
rs10786679	A	G	1.87E-06	-0.09	0.018	-0.033	0.015
rs111358861	G	A	3.30E-06	1.82	0.391	0.032	0.107
rs1115587	A	G	1.82E-07	-0.10	0.018	0.033	0.014
rs11211645	G	A	9.33E-08	0.09	0.017	-0.013	0.013
rs113075322	G	C	7.18E-07	-0.14	0.028	0.020	0.036
rs11611029	T	C	1.16E-08	-0.10	0.017	0.000	0.014
rs117167029	C	G	8.05E-07	2.99	0.606	-0.017	0.111
rs117369326	A	G	3.55E-06	0.24	0.051	-0.027	0.033
rs118075239	C	T	3.45E-06	0.38	0.083	0.026	0.051
rs11822254	G	A	3.52E-06	-0.08	0.017	0.014	0.014
rs11889341	T	C	6.64E-11	0.12	0.019	0.008	0.016
rs1317983	C	T	1.65E-07	0.09	0.018	0.013	0.014
rs13249705	G	A	3.78E-07	-0.09	0.018	0.002	0.016
rs140111921	A	G	1.34E-06	-0.16	0.034	-0.017	0.033
rs150619443	C	G	5.08E-07	0.44	0.088	-0.024	0.059
rs1534430	T	C	9.40E-08	-0.09	0.017	0.016	0.014
rs17490285	G	A	9.26E-07	-0.18	0.038	0.011	0.021
rs1887414	C	T	1.97E-07	0.12	0.022	-0.005	0.019
rs2111485	G	A	4.19E-06	0.08	0.017	0.016	0.013
rs229526	C	G	1.93E-06	0.10	0.022	-0.020	0.017
rs2476601	G	A	3.49E-45	-0.33	0.023	0.040	0.022
rs28367132	A	G	1.21E-07	-0.14	0.026	-0.008	0.017
rs28450758	A	C	4.17E-06	0.14	0.031	0.004	0.025
rs3184504	C	T	1.40E-25	-0.18	0.017	-0.009	0.013
rs34536443	C	G	2.47E-07	-0.25	0.049	-0.062	0.034
rs34636506	G	A	1.92E-14	-0.13	0.018	-0.035	0.014

Supplementary Table 2. Details of Hashimoto's Thyroiditis predicting SNPs with inflammatory proteins from IEU Open GWAS (with SNPs reaching $P < 5 \times 10^{-6}$).

Inflammatory proteins				Hashimoto's Thyroiditis			
SNP	Effect allele	Other allele	P value	Beta	Standard Error	log(OR)	Standard Error
rs61830417	G	A	3.59E-06	0.27	0.059	0.033	0.046
rs62076103	G	A	1.23E-07	-0.19	0.035	-0.050	0.027
rs6448432	A	G	1.75E-06	0.09	0.019	0.011	0.015
rs6501550	T	C	1.42E-06	-0.18	0.037	0.049	0.039
rs7030280	T	C	6.44E-31	0.21	0.018	-0.004	0.014
rs72808590	C	T	1.55E-06	-0.37	0.077	-0.114	0.048
rs73165154	G	A	3.76E-06	-0.15	0.033	0.013	0.024
rs73192661	T	C	1.45E-09	-0.10	0.017	-0.026	0.014
rs7335748	G	A	2.41E-06	0.38	0.080	-0.007	0.047
rs766932	T	A	4.96E-07	0.10	0.019	-0.012	0.015
rs7767668	T	C	9.60E-07	-0.09	0.019	-0.021	0.013
rs7861040	C	G	9.08E-07	0.08	0.017	-0.006	0.014
rs79219789	C	G	2.84E-06	-0.15	0.033	0.077	0.036
rs8084503	T	C	1.97E-07	0.14	0.027	0.003	0.021
rs847803	G	A	7.91E-07	0.09	0.018	0.008	0.014
rs9271365	G	T	1.44E-39	0.25	0.019	-0.002	0.013
rs9357119	T	G	3.65E-14	-0.15	0.020	-0.005	0.015
rs9497965	T	C	1.71E-06	0.08	0.017	-0.006	0.014
**MCP-4 (F-statistic = 38.11, R2=0.44%)**							
rs10424978	A	C	8.97E-07	-0.08	0.017	-0.014	0.012
rs10494077	A	G	9.20E-10	0.12	0.020	-0.009	0.014
rs10786679	A	G	1.87E-06	-0.09	0.018	0.014	0.013
rs111358861	G	A	3.30E-06	1.82	0.391	-0.147	0.107
rs1115587	A	G	1.82E-07	-0.10	0.018	0.021	0.012
rs11211645	G	A	9.33E-08	0.09	0.017	-0.020	0.012
rs113075322	G	C	7.18E-07	-0.14	0.028	0.022	0.033
rs11611029	T	C	1.16E-08	-0.10	0.017	-0.004	0.012
rs117167029	C	G	8.05E-07	2.99	0.606	-0.014	0.089
rs117369326	A	G	3.55E-06	0.24	0.051	0.007	0.029
rs118075239	C	T	3.45E-06	0.38	0.083	0.045	0.047
rs11822254	G	A	3.52E-06	-0.08	0.017	0.005	0.012

Supplementary Table 2. Details of Hashimoto's Thyroiditis predicting SNPs with inflammatory proteins from IEU Open GWAS (with SNPs reaching $P < 5 \times 10^{-6}$).

Inflammatory proteins				Hashimoto's Thyroiditis			
SNP	Effect allele	Other allele	P value	Beta	Standard Error	log(OR)	Standard Error
rs11889341	T	C	6.64E-11	0.12	0.019	-0.019	0.014
rs1317983	C	T	1.65E-07	0.09	0.018	0.019	0.013
rs13249705	G	A	3.78E-07	-0.09	0.018	0.003	0.014
rs140111921	A	G	1.34E-06	-0.16	0.034	0.022	0.031
rs150619443	C	G	5.08E-07	0.44	0.088	0.016	0.054
rs1534430	T	C	9.40E-08	-0.09	0.017	0.010	0.012
rs17490285	G	A	9.26E-07	-0.18	0.038	0.021	0.019
rs1887414	C	T	1.97E-07	0.12	0.022	-0.004	0.017
rs2111485	G	A	4.19E-06	0.08	0.017	-0.010	0.012
rs229526	C	G	1.93E-06	0.10	0.022	0.024	0.015
rs2476601	G	A	3.49E-45	-0.33	0.023	0.021	0.019
rs28367132	A	G	1.21E-07	-0.14	0.026	-0.044	0.015
rs28450758	A	C	4.17E-06	0.14	0.031	-0.035	0.022
rs3184504	C	T	1.40E-25	-0.18	0.017	-0.008	0.013
rs34536443	C	G	2.47E-07	-0.25	0.049	-0.012	0.030
rs34636506	G	A	1.92E-14	-0.13	0.018	-0.003	0.012
rs61830417	G	A	3.59E-06	0.27	0.059	-0.006	0.041
rs62076103	G	A	1.23E-07	-0.19	0.035	-0.001	0.024
rs6448432	A	G	1.75E-06	0.09	0.019	0.032	0.013
rs6501550	T	C	1.42E-06	-0.18	0.037	0.019	0.035
rs7030280	T	C	6.44E-31	0.21	0.018	0.007	0.013
rs72808590	C	T	1.55E-06	-0.37	0.077	-0.051	0.044
rs73165154	G	A	3.76E-06	-0.15	0.033	-0.032	0.022
rs73192661	T	C	1.45E-09	-0.10	0.017	-0.009	0.012
rs7335748	G	A	2.41E-06	0.38	0.080	-0.001	0.041
rs766932	T	A	4.96E-07	0.10	0.019	-0.004	0.013
rs7767668	T	C	9.60E-07	-0.09	0.019	-0.014	0.012
rs7861040	C	G	9.08E-07	0.08	0.017	-0.022	0.012
rs79219789	C	G	2.84E-06	-0.15	0.033	0.048	0.031
rs8084503	T	C	1.97E-07	0.14	0.027	0.010	0.018
rs847803	G	A	7.91E-07	0.09	0.018	0.006	0.013

Supplementary Table 2. Details of Hashimoto's Thyroiditis predicting SNPs with inflammatory proteins from IEU Open GWAS (with SNPs reaching $P < 5 \times 10^{-6}$).

Inflammatory proteins				Hashimoto's Thyroiditis			
SNP	Effect allele	Other allele	P value	Beta	Standard Error	log(OR)	Standard Error
rs9271365	G	T	1.44E-39	0.25	0.019	0.020	0.012
rs9357119	T	G	3.65E-14	-0.15	0.020	-0.005	0.013
rs9497965	T	C	1.71E-06	0.08	0.017	-0.008	0.012
**MIP-1α (F-statistic = 36.52, R2=0.42%)**							
rs10424978	A	C	8.97E-07	-0.08	0.017	-0.037	0.012
rs10494077	A	G	9.20E-10	0.12	0.020	-0.001	0.014
rs10786679	A	G	1.87E-06	-0.09	0.018	-0.029	0.013
rs111358861	G	A	3.30E-06	1.82	0.391	-0.088	0.106
rs1115587	A	G	1.82E-07	-0.10	0.018	-0.009	0.012
rs11211645	G	A	9.33E-08	0.09	0.017	-0.027	0.012
rs113075322	G	C	7.18E-07	-0.14	0.028	0.033	0.033
rs11611029	T	C	1.16E-08	-0.10	0.017	0.002	0.012
rs117167029	C	G	8.05E-07	2.99	0.606	0.047	0.088
rs117369326	A	G	3.55E-06	0.24	0.051	0.007	0.029
rs118075239	C	T	3.45E-06	0.38	0.083	0.068	0.047
rs11822254	G	A	3.52E-06	-0.08	0.017	-0.002	0.012
rs11889341	T	C	6.64E-11	0.12	0.019	0.016	0.014
rs1317983	C	T	1.65E-07	0.09	0.018	-0.006	0.013
rs13249705	G	A	3.78E-07	-0.09	0.018	-0.011	0.014
rs140111921	A	G	1.34E-06	-0.16	0.034	-0.024	0.030
rs150619443	C	G	5.08E-07	0.44	0.088	0.035	0.054
rs1534430	T	C	9.40E-08	-0.09	0.017	0.012	0.012
rs17490285	G	A	9.26E-07	-0.18	0.038	-0.005	0.019
rs1887414	C	T	1.97E-07	0.12	0.022	-0.007	0.017
rs2111485	G	A	4.19E-06	0.08	0.017	-0.017	0.012
rs229526	C	G	1.93E-06	0.10	0.022	-0.027	0.015
rs2476601	G	A	3.49E-45	-0.33	0.023	0.027	0.019
rs28367132	A	G	1.21E-07	-0.14	0.026	-0.014	0.015
rs28450758	A	C	4.17E-06	0.14	0.031	-0.014	0.022
rs34536443	C	G	2.47E-07	-0.25	0.049	-0.038	0.030
rs34636506	G	A	1.92E-14	-0.13	0.018	-0.011	0.012

Supplementary Table 2. Details of Hashimoto's Thyroiditis predicting SNPs with inflammatory proteins from IEU Open GWAS (with SNPs reaching $P < 5 \times 10^{-6}$).

Inflammatory proteins				Hashimoto's Thyroiditis			
SNP	Effect allele	Other allele	P value	Beta	Standard Error	log(OR)	Standard Error
rs61830417	G	A	3.59E-06	0.27	0.059	0.026	0.041
rs62076103	G	A	1.23E-07	-0.19	0.035	0.017	0.024
rs6448432	A	G	1.75E-06	0.09	0.019	0.019	0.013
rs6501550	T	C	1.42E-06	-0.18	0.037	0.029	0.034
rs7030280	T	C	6.44E-31	0.21	0.018	0.003	0.013
rs72808590	C	T	1.55E-06	-0.37	0.077	-0.063	0.045
rs73165154	G	A	3.76E-06	-0.15	0.033	-0.025	0.022
rs73192661	T	C	1.45E-09	-0.10	0.017	-0.010	0.012
rs7335748	G	A	2.41E-06	0.38	0.080	-0.020	0.041
rs766932	T	A	4.96E-07	0.10	0.019	0.010	0.013
rs7767668	T	C	9.60E-07	-0.09	0.019	-0.002	0.012
rs7861040	C	G	9.08E-07	0.08	0.017	-0.014	0.012
rs79219789	C	G	2.84E-06	-0.15	0.033	0.043	0.031
rs8084503	T	C	1.97E-07	0.14	0.027	0.016	0.018
rs847803	G	A	7.91E-07	0.09	0.018	0.023	0.013
rs9271365	G	T	1.44E-39	0.25	0.019	0.004	0.012
rs9357119	T	G	3.65E-14	-0.15	0.020	-0.005	0.013
rs9497965	T	C	1.71E-06	0.08	0.017	-0.007	0.012
**MMP-1 (F-statistic = 38.58, R2=0.43%)**							
rs10424978	A	C	8.97E-07	-0.08	0.017	-0.013	0.012
rs10494077	A	G	9.20E-10	0.12	0.020	-0.014	0.014
rs10786679	A	G	1.87E-06	-0.09	0.018	0.018	0.014
rs111358861	G	A	3.30E-06	1.82	0.391	0.000	0.107
rs11211645	G	A	9.33E-08	0.09	0.017	-0.020	0.012
rs113075322	G	C	7.18E-07	-0.14	0.028	0.000	0.033
rs11611029	T	C	1.16E-08	-0.10	0.017	0.004	0.012
rs117167029	C	G	8.05E-07	2.99	0.606	0.077	0.089
rs117369326	A	G	3.55E-06	0.24	0.051	0.014	0.029
rs118075239	C	T	3.45E-06	0.38	0.083	0.028	0.048
rs11822254	G	A	3.52E-06	-0.08	0.017	-0.015	0.012
rs11889341	T	C	6.64E-11	0.12	0.019	-0.003	0.014

Supplementary Table 2. Details of Hashimoto's Thyroiditis predicting SNPs with inflammatory proteins from IEU Open GWAS (with SNPs reaching *P* < 5 x 10[-6]).

Inflammatory proteins				Hashimoto's Thyroiditis			
SNP	Effect allele	Other allele	P value	Beta	Standard Error	log(OR)	Standard Error
rs1317983	C	T	1.65E-07	0.09	0.018	0.005	0.013
rs13249705	G	A	3.78E-07	-0.09	0.018	-0.002	0.014
rs140111921	A	G	1.34E-06	-0.16	0.034	0.017	0.031
rs150619443	C	G	5.08E-07	0.44	0.088	0.006	0.055
rs1534430	T	C	9.40E-08	-0.09	0.017	0.002	0.012
rs17490285	G	A	9.26E-07	-0.18	0.038	0.019	0.019
rs1887414	C	T	1.97E-07	0.12	0.022	-0.020	0.017
rs2111485	G	A	4.19E-06	0.08	0.017	-0.011	0.012
rs229526	C	G	1.93E-06	0.10	0.022	-0.007	0.015
rs2476601	G	A	3.49E-45	-0.33	0.023	-0.008	0.020
rs28450758	A	C	4.17E-06	0.14	0.031	-0.024	0.023
rs3184504	C	T	1.40E-25	-0.18	0.017	-0.036	0.013
rs34536443	C	G	2.47E-07	-0.25	0.049	-0.011	0.031
rs34636506	G	A	1.92E-14	-0.13	0.018	0.001	0.012
rs61830417	G	A	3.59E-06	0.27	0.059	0.056	0.042
rs62076103	G	A	1.23E-07	-0.19	0.035	0.039	0.025
rs6448432	A	G	1.75E-06	0.09	0.019	0.016	0.013
rs6501550	T	C	1.42E-06	-0.18	0.037	-0.047	0.035
rs7030280	T	C	6.44E-31	0.21	0.018	-0.007	0.013
rs72808590	C	T	1.55E-06	-0.37	0.077	-0.010	0.045
rs73165154	G	A	3.76E-06	-0.15	0.033	-0.023	0.022
rs73192661	T	C	1.45E-09	-0.10	0.017	-0.015	0.012
rs7335748	G	A	2.41E-06	0.38	0.080	0.051	0.041
rs766932	T	A	4.96E-07	0.10	0.019	-0.019	0.013
rs7767668	T	C	9.60E-07	-0.09	0.019	-0.014	0.012
rs7861040	C	G	9.08E-07	0.08	0.017	-0.012	0.012
rs79219789	C	G	2.84E-06	-0.15	0.033	0.028	0.031
rs8084503	T	C	1.97E-07	0.14	0.027	0.016	0.019
rs847803	G	A	7.91E-07	0.09	0.018	-0.003	0.013
rs9271365	G	T	1.44E-39	0.25	0.019	-0.003	0.012
rs9357119	T	G	3.65E-14	-0.15	0.020	0.007	0.013

Supplementary Table 2. Details of Hashimoto's Thyroiditis predicting SNPs with inflammatory proteins from IEU Open GWAS (with SNPs reaching $P < 5 \times 10^{-6}$).

Inflammatory proteins				Hashimoto's Thyroiditis			
SNP	Effect allele	Other allele	P value	Beta	Standard Error	log(OR)	Standard Error
rs9497965	T	C	1.71E-06	0.08	0.017	-0.019	0.012
**MMP-10 (F-statistic = 38.11, R2=0.44%)**							
rs10424978	A	C	8.97E-07	-0.08	0.017	-0.021	0.012
rs10494077	A	G	9.20E-10	0.12	0.020	-0.006	0.014
rs10786679	A	G	1.87E-06	-0.09	0.018	0.002	0.014
rs111358861	G	A	3.30E-06	1.82	0.391	-0.083	0.106
rs1115587	A	G	1.82E-07	-0.10	0.018	0.030	0.012
rs11211645	G	A	9.33E-08	0.09	0.017	-0.006	0.012
rs113075322	G	C	7.18E-07	-0.14	0.028	-0.046	0.033
rs11611029	T	C	1.16E-08	-0.10	0.017	-0.006	0.012
rs117167029	C	G	8.05E-07	2.99	0.606	0.026	0.092
rs117369326	A	G	3.55E-06	0.24	0.051	-0.010	0.029
rs118075239	C	T	3.45E-06	0.38	0.083	0.132	0.047
rs11822254	G	A	3.52E-06	-0.08	0.017	0.013	0.012
rs11889341	T	C	6.64E-11	0.12	0.019	-0.008	0.014
rs1317983	C	T	1.65E-07	0.09	0.018	0.003	0.013
rs13249705	G	A	3.78E-07	-0.09	0.018	-0.012	0.014
rs140111921	A	G	1.34E-06	-0.16	0.034	-0.001	0.031
rs150619443	C	G	5.08E-07	0.44	0.088	-0.083	0.055
rs1534430	T	C	9.40E-08	-0.09	0.017	-0.008	0.012
rs17490285	G	A	9.26E-07	-0.18	0.038	-0.009	0.019
rs1887414	C	T	1.97E-07	0.12	0.022	-0.030	0.017
rs2111485	G	A	4.19E-06	0.08	0.017	-0.020	0.012
rs229526	C	G	1.93E-06	0.10	0.022	0.005	0.015
rs2476601	G	A	3.49E-45	-0.33	0.023	0.014	0.019
rs28367132	A	G	1.21E-07	-0.14	0.026	-0.007	0.015
rs28450758	A	C	4.17E-06	0.14	0.031	0.032	0.023
rs3184504	C	T	1.40E-25	-0.18	0.017	-0.009	0.013
rs34536443	C	G	2.47E-07	-0.25	0.049	0.031	0.030
rs34636506	G	A	1.92E-14	-0.13	0.018	-0.001	0.012
rs61830417	G	A	3.59E-06	0.27	0.059	0.033	0.042

Supplementary Table 2. Details of Hashimoto's Thyroiditis predicting SNPs with inflammatory proteins from IEU Open GWAS (with SNPs reaching *P* < 5 x 10^{-6}).

Inflammatory proteins				Hashimoto's Thyroiditis			
SNP	Effect allele	Other allele	P value	Beta	Standard Error	log(OR)	Standard Error
rs62076103	G	A	1.23E-07	-0.19	0.035	0.036	0.025
rs6448432	A	G	1.75E-06	0.09	0.019	0.000	0.013
rs6501550	T	C	1.42E-06	-0.18	0.037	0.069	0.035
rs7030280	T	C	6.44E-31	0.21	0.018	-0.007	0.013
rs72808590	C	T	1.55E-06	-0.37	0.077	-0.075	0.045
rs73165154	G	A	3.76E-06	-0.15	0.033	-0.004	0.022
rs73192661	T	C	1.45E-09	-0.10	0.017	-0.003	0.012
rs7335748	G	A	2.41E-06	0.38	0.080	0.000	0.041
rs766932	T	A	4.96E-07	0.10	0.019	0.008	0.013
rs7767668	T	C	9.60E-07	-0.09	0.019	-0.006	0.012
rs7861040	C	G	9.08E-07	0.08	0.017	0.016	0.012
rs79219789	C	G	2.84E-06	-0.15	0.033	-0.028	0.031
rs8084503	T	C	1.97E-07	0.14	0.027	0.025	0.018
rs847803	G	A	7.91E-07	0.09	0.018	0.000	0.013
rs9271365	G	T	1.44E-39	0.25	0.019	0.036	0.012
rs9357119	T	G	3.65E-14	-0.15	0.020	0.013	0.013
rs9497965	T	C	1.71E-06	0.08	0.017	-0.006	0.012
**NKR2B4 (F-statistic = 36.52, R2=0.42%)**							
rs10424978	A	C	8.97E-07	-0.08	0.017	-0.025	0.012
rs10494077	A	G	9.20E-10	0.12	0.020	0.012	0.014
rs10786679	A	G	1.87E-06	-0.09	0.018	0.005	0.014
rs111358861	G	A	3.30E-06	1.82	0.391	-0.226	0.107
rs1115587	A	G	1.82E-07	-0.10	0.018	0.014	0.012
rs11211645	G	A	9.33E-08	0.09	0.017	-0.012	0.012
rs113075322	G	C	7.18E-07	-0.14	0.028	-0.018	0.033
rs11611029	T	C	1.16E-08	-0.10	0.017	-0.007	0.012
rs117167029	C	G	8.05E-07	2.99	0.606	-0.145	0.091
rs117369326	A	G	3.55E-06	0.24	0.051	0.014	0.029
rs118075239	C	T	3.45E-06	0.38	0.083	0.047	0.047
rs11822254	G	A	3.52E-06	-0.08	0.017	-0.015	0.012
rs11889341	T	C	6.64E-11	0.12	0.019	-0.013	0.014

Supplementary Table 2. Details of Hashimoto's Thyroiditis predicting SNPs with inflammatory proteins from IEU Open GWAS (with SNPs reaching $P < 5 \times 10^{-6}$).

Inflammatory proteins				Hashimoto's Thyroiditis			
SNP	Effect allele	Other allele	P value	Beta	Standard Error	log(OR)	Standard Error
rs1317983	C	T	1.65E-07	0.09	0.018	-0.004	0.013
rs13249705	G	A	3.78E-07	-0.09	0.018	-0.019	0.014
rs140111921	A	G	1.34E-06	-0.16	0.034	0.009	0.031
rs150619443	C	G	5.08E-07	0.44	0.088	-0.041	0.054
rs1534430	T	C	9.40E-08	-0.09	0.017	-0.005	0.012
rs17490285	G	A	9.26E-07	-0.18	0.038	0.005	0.019
rs1887414	C	T	1.97E-07	0.12	0.022	-0.012	0.016
rs2111485	G	A	4.19E-06	0.08	0.017	-0.028	0.012
rs229526	C	G	1.93E-06	0.10	0.022	0.015	0.015
rs2476601	G	A	3.49E-45	-0.33	0.023	0.015	0.019
rs28367132	A	G	1.21E-07	-0.14	0.026	-0.004	0.015
rs28450758	A	C	4.17E-06	0.14	0.031	-0.043	0.022
rs34536443	C	G	2.47E-07	-0.25	0.049	-0.061	0.030
rs34636506	G	A	1.92E-14	-0.13	0.018	0.001	0.012
rs61830417	G	A	3.59E-06	0.27	0.059	-0.045	0.041
rs62076103	G	A	1.23E-07	-0.19	0.035	0.004	0.024
rs6448432	A	G	1.75E-06	0.09	0.019	-0.003	0.013
rs6501550	T	C	1.42E-06	-0.18	0.037	0.051	0.035
rs7030280	T	C	6.44E-31	0.21	0.018	-0.004	0.013
rs72808590	C	T	1.55E-06	-0.37	0.077	-0.060	0.044
rs73165154	G	A	3.76E-06	-0.15	0.033	0.022	0.022
rs73192661	T	C	1.45E-09	-0.10	0.017	0.002	0.012
rs7335748	G	A	2.41E-06	0.38	0.080	0.024	0.040
rs766932	T	A	4.96E-07	0.10	0.019	0.004	0.013
rs7767668	T	C	9.60E-07	-0.09	0.019	-0.014	0.012
rs7861040	C	G	9.08E-07	0.08	0.017	0.013	0.012
rs79219789	C	G	2.84E-06	-0.15	0.033	-0.020	0.030
rs8084503	T	C	1.97E-07	0.14	0.027	0.003	0.018
rs847803	G	A	7.91E-07	0.09	0.018	0.016	0.013
rs9271365	G	T	1.44E-39	0.25	0.019	0.013	0.012
rs9357119	T	G	3.65E-14	-0.15	0.020	-0.006	0.013

Supplementary Table 2. Details of Hashimoto's Thyroiditis predicting SNPs with inflammatory proteins from IEU Open GWAS (with SNPs reaching $P < 5 \times 10^{-6}$).

Inflammatory proteins				Hashimoto's Thyroiditis			
SNP	Effect allele	Other allele	P value	Beta	Standard Error	log(OR)	Standard Error
rs9497965	T	C	1.71E-06	0.08	0.017	-0.008	0.012
**NT-3 (F-statistic = 38.11, R2=0.44%)**							
rs10424978	A	C	8.97E-07	-0.08	0.017	-0.006	0.012
rs10494077	A	G	9.20E-10	0.12	0.020	0.007	0.014
rs10786679	A	G	1.87E-06	-0.09	0.018	-0.008	0.014
rs111358861	G	A	3.30E-06	1.82	0.391	0.038	0.107
rs1115587	A	G	1.82E-07	-0.10	0.018	0.010	0.012
rs11211645	G	A	9.33E-08	0.09	0.017	0.008	0.012
rs113075322	G	C	7.18E-07	-0.14	0.028	-0.051	0.033
rs11611029	T	C	1.16E-08	-0.10	0.017	-0.018	0.012
rs117167029	C	G	8.05E-07	2.99	0.606	-0.070	0.089
rs117369326	A	G	3.55E-06	0.24	0.051	-0.023	0.029
rs118075239	C	T	3.45E-06	0.38	0.083	-0.017	0.047
rs11822254	G	A	3.52E-06	-0.08	0.017	0.004	0.012
rs11889341	T	C	6.64E-11	0.12	0.019	-0.007	0.014
rs1317983	C	T	1.65E-07	0.09	0.018	-0.016	0.013
rs13249705	G	A	3.78E-07	-0.09	0.018	-0.002	0.014
rs140111921	A	G	1.34E-06	-0.16	0.034	-0.088	0.031
rs150619443	C	G	5.08E-07	0.44	0.088	-0.003	0.054
rs1534430	T	C	9.40E-08	-0.09	0.017	-0.006	0.012
rs17490285	G	A	9.26E-07	-0.18	0.038	0.018	0.019
rs1887414	C	T	1.97E-07	0.12	0.022	-0.006	0.016
rs2111485	G	A	4.19E-06	0.08	0.017	-0.003	0.012
rs229526	C	G	1.93E-06	0.10	0.022	-0.011	0.015
rs2476601	G	A	3.49E-45	-0.33	0.023	0.034	0.019
rs28367132	A	G	1.21E-07	-0.14	0.026	0.010	0.015
rs28450758	A	C	4.17E-06	0.14	0.031	-0.030	0.022
rs3184504	C	T	1.40E-25	-0.18	0.017	0.018	0.013
rs34536443	C	G	2.47E-07	-0.25	0.049	-0.006	0.030
rs34636506	G	A	1.92E-14	-0.13	0.018	0.016	0.012
rs61830417	G	A	3.59E-06	0.27	0.059	-0.009	0.041

Supplementary Table 2. Details of Hashimoto's Thyroiditis predicting SNPs with inflammatory proteins from IEU Open GWAS (with SNPs reaching $P < 5 \times 10^{-6}$).

Inflammatory proteins						Hashimoto's Thyroiditis	
SNP	Effect allele	Other allele	P value	Beta	Standard Error	log(OR)	Standard Error
rs62076103	G	A	1.23E-07	-0.19	0.035	0.018	0.024
rs6448432	A	G	1.75E-06	0.09	0.019	0.003	0.013
rs6501550	T	C	1.42E-06	-0.18	0.037	-0.015	0.035
rs7030280	T	C	6.44E-31	0.21	0.018	-0.001	0.013
rs72808590	C	T	1.55E-06	-0.37	0.077	0.021	0.044
rs73165154	G	A	3.76E-06	-0.15	0.033	-0.033	0.022
rs73192661	T	C	1.45E-09	-0.10	0.017	0.002	0.012
rs7335748	G	A	2.41E-06	0.38	0.080	-0.013	0.041
rs766932	T	A	4.96E-07	0.10	0.019	0.009	0.013
rs7767668	T	C	9.60E-07	-0.09	0.019	0.003	0.012
rs7861040	C	G	9.08E-07	0.08	0.017	0.009	0.012
rs79219789	C	G	2.84E-06	-0.15	0.033	-0.042	0.030
rs8084503	T	C	1.97E-07	0.14	0.027	-0.007	0.018
rs847803	G	A	7.91E-07	0.09	0.018	0.002	0.013
rs9271365	G	T	1.44E-39	0.25	0.019	0.000	0.012
rs9357119	T	G	3.65E-14	-0.15	0.020	-0.018	0.013
rs9497965	T	C	1.71E-06	0.08	0.017	-0.006	0.012
**NTN (F-statistic = 38.11, R2=0.44%)**							
rs10424978	A	C	8.97E-07	-0.08	0.017	-0.012	0.014
rs10494077	A	G	9.20E-10	0.12	0.020	0.002	0.015
rs10786679	A	G	1.87E-06	-0.09	0.018	-0.038	0.015
rs111358861	G	A	3.30E-06	1.82	0.391	0.030	0.107
rs1115587	A	G	1.82E-07	-0.10	0.018	0.014	0.014
rs11211645	G	A	9.33E-08	0.09	0.017	-0.020	0.013
rs113075322	G	C	7.18E-07	-0.14	0.028	0.052	0.036
rs11611029	T	C	1.16E-08	-0.10	0.017	-0.002	0.014
rs117167029	C	G	8.05E-07	2.99	0.606	0.054	0.111
rs117369326	A	G	3.55E-06	0.24	0.051	-0.002	0.032
rs118075239	C	T	3.45E-06	0.38	0.083	-0.015	0.051
rs11822254	G	A	3.52E-06	-0.08	0.017	-0.005	0.014
rs11889341	T	C	6.64E-11	0.12	0.019	0.013	0.015

Supplementary Table 2. Details of Hashimoto's Thyroiditis predicting SNPs with inflammatory proteins from IEU Open GWAS (with SNPs reaching $P < 5 \times 10^{-6}$).

Inflammatory proteins				Hashimoto's Thyroiditis			
SNP	Effect allele	Other allele	P value	Beta	Standard Error	log(OR)	Standard Error
rs1317983	C	T	1.65E-07	0.09	0.018	0.008	0.015
rs13249705	G	A	3.78E-07	-0.09	0.018	-0.023	0.016
rs140111921	A	G	1.34E-06	-0.16	0.034	-0.044	0.033
rs150619443	C	G	5.08E-07	0.44	0.088	-0.017	0.057
rs1534430	T	C	9.40E-08	-0.09	0.017	0.005	0.014
rs17490285	G	A	9.26E-07	-0.18	0.038	0.027	0.021
rs1887414	C	T	1.97E-07	0.12	0.022	-0.011	0.018
rs2111485	G	A	4.19E-06	0.08	0.017	-0.001	0.013
rs229526	C	G	1.93E-06	0.10	0.022	-0.020	0.016
rs2476601	G	A	3.49E-45	-0.33	0.023	0.014	0.021
rs28367132	A	G	1.21E-07	-0.14	0.026	-0.014	0.017
rs28450758	A	C	4.17E-06	0.14	0.031	-0.032	0.025
rs3184504	C	T	1.40E-25	-0.18	0.017	-0.002	0.013
rs34536443	C	G	2.47E-07	-0.25	0.049	0.005	0.033
rs34636506	G	A	1.92E-14	-0.13	0.018	-0.025	0.014
rs61830417	G	A	3.59E-06	0.27	0.059	0.072	0.045
rs62076103	G	A	1.23E-07	-0.19	0.035	-0.041	0.026
rs6448432	A	G	1.75E-06	0.09	0.019	-0.007	0.014
rs6501550	T	C	1.42E-06	-0.18	0.037	-0.035	0.040
rs7030280	T	C	6.44E-31	0.21	0.018	0.017	0.014
rs72808590	C	T	1.55E-06	-0.37	0.077	0.024	0.048
rs73165154	G	A	3.76E-06	-0.15	0.033	-0.013	0.024
rs73192661	T	C	1.45E-09	-0.10	0.017	0.007	0.014
rs7335748	G	A	2.41E-06	0.38	0.080	0.044	0.046
rs766932	T	A	4.96E-07	0.10	0.019	0.015	0.014
rs7767668	T	C	9.60E-07	-0.09	0.019	0.026	0.013
rs7861040	C	G	9.08E-07	0.08	0.017	-0.003	0.013
rs79219789	C	G	2.84E-06	-0.15	0.033	-0.034	0.035
rs8084503	T	C	1.97E-07	0.14	0.027	-0.003	0.021
rs847803	G	A	7.91E-07	0.09	0.018	0.015	0.014
rs9271365	G	T	1.44E-39	0.25	0.019	0.006	0.013

Supplementary Table 2. Details of Hashimoto's Thyroiditis predicting SNPs with inflammatory proteins from IEU Open GWAS (with SNPs reaching $P < 5 \times 10^{-6}$).

Inflammatory proteins				Hashimoto's Thyroiditis			
SNP	Effect allele	Other allele	P value	Beta	Standard Error	log(OR)	Standard Error
rs9357119	T	G	3.65E-14	-0.15	0.020	0.010	0.015
rs9497965	T	C	1.71E-06	0.08	0.017	-0.024	0.013
**OPG (F-statistic = 38.11, R2=0.44%)**							
rs10424978	A	C	8.97E-07	-0.08	0.017	-0.019	0.012
rs10494077	A	G	9.20E-10	0.12	0.020	0.010	0.013
rs10786679	A	G	1.87E-06	-0.09	0.018	-0.027	0.013
rs111358861	G	A	3.30E-06	1.82	0.391	0.087	0.106
rs1115587	A	G	1.82E-07	-0.10	0.018	0.010	0.012
rs11211645	G	A	9.33E-08	0.09	0.017	-0.005	0.011
rs113075322	G	C	7.18E-07	-0.14	0.028	0.108	0.032
rs11611029	T	C	1.16E-08	-0.10	0.017	-0.018	0.012
rs117167029	C	G	8.05E-07	2.99	0.606	0.040	0.086
rs117369326	A	G	3.55E-06	0.24	0.051	0.009	0.028
rs118075239	C	T	3.45E-06	0.38	0.083	0.069	0.045
rs11822254	G	A	3.52E-06	-0.08	0.017	-0.007	0.012
rs11889341	T	C	6.64E-11	0.12	0.019	-0.007	0.013
rs1317983	C	T	1.65E-07	0.09	0.018	0.005	0.012
rs13249705	G	A	3.78E-07	-0.09	0.018	0.021	0.014
rs140111921	A	G	1.34E-06	-0.16	0.034	-0.084	0.029
rs150619443	C	G	5.08E-07	0.44	0.088	-0.034	0.053
rs1534430	T	C	9.40E-08	-0.09	0.017	0.000	0.012
rs17490285	G	A	9.26E-07	-0.18	0.038	0.007	0.018
rs1887414	C	T	1.97E-07	0.12	0.022	-0.014	0.016
rs2111485	G	A	4.19E-06	0.08	0.017	-0.003	0.011
rs229526	C	G	1.93E-06	0.10	0.022	0.021	0.014
rs2476601	G	A	3.49E-45	-0.33	0.023	0.022	0.018
rs28367132	A	G	1.21E-07	-0.14	0.026	-0.022	0.015
rs28450758	A	C	4.17E-06	0.14	0.031	-0.021	0.022
rs3184504	C	T	1.40E-25	-0.18	0.017	-0.002	0.013
rs34536443	C	G	2.47E-07	-0.25	0.049	-0.001	0.029
rs34636506	G	A	1.92E-14	-0.13	0.018	0.019	0.012

Supplementary Table 2. Details of Hashimoto's Thyroiditis predicting SNPs with inflammatory proteins from IEU Open GWAS (with SNPs reaching *P* < 5 x 10^{-6}).

Inflammatory proteins						Hashimoto's Thyroiditis	
SNP	Effect allele	Other allele	P value	Beta	Standard Error	log(OR)	Standard Error
rs61830417	G	A	3.59E-06	0.27	0.059	-0.077	0.040
rs62076103	G	A	1.23E-07	-0.19	0.035	-0.042	0.024
rs6448432	A	G	1.75E-06	0.09	0.019	-0.009	0.012
rs6501550	T	C	1.42E-06	-0.18	0.037	0.036	0.032
rs7030280	T	C	6.44E-31	0.21	0.018	-0.031	0.012
rs72808590	C	T	1.55E-06	-0.37	0.077	-0.064	0.043
rs73165154	G	A	3.76E-06	-0.15	0.033	-0.004	0.021
rs73192661	T	C	1.45E-09	-0.10	0.017	-0.001	0.012
rs7335748	G	A	2.41E-06	0.38	0.080	-0.019	0.040
rs766932	T	A	4.96E-07	0.10	0.019	-0.008	0.012
rs7767668	T	C	9.60E-07	-0.09	0.019	-0.005	0.011
rs7861040	C	G	9.08E-07	0.08	0.017	0.000	0.012
rs79219789	C	G	2.84E-06	-0.15	0.033	0.027	0.029
rs8084503	T	C	1.97E-07	0.14	0.027	0.018	0.018
rs847803	G	A	7.91E-07	0.09	0.018	-0.029	0.012
rs9271365	G	T	1.44E-39	0.25	0.019	-0.023	0.011
rs9357119	T	G	3.65E-14	-0.15	0.020	-0.020	0.013
rs9497965	T	C	1.71E-06	0.08	0.017	0.003	0.012
**OSM (F-statistic = 36.52, R2=0.42%)**							
rs10424978	A	C	8.97E-07	-0.08	0.017	-0.003	0.012
rs10494077	A	G	9.20E-10	0.12	0.020	0.001	0.014
rs10786679	A	G	1.87E-06	-0.09	0.018	0.002	0.014
rs111358861	G	A	3.30E-06	1.82	0.391	-0.188	0.107
rs1115587	A	G	1.82E-07	-0.10	0.018	0.026	0.012
rs11211645	G	A	9.33E-08	0.09	0.017	0.012	0.012
rs113075322	G	C	7.18E-07	-0.14	0.028	0.002	0.033
rs11611029	T	C	1.16E-08	-0.10	0.017	0.004	0.012
rs117167029	C	G	8.05E-07	2.99	0.606	-0.001	0.090
rs117369326	A	G	3.55E-06	0.24	0.051	-0.011	0.029
rs118075239	C	T	3.45E-06	0.38	0.083	-0.023	0.048
rs11822254	G	A	3.52E-06	-0.08	0.017	0.000	0.012

Supplementary Table 2. Details of Hashimoto's Thyroiditis predicting SNPs with inflammatory proteins from IEU Open GWAS (with SNPs reaching $P < 5 \times 10^{-6}$).

Inflammatory proteins				Hashimoto's Thyroiditis			
SNP	Effect allele	Other allele	P value	Beta	Standard Error	log(OR)	Standard Error
rs11889341	T	C	6.64E-11	0.12	0.019	0.024	0.014
rs1317983	C	T	1.65E-07	0.09	0.018	0.014	0.013
rs13249705	G	A	3.78E-07	-0.09	0.018	0.033	0.014
rs140111921	A	G	1.34E-06	-0.16	0.034	-0.020	0.031
rs150619443	C	G	5.08E-07	0.44	0.088	-0.029	0.055
rs1534430	T	C	9.40E-08	-0.09	0.017	0.032	0.012
rs17490285	G	A	9.26E-07	-0.18	0.038	-0.005	0.019
rs1887414	C	T	1.97E-07	0.12	0.022	-0.017	0.017
rs2111485	G	A	4.19E-06	0.08	0.017	0.015	0.012
rs229526	C	G	1.93E-06	0.10	0.022	0.005	0.015
rs2476601	G	A	3.49E-45	-0.33	0.023	0.039	0.020
rs28367132	A	G	1.21E-07	-0.14	0.026	-0.017	0.015
rs28450758	A	C	4.17E-06	0.14	0.031	-0.017	0.023
rs34536443	C	G	2.47E-07	-0.25	0.049	-0.019	0.031
rs34636506	G	A	1.92E-14	-0.13	0.018	-0.016	0.012
rs61830417	G	A	3.59E-06	0.27	0.059	0.079	0.042
rs62076103	G	A	1.23E-07	-0.19	0.035	0.004	0.025
rs6448432	A	G	1.75E-06	0.09	0.019	0.017	0.013
rs6501550	T	C	1.42E-06	-0.18	0.037	-0.001	0.035
rs7030280	T	C	6.44E-31	0.21	0.018	-0.005	0.013
rs72808590	C	T	1.55E-06	-0.37	0.077	-0.085	0.045
rs73165154	G	A	3.76E-06	-0.15	0.033	-0.001	0.022
rs73192661	T	C	1.45E-09	-0.10	0.017	-0.004	0.012
rs7335748	G	A	2.41E-06	0.38	0.080	-0.047	0.041
rs766932	T	A	4.96E-07	0.10	0.019	0.008	0.013
rs7767668	T	C	9.60E-07	-0.09	0.019	-0.003	0.012
rs7861040	C	G	9.08E-07	0.08	0.017	-0.021	0.012
rs79219789	C	G	2.84E-06	-0.15	0.033	0.008	0.031
rs8084503	T	C	1.97E-07	0.14	0.027	0.011	0.019
rs847803	G	A	7.91E-07	0.09	0.018	0.009	0.013
rs9271365	G	T	1.44E-39	0.25	0.019	0.007	0.012

Supplementary Table 2. Details of Hashimoto's Thyroiditis predicting SNPs with inflammatory proteins from IEU Open GWAS (with SNPs reaching *P* < 5 x 10^{-6}).

Inflammatory proteins						Hashimoto's Thyroiditis	
SNP	Effect allele	Other allele	P value	Beta	Standard Error	log(OR)	Standard Error
rs9357119	T	G	3.65E-14	-0.15	0.020	-0.022	0.013
rs9497965	T	C	1.71E-06	0.08	0.017	-0.007	0.012
**PD-L1 (F-statistic = 36.52, R2=0.42%)**							
rs10424978	A	C	8.97E-07	-0.08	0.017	-0.030	0.012
rs10494077	A	G	9.20E-10	0.12	0.020	0.002	0.014
rs10786679	A	G	1.87E-06	-0.09	0.018	-0.004	0.013
rs111358861	G	A	3.30E-06	1.82	0.391	-0.157	0.106
rs1115587	A	G	1.82E-07	-0.10	0.018	0.011	0.012
rs11211645	G	A	9.33E-08	0.09	0.017	-0.017	0.012
rs113075322	G	C	7.18E-07	-0.14	0.028	0.001	0.033
rs11611029	T	C	1.16E-08	-0.10	0.017	0.025	0.012
rs117167029	C	G	8.05E-07	2.99	0.606	-0.030	0.089
rs117369326	A	G	3.55E-06	0.24	0.051	-0.036	0.029
rs118075239	C	T	3.45E-06	0.38	0.083	0.039	0.047
rs11822254	G	A	3.52E-06	-0.08	0.017	0.015	0.012
rs11889341	T	C	6.64E-11	0.12	0.019	-0.007	0.014
rs1317983	C	T	1.65E-07	0.09	0.018	0.019	0.013
rs13249705	G	A	3.78E-07	-0.09	0.018	-0.004	0.014
rs140111921	A	G	1.34E-06	-0.16	0.034	-0.022	0.030
rs150619443	C	G	5.08E-07	0.44	0.088	0.017	0.054
rs1534430	T	C	9.40E-08	-0.09	0.017	-0.008	0.012
rs17490285	G	A	9.26E-07	-0.18	0.038	-0.014	0.019
rs1887414	C	T	1.97E-07	0.12	0.022	0.000	0.016
rs2111485	G	A	4.19E-06	0.08	0.017	-0.019	0.012
rs229526	C	G	1.93E-06	0.10	0.022	0.005	0.015
rs2476601	G	A	3.49E-45	-0.33	0.023	0.003	0.019
rs28367132	A	G	1.21E-07	-0.14	0.026	0.012	0.015
rs28450758	A	C	4.17E-06	0.14	0.031	-0.020	0.022
rs34536443	C	G	2.47E-07	-0.25	0.049	-0.018	0.030
rs34636506	G	A	1.92E-14	-0.13	0.018	-0.014	0.012
rs61830417	G	A	3.59E-06	0.27	0.059	0.015	0.041

Supplementary Table 2. Details of Hashimoto's Thyroiditis predicting SNPs with inflammatory proteins from IEU Open GWAS (with SNPs reaching $P < 5 \times 10^{-6}$).

Inflammatory proteins				Hashimoto's Thyroiditis			
SNP	Effect allele	Other allele	P value	Beta	Standard Error	log(OR)	Standard Error
rs62076103	G	A	1.23E-07	-0.19	0.035	0.015	0.024
rs6448432	A	G	1.75E-06	0.09	0.019	-0.015	0.013
rs6501550	T	C	1.42E-06	-0.18	0.037	0.039	0.034
rs7030280	T	C	6.44E-31	0.21	0.018	-0.013	0.013
rs72808590	C	T	1.55E-06	-0.37	0.077	-0.045	0.044
rs73165154	G	A	3.76E-06	-0.15	0.033	0.024	0.022
rs73192661	T	C	1.45E-09	-0.10	0.017	0.007	0.012
rs7335748	G	A	2.41E-06	0.38	0.080	0.044	0.040
rs766932	T	A	4.96E-07	0.10	0.019	-0.003	0.013
rs7767668	T	C	9.60E-07	-0.09	0.019	0.012	0.012
rs7861040	C	G	9.08E-07	0.08	0.017	0.005	0.012
rs79219789	C	G	2.84E-06	-0.15	0.033	0.005	0.030
rs8084503	T	C	1.97E-07	0.14	0.027	0.033	0.018
rs847803	G	A	7.91E-07	0.09	0.018	0.015	0.013
rs9271365	G	T	1.44E-39	0.25	0.019	0.014	0.012
rs9357119	T	G	3.65E-14	-0.15	0.020	0.028	0.013
rs9497965	T	C	1.71E-06	0.08	0.017	-0.003	0.012
**S100A12 (F-statistic = 38.11, R2=0.44%)**							
rs10424978	A	C	8.97E-07	-0.08	0.017	-0.018	0.012
rs10494077	A	G	9.20E-10	0.12	0.020	0.026	0.014
rs10786679	A	G	1.87E-06	-0.09	0.018	-0.014	0.013
rs111358861	G	A	3.30E-06	1.82	0.391	-0.211	0.106
rs1115587	A	G	1.82E-07	-0.10	0.018	0.014	0.012
rs11211645	G	A	9.33E-08	0.09	0.017	0.009	0.012
rs113075322	G	C	7.18E-07	-0.14	0.028	-0.005	0.033
rs11611029	T	C	1.16E-08	-0.10	0.017	0.001	0.012
rs117167029	C	G	8.05E-07	2.99	0.606	0.162	0.089
rs117369326	A	G	3.55E-06	0.24	0.051	-0.003	0.029
rs118075239	C	T	3.45E-06	0.38	0.083	0.018	0.047
rs11822254	G	A	3.52E-06	-0.08	0.017	0.001	0.012
rs11889341	T	C	6.64E-11	0.12	0.019	0.010	0.014

Supplementary Table 2. Details of Hashimoto's Thyroiditis predicting SNPs with inflammatory proteins from IEU Open GWAS (with SNPs reaching $P < 5 \times 10^{-6}$).

Inflammatory proteins				Hashimoto's Thyroiditis			
SNP	Effect allele	Other allele	P value	Beta	Standard Error	log(OR)	Standard Error
rs1317983	C	T	1.65E-07	0.09	0.018	0.001	0.013
rs13249705	G	A	3.78E-07	-0.09	0.018	0.012	0.014
rs140111921	A	G	1.34E-06	-0.16	0.034	0.025	0.030
rs150619443	C	G	5.08E-07	0.44	0.088	0.035	0.055
rs1534430	T	C	9.40E-08	-0.09	0.017	0.023	0.012
rs17490285	G	A	9.26E-07	-0.18	0.038	-0.012	0.019
rs1887414	C	T	1.97E-07	0.12	0.022	-0.012	0.017
rs2111485	G	A	4.19E-06	0.08	0.017	0.005	0.012
rs229526	C	G	1.93E-06	0.10	0.022	-0.002	0.015
rs2476601	G	A	3.49E-45	-0.33	0.023	0.026	0.020
rs28367132	A	G	1.21E-07	-0.14	0.026	-0.015	0.015
rs28450758	A	C	4.17E-06	0.14	0.031	-0.014	0.023
rs3184504	C	T	1.40E-25	-0.18	0.017	-0.008	0.013
rs34536443	C	G	2.47E-07	-0.25	0.049	-0.010	0.031
rs34636506	G	A	1.92E-14	-0.13	0.018	-0.010	0.012
rs61830417	G	A	3.59E-06	0.27	0.059	0.073	0.042
rs62076103	G	A	1.23E-07	-0.19	0.035	-0.046	0.025
rs6448432	A	G	1.75E-06	0.09	0.019	0.018	0.013
rs6501550	T	C	1.42E-06	-0.18	0.037	0.010	0.035
rs7030280	T	C	6.44E-31	0.21	0.018	-0.008	0.013
rs72808590	C	T	1.55E-06	-0.37	0.077	-0.051	0.045
rs73165154	G	A	3.76E-06	-0.15	0.033	-0.022	0.022
rs73192661	T	C	1.45E-09	-0.10	0.017	-0.010	0.012
rs7335748	G	A	2.41E-06	0.38	0.080	-0.010	0.041
rs766932	T	A	4.96E-07	0.10	0.019	-0.009	0.013
rs7767668	T	C	9.60E-07	-0.09	0.019	0.023	0.012
rs7861040	C	G	9.08E-07	0.08	0.017	-0.007	0.012
rs79219789	C	G	2.84E-06	-0.15	0.033	-0.047	0.031
rs8084503	T	C	1.97E-07	0.14	0.027	0.003	0.019
rs847803	G	A	7.91E-07	0.09	0.018	0.017	0.013
rs9271365	G	T	1.44E-39	0.25	0.019	-0.004	0.012

Supplementary Table 2. Details of Hashimoto's Thyroiditis predicting SNPs with inflammatory proteins from IEU Open GWAS (with SNPs reaching *P* < 5 x 10^{-6}).

Inflammatory proteins				Hashimoto's Thyroiditis			
SNP	Effect allele	Other allele	P value	Beta	Standard Error	log(OR)	Standard Error
rs9357119	T	G	3.65E-14	-0.15	0.020	-0.009	0.013
rs9497965	T	C	1.71E-06	0.08	0.017	-0.015	0.012
**SCF (F-statistic = 38.36, R2=0.44%)**							
rs10424978	A	C	8.97E-07	-0.08	0.017	-0.020	0.012
rs10494077	A	G	9.20E-10	0.12	0.020	-0.004	0.014
rs10786679	A	G	1.87E-06	-0.09	0.018	0.038	0.013
rs111358861	G	A	3.30E-06	1.82	0.391	-0.017	0.107
rs1115587	A	G	1.82E-07	-0.10	0.018	0.003	0.012
rs11211645	G	A	9.33E-08	0.09	0.017	0.017	0.012
rs113075322	G	C	7.18E-07	-0.14	0.028	-0.003	0.033
rs11611029	T	C	1.16E-08	-0.10	0.017	-0.005	0.012
rs117167029	C	G	8.05E-07	2.99	0.606	-0.052	0.089
rs117369326	A	G	3.55E-06	0.24	0.051	0.000	0.029
rs118075239	C	T	3.45E-06	0.38	0.083	0.095	0.047
rs11822254	G	A	3.52E-06	-0.08	0.017	-0.006	0.012
rs11889341	T	C	6.64E-11	0.12	0.019	-0.037	0.014
rs1317983	C	T	1.65E-07	0.09	0.018	-0.024	0.013
rs13249705	G	A	3.78E-07	-0.09	0.018	0.004	0.014
rs140111921	A	G	1.34E-06	-0.16	0.034	-0.007	0.031
rs150619443	C	G	5.08E-07	0.44	0.088	-0.026	0.054
rs1534430	T	C	9.40E-08	-0.09	0.017	-0.012	0.012
rs17490285	G	A	9.26E-07	-0.18	0.038	-0.003	0.019
rs1887414	C	T	1.97E-07	0.12	0.022	-0.001	0.017
rs2111485	G	A	4.19E-06	0.08	0.017	-0.025	0.012
rs229526	C	G	1.93E-06	0.10	0.022	0.014	0.015
rs2476601	G	A	3.49E-45	-0.33	0.023	0.000	0.019
rs28367132	A	G	1.21E-07	-0.14	0.026	-0.003	0.015
rs28450758	A	C	4.17E-06	0.14	0.031	0.030	0.022
rs3184504	C	T	1.40E-25	-0.18	0.017	0.017	0.013
rs34636506	G	A	1.92E-14	-0.13	0.018	-0.005	0.012
rs61830417	G	A	3.59E-06	0.27	0.059	-0.061	0.041

Supplementary Table 2. Details of Hashimoto's Thyroiditis predicting SNPs with inflammatory proteins from IEU Open GWAS (with SNPs reaching *P* < 5 x 10^{-6}).

Inflammatory proteins				Hashimoto's Thyroiditis			
SNP	Effect allele	Other allele	P value	Beta	Standard Error	log(OR)	Standard Error
rs62076103	G	A	1.23E-07	-0.19	0.035	0.038	0.024
rs6448432	A	G	1.75E-06	0.09	0.019	0.003	0.013
rs6501550	T	C	1.42E-06	-0.18	0.037	-0.014	0.034
rs7030280	T	C	6.44E-31	0.21	0.018	-0.011	0.013
rs72808590	C	T	1.55E-06	-0.37	0.077	0.051	0.045
rs73165154	G	A	3.76E-06	-0.15	0.033	-0.048	0.022
rs73192661	T	C	1.45E-09	-0.10	0.017	-0.009	0.012
rs7335748	G	A	2.41E-06	0.38	0.080	-0.015	0.041
rs766932	T	A	4.96E-07	0.10	0.019	0.008	0.013
rs7767668	T	C	9.60E-07	-0.09	0.019	-0.007	0.012
rs7861040	C	G	9.08E-07	0.08	0.017	-0.001	0.012
rs79219789	C	G	2.84E-06	-0.15	0.033	-0.057	0.031
rs8084503	T	C	1.97E-07	0.14	0.027	-0.013	0.018
rs847803	G	A	7.91E-07	0.09	0.018	0.005	0.013
rs9271365	G	T	1.44E-39	0.25	0.019	0.019	0.012
rs9357119	T	G	3.65E-14	-0.15	0.020	-0.034	0.013
rs9497965	T	C	1.71E-06	0.08	0.017	-0.010	0.012
**SIRT2 (F-statistic = 38.11, R2=0.44%)**							
rs10424978	A	C	8.97E-07	-0.08	0.017	-0.007	0.012
rs10494077	A	G	9.20E-10	0.12	0.020	0.012	0.014
rs10786679	A	G	1.87E-06	-0.09	0.018	-0.010	0.014
rs111358861	G	A	3.30E-06	1.82	0.391	-0.204	0.106
rs1115587	A	G	1.82E-07	-0.10	0.018	0.009	0.012
rs11211645	G	A	9.33E-08	0.09	0.017	-0.026	0.012
rs113075322	G	C	7.18E-07	-0.14	0.028	-0.009	0.033
rs11611029	T	C	1.16E-08	-0.10	0.017	0.013	0.012
rs117167029	C	G	8.05E-07	2.99	0.606	-0.117	0.089
rs117369326	A	G	3.55E-06	0.24	0.051	-0.007	0.029
rs118075239	C	T	3.45E-06	0.38	0.083	-0.051	0.048
rs11822254	G	A	3.52E-06	-0.08	0.017	-0.014	0.012
rs11889341	T	C	6.64E-11	0.12	0.019	-0.013	0.014

Supplementary Table 2. Details of Hashimoto's Thyroiditis predicting SNPs with inflammatory proteins from IEU Open GWAS (with SNPs reaching $P < 5 \times 10^{-6}$).

Inflammatory proteins				Hashimoto's Thyroiditis			
SNP	Effect allele	Other allele	P value	Beta	Standard Error	log(OR)	Standard Error
rs1317983	C	T	1.65E-07	0.09	0.018	-0.008	0.013
rs13249705	G	A	3.78E-07	-0.09	0.018	0.003	0.014
rs140111921	A	G	1.34E-06	-0.16	0.034	0.022	0.031
rs150619443	C	G	5.08E-07	0.44	0.088	-0.075	0.054
rs1534430	T	C	9.40E-08	-0.09	0.017	0.014	0.012
rs17490285	G	A	9.26E-07	-0.18	0.038	-0.013	0.019
rs1887414	C	T	1.97E-07	0.12	0.022	0.004	0.017
rs2111485	G	A	4.19E-06	0.08	0.017	0.000	0.012
rs229526	C	G	1.93E-06	0.10	0.022	0.014	0.015
rs2476601	G	A	3.49E-45	-0.33	0.023	0.022	0.019
rs28367132	A	G	1.21E-07	-0.14	0.026	0.001	0.015
rs28450758	A	C	4.17E-06	0.14	0.031	-0.030	0.023
rs3184504	C	T	1.40E-25	-0.18	0.017	-0.017	0.013
rs34536443	C	G	2.47E-07	-0.25	0.049	-0.030	0.030
rs34636506	G	A	1.92E-14	-0.13	0.018	0.008	0.012
rs61830417	G	A	3.59E-06	0.27	0.059	0.019	0.042
rs62076103	G	A	1.23E-07	-0.19	0.035	0.021	0.025
rs6448432	A	G	1.75E-06	0.09	0.019	0.005	0.013
rs6501550	T	C	1.42E-06	-0.18	0.037	0.015	0.035
rs7030280	T	C	6.44E-31	0.21	0.018	0.001	0.013
rs72808590	C	T	1.55E-06	-0.37	0.077	0.017	0.045
rs73165154	G	A	3.76E-06	-0.15	0.033	-0.018	0.022
rs73192661	T	C	1.45E-09	-0.10	0.017	-0.023	0.012
rs7335748	G	A	2.41E-06	0.38	0.080	0.024	0.041
rs766932	T	A	4.96E-07	0.10	0.019	-0.021	0.013
rs7767668	T	C	9.60E-07	-0.09	0.019	-0.005	0.012
rs7861040	C	G	9.08E-07	0.08	0.017	-0.007	0.012
rs79219789	C	G	2.84E-06	-0.15	0.033	-0.013	0.031
rs8084503	T	C	1.97E-07	0.14	0.027	0.008	0.018
rs847803	G	A	7.91E-07	0.09	0.018	0.006	0.013
rs9271365	G	T	1.44E-39	0.25	0.019	0.005	0.012

Supplementary Table 2. Details of Hashimoto's Thyroiditis predicting SNPs with inflammatory proteins from IEU Open GWAS (with SNPs reaching $P < 5 \times 10^{-6}$).

Inflammatory proteins				Hashimoto's Thyroiditis			
SNP	Effect allele	Other allele	P value	Beta	Standard Error	log(OR)	Standard Error
rs9357119	T	G	3.65E-14	-0.15	0.020	-0.002	0.013
rs9497965	T	C	1.71E-06	0.08	0.017	-0.012	0.012
**SLAM (F-statistic = 36.52, R2=0.42%)**							
rs10424978	A	C	8.97E-07	-0.08	0.017	-0.009	0.012
rs10494077	A	G	9.20E-10	0.12	0.020	-0.006	0.014
rs10786679	A	G	1.87E-06	-0.09	0.018	-0.006	0.013
rs111358861	G	A	3.30E-06	1.82	0.391	-0.039	0.106
rs1115587	A	G	1.82E-07	-0.10	0.018	-0.003	0.012
rs11211645	G	A	9.33E-08	0.09	0.017	0.000	0.012
rs113075322	G	C	7.18E-07	-0.14	0.028	0.014	0.033
rs11611029	T	C	1.16E-08	-0.10	0.017	0.008	0.012
rs117167029	C	G	8.05E-07	2.99	0.606	-0.259	0.089
rs117369326	A	G	3.55E-06	0.24	0.051	-0.001	0.029
rs118075239	C	T	3.45E-06	0.38	0.083	0.124	0.047
rs11822254	G	A	3.52E-06	-0.08	0.017	-0.012	0.012
rs11889341	T	C	6.64E-11	0.12	0.019	-0.003	0.014
rs1317983	C	T	1.65E-07	0.09	0.018	0.023	0.013
rs13249705	G	A	3.78E-07	-0.09	0.018	-0.021	0.014
rs140111921	A	G	1.34E-06	-0.16	0.034	-0.009	0.031
rs150619443	C	G	5.08E-07	0.44	0.088	-0.042	0.054
rs1534430	T	C	9.40E-08	-0.09	0.017	0.005	0.012
rs17490285	G	A	9.26E-07	-0.18	0.038	-0.017	0.019
rs1887414	C	T	1.97E-07	0.12	0.022	-0.024	0.017
rs2111485	G	A	4.19E-06	0.08	0.017	-0.010	0.012
rs229526	C	G	1.93E-06	0.10	0.022	0.018	0.015
rs2476601	G	A	3.49E-45	-0.33	0.023	0.020	0.019
rs28367132	A	G	1.21E-07	-0.14	0.026	-0.020	0.015
rs28450758	A	C	4.17E-06	0.14	0.031	-0.014	0.023
rs34536443	C	G	2.47E-07	-0.25	0.049	-0.016	0.030
rs34636506	G	A	1.92E-14	-0.13	0.018	-0.017	0.012
rs61830417	G	A	3.59E-06	0.27	0.059	0.022	0.042

Supplementary Table 2. Details of Hashimoto's Thyroiditis predicting SNPs with inflammatory proteins from IEU Open GWAS (with SNPs reaching $P < 5 \times 10^{-6}$).

Inflammatory proteins				Hashimoto's Thyroiditis			
SNP	Effect allele	Other allele	P value	Beta	Standard Error	log(OR)	Standard Error
rs62076103	G	A	1.23E-07	-0.19	0.035	0.034	0.024
rs6448432	A	G	1.75E-06	0.09	0.019	0.012	0.013
rs6501550	T	C	1.42E-06	-0.18	0.037	0.053	0.034
rs7030280	T	C	6.44E-31	0.21	0.018	-0.020	0.013
rs72808590	C	T	1.55E-06	-0.37	0.077	-0.023	0.045
rs73165154	G	A	3.76E-06	-0.15	0.033	0.050	0.022
rs73192661	T	C	1.45E-09	-0.10	0.017	-0.005	0.012
rs7335748	G	A	2.41E-06	0.38	0.080	-0.020	0.041
rs766932	T	A	4.96E-07	0.10	0.019	-0.009	0.013
rs7767668	T	C	9.60E-07	-0.09	0.019	0.019	0.012
rs7861040	C	G	9.08E-07	0.08	0.017	-0.003	0.012
rs79219789	C	G	2.84E-06	-0.15	0.033	0.031	0.031
rs8084503	T	C	1.97E-07	0.14	0.027	0.028	0.018
rs847803	G	A	7.91E-07	0.09	0.018	-0.011	0.013
rs9271365	G	T	1.44E-39	0.25	0.019	0.006	0.012
rs9357119	T	G	3.65E-14	-0.15	0.020	0.024	0.013
rs9497965	T	C	1.71E-06	0.08	0.017	-0.004	0.012
**STAMBP (F-statistic = 38.11, R2=0.44%)**							
rs10424978	A	C	8.97E-07	-0.08	0.017	-0.001	0.012
rs10494077	A	G	9.20E-10	0.12	0.020	0.008	0.014
rs10786679	A	G	1.87E-06	-0.09	0.018	-0.010	0.014
rs111358861	G	A	3.30E-06	1.82	0.391	-0.178	0.107
rs1115587	A	G	1.82E-07	-0.10	0.018	-0.003	0.012
rs11211645	G	A	9.33E-08	0.09	0.017	-0.023	0.012
rs113075322	G	C	7.18E-07	-0.14	0.028	-0.019	0.033
rs11611029	T	C	1.16E-08	-0.10	0.017	0.009	0.012
rs117167029	C	G	8.05E-07	2.99	0.606	-0.081	0.090
rs117369326	A	G	3.55E-06	0.24	0.051	-0.009	0.029
rs118075239	C	T	3.45E-06	0.38	0.083	-0.060	0.047
rs11822254	G	A	3.52E-06	-0.08	0.017	-0.016	0.012
rs11889341	T	C	6.64E-11	0.12	0.019	-0.009	0.014

Supplementary Table 2. Details of Hashimoto's Thyroiditis predicting SNPs with inflammatory proteins from IEU Open GWAS (with SNPs reaching $P < 5 \times 10^{-6}$).

Inflammatory proteins				Hashimoto's Thyroiditis			
SNP	Effect allele	Other allele	P value	Beta	Standard Error	log(OR)	Standard Error
rs1317983	C	T	1.65E-07	0.09	0.018	-0.013	0.013
rs13249705	G	A	3.78E-07	-0.09	0.018	0.003	0.014
rs140111921	A	G	1.34E-06	-0.16	0.034	0.015	0.031
rs150619443	C	G	5.08E-07	0.44	0.088	-0.078	0.054
rs1534430	T	C	9.40E-08	-0.09	0.017	0.013	0.012
rs17490285	G	A	9.26E-07	-0.18	0.038	-0.005	0.019
rs1887414	C	T	1.97E-07	0.12	0.022	0.003	0.016
rs2111485	G	A	4.19E-06	0.08	0.017	-0.003	0.012
rs229526	C	G	1.93E-06	0.10	0.022	0.007	0.015
rs2476601	G	A	3.49E-45	-0.33	0.023	0.013	0.019
rs28367132	A	G	1.21E-07	-0.14	0.026	0.007	0.015
rs28450758	A	C	4.17E-06	0.14	0.031	-0.020	0.022
rs3184504	C	T	1.40E-25	-0.18	0.017	-0.012	0.013
rs34536443	C	G	2.47E-07	-0.25	0.049	-0.030	0.030
rs34636506	G	A	1.92E-14	-0.13	0.018	0.001	0.012
rs61830417	G	A	3.59E-06	0.27	0.059	-0.010	0.041
rs62076103	G	A	1.23E-07	-0.19	0.035	0.005	0.024
rs6448432	A	G	1.75E-06	0.09	0.019	0.002	0.013
rs6501550	T	C	1.42E-06	-0.18	0.037	0.029	0.035
rs7030280	T	C	6.44E-31	0.21	0.018	-0.001	0.013
rs72808590	C	T	1.55E-06	-0.37	0.077	0.028	0.044
rs73165154	G	A	3.76E-06	-0.15	0.033	-0.004	0.022
rs73192661	T	C	1.45E-09	-0.10	0.017	-0.023	0.012
rs7335748	G	A	2.41E-06	0.38	0.080	0.025	0.041
rs766932	T	A	4.96E-07	0.10	0.019	-0.014	0.013
rs7767668	T	C	9.60E-07	-0.09	0.019	-0.008	0.012
rs7861040	C	G	9.08E-07	0.08	0.017	0.002	0.012
rs79219789	C	G	2.84E-06	-0.15	0.033	-0.013	0.031
rs8084503	T	C	1.97E-07	0.14	0.027	0.007	0.018
rs847803	G	A	7.91E-07	0.09	0.018	0.012	0.013
rs9271365	G	T	1.44E-39	0.25	0.019	0.009	0.012

Supplementary Table 2. Details of Hashimoto's Thyroiditis predicting SNPs with inflammatory proteins from IEU Open GWAS (with SNPs reaching $P < 5 \times 10^{-6}$).

Inflammatory proteins				Hashimoto's Thyroiditis			
SNP	Effect allele	Other allele	P value	Beta	Standard Error	log(OR)	Standard Error
rs9357119	T	G	3.65E-14	-0.15	0.020	0.000	0.013
rs9497965	T	C	1.71E-06	0.08	0.017	-0.016	0.012
**SULT1A1 (F-statistic = 38.11, R2=0.44%)**							
rs10424978	A	C	8.97E-07	-0.08	0.017	0.007	0.014
rs10494077	A	G	9.20E-10	0.12	0.020	0.002	0.016
rs10786679	A	G	1.87E-06	-0.09	0.018	-0.028	0.015
rs111358861	G	A	3.30E-06	1.82	0.391	-0.226	0.107
rs1115587	A	G	1.82E-07	-0.10	0.018	0.029	0.014
rs11211645	G	A	9.33E-08	0.09	0.017	-0.011	0.014
rs113075322	G	C	7.18E-07	-0.14	0.028	-0.038	0.036
rs11611029	T	C	1.16E-08	-0.10	0.017	0.009	0.014
rs117167029	C	G	8.05E-07	2.99	0.606	-0.065	0.111
rs117369326	A	G	3.55E-06	0.24	0.051	-0.056	0.033
rs118075239	C	T	3.45E-06	0.38	0.083	-0.047	0.052
rs11822254	G	A	3.52E-06	-0.08	0.017	-0.008	0.014
rs11889341	T	C	6.64E-11	0.12	0.019	0.014	0.016
rs1317983	C	T	1.65E-07	0.09	0.018	-0.008	0.015
rs13249705	G	A	3.78E-07	-0.09	0.018	-0.015	0.016
rs140111921	A	G	1.34E-06	-0.16	0.034	-0.002	0.034
rs150619443	C	G	5.08E-07	0.44	0.088	-0.017	0.059
rs1534430	T	C	9.40E-08	-0.09	0.017	0.008	0.014
rs17490285	G	A	9.26E-07	-0.18	0.038	-0.015	0.021
rs1887414	C	T	1.97E-07	0.12	0.022	-0.020	0.019
rs2111485	G	A	4.19E-06	0.08	0.017	0.007	0.014
rs229526	C	G	1.93E-06	0.10	0.022	-0.012	0.017
rs2476601	G	A	3.49E-45	-0.33	0.023	0.049	0.022
rs28367132	A	G	1.21E-07	-0.14	0.026	-0.002	0.017
rs28450758	A	C	4.17E-06	0.14	0.031	-0.027	0.025
rs3184504	C	T	1.40E-25	-0.18	0.017	-0.012	0.013
rs34536443	C	G	2.47E-07	-0.25	0.049	-0.049	0.034
rs34636506	G	A	1.92E-14	-0.13	0.018	0.000	0.014

Supplementary Table 2. Details of Hashimoto's Thyroiditis predicting SNPs with inflammatory proteins from IEU Open GWAS (with SNPs reaching $P < 5 \times 10^{-6}$).

Inflammatory proteins				Hashimoto's Thyroiditis			
SNP	Effect allele	Other allele	P value	Beta	Standard Error	log(OR)	Standard Error
rs61830417	G	A	3.59E-06	0.27	0.059	0.029	0.046
rs62076103	G	A	1.23E-07	-0.19	0.035	0.006	0.027
rs6448432	A	G	1.75E-06	0.09	0.019	0.019	0.015
rs6501550	T	C	1.42E-06	-0.18	0.037	0.002	0.040
rs7030280	T	C	6.44E-31	0.21	0.018	0.009	0.014
rs72808590	C	T	1.55E-06	-0.37	0.077	0.067	0.049
rs73165154	G	A	3.76E-06	-0.15	0.033	-0.011	0.024
rs73192661	T	C	1.45E-09	-0.10	0.017	-0.026	0.014
rs7335748	G	A	2.41E-06	0.38	0.080	0.004	0.047
rs766932	T	A	4.96E-07	0.10	0.019	-0.023	0.015
rs7767668	T	C	9.60E-07	-0.09	0.019	-0.004	0.014
rs7861040	C	G	9.08E-07	0.08	0.017	-0.011	0.014
rs79219789	C	G	2.84E-06	-0.15	0.033	0.004	0.036
rs8084503	T	C	1.97E-07	0.14	0.027	0.000	0.021
rs847803	G	A	7.91E-07	0.09	0.018	0.015	0.014
rs9271365	G	T	1.44E-39	0.25	0.019	-0.004	0.014
rs9357119	T	G	3.65E-14	-0.15	0.020	-0.005	0.016
rs9497965	T	C	1.71E-06	0.08	0.017	0.004	0.014
**TGF-α (F-statistic = 36.52, R2=0.42%)**							
rs10424978	A	C	8.97E-07	-0.08	0.017	-0.017	0.012
rs10494077	A	G	9.20E-10	0.12	0.020	-0.009	0.014
rs10786679	A	G	1.87E-06	-0.09	0.018	-0.012	0.013
rs111358861	G	A	3.30E-06	1.82	0.391	-0.149	0.107
rs1115587	A	G	1.82E-07	-0.10	0.018	0.022	0.012
rs11211645	G	A	9.33E-08	0.09	0.017	-0.010	0.012
rs113075322	G	C	7.18E-07	-0.14	0.028	-0.007	0.033
rs11611029	T	C	1.16E-08	-0.10	0.017	-0.004	0.012
rs117167029	C	G	8.05E-07	2.99	0.606	0.069	0.089
rs117369326	A	G	3.55E-06	0.24	0.051	0.002	0.029
rs118075239	C	T	3.45E-06	0.38	0.083	0.048	0.047
rs11822254	G	A	3.52E-06	-0.08	0.017	0.003	0.012

Supplementary Table 2. Details of Hashimoto's Thyroiditis predicting SNPs with inflammatory proteins from IEU Open GWAS (with SNPs reaching $P < 5 \times 10^{-6}$).

Inflammatory proteins				Hashimoto's Thyroiditis			
SNP	Effect allele	Other allele	P value	Beta	Standard Error	log(OR)	Standard Error
rs11889341	T	C	6.64E-11	0.12	0.019	0.027	0.014
rs1317983	C	T	1.65E-07	0.09	0.018	0.009	0.013
rs13249705	G	A	3.78E-07	-0.09	0.018	0.026	0.014
rs140111921	A	G	1.34E-06	-0.16	0.034	-0.033	0.030
rs150619443	C	G	5.08E-07	0.44	0.088	-0.019	0.054
rs1534430	T	C	9.40E-08	-0.09	0.017	0.011	0.012
rs17490285	G	A	9.26E-07	-0.18	0.038	-0.002	0.019
rs1887414	C	T	1.97E-07	0.12	0.022	-0.019	0.017
rs2111485	G	A	4.19E-06	0.08	0.017	0.014	0.012
rs229526	C	G	1.93E-06	0.10	0.022	-0.009	0.015
rs2476601	G	A	3.49E-45	-0.33	0.023	0.008	0.019
rs28367132	A	G	1.21E-07	-0.14	0.026	-0.013	0.015
rs28450758	A	C	4.17E-06	0.14	0.031	-0.025	0.022
rs34536443	C	G	2.47E-07	-0.25	0.049	-0.001	0.030
rs34636506	G	A	1.92E-14	-0.13	0.018	-0.009	0.012
rs61830417	G	A	3.59E-06	0.27	0.059	0.054	0.041
rs62076103	G	A	1.23E-07	-0.19	0.035	-0.008	0.024
rs6448432	A	G	1.75E-06	0.09	0.019	0.013	0.013
rs6501550	T	C	1.42E-06	-0.18	0.037	0.043	0.035
rs7030280	T	C	6.44E-31	0.21	0.018	-0.010	0.013
rs72808590	C	T	1.55E-06	-0.37	0.077	-0.123	0.044
rs73165154	G	A	3.76E-06	-0.15	0.033	-0.014	0.022
rs73192661	T	C	1.45E-09	-0.10	0.017	-0.006	0.012
rs7335748	G	A	2.41E-06	0.38	0.080	-0.027	0.041
rs766932	T	A	4.96E-07	0.10	0.019	0.003	0.013
rs7767668	T	C	9.60E-07	-0.09	0.019	0.006	0.012
rs7861040	C	G	9.08E-07	0.08	0.017	-0.018	0.012
rs79219789	C	G	2.84E-06	-0.15	0.033	-0.055	0.031
rs8084503	T	C	1.97E-07	0.14	0.027	0.012	0.018
rs847803	G	A	7.91E-07	0.09	0.018	0.009	0.013
rs9271365	G	T	1.44E-39	0.25	0.019	0.016	0.012

Supplementary Table 2. Details of Hashimoto's Thyroiditis predicting SNPs with inflammatory proteins from IEU Open GWAS (with SNPs reaching $P < 5 \times 10^{-6}$).

Inflammatory proteins				Hashimoto's Thyroiditis			
SNP	Effect allele	Other allele	P value	Beta	Standard Error	log(OR)	Standard Error
rs9357119	T	G	3.65E-14	-0.15	0.020	-0.017	0.013
rs9497965	T	C	1.71E-06	0.08	0.017	-0.013	0.012
**TNF (F-statistic = 38.11, R2=0.44%)**							
rs10424978	A	C	8.97E-07	-0.08	0.017	-0.024	0.014
rs10494077	A	G	9.20E-10	0.12	0.020	-0.025	0.016
rs10786679	A	G	1.87E-06	-0.09	0.018	0.003	0.015
rs111358861	G	A	3.30E-06	1.82	0.391	0.161	0.107
rs1115587	A	G	1.82E-07	-0.10	0.018	-0.006	0.014
rs11211645	G	A	9.33E-08	0.09	0.017	-0.007	0.013
rs113075322	G	C	7.18E-07	-0.14	0.028	-0.011	0.036
rs11611029	T	C	1.16E-08	-0.10	0.017	-0.002	0.014
rs117167029	C	G	8.05E-07	2.99	0.606	0.126	0.111
rs117369326	A	G	3.55E-06	0.24	0.051	-0.021	0.033
rs118075239	C	T	3.45E-06	0.38	0.083	-0.032	0.051
rs11822254	G	A	3.52E-06	-0.08	0.017	-0.014	0.014
rs11889341	T	C	6.64E-11	0.12	0.019	0.006	0.016
rs1317983	C	T	1.65E-07	0.09	0.018	0.013	0.015
rs13249705	G	A	3.78E-07	-0.09	0.018	-0.020	0.016
rs140111921	A	G	1.34E-06	-0.16	0.034	-0.038	0.033
rs150619443	C	G	5.08E-07	0.44	0.088	-0.033	0.058
rs1534430	T	C	9.40E-08	-0.09	0.017	-0.024	0.014
rs17490285	G	A	9.26E-07	-0.18	0.038	0.002	0.021
rs1887414	C	T	1.97E-07	0.12	0.022	-0.023	0.018
rs2111485	G	A	4.19E-06	0.08	0.017	0.003	0.013
rs229526	C	G	1.93E-06	0.10	0.022	-0.023	0.016
rs2476601	G	A	3.49E-45	-0.33	0.023	-0.025	0.021
rs28367132	A	G	1.21E-07	-0.14	0.026	0.000	0.017
rs28450758	A	C	4.17E-06	0.14	0.031	-0.029	0.025
rs3184504	C	T	1.40E-25	-0.18	0.017	0.006	0.013
rs34536443	C	G	2.47E-07	-0.25	0.049	-0.028	0.034
rs34636506	G	A	1.92E-14	-0.13	0.018	-0.002	0.014

Supplementary Table 2. Details of Hashimoto's Thyroiditis predicting SNPs with inflammatory proteins from IEU Open GWAS (with SNPs reaching $P < 5 \times 10^{-6}$).

Inflammatory proteins				Hashimoto's Thyroiditis			
SNP	Effect allele	Other allele	P value	Beta	Standard Error	log(OR)	Standard Error
rs61830417	G	A	3.59E-06	0.27	0.059	0.115	0.046
rs62076103	G	A	1.23E-07	-0.19	0.035	-0.030	0.026
rs6448432	A	G	1.75E-06	0.09	0.019	-0.009	0.014
rs6501550	T	C	1.42E-06	-0.18	0.037	-0.002	0.040
rs7030280	T	C	6.44E-31	0.21	0.018	0.023	0.014
rs72808590	C	T	1.55E-06	-0.37	0.077	0.039	0.048
rs73165154	G	A	3.76E-06	-0.15	0.033	0.010	0.024
rs73192661	T	C	1.45E-09	-0.10	0.017	0.002	0.014
rs7335748	G	A	2.41E-06	0.38	0.080	0.028	0.046
rs766932	T	A	4.96E-07	0.10	0.019	0.011	0.015
rs7767668	T	C	9.60E-07	-0.09	0.019	0.001	0.013
rs7861040	C	G	9.08E-07	0.08	0.017	0.006	0.013
rs79219789	C	G	2.84E-06	-0.15	0.033	-0.008	0.035
rs8084503	T	C	1.97E-07	0.14	0.027	-0.007	0.021
rs847803	G	A	7.91E-07	0.09	0.018	0.008	0.014
rs9271365	G	T	1.44E-39	0.25	0.019	-0.007	0.013
rs9357119	T	G	3.65E-14	-0.15	0.020	0.008	0.015
rs9497965	T	C	1.71E-06	0.08	0.017	-0.004	0.013
**TNFRSF9 (F-statistic = 36.52, R2=0.42%)**							
rs10424978	A	C	8.97E-07	-0.08	0.017	-0.021	0.014
rs10494077	A	G	9.20E-10	0.12	0.020	-0.011	0.016
rs10786679	A	G	1.87E-06	-0.09	0.018	-0.003	0.015
rs111358861	G	A	3.30E-06	1.82	0.391	-0.078	0.106
rs1115587	A	G	1.82E-07	-0.10	0.018	-0.010	0.014
rs11211645	G	A	9.33E-08	0.09	0.017	-0.010	0.013
rs113075322	G	C	7.18E-07	-0.14	0.028	-0.010	0.036
rs11611029	T	C	1.16E-08	-0.10	0.017	0.002	0.014
rs117167029	C	G	8.05E-07	2.99	0.606	-0.129	0.111
rs117369326	A	G	3.55E-06	0.24	0.051	-0.030	0.033
rs118075239	C	T	3.45E-06	0.38	0.083	0.070	0.051
rs11822254	G	A	3.52E-06	-0.08	0.017	0.000	0.014

Supplementary Table 2. Details of Hashimoto's Thyroiditis predicting SNPs with inflammatory proteins from IEU Open GWAS (with SNPs reaching $P < 5 \times 10^{-6}$).

Inflammatory proteins				Hashimoto's Thyroiditis			
SNP	Effect allele	Other allele	P value	Beta	Standard Error	log(OR)	Standard Error
rs11889341	T	C	6.64E-11	0.12	0.019	0.011	0.016
rs1317983	C	T	1.65E-07	0.09	0.018	0.006	0.015
rs13249705	G	A	3.78E-07	-0.09	0.018	-0.006	0.016
rs140111921	A	G	1.34E-06	-0.16	0.034	-0.003	0.033
rs150619443	C	G	5.08E-07	0.44	0.088	-0.069	0.058
rs1534430	T	C	9.40E-08	-0.09	0.017	-0.009	0.014
rs17490285	G	A	9.26E-07	-0.18	0.038	-0.017	0.021
rs1887414	C	T	1.97E-07	0.12	0.022	-0.001	0.019
rs2111485	G	A	4.19E-06	0.08	0.017	0.004	0.013
rs229526	C	G	1.93E-06	0.10	0.022	-0.009	0.017
rs2476601	G	A	3.49E-45	-0.33	0.023	0.004	0.021
rs28367132	A	G	1.21E-07	-0.14	0.026	0.006	0.017
rs28450758	A	C	4.17E-06	0.14	0.031	0.022	0.025
rs34536443	C	G	2.47E-07	-0.25	0.049	-0.023	0.034
rs34636506	G	A	1.92E-14	-0.13	0.018	-0.046	0.014
rs61830417	G	A	3.59E-06	0.27	0.059	0.094	0.046
rs62076103	G	A	1.23E-07	-0.19	0.035	-0.034	0.026
rs6448432	A	G	1.75E-06	0.09	0.019	0.012	0.014
rs6501550	T	C	1.42E-06	-0.18	0.037	0.034	0.040
rs7030280	T	C	6.44E-31	0.21	0.018	-0.018	0.014
rs72808590	C	T	1.55E-06	-0.37	0.077	-0.098	0.048
rs73165154	G	A	3.76E-06	-0.15	0.033	-0.003	0.024
rs73192661	T	C	1.45E-09	-0.10	0.017	0.009	0.014
rs7335748	G	A	2.41E-06	0.38	0.080	-0.001	0.046
rs766932	T	A	4.96E-07	0.10	0.019	-0.017	0.015
rs7767668	T	C	9.60E-07	-0.09	0.019	-0.034	0.013
rs7861040	C	G	9.08E-07	0.08	0.017	0.008	0.013
rs79219789	C	G	2.84E-06	-0.15	0.033	0.008	0.035
rs8084503	T	C	1.97E-07	0.14	0.027	0.020	0.021
rs847803	G	A	7.91E-07	0.09	0.018	0.036	0.014
rs9271365	G	T	1.44E-39	0.25	0.019	0.032	0.013

Supplementary Table 2. Details of Hashimoto's Thyroiditis predicting SNPs with inflammatory proteins from IEU Open GWAS (with SNPs reaching $P < 5 \times 10^{-6}$).

Inflammatory proteins				Hashimoto's Thyroiditis			
SNP	Effect allele	Other allele	P value	Beta	Standard Error	log(OR)	Standard Error
rs9357119	T	G	3.65E-14	-0.15	0.020	-0.031	0.015
rs9497965	T	C	1.71E-06	0.08	0.017	-0.008	0.013
**TNFSF12 (F-statistic = 38.11, R2=0.44%)**							
rs10424978	A	C	8.97E-07	-0.08	0.017	-0.018	0.012
rs10494077	A	G	9.20E-10	0.12	0.020	0.001	0.014
rs10786679	A	G	1.87E-06	-0.09	0.018	-0.005	0.014
rs111358861	G	A	3.30E-06	1.82	0.391	-0.033	0.106
rs1115587	A	G	1.82E-07	-0.10	0.018	0.021	0.012
rs11211645	G	A	9.33E-08	0.09	0.017	-0.003	0.012
rs113075322	G	C	7.18E-07	-0.14	0.028	-0.058	0.033
rs11611029	T	C	1.16E-08	-0.10	0.017	-0.013	0.012
rs117167029	C	G	8.05E-07	2.99	0.606	0.081	0.090
rs117369326	A	G	3.55E-06	0.24	0.051	-0.011	0.029
rs118075239	C	T	3.45E-06	0.38	0.083	0.033	0.047
rs11822254	G	A	3.52E-06	-0.08	0.017	0.011	0.012
rs11889341	T	C	6.64E-11	0.12	0.019	0.000	0.014
rs1317983	C	T	1.65E-07	0.09	0.018	-0.007	0.013
rs13249705	G	A	3.78E-07	-0.09	0.018	-0.028	0.014
rs140111921	A	G	1.34E-06	-0.16	0.034	-0.042	0.031
rs150619443	C	G	5.08E-07	0.44	0.088	-0.056	0.054
rs1534430	T	C	9.40E-08	-0.09	0.017	-0.006	0.012
rs17490285	G	A	9.26E-07	-0.18	0.038	-0.002	0.019
rs1887414	C	T	1.97E-07	0.12	0.022	-0.005	0.016
rs2111485	G	A	4.19E-06	0.08	0.017	0.012	0.012
rs229526	C	G	1.93E-06	0.10	0.022	0.010	0.015
rs2476601	G	A	3.49E-45	-0.33	0.023	0.028	0.019
rs28367132	A	G	1.21E-07	-0.14	0.026	-0.025	0.015
rs28450758	A	C	4.17E-06	0.14	0.031	-0.010	0.022
rs3184504	C	T	1.40E-25	-0.18	0.017	-0.025	0.013
rs34536443	C	G	2.47E-07	-0.25	0.049	0.025	0.030
rs34636506	G	A	1.92E-14	-0.13	0.018	-0.005	0.012

Supplementary Table 2. Details of Hashimoto's Thyroiditis predicting SNPs with inflammatory proteins from IEU Open GWAS (with SNPs reaching $P < 5 \times 10^{-6}$).

Inflammatory proteins				Hashimoto's Thyroiditis			
SNP	Effect allele	Other allele	P value	Beta	Standard Error	log(OR)	Standard Error
rs61830417	G	A	3.59E-06	0.27	0.059	-0.016	0.041
rs62076103	G	A	1.23E-07	-0.19	0.035	0.015	0.024
rs6448432	A	G	1.75E-06	0.09	0.019	0.001	0.013
rs6501550	T	C	1.42E-06	-0.18	0.037	-0.030	0.035
rs7030280	T	C	6.44E-31	0.21	0.018	0.010	0.013
rs72808590	C	T	1.55E-06	-0.37	0.077	0.028	0.044
rs73165154	G	A	3.76E-06	-0.15	0.033	0.003	0.022
rs73192661	T	C	1.45E-09	-0.10	0.017	0.001	0.012
rs7335748	G	A	2.41E-06	0.38	0.080	-0.061	0.040
rs766932	T	A	4.96E-07	0.10	0.019	0.012	0.013
rs7767668	T	C	9.60E-07	-0.09	0.019	-0.010	0.012
rs7861040	C	G	9.08E-07	0.08	0.017	-0.005	0.012
rs79219789	C	G	2.84E-06	-0.15	0.033	-0.012	0.030
rs8084503	T	C	1.97E-07	0.14	0.027	0.023	0.018
rs847803	G	A	7.91E-07	0.09	0.018	0.003	0.013
rs9271365	G	T	1.44E-39	0.25	0.019	-0.006	0.012
rs9357119	T	G	3.65E-14	-0.15	0.020	-0.010	0.013
rs9497965	T	C	1.71E-06	0.08	0.017	-0.003	0.012
**TNFSF14 (F-statistic = 38.11, R2=0.44%)**							
rs10424978	A	C	8.97E-07	-0.08	0.017	-0.020	0.014
rs10494077	A	G	9.20E-10	0.12	0.020	-0.008	0.016
rs10786679	A	G	1.87E-06	-0.09	0.018	-0.022	0.015
rs111358861	G	A	3.30E-06	1.82	0.391	-0.304	0.106
rs1115587	A	G	1.82E-07	-0.10	0.018	0.029	0.014
rs11211645	G	A	9.33E-08	0.09	0.017	-0.021	0.013
rs113075322	G	C	7.18E-07	-0.14	0.028	-0.073	0.036
rs11611029	T	C	1.16E-08	-0.10	0.017	-0.002	0.014
rs117167029	C	G	8.05E-07	2.99	0.606	0.047	0.111
rs117369326	A	G	3.55E-06	0.24	0.051	-0.035	0.033
rs118075239	C	T	3.45E-06	0.38	0.083	-0.004	0.051
rs11822254	G	A	3.52E-06	-0.08	0.017	0.003	0.014

Supplementary Table 2. Details of Hashimoto's Thyroiditis predicting SNPs with inflammatory proteins from IEU Open GWAS (with SNPs reaching $P < 5 \times 10^{-6}$).

Inflammatory proteins				Hashimoto's Thyroiditis			
SNP	Effect allele	Other allele	P value	Beta	Standard Error	log(OR)	Standard Error
rs11889341	T	C	6.64E-11	0.12	0.019	0.017	0.016
rs1317983	C	T	1.65E-07	0.09	0.018	0.000	0.015
rs13249705	G	A	3.78E-07	-0.09	0.018	-0.019	0.016
rs140111921	A	G	1.34E-06	-0.16	0.034	0.011	0.033
rs150619443	C	G	5.08E-07	0.44	0.088	-0.080	0.058
rs1534430	T	C	9.40E-08	-0.09	0.017	0.020	0.014
rs17490285	G	A	9.26E-07	-0.18	0.038	0.000	0.021
rs1887414	C	T	1.97E-07	0.12	0.022	-0.018	0.019
rs2111485	G	A	4.19E-06	0.08	0.017	0.013	0.013
rs229526	C	G	1.93E-06	0.10	0.022	0.005	0.017
rs2476601	G	A	3.49E-45	-0.33	0.023	0.054	0.021
rs28367132	A	G	1.21E-07	-0.14	0.026	-0.034	0.017
rs28450758	A	C	4.17E-06	0.14	0.031	-0.046	0.025
rs3184504	C	T	1.40E-25	-0.18	0.017	-0.033	0.013
rs34536443	C	G	2.47E-07	-0.25	0.049	-0.007	0.034
rs34636506	G	A	1.92E-14	-0.13	0.018	-0.011	0.014
rs61830417	G	A	3.59E-06	0.27	0.059	0.040	0.046
rs62076103	G	A	1.23E-07	-0.19	0.035	0.004	0.027
rs6448432	A	G	1.75E-06	0.09	0.019	0.029	0.015
rs6501550	T	C	1.42E-06	-0.18	0.037	0.015	0.040
rs7030280	T	C	6.44E-31	0.21	0.018	-0.008	0.014
rs72808590	C	T	1.55E-06	-0.37	0.077	-0.013	0.049
rs73165154	G	A	3.76E-06	-0.15	0.033	-0.002	0.024
rs73192661	T	C	1.45E-09	-0.10	0.017	-0.012	0.014
rs7335748	G	A	2.41E-06	0.38	0.080	-0.043	0.047
rs766932	T	A	4.96E-07	0.10	0.019	-0.003	0.015
rs7767668	T	C	9.60E-07	-0.09	0.019	-0.006	0.013
rs7861040	C	G	9.08E-07	0.08	0.017	-0.015	0.014
rs79219789	C	G	2.84E-06	-0.15	0.033	0.032	0.036
rs8084503	T	C	1.97E-07	0.14	0.027	-0.002	0.021
rs847803	G	A	7.91E-07	0.09	0.018	0.011	0.014

Supplementary Table 2. Details of Hashimoto's Thyroiditis predicting SNPs with inflammatory proteins from IEU Open GWAS (with SNPs reaching $P < 5 \times 10^{-6}$).

Inflammatory proteins				Hashimoto's Thyroiditis			
SNP	Effect allele	Other allele	P value	Beta	Standard Error	log(OR)	Standard Error
rs9271365	G	T	1.44E-39	0.25	0.019	-0.004	0.013
rs9357119	T	G	3.65E-14	-0.15	0.020	-0.022	0.015
rs9497965	T	C	1.71E-06	0.08	0.017	-0.017	0.014
**TRAIL (F-statistic = 38.11, R2=0.44%)**							
rs10424978	A	C	8.97E-07	-0.08	0.017	0.000	0.012
rs10494077	A	G	9.20E-10	0.12	0.020	0.006	0.014
rs10786679	A	G	1.87E-06	-0.09	0.018	-0.006	0.014
rs111358861	G	A	3.30E-06	1.82	0.391	-0.060	0.107
rs1115587	A	G	1.82E-07	-0.10	0.018	0.016	0.012
rs11211645	G	A	9.33E-08	0.09	0.017	-0.013	0.012
rs113075322	G	C	7.18E-07	-0.14	0.028	-0.074	0.033
rs11611029	T	C	1.16E-08	-0.10	0.017	-0.006	0.012
rs117167029	C	G	8.05E-07	2.99	0.606	0.023	0.090
rs117369326	A	G	3.55E-06	0.24	0.051	0.011	0.029
rs118075239	C	T	3.45E-06	0.38	0.083	0.064	0.047
rs11822254	G	A	3.52E-06	-0.08	0.017	0.004	0.012
rs11889341	T	C	6.64E-11	0.12	0.019	-0.012	0.014
rs1317983	C	T	1.65E-07	0.09	0.018	0.003	0.013
rs13249705	G	A	3.78E-07	-0.09	0.018	-0.003	0.014
rs140111921	A	G	1.34E-06	-0.16	0.034	0.008	0.031
rs150619443	C	G	5.08E-07	0.44	0.088	-0.034	0.054
rs1534430	T	C	9.40E-08	-0.09	0.017	-0.020	0.012
rs17490285	G	A	9.26E-07	-0.18	0.038	-0.014	0.019
rs1887414	C	T	1.97E-07	0.12	0.022	-0.018	0.016
rs2111485	G	A	4.19E-06	0.08	0.017	0.006	0.012
rs229526	C	G	1.93E-06	0.10	0.022	-0.011	0.015
rs2476601	G	A	3.49E-45	-0.33	0.023	0.019	0.019
rs28367132	A	G	1.21E-07	-0.14	0.026	0.002	0.015
rs28450758	A	C	4.17E-06	0.14	0.031	-0.015	0.022
rs3184504	C	T	1.40E-25	-0.18	0.017	-0.024	0.013
rs34536443	C	G	2.47E-07	-0.25	0.049	-0.016	0.030

Supplementary Table 2. Details of Hashimoto's Thyroiditis predicting SNPs with inflammatory proteins from IEU Open GWAS (with SNPs reaching $P < 5 \times 10^{-6}$).

Inflammatory proteins				Hashimoto's Thyroiditis			
SNP	Effect allele	Other allele	P value	Beta	Standard Error	log(OR)	Standard Error
rs34636506	G	A	1.92E-14	-0.13	0.018	-0.003	0.012
rs61830417	G	A	3.59E-06	0.27	0.059	-0.015	0.041
rs62076103	G	A	1.23E-07	-0.19	0.035	-0.012	0.024
rs6448432	A	G	1.75E-06	0.09	0.019	0.014	0.013
rs6501550	T	C	1.42E-06	-0.18	0.037	-0.028	0.035
rs7030280	T	C	6.44E-31	0.21	0.018	-0.012	0.013
rs72808590	C	T	1.55E-06	-0.37	0.077	0.023	0.044
rs73165154	G	A	3.76E-06	-0.15	0.033	0.025	0.022
rs73192661	T	C	1.45E-09	-0.10	0.017	0.008	0.012
rs7335748	G	A	2.41E-06	0.38	0.080	-0.013	0.041
rs766932	T	A	4.96E-07	0.10	0.019	-0.008	0.013
rs7767668	T	C	9.60E-07	-0.09	0.019	-0.002	0.012
rs7861040	C	G	9.08E-07	0.08	0.017	-0.021	0.012
rs79219789	C	G	2.84E-06	-0.15	0.033	0.023	0.031
rs8084503	T	C	1.97E-07	0.14	0.027	0.023	0.018
rs847803	G	A	7.91E-07	0.09	0.018	0.003	0.013
rs9271365	G	T	1.44E-39	0.25	0.019	-0.005	0.012
rs9357119	T	G	3.65E-14	-0.15	0.020	-0.009	0.013
rs9497965	T	C	1.71E-06	0.08	0.017	-0.004	0.012
**TRANCE (F-statistic = 36.52, R2=0.42%)**							
rs10424978	A	C	8.97E-07	-0.08	0.017	-0.013	0.012
rs10494077	A	G	9.20E-10	0.12	0.020	-0.006	0.014
rs10786679	A	G	1.87E-06	-0.09	0.018	-0.013	0.013
rs111358861	G	A	3.30E-06	1.82	0.391	0.030	0.106
rs1115587	A	G	1.82E-07	-0.10	0.018	-0.001	0.012
rs11211645	G	A	9.33E-08	0.09	0.017	0.011	0.012
rs113075322	G	C	7.18E-07	-0.14	0.028	-0.057	0.033
rs11611029	T	C	1.16E-08	-0.10	0.017	0.000	0.012
rs117167029	C	G	8.05E-07	2.99	0.606	0.004	0.090
rs117369326	A	G	3.55E-06	0.24	0.051	-0.012	0.029
rs118075239	C	T	3.45E-06	0.38	0.083	0.025	0.047

Supplementary Table 2. Details of Hashimoto's Thyroiditis predicting SNPs with inflammatory proteins from IEU Open GWAS (with SNPs reaching $P < 5 \times 10^{-6}$).

Inflammatory proteins				Hashimoto's Thyroiditis			
SNP	Effect allele	Other allele	P value	Beta	Standard Error	log(OR)	Standard Error
rs11822254	G	A	3.52E-06	-0.08	0.017	0.021	0.012
rs11889341	T	C	6.64E-11	0.12	0.019	-0.006	0.014
rs1317983	C	T	1.65E-07	0.09	0.018	-0.010	0.013
rs13249705	G	A	3.78E-07	-0.09	0.018	-0.008	0.014
rs140111921	A	G	1.34E-06	-0.16	0.034	0.016	0.030
rs150619443	C	G	5.08E-07	0.44	0.088	-0.090	0.054
rs1534430	T	C	9.40E-08	-0.09	0.017	-0.015	0.012
rs17490285	G	A	9.26E-07	-0.18	0.038	-0.011	0.019
rs1887414	C	T	1.97E-07	0.12	0.022	-0.009	0.017
rs2111485	G	A	4.19E-06	0.08	0.017	0.003	0.012
rs229526	C	G	1.93E-06	0.10	0.022	-0.022	0.015
rs2476601	G	A	3.49E-45	-0.33	0.023	-0.008	0.019
rs28367132	A	G	1.21E-07	-0.14	0.026	0.011	0.015
rs28450758	A	C	4.17E-06	0.14	0.031	0.023	0.023
rs34536443	C	G	2.47E-07	-0.25	0.049	-0.034	0.030
rs34636506	G	A	1.92E-14	-0.13	0.018	-0.032	0.012
rs61830417	G	A	3.59E-06	0.27	0.059	0.094	0.041
rs62076103	G	A	1.23E-07	-0.19	0.035	0.014	0.025
rs6448432	A	G	1.75E-06	0.09	0.019	0.032	0.013
rs6501550	T	C	1.42E-06	-0.18	0.037	-0.046	0.035
rs7030280	T	C	6.44E-31	0.21	0.018	-0.014	0.013
rs72808590	C	T	1.55E-06	-0.37	0.077	0.074	0.045
rs73165154	G	A	3.76E-06	-0.15	0.033	0.012	0.022
rs73192661	T	C	1.45E-09	-0.10	0.017	0.003	0.012
rs7335748	G	A	2.41E-06	0.38	0.080	-0.034	0.041
rs766932	T	A	4.96E-07	0.10	0.019	0.030	0.013
rs7767668	T	C	9.60E-07	-0.09	0.019	0.002	0.012
rs7861040	C	G	9.08E-07	0.08	0.017	-0.007	0.012
rs79219789	C	G	2.84E-06	-0.15	0.033	0.033	0.031
rs8084503	T	C	1.97E-07	0.14	0.027	0.008	0.018
rs847803	G	A	7.91E-07	0.09	0.018	0.030	0.013

Supplementary Table 2. Details of Hashimoto's Thyroiditis predicting SNPs with inflammatory proteins from IEU Open GWAS (with SNPs reaching $P < 5 \times 10^{-6}$).

Inflammatory proteins				Hashimoto's Thyroiditis			
SNP	Effect allele	Other allele	P value	Beta	Standard Error	log(OR)	Standard Error
rs9271365	G	T	1.44E-39	0.25	0.019	0.021	0.012
rs9357119	T	G	3.65E-14	-0.15	0.020	-0.016	0.013
rs9497965	T	C	1.71E-06	0.08	0.017	-0.012	0.012
**TSLP (F-statistic = 38.11, R2=0.44%)**							
rs10424978	A	C	8.97E-07	-0.08	0.017	-0.003	0.014
rs10494077	A	G	9.20E-10	0.12	0.020	0.016	0.016
rs10786679	A	G	1.87E-06	-0.09	0.018	-0.010	0.015
rs111358861	G	A	3.30E-06	1.82	0.391	0.140	0.107
rs1115587	A	G	1.82E-07	-0.10	0.018	0.025	0.014
rs11211645	G	A	9.33E-08	0.09	0.017	-0.008	0.013
rs113075322	G	C	7.18E-07	-0.14	0.028	-0.006	0.036
rs11611029	T	C	1.16E-08	-0.10	0.017	-0.004	0.014
rs117167029	C	G	8.05E-07	2.99	0.606	0.105	0.111
rs117369326	A	G	3.55E-06	0.24	0.051	-0.008	0.033
rs118075239	C	T	3.45E-06	0.38	0.083	-0.005	0.051
rs11822254	G	A	3.52E-06	-0.08	0.017	-0.001	0.014
rs11889341	T	C	6.64E-11	0.12	0.019	-0.020	0.016
rs1317983	C	T	1.65E-07	0.09	0.018	0.037	0.015
rs13249705	G	A	3.78E-07	-0.09	0.018	-0.015	0.016
rs140111921	A	G	1.34E-06	-0.16	0.034	0.009	0.033
rs150619443	C	G	5.08E-07	0.44	0.088	-0.059	0.059
rs1534430	T	C	9.40E-08	-0.09	0.017	0.010	0.014
rs17490285	G	A	9.26E-07	-0.18	0.038	0.015	0.021
rs1887414	C	T	1.97E-07	0.12	0.022	-0.010	0.019
rs2111485	G	A	4.19E-06	0.08	0.017	0.008	0.013
rs229526	C	G	1.93E-06	0.10	0.022	-0.008	0.017
rs2476601	G	A	3.49E-45	-0.33	0.023	0.008	0.021
rs28367132	A	G	1.21E-07	-0.14	0.026	-0.008	0.017
rs28450758	A	C	4.17E-06	0.14	0.031	-0.032	0.025
rs3184504	C	T	1.40E-25	-0.18	0.017	-0.004	0.013
rs34536443	C	G	2.47E-07	-0.25	0.049	-0.011	0.034

Supplementary Table 2. Details of Hashimoto's Thyroiditis predicting SNPs with inflammatory proteins from IEU Open GWAS (with SNPs reaching $P < 5 \times 10^{-6}$).

Inflammatory proteins				Hashimoto's Thyroiditis			
SNP	Effect allele	Other allele	P value	Beta	Standard Error	log(OR)	Standard Error
rs34636506	G	A	1.92E-14	-0.13	0.018	-0.011	0.014
rs61830417	G	A	3.59E-06	0.27	0.059	0.031	0.046
rs62076103	G	A	1.23E-07	-0.19	0.035	-0.015	0.027
rs6448432	A	G	1.75E-06	0.09	0.019	0.011	0.015
rs6501550	T	C	1.42E-06	-0.18	0.037	0.027	0.040
rs7030280	T	C	6.44E-31	0.21	0.018	0.005	0.014
rs72808590	C	T	1.55E-06	-0.37	0.077	0.087	0.049
rs73165154	G	A	3.76E-06	-0.15	0.033	0.016	0.024
rs73192661	T	C	1.45E-09	-0.10	0.017	0.000	0.014
rs7335748	G	A	2.41E-06	0.38	0.080	0.032	0.047
rs766932	T	A	4.96E-07	0.10	0.019	-0.012	0.015
rs7767668	T	C	9.60E-07	-0.09	0.019	0.001	0.013
rs7861040	C	G	9.08E-07	0.08	0.017	0.004	0.014
rs79219789	C	G	2.84E-06	-0.15	0.033	0.000	0.036
rs8084503	T	C	1.97E-07	0.14	0.027	0.057	0.021
rs847803	G	A	7.91E-07	0.09	0.018	0.005	0.014
rs9271365	G	T	1.44E-39	0.25	0.019	-0.005	0.013
rs9357119	T	G	3.65E-14	-0.15	0.020	0.004	0.015
rs9497965	T	C	1.71E-06	0.08	0.017	0.007	0.014
**uPA (F-statistic = 36.52, R2=0.42%)**							
rs10424978	A	C	8.97E-07	-0.08	0.017	-0.019	0.012
rs10494077	A	G	9.20E-10	0.12	0.020	0.009	0.014
rs10786679	A	G	1.87E-06	-0.09	0.018	0.009	0.014
rs111358861	G	A	3.30E-06	1.82	0.391	-0.146	0.107
rs1115587	A	G	1.82E-07	-0.10	0.018	0.002	0.012
rs11211645	G	A	9.33E-08	0.09	0.017	-0.007	0.012
rs113075322	G	C	7.18E-07	-0.14	0.028	0.005	0.033
rs11611029	T	C	1.16E-08	-0.10	0.017	-0.014	0.012
rs117167029	C	G	8.05E-07	2.99	0.606	0.095	0.093
rs117369326	A	G	3.55E-06	0.24	0.051	0.018	0.029
rs118075239	C	T	3.45E-06	0.38	0.083	0.095	0.047

Supplementary Table 2. Details of Hashimoto's Thyroiditis predicting SNPs with inflammatory proteins from IEU Open GWAS (with SNPs reaching $P < 5 \times 10^{-6}$).

Inflammatory proteins				Hashimoto's Thyroiditis			
SNP	Effect allele	Other allele	P value	Beta	Standard Error	log(OR)	Standard Error
rs11822254	G	A	3.52E-06	-0.08	0.017	-0.014	0.012
rs11889341	T	C	6.64E-11	0.12	0.019	-0.010	0.014
rs1317983	C	T	1.65E-07	0.09	0.018	0.022	0.013
rs13249705	G	A	3.78E-07	-0.09	0.018	-0.018	0.014
rs140111921	A	G	1.34E-06	-0.16	0.034	-0.029	0.031
rs150619443	C	G	5.08E-07	0.44	0.088	-0.046	0.054
rs1534430	T	C	9.40E-08	-0.09	0.017	-0.008	0.012
rs17490285	G	A	9.26E-07	-0.18	0.038	-0.023	0.019
rs1887414	C	T	1.97E-07	0.12	0.022	0.005	0.016
rs2111485	G	A	4.19E-06	0.08	0.017	-0.005	0.012
rs229526	C	G	1.93E-06	0.10	0.022	0.028	0.015
rs2476601	G	A	3.49E-45	-0.33	0.023	0.048	0.019
rs28367132	A	G	1.21E-07	-0.14	0.026	-0.003	0.015
rs28450758	A	C	4.17E-06	0.14	0.031	-0.029	0.022
rs34536443	C	G	2.47E-07	-0.25	0.049	-0.006	0.030
rs34636506	G	A	1.92E-14	-0.13	0.018	0.004	0.012
rs61830417	G	A	3.59E-06	0.27	0.059	0.004	0.041
rs62076103	G	A	1.23E-07	-0.19	0.035	-0.017	0.024
rs6448432	A	G	1.75E-06	0.09	0.019	-0.004	0.013
rs6501550	T	C	1.42E-06	-0.18	0.037	-0.021	0.035
rs7030280	T	C	6.44E-31	0.21	0.018	-0.010	0.013
rs72808590	C	T	1.55E-06	-0.37	0.077	-0.001	0.044
rs73165154	G	A	3.76E-06	-0.15	0.033	0.016	0.022
rs73192661	T	C	1.45E-09	-0.10	0.017	0.005	0.012
rs7335748	G	A	2.41E-06	0.38	0.080	0.000	0.040
rs766932	T	A	4.96E-07	0.10	0.019	-0.007	0.013
rs7767668	T	C	9.60E-07	-0.09	0.019	0.002	0.012
rs7861040	C	G	9.08E-07	0.08	0.017	0.003	0.012
rs79219789	C	G	2.84E-06	-0.15	0.033	0.015	0.030
rs8084503	T	C	1.97E-07	0.14	0.027	0.040	0.018
rs847803	G	A	7.91E-07	0.09	0.018	0.005	0.013

Supplementary Table 2. Details of Hashimoto's Thyroiditis predicting SNPs with inflammatory proteins from IEU Open GWAS (with SNPs reaching $P < 5 \times 10^{-6}$).

Inflammatory proteins				Hashimoto's Thyroiditis			
SNP	Effect allele	Other allele	P value	Beta	Standard Error	log(OR)	Standard Error
rs9271365	G	T	1.44E-39	0.25	0.019	0.014	0.012
rs9357119	T	G	3.65E-14	-0.15	0.020	-0.028	0.013
rs9497965	T	C	1.71E-06	0.08	0.017	0.015	0.012
**VEGF-A (F-statistic = 38.11, R2=0.44%)**							
rs10424978	A	C	8.97E-07	-0.08	0.017	-0.027	0.012
rs10494077	A	G	9.20E-10	0.12	0.020	-0.017	0.014
rs10786679	A	G	1.87E-06	-0.09	0.018	0.010	0.013
rs111358861	G	A	3.30E-06	1.82	0.391	-0.001	0.107
rs1115587	A	G	1.82E-07	-0.10	0.018	0.003	0.012
rs11211645	G	A	9.33E-08	0.09	0.017	-0.010	0.012
rs113075322	G	C	7.18E-07	-0.14	0.028	-0.025	0.033
rs11611029	T	C	1.16E-08	-0.10	0.017	-0.007	0.012
rs117167029	C	G	8.05E-07	2.99	0.606	0.025	0.090
rs117369326	A	G	3.55E-06	0.24	0.051	-0.012	0.029
rs118075239	C	T	3.45E-06	0.38	0.083	0.002	0.047
rs11822254	G	A	3.52E-06	-0.08	0.017	-0.002	0.012
rs11889341	T	C	6.64E-11	0.12	0.019	0.008	0.014
rs1317983	C	T	1.65E-07	0.09	0.018	0.022	0.013
rs13249705	G	A	3.78E-07	-0.09	0.018	0.001	0.014
rs140111921	A	G	1.34E-06	-0.16	0.034	-0.007	0.030
rs150619443	C	G	5.08E-07	0.44	0.088	0.003	0.054
rs1534430	T	C	9.40E-08	-0.09	0.017	0.014	0.012
rs17490285	G	A	9.26E-07	-0.18	0.038	-0.018	0.019
rs1887414	C	T	1.97E-07	0.12	0.022	-0.012	0.017
rs2111485	G	A	4.19E-06	0.08	0.017	0.000	0.012
rs229526	C	G	1.93E-06	0.10	0.022	-0.001	0.015
rs2476601	G	A	3.49E-45	-0.33	0.023	0.022	0.019
rs28367132	A	G	1.21E-07	-0.14	0.026	-0.019	0.015
rs28450758	A	C	4.17E-06	0.14	0.031	-0.034	0.022
rs3184504	C	T	1.40E-25	-0.18	0.017	-0.025	0.013
rs34536443	C	G	2.47E-07	-0.25	0.049	0.026	0.030

Supplementary Table 2. Details of Hashimoto's Thyroiditis predicting SNPs with inflammatory proteins from IEU Open GWAS (with SNPs reaching $P < 5 \times 10^{-6}$).

Inflammatory proteins				Hashimoto's Thyroiditis			
SNP	Effect allele	Other allele	P value	Beta	Standard Error	log(OR)	Standard Error
rs34636506	G	A	1.92E-14	-0.13	0.018	-0.009	0.012
rs61830417	G	A	3.59E-06	0.27	0.059	0.047	0.041
rs62076103	G	A	1.23E-07	-0.19	0.035	-0.021	0.024
rs6448432	A	G	1.75E-06	0.09	0.019	0.015	0.013
rs6501550	T	C	1.42E-06	-0.18	0.037	0.044	0.034
rs7030280	T	C	6.44E-31	0.21	0.018	-0.002	0.013
rs72808590	C	T	1.55E-06	-0.37	0.077	-0.087	0.044
rs73165154	G	A	3.76E-06	-0.15	0.033	-0.033	0.022
rs73192661	T	C	1.45E-09	-0.10	0.017	-0.013	0.012
rs7335748	G	A	2.41E-06	0.38	0.080	-0.038	0.041
rs766932	T	A	4.96E-07	0.10	0.019	-0.016	0.013
rs7767668	T	C	9.60E-07	-0.09	0.019	-0.003	0.012
rs7861040	C	G	9.08E-07	0.08	0.017	-0.009	0.012
rs79219789	C	G	2.84E-06	-0.15	0.033	0.008	0.031
rs8084503	T	C	1.97E-07	0.14	0.027	0.019	0.018
rs847803	G	A	7.91E-07	0.09	0.018	0.003	0.013
rs9271365	G	T	1.44E-39	0.25	0.019	-0.004	0.012
rs9357119	T	G	3.65E-14	-0.15	0.020	-0.005	0.013
rs9497965	T	C	1.71E-06	0.08	0.017	-0.024	0.012
**β-NGF (F-statistic = 38.11, R2=0.44%)**							
rs10424978	A	C	8.97E-07	-0.08	0.017	-0.008	0.012
rs10494077	A	G	9.20E-10	0.12	0.020	-0.003	0.013
rs10786679	A	G	1.87E-06	-0.09	0.018	0.009	0.013
rs111358861	G	A	3.30E-06	1.82	0.391	0.010	0.106
rs1115587	A	G	1.82E-07	-0.10	0.018	-0.003	0.012
rs11211645	G	A	9.33E-08	0.09	0.017	-0.008	0.011
rs113075322	G	C	7.18E-07	-0.14	0.028	-0.031	0.033
rs11611029	T	C	1.16E-08	-0.10	0.017	-0.001	0.012
rs117167029	C	G	8.05E-07	2.99	0.606	-0.018	0.088
rs117369326	A	G	3.55E-06	0.24	0.051	-0.026	0.028
rs118075239	C	T	3.45E-06	0.38	0.083	0.061	0.046

Supplementary Table 2. Details of Hashimoto's Thyroiditis predicting SNPs with inflammatory proteins from IEU Open GWAS (with SNPs reaching $P < 5 \times 10^{-6}$).

Inflammatory proteins				Hashimoto's Thyroiditis			
SNP	Effect allele	Other allele	P value	Beta	Standard Error	log(OR)	Standard Error
rs11822254	G	A	3.52E-06	-0.08	0.017	0.002	0.012
rs11889341	T	C	6.64E-11	0.12	0.019	-0.011	0.013
rs1317983	C	T	1.65E-07	0.09	0.018	0.012	0.013
rs13249705	G	A	3.78E-07	-0.09	0.018	-0.008	0.014
rs140111921	A	G	1.34E-06	-0.16	0.034	-0.046	0.030
rs150619443	C	G	5.08E-07	0.44	0.088	0.070	0.052
rs1534430	T	C	9.40E-08	-0.09	0.017	-0.016	0.012
rs17490285	G	A	9.26E-07	-0.18	0.038	0.012	0.019
rs1887414	C	T	1.97E-07	0.12	0.022	0.007	0.016
rs2111485	G	A	4.19E-06	0.08	0.017	-0.004	0.011
rs229526	C	G	1.93E-06	0.10	0.022	-0.015	0.014
rs2476601	G	A	3.49E-45	-0.33	0.023	0.004	0.018
rs28367132	A	G	1.21E-07	-0.14	0.026	-0.014	0.015
rs28450758	A	C	4.17E-06	0.14	0.031	-0.027	0.021
rs3184504	C	T	1.40E-25	-0.18	0.017	-0.027	0.012
rs34536443	C	G	2.47E-07	-0.25	0.049	0.007	0.029
rs34636506	G	A	1.92E-14	-0.13	0.018	-0.004	0.012
rs61830417	G	A	3.59E-06	0.27	0.059	-0.011	0.040
rs62076103	G	A	1.23E-07	-0.19	0.035	-0.012	0.023
rs6448432	A	G	1.75E-06	0.09	0.019	-0.002	0.012
rs6501550	T	C	1.42E-06	-0.18	0.037	0.013	0.034
rs7030280	T	C	6.44E-31	0.21	0.018	-0.015	0.013
rs72808590	C	T	1.55E-06	-0.37	0.077	0.009	0.043
rs73165154	G	A	3.76E-06	-0.15	0.033	-0.002	0.022
rs73192661	T	C	1.45E-09	-0.10	0.017	0.003	0.012
rs7335748	G	A	2.41E-06	0.38	0.080	0.029	0.039
rs766932	T	A	4.96E-07	0.10	0.019	0.006	0.012
rs7767668	T	C	9.60E-07	-0.09	0.019	0.010	0.011
rs7861040	C	G	9.08E-07	0.08	0.017	0.010	0.012
rs79219789	C	G	2.84E-06	-0.15	0.033	-0.035	0.029
rs8084503	T	C	1.97E-07	0.14	0.027	0.022	0.017

Supplementary Table 2. Details of Hashimoto's Thyroiditis predicting SNPs with inflammatory proteins from IEU Open GWAS (with SNPs reaching $P < 5 \times 10^{-6}$).

Inflammatory proteins				Hashimoto's Thyroiditis			
SNP	Effect allele	Other allele	P value	Beta	Standard Error	log(OR)	Standard Error
rs847803	G	A	7.91E-07	0.09	0.018	-0.011	0.013
rs9271365	G	T	1.44E-39	0.25	0.019	-0.018	0.011
rs9357119	T	G	3.65E-14	-0.15	0.020	0.022	0.012
rs9497965	T	C	1.71E-06	0.08	0.017	-0.010	0.011
**TNF-β (F-statistic = 36.04, R2=0.40%)**							
rs10424978	A	C	8.97E-07	-0.08	0.017	0.009	0.014
rs10494077	A	G	9.20E-10	0.12	0.020	0.006	0.016
rs10786679	A	G	1.87E-06	-0.09	0.018	-0.013	0.015
rs111358861	G	A	3.30E-06	1.82	0.391	0.074	0.106
rs1115587	A	G	1.82E-07	-0.10	0.018	-0.002	0.014
rs11211645	G	A	9.33E-08	0.09	0.017	-0.002	0.013
rs113075322	G	C	7.18E-07	-0.14	0.028	-0.014	0.036
rs11611029	T	C	1.16E-08	-0.10	0.017	0.003	0.014
rs117167029	C	G	8.05E-07	2.99	0.606	-0.082	0.111
rs117369326	A	G	3.55E-06	0.24	0.051	0.019	0.033
rs118075239	C	T	3.45E-06	0.38	0.083	0.045	0.051
rs11822254	G	A	3.52E-06	-0.08	0.017	-0.008	0.014
rs11889341	T	C	6.64E-11	0.12	0.019	0.013	0.016
rs1317983	C	T	1.65E-07	0.09	0.018	-0.012	0.015
rs13249705	G	A	3.78E-07	-0.09	0.018	0.004	0.016
rs140111921	A	G	1.34E-06	-0.16	0.034	0.018	0.033
rs150619443	C	G	5.08E-07	0.44	0.088	-0.097	0.058
rs1534430	T	C	9.40E-08	-0.09	0.017	-0.011	0.014
rs17490285	G	A	9.26E-07	-0.18	0.038	0.010	0.021
rs1887414	C	T	1.97E-07	0.12	0.022	-0.008	0.019
rs2111485	G	A	4.19E-06	0.08	0.017	-0.003	0.013
rs229526	C	G	1.93E-06	0.10	0.022	-0.026	0.017
rs2476601	G	A	3.49E-45	-0.33	0.023	-0.006	0.021
rs28367132	A	G	1.21E-07	-0.14	0.026	-0.001	0.017
rs28450758	A	C	4.17E-06	0.14	0.031	0.008	0.025
rs34536443	C	G	2.47E-07	-0.25	0.049	-0.059	0.034

Supplementary Table 2. Details of Hashimoto's Thyroiditis predicting SNPs with inflammatory proteins from IEU Open GWAS (with SNPs reaching *P* < 5 x 10^{-6}).

Inflammatory proteins				Hashimoto's Thyroiditis			
SNP	Effect allele	Other allele	P value	Beta	Standard Error	log(OR)	Standard Error
rs34636506	G	A	1.92E-14	-0.13	0.018	-0.048	0.014
rs61830417	G	A	3.59E-06	0.27	0.059	0.040	0.046
rs62076103	G	A	1.23E-07	-0.19	0.035	-0.022	0.027
rs6448432	A	G	1.75E-06	0.09	0.019	-0.015	0.014
rs6501550	T	C	1.42E-06	-0.18	0.037	-0.059	0.040
rs7030280	T	C	6.44E-31	0.21	0.018	-0.027	0.014
rs72808590	C	T	1.55E-06	-0.37	0.077	-0.062	0.048
rs73165154	G	A	3.76E-06	-0.15	0.033	0.046	0.024
rs73192661	T	C	1.45E-09	-0.10	0.017	0.013	0.014
rs7335748	G	A	2.41E-06	0.38	0.080	-0.010	0.047
rs766932	T	A	4.96E-07	0.10	0.019	0.011	0.015
rs7767668	T	C	9.60E-07	-0.09	0.019	-0.034	0.013
rs7861040	C	G	9.08E-07	0.08	0.017	-0.004	0.014
rs79219789	C	G	2.84E-06	-0.15	0.033	0.047	0.036
rs8084503	T	C	1.97E-07	0.14	0.027	0.001	0.021
rs847803	G	A	7.91E-07	0.09	0.018	0.017	0.014
rs9271365	G	T	1.44E-39	0.25	0.019	-0.030	0.013
rs9497965	T	C	1.71E-06	0.08	0.017	-0.011	0.014

Supplementary Table 3. Associations of inflammatory proteins with Hashimoto's Thyroiditis from GWAS using Mendelian randomization (with SNPs reaching *P* < 5 x 10^{-6}).

Category	Exposures	n SNPs	IVW						MR-Egger					Weighted Median			Weighted Mode			Simple Mode			MR-PRESSO
			Beta	OR(95% CI)	P	Q	P_Q	I^2	Beta	95% CI	P	intercept	$P_{intercept}$	Beta	95% CI	P	Beta	95% CI	P	Beta	95% CI	P	$P_{Global\ Test}$
**Chemokines**																							
	CCL19	19	-0.015	0.98 (0.88-1.10)	0.78	27.75	0.07	0.35	0.019	1.02 (0.79-1.32)	0.89	-0.004	0.76	-0.005	1.00 (0.85-1.16)	0.95	-0.039	0.96 (0.82-1.13)	0.64	-0.085	0.92 (0.71-1.19)	0.53	0.04
	CCL20	20	-0.156	0.86 (0.76-0.97)	0.01	14.43	0.76	0.00	-0.184	0.83 (0.61-1.13)	0.26	0.003	0.85	-0.193	0.82 (0.70-0.98)	0.03	-0.252	0.78 (0.58-1.05)	0.11	-0.260	0.77 (0.57-1.05)	0.12	0.77
	CCL23	26	-0.035	0.97 (0.90-1.04)	0.34	30.60	0.20	0.18	-0.024	0.98 (0.87-1.09)	0.68	-0.002	0.78	-0.051	0.95 (0.87-1.04)	0.27	-0.061	0.94 (0.86-1.03)	0.18	-0.115	0.89 (0.70-1.13)	0.35	0.29
	CCL25	24	0.018	1.02 (0.96-1.08)	0.56	28.29	0.21	0.19	-0.039	0.96 (0.87-1.07)	0.47	0.011	0.18	0.020	1.02 (0.94-1.11)	0.62	0.017	1.02 (0.95-1.09)	0.64	-0.094	0.91 (0.76-1.10)	0.33	0.27
	CCL28	22	0.079	1.08 (0.95-1.23)	0.23	8.04	0.99	0.00	-0.006	0.99 (0.76-1.31)	0.97	0.008	0.50	0.083	1.09 (0.92-1.28)	0.32	0.101	1.11 (0.85-1.45)	0.47	0.131	1.14 (0.87-1.50)	0.36	1.00
	CCL4	19	0.029	1.03 (0.97-1.09)	0.34	10.13	0.93	0.00	0.046	1.05 (0.96-1.14)	0.31	-0.005	0.60	0.038	1.04 (0.97-1.11)	0.28	0.037	1.04 (0.97-1.11)	0.28	0.043	1.04 (0.88-1.24)	0.64	0.95
	CS3CL1	22	-0.051	0.95 (0.81-1.11)	0.51	32.73	0.05	0.36	-0.367	0.69 (0.48-1.01)	0.07	0.030	0.09	-0.028	0.97 (0.81-1.16)	0.76	-0.004	1.00 (0.75-1.32)	0.98	-0.108	0.90 (0.65-1.24)	0.52	0.05
	CXCL1	12	0.015	1.02 (0.91-1.13)	0.78	18.03	0.08	0.39	0.078	1.08 (0.88-1.33)	0.48	-0.012	0.46	0.040	1.04 (0.93-1.17)	0.49	0.037	1.04 (0.93-1.16)	0.54	-0.003	1.00 (0.76-1.31)	0.99	0.25
	CXCL10	19	0.127	1.14 (1.01-1.28)	0.04	20.04	0.33	0.10	0.137	1.15 (0.89-1.48)	0.30	-0.001	0.93	0.155	1.17 (0.98-1.39)	0.09	0.188	1.21 (0.98-1.48)	0.09	0.193	1.21 (0.94-1.57)	0.16	0.00
	CXCL11	23	-0.050	0.95 (0.85-1.06)	0.36	24.22	0.34	0.09	-0.135	0.87 (0.66-1.16)	0.36	0.009	0.53	-0.076	0.93 (0.79-1.08)	0.34	-0.075	0.93 (0.79-1.09)	0.36	-0.114	0.89 (0.70-1.14)	0.36	0.38
	CXCL5	15	0.072	1.07 (0.99-1.17)	0.08	11.12	0.68	0.00	0.102	1.11 (0.98-1.25)	0.12	-0.006	0.52	0.083	1.09 (0.99-1.20)	0.10	0.082	1.09 (0.99-1.19)	0.10	0.101	1.11 (0.92-1.32)	0.29	0.76
	CXCL6	12	0.015	1.01 (0.94-1.10)	0.71	15.70	0.15	0.30	0.055	1.06 (0.90-1.24)	0.51	-0.011	0.55	0.017	1.02 (0.94-1.10)	0.69	0.007	1.01 (0.93-1.09)	0.86	-0.060	0.94 (0.76-1.17)	0.59	0.39
	CXCL9	21	0.138	1.15 (0.99-1.34)	0.08	31.62	0.05	0.37	0.151	1.16 (0.85-1.59)	0.36	-0.002	0.92	0.121	1.13 (0.96-1.33)	0.15	0.117	1.12 (0.91-1.38)	0.29	0.106	1.11 (0.88-1.41)	0.39	0.07
	Eotaxin	20	-0.052	0.95 (0.86-1.05)	0.32	21.78	0.30	0.13	0.155	1.17 (0.95-1.44)	0.16	-0.027	0.04	0.008	1.01 (0.87-1.17)	0.91	-0.004	1.00 (0.84-1.18)	0.97	-0.231	0.79 (0.60-1.05)	0.12	0.31
	MCP-1	23	-0.057	0.94 (0.88-1.02)	0.14	20.50	0.55	0.00	-0.030	0.97 (0.85-1.10)	0.65	-0.005	0.62	-0.101	0.90 (0.80-1.02)	0.09	-0.082	0.92 (0.80-1.06)	0.26	-0.120	0.89 (0.74-1.06)	0.20	0.55
	MCP-2	30	0.030	1.03 (0.97-1.09)	0.32	49.86	0.01	0.42	0.015	1.02 (0.94-1.10)	0.70	0.006	0.53	0.025	1.03 (0.96-1.10)	0.49	0.034	1.03 (0.98-1.09)	0.25	0.029	1.03 (0.91-1.17)	0.65	0.01
	MCP-3	19	0.039	1.04 (0.94-1.15)	0.44	15.59	0.62	0.00	-0.096	0.91 (0.73-1.13)	0.40	0.018	0.19	0.044	1.04 (0.90-1.22)	0.57	0.048	1.05 (0.89-1.24)	0.59	-0.078	0.92 (0.73-1.18)	0.53	0.64
	MCP-4	19	-0.008	0.99 (0.90-1.10)	0.87	30.05	0.04	0.40	-0.086	0.92 (0.75-1.12)	0.42	0.012	0.40	-0.060	0.94 (0.85-1.05)	0.28	-0.053	0.95 (0.86-1.05)	0.32	-0.041	0.96 (0.83-1.11)	0.59	0.06
	MIP-1α	14	0.085	1.09 (1.00-1.18)	0.05	15.20	0.30	0.14	0.007	1.01 (0.87-1.16)	0.93	0.018	0.19	0.060	1.06 (0.96-1.17)	0.22	0.057	1.06 (0.96-1.16)	0.25	0.156	1.17 (0.91-1.50)	0.25	0.36

Supplementary Table 3. Associations of inflammatory proteins with Hashimoto's Thyroiditis from GWAS using Mendelian randomization (with SNPs reaching *P* < 5 x 10^{-6}).

Category	Exposures	n SNPs	IVW						MR-Egger					Weighted Median			Weighted Mode			Simple Mode			MR-PRESSO
			Beta	OR(95% CI)	P	Q	P_Q	I^2	Beta	95% CI	P	intercept	$P_{intercept}$	Beta	95% CI	P	Beta	95% CI	P	Beta	95% CI	P	$P_{Global\ Test}$
**Growth factors**																							
	ARTN	20	0.027	1.03 (0.91-1.16)	0.67	27.61	0.09	0.31	-0.127	0.88 (0.60-1.29)	0.52	0.015	0.40	0.024	1.02 (0.86-1.22)	0.79	0.024	1.02 (0.80-1.31)	0.85	0.017	1.02 (0.77-1.34)	0.90	0.09
	FGF19	21	-0.105	0.90 (0.81-1.00)	0.04	24.69	0.21	0.19	-0.386	0.68 (0.53-0.87)	0.01	0.033	0.03	-0.156	0.86 (0.74-0.99)	0.03	-0.157	0.85 (0.74-0.99)	0.04	-0.145	0.87 (0.71-1.05)	0.16	0.03
	FGF21	14	0.018	1.02 (0.91-1.13)	0.75	9.28	0.75	0.00	0.114	1.12 (0.87-1.45)	0.40	-0.012	0.44	0.080	1.08 (0.93-1.26)	0.30	0.054	1.06 (0.90-1.24)	0.53	-0.062	0.94 (0.72-1.22)	0.65	0.72
	FGF23	17	-0.140	0.87 (0.76-1.00)	0.04	21.92	0.15	0.27	-0.505	0.60 (0.38-0.96)	0.05	0.035	0.13	-0.129	0.88 (0.71-1.08)	0.22	-0.307	0.74 (0.51-1.06)	0.12	0.047	1.05 (0.73-1.51)	0.80	0.12
	FGF5	25	0.076	1.08 (1.02-1.14)	0.01	19.90	0.70	0.00	0.120	1.13 (1.03-1.23)	0.01	-0.011	0.19	0.099	1.10 (1.03-1.18)	0.01	0.100	1.11 (1.02-1.19)	0.02	0.092	1.10 (0.92-1.30)	0.31	0.69
	GDNF	16	-0.038	0.96 (0.87-1.07)	0.48	17.90	0.27	0.16	-0.182	0.83 (0.69-1.00)	0.07	0.025	0.08	-0.076	0.93 (0.80-1.07)	0.30	-0.098	0.91 (0.79-1.04)	0.18	0.080	1.08 (0.85-1.38)	0.53	0.32
	HGF	19	-0.024	0.98 (0.85-1.12)	0.73	14.77	0.68	0.00	-0.027	0.97 (0.72-1.32)	0.86	0.000	0.98	-0.051	0.95 (0.79-1.14)	0.59	-0.050	0.95 (0.77-1.18)	0.66	-0.055	0.95 (0.71-1.27)	0.72	0.69
	M-CSF	17	0.048	1.05 (0.93-1.18)	0.44	22.26	0.14	0.28	0.423	1.53 (1.09-2.14)	0.03	-0.045	0.03	0.086	1.09 (0.91-1.30)	0.34	0.145	1.16 (0.97-1.38)	0.13	-0.147	0.86 (0.60-1.24)	0.44	0.13
	NT-3	18	-0.036	0.96 (0.85-1.10)	0.59	17.99	0.39	0.05	-0.320	0.73 (0.54-0.97)	0.05	0.029	0.05	-0.093	0.91 (0.76-1.09)	0.32	-0.111	0.90 (0.66-1.21)	0.49	-0.105	0.90 (0.65-1.24)	0.53	0.38
	NTN	14	-0.050	0.95 (0.84-1.07)	0.41	12.20	0.51	0.00	-0.156	0.86 (0.69-1.06)	0.17	0.016	0.25	-0.142	0.87 (0.73-1.03)	0.10	-0.169	0.84 (0.68-1.04)	0.14	-0.159	0.85 (0.66-1.10)	0.24	0.49
	SCF	32	-0.020	0.98 (0.92-1.05)	0.57	19.24	0.95	0.00	-0.010	0.99 (0.88-1.12)	0.87	-0.001	0.85	-0.053	0.95 (0.86-1.05)	0.30	-0.065	0.94 (0.84-1.05)	0.26	-0.079	0.92 (0.79-1.08)	0.34	0.95
	TGF-α	13	-0.045	0.96 (0.83-1.10)	0.53	15.71	0.20	0.24	-0.152	0.86 (0.60-1.24)	0.43	0.014	0.53	-0.097	0.91 (0.74-1.12)	0.36	-0.086	0.92 (0.71-1.18)	0.52	-0.013	0.99 (0.74-1.31)	0.93	0.21
	VEFG-A	24	0.025	1.03 (0.96-1.10)	0.48	32.03	0.10	0.28	-0.032	0.97 (0.84-1.12)	0.66	0.012	0.34	0.026	1.03 (0.94-1.12)	0.55	0.029	1.03 (0.94-1.13)	0.54	0.156	1.17 (0.93-1.47)	0.19	0.18
	β-NGF	22	0.043	1.04 (0.92-1.18)	0.49	30.04	0.09	0.30	0.033	1.03 (0.71-1.51)	0.87	0.001	0.96	-0.042	0.96 (0.79-1.16)	0.67	-0.132	0.88 (0.67-1.15)	0.35	-0.127	0.88 (0.63-1.23)	0.47	0.09
Interleukins																							
	IL-10	17	0.004	1.00 (0.90-1.12)	0.94	23.63	0.10	0.32	-0.023	0.98 (0.74-1.30)	0.88	0.003	0.84	0.022	1.02 (0.87-1.20)	0.79	0.079	1.08 (0.83-1.41)	0.57	0.122	1.13 (0.86-1.49)	0.40	0.10
	IL-10Rα	10	0.036	1.04 (0.92-1.17)	0.56	12.17	0.20	0.26	0.110	1.12 (0.89-1.40)	0.37	-0.013	0.43	0.012	1.01 (0.86-1.19)	0.89	-0.004	1.00 (0.84-1.18)	0.96	0.078	1.08 (0.85-1.38)	0.55	0.27
	IL-10Rβ	19	-0.029	0.97 (0.91-1.03)	0.36	26.36	0.09	0.32	0.012	1.01 (0.90-1.13)	0.83	-0.011	0.34	-0.036	0.96 (0.90-1.04)	0.33	-0.036	0.97 (0.90-1.03)	0.33	-0.152	0.86 (0.71-1.04)	0.14	0.21
	IL-12p40	25	0.251	1.29 (1.16-1.42)	0.00	34.20	0.08	0.30	-0.002	1.00 (0.75-1.32)	0.99	0.027	0.07	0.200	1.22 (1.04-1.43)	0.01	0.090	1.09 (0.84-1.42)	0.51	0.083	1.09 (0.78-1.51)	0.62	0.04
	IL-13	13	0.077	1.08 (0.96-1.22)	0.21	9.23	0.68	0.00	-0.012	0.99 (0.77-1.27)	0.93	0.011	0.44	0.076	1.08 (0.92-1.27)	0.36	0.058	1.06 (0.84-1.33)	0.63	0.027	1.03 (0.80-1.32)	0.84	0.71

Supplementary Table 3. Associations of inflammatory proteins with Hashimoto's Thyroiditis from GWAS using Mendelian randomization (with SNPs reaching $P < 5 \times 10^{-6}$).

Category	Exposures	n SNPs	IVW						MR-Egger					Weighted Median			Weighted Mode			Simple Mode			MR-PRESSO
			Beta	OR(95% CI)	P	Q	P_Q	I^2	Beta	95% CI	P	intercept	$P_{intercept}$	Beta	95% CI	P	Beta	95% CI	P	Beta	95% CI	P	$P_{Global\ Test}$
	IL-15Rα	12	-0.044	0.96 (0.86-1.06)	0.40	22.83	0.02	0.52	-0.064	0.94 (0.77-1.14)	0.52	0.005	0.81	-0.058	0.94 (0.87-1.03)	0.20	-0.064	0.94 (0.86-1.02)	0.17	-0.146	0.86 (0.71-1.05)	0.17	0.09
	IL-17A	13	-0.019	0.98 (0.84-1.14)	0.80	19.92	0.07	0.40	-0.256	0.77 (0.48-1.25)	0.32	0.025	0.31	-0.040	0.96 (0.76-1.21)	0.73	-0.045	0.96 (0.70-1.31)	0.78	-0.031	0.97 (0.70-1.35)	0.86	0.01
	IL-17C	20	0.012	1.01 (0.90-1.14)	0.85	14.92	0.73	0.00	-0.167	0.85 (0.59-1.22)	0.38	0.018	0.33	-0.034	0.97 (0.82-1.13)	0.68	-0.060	0.94 (0.74-1.19)	0.63	0.011	1.01 (0.78-1.31)	0.93	0.70
	IL-18	23	-0.032	0.97 (0.89-1.06)	0.47	26.22	0.24	0.16	0.023	1.02 (0.83-1.26)	0.83	-0.007	0.57	-0.049	0.95 (0.84-1.08)	0.44	0.012	1.01 (0.89-1.16)	0.86	0.025	1.03 (0.84-1.25)	0.81	0.27
	IL-18R1	23	0.041	1.04 (0.99-1.10)	0.12	13.63	0.91	0.00	0.062	1.06 (0.98-1.15)	0.14	-0.007	0.50	0.042	1.04 (0.98-1.11)	0.18	0.044	1.04 (0.99-1.11)	0.15	0.025	1.03 (0.88-1.19)	0.74	0.94
	IL-1α	14	0.037	1.04 (0.91-1.18)	0.56	9.75	0.71	0.00	0.091	1.09 (0.86-1.39)	0.47	-0.008	0.61	0.097	1.10 (0.93-1.31)	0.27	0.120	1.13 (0.93-1.37)	0.24	0.130	1.14 (0.90-1.45)	0.31	0.71
	IL-2	15	0.126	1.13 (1.00-1.29)	0.06	21.93	0.08	0.36	0.018	1.02 (0.66-1.56)	0.94	0.013	0.60	0.138	1.15 (0.94-1.40)	0.18	0.182	1.20 (0.88-1.63)	0.27	0.218	1.24 (0.88-1.77)	0.24	0.01
	IL-20	13	0.102	1.11 (0.95-1.29)	0.20	17.50	0.13	0.31	0.013	1.01 (0.67-1.53)	0.95	0.016	0.64	0.192	1.21 (0.97-1.51)	0.09	0.230	1.26 (0.95-1.67)	0.14	0.209	1.23 (0.86-1.76)	0.27	0.14
	IL-20Rα	11	0.067	1.07 (0.91-1.26)	0.42	14.32	0.16	0.30	-0.119	0.89 (0.58-1.37)	0.60	0.034	0.37	0.048	1.05 (0.84-1.30)	0.67	0.072	1.07 (0.87-1.33)	0.52	0.051	1.05 (0.80-1.39)	0.73	0.02
	IL-22Rα1	12	0.020	1.02 (0.87-1.20)	0.80	10.55	0.48	0.00	-0.184	0.83 (0.62-1.12)	0.26	0.032	0.14	0.053	1.05 (0.84-1.33)	0.65	0.095	1.10 (0.79-1.53)	0.58	0.136	1.15 (0.80-1.65)	0.48	0.49
	IL-24	12	-0.149	0.86 (0.71-1.04)	0.13	11.97	0.37	0.08	-0.454	0.64 (0.41-0.98)	0.07	0.038	0.16	-0.235	0.79 (0.62-1.01)	0.06	-0.233	0.79 (0.59-1.07)	0.16	-0.182	0.83 (0.61-1.14)	0.28	0.44
	IL-2Rβ	11	0.072	1.08 (0.90-1.28)	0.42	8.55	0.58	0.00	0.448	1.57 (1.07-2.28)	0.04	-0.042	0.05	0.213	1.24 (0.98-1.56)	0.07	0.249	1.28 (0.92-1.80)	0.18	0.262	1.30 (0.92-1.84)	0.17	0.56
	IL-33	12	0.053	1.05 (0.92-1.21)	0.46	14.26	0.22	0.23	0.425	1.53 (1.06-2.21)	0.05	-0.040	0.06	0.051	1.05 (0.86-1.28)	0.62	0.106	1.11 (0.84-1.47)	0.47	0.099	1.10 (0.80-1.52)	0.56	0.24
	IL-4	11	0.148	1.16 (0.99-1.36)	0.07	11.23	0.34	0.11	-0.192	0.83 (0.55-1.25)	0.38	0.036	0.11	0.198	1.22 (0.97-1.53)	0.08	0.198	1.22 (0.90-1.65)	0.23	0.177	1.19 (0.87-1.64)	0.30	0.00
	IL-5	11	-0.045	0.96 (0.83-1.10)	0.53	4.97	0.89	0.00	-0.034	0.97 (0.74-1.27)	0.81	-0.002	0.93	-0.051	0.95 (0.78-1.16)	0.62	-0.064	0.94 (0.72-1.22)	0.64	-0.010	0.99 (0.74-1.32)	0.95	0.90
	IL-6	8	-0.032	0.97 (0.84-1.12)	0.66	7.24	0.40	0.03	-0.061	0.94 (0.69-1.28)	0.71	0.004	0.84	-0.051	0.95 (0.80-1.13)	0.55	-0.052	0.95 (0.80-1.13)	0.58	-0.069	0.93 (0.71-1.23)	0.63	0.54
	IL-7	16	0.152	1.16 (0.99-1.37)	0.07	21.36	0.13	0.30	0.288	1.33 (0.71-2.52)	0.39	-0.011	0.66	0.168	1.18 (0.94-1.50)	0.16	0.121	1.13 (0.82-1.55)	0.46	0.146	1.16 (0.80-1.67)	0.45	0.15
	IL-8	17	-0.020	0.98 (0.86-1.12)	0.76	7.96	0.95	0.00	0.056	1.06 (0.82-1.37)	0.68	-0.008	0.51	0.021	1.02 (0.86-1.21)	0.81	0.049	1.05 (0.84-1.31)	0.67	0.026	1.03 (0.76-1.39)	0.87	0.95
	INF-γ	10	-0.165	0.85 (0.73-0.98)	0.03	11.51	0.24	0.22	0.128	1.14 (0.87-1.48)	0.37	-0.040	0.03	-0.091	0.91 (0.74-1.13)	0.41	0.031	1.03 (0.71-1.50)	0.87	0.038	1.04 (0.71-1.52)	0.85	0.28
	LAP TGF-β1	20	0.100	1.11 (0.99-1.23)	0.07	24.75	0.17	0.23	0.195	1.22 (0.98-1.50)	0.09	-0.013	0.30	0.125	1.13 (0.97-1.33)	0.12	0.158	1.17 (1.01-1.36)	0.06	0.215	1.24 (0.97-1.58)	0.10	0.22

Supplementary Table 3. Associations of inflammatory proteins with Hashimoto's Thyroiditis from GWAS using Mendelian randomization (with SNPs reaching *P* < 5 x 10^{-6}).

Category	Exposures	n SNPs	IVW						MR-Egger					Weighted Median			Weighted Mode			Simple Mode			MR-PRESSO
			Beta	OR(95% CI)	P	Q	P_Q	I^2	Beta	95% CI	P	intercept	$P_{intercept}$	Beta	95% CI	P	Beta	95% CI	P	Beta	95% CI	P	$P_{Global\ Test}$
	LIF	12	-0.009	0.99 (0.85-1.15)	0.91	9.21	0.60	0.00	0.336	1.40 (1.01-1.93)	0.07	-0.040	0.04	-0.027	0.97 (0.79-1.20)	0.80	0.157	1.17 (0.85-1.61)	0.35	-0.238	0.79 (0.56-1.12)	0.21	0.57
	LIFR	18	0.070	1.07 (0.98-1.17)	0.13	18.99	0.33	0.10	0.047	1.05 (0.87-1.27)	0.64	0.004	0.78	0.045	1.05 (0.93-1.18)	0.47	0.028	1.03 (0.92-1.16)	0.64	0.186	1.20 (1.00-1.45)	0.07	0.35
	TSLP	15	0.051	1.05 (0.92-1.21)	0.47	19.24	0.16	0.27	-0.072	0.93 (0.65-1.33)	0.70	0.015	0.46	-0.016	0.98 (0.81-1.20)	0.87	-0.010	0.99 (0.75-1.30)	0.94	0.040	1.04 (0.77-1.41)	0.80	0.18
**Tumor Necrosis Factor**																							
	TNF	18	0.151	1.16 (1.02-1.33)	0.03	15.77	0.54	0.00	-0.032	0.97 (0.68-1.38)	0.86	0.018	0.29	0.221	1.25 (1.03-1.51)	0.03	0.265	1.30 (0.99-1.72)	0.08	0.290	1.34 (1.00-1.79)	0.07	0.57
	TNFRSF9	26	0.093	1.10 (0.98-1.24)	0.12	41.32	0.02	0.40	0.038	1.04 (0.80-1.35)	0.78	0.007	0.65	0.080	1.08 (0.94-1.25)	0.27	0.014	1.01 (0.80-1.28)	0.91	-0.069	0.93 (0.72-1.20)	0.60	0.03
	TNFSF12	29	0.033	1.03 (0.93-1.15)	0.53	36.71	0.13	0.24	-0.063	0.94 (0.70-1.26)	0.68	0.010	0.49	0.010	1.01 (0.87-1.18)	0.90	-0.007	0.99 (0.80-1.23)	0.95	0.097	1.10 (0.85-1.43)	0.47	0.13
	TNFSF14	26	0.018	1.02 (0.94-1.11)	0.67	24.05	0.52	0.00	-0.081	0.92 (0.81-1.05)	0.24	0.016	0.07	-0.014	0.99 (0.88-1.11)	0.82	-0.016	0.98 (0.88-1.11)	0.79	0.036	1.04 (0.83-1.29)	0.75	0.53
	TNF-β	24	-0.019	0.98 (0.93-1.04)	0.53	15.32	0.88	0.00	0.046	1.05 (0.96-1.14)	0.32	-0.019	0.07	0.008	1.01 (0.94-1.08)	0.83	0.004	1.00 (0.94-1.07)	0.91	-0.129	0.88 (0.74-1.05)	0.16	0.73
	TRAIL	26	-0.042	0.96 (0.90-1.02)	0.20	29.29	0.25	0.15	-0.046	0.95 (0.85-1.07)	0.42	0.001	0.92	-0.034	0.97 (0.88-1.06)	0.46	-0.039	0.96 (0.88-1.05)	0.39	-0.063	0.94 (0.81-1.09)	0.42	0.30
	TRANCE	29	-0.070	0.93 (0.86-1.01)	0.07	37.51	0.11	0.25	-0.048	0.95 (0.79-1.14)	0.61	-0.003	0.79	-0.017	0.98 (0.87-1.11)	0.79	-0.079	0.92 (0.82-1.04)	0.21	-0.110	0.90 (0.73-1.10)	0.31	0.11
**Others**																							
	OPG	21	-0.119	0.89 (0.80-0.98)	0.02	14.50	0.80	0.00	-0.007	0.99 (0.80-1.23)	0.95	-0.015	0.27	-0.126	0.88 (0.76-1.02)	0.10	-0.145	0.86 (0.74-1.00)	0.07	-0.109	0.90 (0.72-1.12)	0.35	0.79
	EIF4E-BP1	11	-0.107	0.90 (0.78-1.03)	0.12	8.53	0.58	0.00	-0.225	0.80 (0.57-1.12)	0.23	0.012	0.48	-0.132	0.88 (0.73-1.05)	0.16	-0.131	0.88 (0.71-1.08)	0.24	-0.119	0.89 (0.66-1.19)	0.44	0.63
	FLT3L	27	-0.106	0.90 (0.81-0.99)	0.04	33.26	0.15	0.22	0.013	1.01 (0.73-1.40)	0.94	-0.011	0.45	-0.095	0.91 (0.78-1.05)	0.21	-0.115	0.89 (0.71-1.12)	0.33	-0.089	0.91 (0.69-1.22)	0.55	0.00
	CDCP1	24	0.078	1.08 (1.00-1.16)	0.04	26.72	0.27	0.14	0.017	1.02 (0.88-1.17)	0.81	0.010	0.31	0.018	1.02 (0.91-1.14)	0.76	0.019	1.02 (0.90-1.15)	0.77	0.137	1.15 (0.93-1.41)	0.20	0.24
	CD5	20	-0.153	0.86 (0.74-0.99)	0.04	32.35	0.03	0.41	-0.342	0.71 (0.48-1.05)	0.10	0.020	0.32	-0.113	0.89 (0.75-1.07)	0.22	-0.148	0.86 (0.63-1.19)	0.38	-0.164	0.85 (0.62-1.17)	0.33	0.03
	MMP-10	16	-0.095	0.91 (0.82-1.00)	0.06	18.05	0.26	0.17	0.041	1.04 (0.89-1.22)	0.61	-0.026	0.04	-0.012	0.99 (0.87-1.13)	0.85	-0.025	0.98 (0.86-1.11)	0.71	-0.137	0.87 (0.70-1.09)	0.25	0.29
	PD-L1	18	-0.093	0.91 (0.81-1.03)	0.14	23.67	0.13	0.28	0.031	1.03 (0.73-1.45)	0.86	-0.013	0.44	-0.169	0.84 (0.71-1.01)	0.06	-0.177	0.84 (0.69-1.02)	0.10	-0.188	0.83 (0.65-1.06)	0.15	0.17
	OSM	13	0.094	1.10 (0.97-1.24)	0.14	8.50	0.74	0.00	0.086	1.09 (0.83-1.43)	0.54	0.001	0.95	0.080	1.08 (0.92-1.28)	0.34	0.088	1.09 (0.90-1.33)	0.40	0.109	1.12 (0.87-1.43)	0.41	0.00
	CD40L	17	-0.054	0.95 (0.88-1.02)	0.14	20.42	0.20	0.22	-0.091	0.91 (0.81-1.03)	0.15	0.008	0.41	-0.090	0.91 (0.84-0.99)	0.03	-0.087	0.92 (0.84-0.99)	0.05	0.147	1.16 (0.90-1.49)	0.27	0.28

Supplementary Table 3. Associations of inflammatory proteins with Hashimoto's Thyroiditis from GWAS using Mendelian randomization (with SNPs reaching *P* < 5 x 10^{-6}).

Category	Exposures	n SNPs	IVW						MR-Egger					Weighted Median			Weighted Mode			Simple Mode			MR-PRESSO
			Beta	OR(95% CI)	P	Q	P_Q	I^2	Beta	95% CI	P	intercept	$P_{intercept}$	Beta	95% CI	P	Beta	95% CI	P	Beta	95% CI	P	$P_{Global\ Test}$
	SLAM	27	-0.070	0.93 (0.83-1.04)	0.22	29.23	0.30	0.11	0.051	1.05 (0.79-1.39)	0.72	-0.011	0.36	-0.088	0.92 (0.78-1.07)	0.28	-0.067	0.94 (0.76-1.15)	0.54	-0.040	0.96 (0.76-1.22)	0.74	0.33
	CD6	16	0.040	1.04 (0.97-1.11)	0.24	16.72	0.34	0.10	0.033	1.03 (0.93-1.14)	0.53	0.002	0.86	0.018	1.02 (0.94-1.10)	0.64	0.005	1.00 (0.92-1.10)	0.91	-0.116	0.89 (0.68-1.17)	0.42	0.38
	NKR2B4	20	0.046	1.05 (0.95-1.15)	0.35	17.43	0.56	0.00	-0.127	0.88 (0.72-1.07)	0.22	0.019	0.06	-0.049	0.95 (0.83-1.09)	0.47	-0.059	0.94 (0.82-1.09)	0.43	-0.051	0.95 (0.74-1.23)	0.70	0.48
	S100A12	17	0.051	1.05 (0.94-1.18)	0.38	11.63	0.77	0.00	-0.121	0.89 (0.70-1.12)	0.32	0.021	0.12	0.038	1.04 (0.89-1.22)	0.64	0.021	1.02 (0.85-1.23)	0.83	0.013	1.01 (0.79-1.29)	0.92	0.80
	uPA	23	-0.040	0.96 (0.87-1.06)	0.40	28.28	0.17	0.22	-0.096	0.91 (0.73-1.13)	0.40	0.007	0.57	-0.089	0.92 (0.79-1.06)	0.22	-0.095	0.91 (0.77-1.07)	0.26	-0.051	0.95 (0.76-1.19)	0.66	0.17
	AXIN1	8	0.068	1.07 (0.90-1.28)	0.45	1.79	0.97	0.00	0.151	1.16 (0.76-1.78)	0.51	-0.009	0.69	0.094	1.10 (0.87-1.38)	0.42	0.107	1.11 (0.82-1.51)	0.51	0.106	1.11 (0.81-1.53)	0.54	0.97
	SIRT2	13	-0.037	0.96 (0.84-1.10)	0.60	14.71	0.26	0.18	-0.136	0.87 (0.64-1.19)	0.41	0.013	0.49	-0.076	0.93 (0.77-1.12)	0.43	-0.091	0.91 (0.71-1.18)	0.50	-0.091	0.91 (0.65-1.28)	0.61	0.28
	STAMBP	12	-0.044	0.96 (0.80-1.15)	0.64	16.51	0.12	0.33	-0.468	0.63 (0.35-1.13)	0.15	0.053	0.16	-0.106	0.90 (0.68-1.19)	0.46	-0.294	0.75 (0.50-1.11)	0.17	0.255	1.29 (0.76-2.20)	0.37	0.11
	DNER	16	0.021	1.02 (0.92-1.14)	0.69	11.48	0.72	0.00	0.034	1.03 (0.81-1.32)	0.79	-0.001	0.91	0.056	1.06 (0.92-1.21)	0.42	0.059	1.06 (0.93-1.21)	0.40	0.040	1.04 (0.85-1.27)	0.70	0.78
	ADA	15	0.010	1.01 (0.94-1.08)	0.77	12.93	0.53	0.00	-0.016	0.98 (0.90-1.07)	0.72	0.009	0.33	0.006	1.01 (0.93-1.09)	0.89	0.007	1.01 (0.93-1.09)	0.86	0.047	1.05 (0.88-1.25)	0.61	0.64
	Capase	14	-0.023	0.98 (0.83-1.15)	0.79	22.64	0.05	0.43	0.044	1.05 (0.77-1.42)	0.78	-0.011	0.62	0.019	1.02 (0.85-1.22)	0.84	0.031	1.03 (0.84-1.27)	0.77	-0.109	0.90 (0.64-1.25)	0.53	0.06
	SULT1A1	23	0.004	1.00 (0.92-1.10)	0.93	18.29	0.69	0.00	0.000	1.00 (0.79-1.27)	1.00	0.000	0.97	-0.021	0.98 (0.86-1.12)	0.76	0.015	1.02 (0.86-1.19)	0.86	-0.083	0.92 (0.74-1.15)	0.48	0.70
	MMP-1	15	-0.004	1.00 (0.90-1.11)	0.95	14.35	0.42	0.02	-0.039	0.96 (0.79-1.18)	0.71	0.005	0.68	0.012	1.01 (0.86-1.18)	0.88	-0.004	1.00 (0.80-1.24)	0.98	0.038	1.04 (0.81-1.34)	0.77	0.04
	CST5	33	-0.044	0.96 (0.90-1.02)	0.16	28.53	0.64	0.00	-0.055	0.95 (0.86-1.04)	0.26	0.002	0.76	-0.069	0.93 (0.86-1.01)	0.10	-0.071	0.93 (0.86-1.01)	0.10	-0.028	0.97 (0.81-1.16)	0.76	0.67

Abbreviations: CI, Confidence interval; pval, p-value; Q, Cochran Q statistics; SNPs, single nucleotide polymorphisms

Beta and 95% CI represent change in SD of Inflammatory proteins per log odds increase in Hashimoto's Thyroiditis

Supplementary Table 4. Associations of Hashimoto's Thyroiditis with inflammatory proteins from IEU Open GWAS using Mendelian randomization (with SNPs reaching $P < 5\times10^{-6}$)

Category	Exposures	n SNPs	IVW						MR-Egger					Weighted Median			Weighted Mode			Simple Mode			MR-PRESSO
			Beta	OR(95% CI)	P	Q	P_Q	I^2	Beta	95% CI	P	intercept	$P_{intercept}$	Beta	95% CI	P	Beta	95% CI	P	Beta	95% CI	P	$P_{Global\ Test}$
**Chemokines**																							
	CCL19	43	0.042	1.04 (1.01-1.08)	0.01	44.51	0.37	0.06	0.040	1.04 (0.99-1.09)	0.11	0.001	0.87	0.043	1.04 (1.00-1.09)	0.08	0.038	1.04 (0.99-1.09)	0.14	0.082	1.09 (0.99-1.19)	0.08	0.33
	CCL20	46	-0.023	0.98 (0.95-1.00)	0.10	54.97	0.15	0.18	0.021	1.02 (0.97-1.07)	0.40	0.004	0.25	0.008	1.01 (0.95-1.06)	0.77	0.012	1.01 (0.96-1.07)	0.68	0.042	1.04 (0.93-1.17)	0.47	0.36
	CCL23	46	0.013	1.01 (0.99-1.04)	0.34	51.46	0.24	0.13	0.044	1.04 (1.00-1.09)	0.08	-0.002	0.67	0.037	1.04 (0.99-1.09)	0.11	0.036	1.04 (0.99-1.08)	0.12	0.050	1.05 (0.98-1.13)	0.20	0.37
	CCL25	46	0.043	1.04 (1.02-1.07)	0.00	49.37	0.30	0.09	-0.007	0.99 (0.94-1.04)	0.78	-0.003	0.42	-0.048	0.95 (0.91-1.00)	0.05	-0.052	0.95 (0.90-1.00)	0.06	-0.022	0.98 (0.89-1.07)	0.65	0.14
	CCL28	46	-0.020	0.98 (0.95-1.01)	0.14	55.10	0.14	0.18	-0.022	0.98 (0.93-1.03)	0.38	0.000	0.94	-0.034	0.97 (0.93-1.01)	0.11	-0.036	0.97 (0.92-1.01)	0.11	-0.036	0.97 (0.89-1.04)	0.36	0.14
	CCL4	46	0.036	1.04 (1.01-1.07)	0.01	48.64	0.33	0.07	-0.037	0.96 (0.92-1.01)	0.11	0.004	0.33	-0.030	0.97 (0.93-1.01)	0.16	-0.035	0.97 (0.92-1.01)	0.13	-0.012	0.99 (0.91-1.07)	0.77	0.69
	CX3CL1	45	0.008	1.01 (0.98-1.04)	0.56	27.08	0.98	-0.62	-0.002	1.00 (0.95-1.05)	0.95	0.004	0.37	-0.002	1.00 (0.95-1.05)	0.95	-0.002	1.00 (0.95-1.05)	0.94	0.009	1.01 (0.92-1.10)	0.84	0.06
	CXCL1	46	-0.012	0.99 (0.96-1.02)	0.41	51.95	0.22	0.13	-0.035	0.97 (0.92-1.01)	0.17	0.004	0.34	-0.033	0.97 (0.92-1.02)	0.18	-0.033	0.97 (0.92-1.02)	0.23	-0.016	0.98 (0.89-1.09)	0.76	0.15
	CXCL10	44	0.015	1.02 (0.98-1.05)	0.38	60.55	0.04	0.29	0.025	1.03 (0.98-1.07)	0.28	-0.002	0.61	0.019	1.02 (0.98-1.06)	0.38	0.012	1.01 (0.97-1.06)	0.60	-0.019	0.98 (0.90-1.06)	0.64	0.65
	CXCL11	45	-0.004	1.00 (0.97-1.02)	0.78	55.54	0.11	0.21	0.007	1.01 (0.96-1.06)	0.79	0.002	0.65	0.015	1.02 (0.97-1.07)	0.53	0.013	1.01 (0.97-1.06)	0.58	0.062	1.06 (0.98-1.16)	0.16	0.17
	CXCL5	46	-0.020	0.98 (0.95-1.01)	0.16	40.21	0.67	-0.12	0.003	1.00 (0.96-1.05)	0.91	0.002	0.58	0.004	1.00 (0.96-1.05)	0.88	0.008	1.01 (0.96-1.05)	0.73	0.022	1.02 (0.94-1.11)	0.63	0.25
	CXCL6	45	-0.010	0.99 (0.96-1.02)	0.48	29.39	0.96	-0.50	0.013	1.01 (0.96-1.07)	0.64	0.001	0.91	0.015	1.01 (0.97-1.07)	0.55	0.010	1.01 (0.97-1.06)	0.66	0.029	1.03 (0.94-1.13)	0.53	0.05
	CXCL9	43	0.017	1.02 (0.99-1.05)	0.25	57.32	0.06	0.27	-0.022	0.98 (0.93-1.03)	0.37	0.002	0.57	-0.036	0.96 (0.92-1.01)	0.12	-0.044	0.96 (0.91-1.01)	0.10	-0.053	0.95 (0.87-1.04)	0.26	0.20
	Eotaxin	46	0.009	1.01 (0.98-1.04)	0.53	51.06	0.25	0.12	-0.028	0.97 (0.93-1.02)	0.23	0.004	0.32	-0.029	0.97 (0.93-1.02)	0.21	-0.030	0.97 (0.93-1.02)	0.24	-0.019	0.98 (0.91-1.06)	0.64	0.95
	MCP-1	46	0.016	1.02 (0.99-1.04)	0.26	41.24	0.63	-0.09	0.018	1.02 (0.97-1.07)	0.46	-0.002	0.62	0.000	1.00 (0.95-1.05)	1.00	-0.006	0.99 (0.95-1.04)	0.80	-0.047	0.95 (0.87-1.05)	0.33	0.24
	MCP-2	46	-0.016	0.98 (0.96-1.01)	0.26	53.90	0.17	0.17	-0.001	1.00 (0.94-1.06)	0.98	0.002	0.66	-0.006	0.99 (0.95-1.04)	0.82	-0.002	1.00 (0.95-1.05)	0.93	-0.007	0.99 (0.92-1.08)	0.86	0.10
	MCP-3	46	0.010	1.01 (0.98-1.04)	0.54	58.44	0.09	0.23	-0.011	0.99 (0.95-1.03)	0.63	0.004	0.28	0.010	1.01 (0.97-1.05)	0.65	0.005	1.00 (0.96-1.05)	0.82	0.019	1.02 (0.95-1.09)	0.60	0.98
	MCP-4	46	0.004	1.00 (0.98-1.03)	0.76	56.80	0.11	0.21	0.002	1.00 (0.95-1.05)	0.94	0.001	0.90	-0.003	1.00 (0.95-1.05)	0.91	0.009	1.01 (0.96-1.06)	0.71	0.009	1.01 (0.93-1.09)	0.82	0.12
	MIP-1a	45	0.016	1.02 (0.99-1.05)	0.27	54.62	0.13	0.19	-0.008	0.99 (0.94-1.04)	0.76	0.001	0.84	-0.003	1.00 (0.95-1.04)	0.88	-0.002	1.00 (0.95-1.04)	0.93	0.007	1.01 (0.93-1.09)	0.87	0.15
**Growth factors**																							
	ARTN	46	0.015	1.02 (0.98-1.05)	0.34	45.74	0.44	0.02	0.021	1.02 (0.97-1.08)	0.44	0.000	0.95	0.018	1.02 (0.97-1.07)	0.51	0.027	1.03 (0.96-1.09)	0.41	0.036	1.04 (0.93-1.16)	0.52	0.05
	FGF19	46	-0.003	1.00 (0.97-1.02)	0.81	37.52	0.78	-0.20	-0.017	0.98 (0.94-1.03)	0.49	0.000	0.99	-0.031	0.97 (0.93-1.02)	0.19	-0.039	0.96 (0.92-1.01)	0.09	-0.024	0.98 (0.90-1.06)	0.57	0.19
	FGF21	46	-0.018	0.98 (0.96-1.01)	0.21	52.79	0.20	0.15	-0.003	1.00 (0.95-1.05)	0.92	-0.003	0.43	-0.011	0.99 (0.94-1.04)	0.65	-0.018	0.98 (0.93-1.03)	0.48	-0.050	0.95 (0.88-1.03)	0.23	0.96
	FGF23	46	0.019	1.02 (0.99-1.05)	0.17	61.24	0.05	0.27	0.020	1.02 (0.97-1.07)	0.42	-0.001	0.79	0.022	1.02 (0.97-1.07)	0.39	0.022	1.02 (0.97-1.07)	0.38	0.050	1.05 (0.96-1.15)	0.31	0.28
	FGF5	46	-0.019	0.98 (0.95-1.01)	0.22	30.16	0.96	-0.49	-0.029	0.97 (0.93-1.01)	0.20	0.003	0.40	-0.022	0.98 (0.94-1.02)	0.31	-0.023	0.98 (0.93-1.02)	0.33	-0.041	0.96 (0.89-1.04)	0.30	0.81
	GDNF	46	0.010	1.01 (0.98-1.04)	0.47	42.17	0.59	-0.07	0.021	1.02 (0.97-1.08)	0.43	-0.001	0.78	0.036	1.04 (0.98-1.09)	0.17	0.033	1.03 (0.97-1.10)	0.32	0.036	1.04 (0.95-1.13)	0.44	0.44
	HGF	45	-0.008	0.99 (0.96-1.02)	0.57	50.42	0.23	0.13	0.006	1.01 (0.96-1.05)	0.78	0.001	0.73	0.003	1.00 (0.96-1.05)	0.90	0.014	1.01 (0.96-1.07)	0.61	0.019	1.02 (0.93-1.12)	0.70	0.41
	M-CSF1	46	0.013	1.01 (0.99-1.04)	0.36	46.12	0.43	0.02	0.023	1.02 (0.97-1.08)	0.41	-0.002	0.71	0.018	1.02 (0.97-1.07)	0.47	0.011	1.01 (0.96-1.06)	0.66	-0.014	0.99 (0.91-1.07)	0.75	0.42

Supplementary Table 4. Associations of Hashimoto's Thyroiditis with inflammatory proteins from IEU Open GWAS using Mendelian randomization (with SNPs reaching $P< 5×10^{-6}$)

Category	Exposures	n SNPs	IVW						MR-Egger					Weighted Median			Weighted Mode			Simple Mode			MR-PRESSO
			Beta	OR(95% CI)	P	Q	P_Q	I^2	Beta	95% CI	P	intercept	$P_{intercept}$	Beta	95% CI	P	Beta	95% CI	P	Beta	95% CI	P	$P_{Global Test}$
	NT-3	46	-0.015	0.99 (0.96-1.01)	0.30	37.13	0.79	-0.21	0.002	1.00 (0.96-1.05)	0.92	0.002	0.66	-0.012	0.99 (0.95-1.03)	0.60	-0.020	0.98 (0.94-1.03)	0.40	-0.025	0.98 (0.90-1.06)	0.55	0.59
	NTN	46	0.015	1.01 (0.98-1.05)	0.36	47.40	0.37	0.05	0.007	1.01 (0.96-1.05)	0.78	0.001	0.85	0.008	1.01 (0.96-1.05)	0.74	0.000	1.00 (0.96-1.05)	1.00	0.006	1.01 (0.93-1.09)	0.89	0.50
	SCF	45	0.001	1.00 (0.97-1.04)	0.95	65.72	0.02	0.33	-0.016	0.98 (0.94-1.03)	0.53	0.002	0.70	-0.016	0.98 (0.94-1.03)	0.49	-0.013	0.99 (0.94-1.04)	0.60	-0.039	0.96 (0.89-1.04)	0.35	0.26
	TGF-α	45	0.015	1.02 (0.99-1.04)	0.29	49.99	0.25	0.12	-0.004	1.00 (0.95-1.04)	0.86	0.000	0.99	-0.009	0.99 (0.95-1.04)	0.70	-0.019	0.98 (0.94-1.03)	0.42	-0.037	0.96 (0.89-1.04)	0.37	0.82
	VEGF-A	46	0.010	1.01 (0.98-1.04)	0.48	45.29	0.46	0.01	-0.019	0.98 (0.94-1.03)	0.42	0.003	0.40	-0.016	0.98 (0.94-1.03)	0.47	-0.019	0.98 (0.94-1.02)	0.39	0.000	1.00 (0.94-1.07)	1.00	0.79
	β-NGF	46	-0.004	1.00 (0.97-1.02)	0.76	36.43	0.82	-0.24	-0.005	0.99 (0.94-1.05)	0.85	0.001	0.78	-0.011	0.99 (0.94-1.03)	0.62	-0.011	0.99 (0.95-1.03)	0.63	0.014	1.01 (0.93-1.11)	0.76	0.00
**Interleukins**																							
	IL-10	46	0.030	1.03 (0.99-1.07)	0.11	74.57	0.00	0.40	-0.008	0.99 (0.94-1.05)	0.79	0.008	0.08	0.001	1.00 (0.95-1.05)	0.96	-0.003	1.00 (0.95-1.05)	0.90	0.027	1.03 (0.93-1.14)	0.60	0.15
	IL-10Rα	46	0.004	1.00 (0.97-1.04)	0.81	56.89	0.11	0.21	0.008	1.01 (0.95-1.07)	0.79	0.005	0.35	-0.001	1.00 (0.95-1.05)	0.98	-0.014	0.99 (0.94-1.04)	0.57	-0.027	0.97 (0.87-1.09)	0.64	0.00
	IL-10Rβ	46	0.002	1.00 (0.97-1.03)	0.90	53.16	0.19	0.15	0.046	1.05 (0.99-1.11)	0.13	-0.005	0.30	0.022	1.02 (0.97-1.08)	0.42	0.018	1.02 (0.96-1.08)	0.55	0.013	1.01 (0.92-1.11)	0.79	0.15
	IL-13	46	-0.012	0.99 (0.96-1.02)	0.46	51.54	0.23	0.13	0.034	1.03 (0.98-1.09)	0.25	-0.003	0.52	0.009	1.01 (0.96-1.06)	0.76	0.003	1.00 (0.94-1.07)	0.92	0.060	1.06 (0.96-1.17)	0.24	0.17
	IL-15Rα	45	0.019	1.02 (0.99-1.05)	0.24	52.54	0.18	0.16	-0.013	0.99 (0.93-1.04)	0.65	-0.001	0.80	-0.024	0.98 (0.93-1.03)	0.36	-0.031	0.97 (0.92-1.03)	0.29	-0.044	0.96 (0.88-1.05)	0.33	0.03
	IL-17A	45	0.001	1.00 (0.96-1.04)	0.97	75.63	0.00	0.42	0.042	1.04 (0.99-1.10)	0.12	-0.006	0.19	0.026	1.03 (0.97-1.08)	0.35	0.040	1.04 (0.98-1.10)	0.19	-0.067	0.93 (0.83-1.05)	0.26	0.37
	IL-17C	46	0.015	1.01 (0.98-1.05)	0.36	46.96	0.39	0.04	0.042	1.04 (0.99-1.10)	0.13	-0.005	0.21	0.053	1.05 (1.00-1.11)	0.03	0.051	1.05 (0.99-1.11)	0.09	0.016	1.02 (0.92-1.13)	0.76	0.23
	IL-18	45	-0.003	1.00 (0.96-1.03)	0.86	64.98	0.02	0.32	0.015	1.02 (0.96-1.07)	0.56	0.000	0.91	0.024	1.02 (0.98-1.07)	0.32	0.027	1.03 (0.98-1.08)	0.30	0.040	1.04 (0.96-1.13)	0.35	0.82
	IL-18R1	46	-0.005	1.00 (0.97-1.02)	0.73	53.58	0.18	0.16	-0.010	0.99 (0.93-1.05)	0.75	-0.001	0.91	-0.023	0.98 (0.93-1.03)	0.37	-0.024	0.98 (0.93-1.03)	0.39	-0.014	0.99 (0.90-1.08)	0.78	0.10
	IL-1α	45	0.008	1.01 (0.98-1.04)	0.61	36.31	0.79	-0.21	-0.007	0.99 (0.94-1.05)	0.81	-0.001	0.82	0.012	1.01 (0.96-1.07)	0.66	0.020	1.02 (0.96-1.08)	0.51	0.012	1.01 (0.91-1.12)	0.82	0.22
	IL-2	46	0.008	1.01 (0.98-1.04)	0.62	45.95	0.43	0.02	0.028	1.03 (0.97-1.09)	0.34	-0.003	0.48	-0.007	0.99 (0.94-1.04)	0.78	0.000	1.00 (0.95-1.06)	1.00	0.014	1.01 (0.92-1.11)	0.77	0.17
	IL-20	46	-0.001	1.00 (0.97-1.03)	0.96	28.94	0.97	-0.55	-0.002	1.00 (0.95-1.05)	0.95	0.002	0.56	0.018	1.02 (0.97-1.07)	0.48	0.006	1.01 (0.94-1.08)	0.86	-0.056	0.95 (0.85-1.06)	0.34	0.70
	IL-20Rα	46	0.021	1.02 (0.99-1.05)	0.19	54.90	0.15	0.18	-0.034	0.97 (0.91-1.03)	0.28	0.005	0.32	-0.003	1.00 (0.94-1.05)	0.91	-0.047	0.95 (0.87-1.05)	0.32	-0.027	0.97 (0.87-1.08)	0.62	0.05
	IL-22Rα1	46	0.013	1.01 (0.98-1.05)	0.43	43.42	0.54	-0.04	-0.003	1.00 (0.95-1.05)	0.91	0.002	0.60	0.006	1.01 (0.96-1.06)	0.81	0.011	1.01 (0.96-1.07)	0.69	0.044	1.04 (0.96-1.14)	0.33	0.81
	IL-24	46	-0.009	0.99 (0.96-1.02)	0.56	59.85	0.07	0.25	0.001	1.00 (0.95-1.06)	0.96	0.001	0.75	-0.002	1.00 (0.95-1.05)	0.95	-0.016	0.98 (0.93-1.04)	0.59	-0.042	0.96 (0.87-1.06)	0.42	0.45
	IL-2Rβ	45	-0.018	0.98 (0.95-1.01)	0.26	48.74	0.29	0.10	-0.012	0.99 (0.94-1.03)	0.60	0.001	0.76	0.001	1.00 (0.96-1.05)	0.96	0.004	1.00 (0.96-1.05)	0.87	-0.037	0.96 (0.89-1.04)	0.34	0.90
	IL-33	46	-0.012	0.99 (0.96-1.02)	0.43	58.36	0.09	0.23	-0.004	1.00 (0.94-1.05)	0.90	-0.001	0.91	-0.001	1.00 (0.94-1.06)	0.98	-0.011	0.99 (0.92-1.06)	0.76	0.017	1.02 (0.91-1.14)	0.77	0.15
	IL-4	46	0.032	1.03 (1.00-1.07)	0.05	54.82	0.15	0.18	-0.022	0.98 (0.93-1.03)	0.39	0.004	0.39	-0.017	0.98 (0.94-1.03)	0.50	-0.029	0.97 (0.93-1.02)	0.22	-0.042	0.96 (0.88-1.05)	0.35	0.17
	IL-5	46	-0.006	0.99 (0.96-1.03)	0.71	52.93	0.19	0.15	-0.036	0.97 (0.91-1.02)	0.23	0.008	0.09	-0.014	0.99 (0.94-1.04)	0.59	-0.040	0.96 (0.91-1.01)	0.14	0.026	1.03 (0.92-1.14)	0.63	0.10
	IL-6	46	0.000	1.00 (0.97-1.03)	0.99	28.94	0.97	0.56	0.000	1.00 (0.96-1.05)	0.98	0.000	0.97	-0.0044	1.00 (0.95-1.04)	0.85	-6.87E-05	1.00 (0.96-1.04)	1.00	0.0341	1.03 (0.96-1.11)	0.35	0.98
	IL-7	46	-0.007	0.99 (0.97-1.02)	0.65	33.58	0.89	-0.34	-0.010	0.99 (0.94-1.05)	0.73	0.001	0.76	0.012	1.01 (0.96-1.06)	0.64	-0.010	0.99 (0.94-1.05)	0.72	-0.066	0.94 (0.84-1.04)	0.22	0.00
	IL-8	45	0.001	1.00 (0.97-1.03)	0.96	62.65	0.03	0.30	-0.014	0.99 (0.94-1.03)	0.57	0.002	0.53	-0.023	0.98 (0.93-1.02)	0.32	-0.030	0.97 (0.92-1.02)	0.23	-0.017	0.98 (0.91-1.07)	0.68	0.26

Supplementary Table 4. Associations of Hashimoto's Thyroiditis with inflammatory proteins from IEU Open GWAS using Mendelian randomization (with SNPs reaching *P*< 5×10^{-6})

Category	Exposures	n SNPs	IVW						MR-Egger					Weighted Median			Weighted Mode			Simple Mode			MR-PRESSO
			Beta	OR(95% CI)	P	Q	P_Q	I^2	Beta	95% CI	P	intercept	$P_{intercept}$	Beta	95% CI	P	Beta	95% CI	P	Beta	95% CI	P	$P_{Global\ Test}$
	IL-12p40	41	0.022	1.02 (0.99-1.05)	0.14	53.63	0.07	0.25	-0.024	0.98 (0.93-1.02)	0.34	0.005	0.20	-0.017	0.98 (0.94-1.03)	0.46	-0.025	0.98 (0.93-1.03)	0.34	0.025	1.03 (0.94-1.12)	0.59	0.17
	INF-γ	46	0.011	1.01 (0.98-1.04)	0.47	53.05	0.19	0.15	0.014	1.01 (0.96-1.07)	0.60	-0.003	0.48	-0.014	0.99 (0.94-1.03)	0.54	-0.017	0.98 (0.93-1.04)	0.54	-0.020	0.98 (0.90-1.07)	0.66	0.97
	LAP TGF-β1	46	-0.002	1.00 (0.97-1.03)	0.89	50.33	0.27	0.11	0.026	1.03 (0.97-1.08)	0.35	-0.005	0.25	-0.006	0.99 (0.95-1.04)	0.81	-0.004	1.00 (0.96-1.04)	0.84	-0.002	1.00 (0.92-1.08)	0.96	0.01
	LIF	46	0.010	1.01 (0.98-1.04)	0.52	39.16	0.72	-0.15	0.025	1.03 (0.97-1.08)	0.35	-0.002	0.56	0.007	1.01 (0.96-1.06)	0.78	0.005	1.00 (0.95-1.06)	0.87	0.078	1.08 (0.99-1.18)	0.09	0.56
	LIFR	46	0.014	1.01 (0.98-1.05)	0.36	50.85	0.25	0.12	0.034	1.03 (0.97-1.11)	0.33	-0.007	0.23	-0.016	0.98 (0.93-1.04)	0.57	0.021	1.02 (0.95-1.10)	0.57	0.044	1.04 (0.91-1.20)	0.54	0.00
**Tumor Nerosis Factor**																							
	TNF	46	0.027	1.03 (1.00-1.06)	0.09	36.87	0.80	-0.22	-0.012	0.99 (0.94-1.04)	0.65	0.008	0.09	-0.001	1.00 (0.95-1.05)	0.97	0.004	1.00 (0.95-1.06)	0.88	0.029	1.03 (0.92-1.15)	0.62	0.06
	TNFRSF9	45	0.034	1.03 (1.00-1.07)	0.04	60.19	0.05	0.27	0.038	1.04 (0.99-1.09)	0.16	-0.002	0.61	0.037	1.04 (0.99-1.09)	0.14	0.045	1.05 (0.99-1.10)	0.11	0.020	1.02 (0.94-1.11)	0.65	0.82
	TNFSF12	46	0.015	1.02 (0.99-1.04)	0.29	38.32	0.75	-0.17	-0.013	0.99 (0.93-1.05)	0.67	0.010	0.05	-0.012	0.99 (0.94-1.04)	0.66	-0.039	0.96 (0.91-1.02)	0.21	0.045	1.05 (0.93-1.18)	0.47	0.04
	TNFSF14	46	-0.010	0.99 (0.95-1.03)	0.60	67.38	0.02	0.33	-0.007	0.99 (0.95-1.04)	0.75	0.005	0.22	0.017	1.02 (0.97-1.06)	0.43	0.010	1.01 (0.96-1.06)	0.69	-0.022	0.98 (0.90-1.06)	0.61	0.76
	TNF-β	44	-0.005	1.00 (0.96-1.03)	0.78	58.17	0.06	0.26	-0.047	0.95 (0.90-1.02)	0.15	0.007	0.16	0.000	1.00 (0.95-1.05)	0.99	-0.001	1.00 (0.93-1.07)	0.98	-0.003	1.00 (0.89-1.11)	0.95	0.02
	TRAIL	46	-0.003	1.00 (0.97-1.03)	0.84	34.46	0.87	-0.31	-0.017	0.98 (0.92-1.04)	0.58	0.003	0.61	-0.027	0.97 (0.92-1.03)	0.37	-0.020	0.98 (0.92-1.05)	0.56	0.003	1.00 (0.90-1.12)	0.95	0.06
	TRANCE	45	0.023	1.02 (0.99-1.06)	0.19	62.03	0.04	0.29	-0.002	1.00 (0.95-1.04)	0.92	0.000	0.98	0.003	1.00 (0.96-1.05)	0.89	-0.011	0.99 (0.95-1.04)	0.65	-0.037	0.96 (0.89-1.04)	0.35	0.89
	TSLP	46	0.013	1.01 (0.98-1.05)	0.42	36.70	0.81	-0.23	0.001	1.00 (0.95-1.06)	0.96	0.004	0.32	0.005	1.01 (0.96-1.05)	0.83	0.007	1.01 (0.96-1.05)	0.77	-0.039	0.96 (0.88-1.05)	0.37	0.01
**Others**																							
	ADA	46	-0.004	1.00 (0.97-1.02)	0.75	44.71	0.48	-0.01	-0.059	0.94 (0.90-0.99)	0.02	0.007	0.12	-0.054	0.95 (0.91-0.99)	0.02	-0.065	0.94 (0.89-0.98)	0.01	-0.061	0.94 (0.85-1.04)	0.24	0.11
	AXIN1	46	-0.016	0.98 (0.95-1.02)	0.33	39.46	0.70	-0.14	-0.006	0.99 (0.95-1.04)	0.80	0.006	0.15	0.029	1.03 (0.98-1.08)	0.24	0.025	1.03 (0.97-1.09)	0.41	-0.024	0.98 (0.89-1.07)	0.62	0.31
	Caspase 8	46	-0.003	1.00 (0.97-1.02)	0.81	54.36	0.16	0.17	-0.039	0.96 (0.92-1.01)	0.10	0.004	0.32	-0.035	0.97 (0.92-1.01)	0.13	-0.030	0.97 (0.93-1.02)	0.21	0.060	1.06 (0.97-1.16)	0.20	0.80
	CD40LR	46	-0.002	1.00 (0.97-1.03)	0.88	50.13	0.28	0.10	-0.053	0.95 (0.90-1.00)	0.05	0.007	0.12	-0.017	0.98 (0.93-1.04)	0.52	-0.006	0.99 (0.93-1.06)	0.86	0.011	1.01 (0.91-1.12)	0.83	0.54
	CD5	44	-0.005	1.00 (0.96-1.03)	0.79	62.45	0.03	0.31	0.020	1.02 (0.97-1.07)	0.42	-0.001	0.86	0.043	1.04 (1.00-1.09)	0.05	0.037	1.04 (0.98-1.10)	0.19	0.037	1.04 (0.94-1.14)	0.44	0.32
	CD6	45	-0.001	1.00 (0.96-1.03)	0.95	66.66	0.02	0.34	-0.032	0.97 (0.93-1.01)	0.17	0.003	0.36	-0.027	0.97 (0.93-1.02)	0.24	-0.015	0.99 (0.94-1.03)	0.53	0.007	1.01 (0.93-1.09)	0.86	0.95
	CDCP1	45	-0.006	0.99 (0.96-1.03)	0.76	75.47	0.00	0.42	0.002	1.00 (0.96-1.05)	0.95	0.003	0.46	0.010	1.01 (0.96-1.06)	0.67	0.011	1.01 (0.96-1.06)	0.66	0.043	1.04 (0.96-1.14)	0.34	0.86
	CST5	46	0.007	1.01 (0.98-1.03)	0.62	42.62	0.57	-0.06	-0.046	0.96 (0.91-1.01)	0.09	0.006	0.15	-0.022	0.98 (0.93-1.03)	0.42	0.003	1.00 (0.94-1.07)	0.93	0.027	1.03 (0.93-1.14)	0.61	0.70
	DNER	45	0.007	1.01 (0.98-1.04)	0.60	51.50	0.20	0.15	0.017	1.02 (0.96-1.07)	0.54	-0.001	0.82	0.008	1.01 (0.96-1.06)	0.74	-0.003	1.00 (0.95-1.05)	0.90	0.009	1.01 (0.92-1.10)	0.85	0.07
	EIF4E-BP1	46	0.015	1.02 (0.99-1.04)	0.30	35.33	0.85	-0.27	-0.003	1.00 (0.95-1.05)	0.90	-0.002	0.65	-0.010	0.99 (0.94-1.04)	0.69	-0.005	1.00 (0.95-1.05)	0.86	0.032	1.03 (0.94-1.13)	0.48	0.31
	Flt3L	46	-0.009	0.99 (0.96-1.02)	0.50	48.24	0.34	0.07	-0.041	0.96 (0.92-1.01)	0.10	0.006	0.11	-0.026	0.97 (0.93-1.02)	0.26	-0.029	0.97 (0.92-1.02)	0.27	-0.019	0.98 (0.89-1.08)	0.69	0.23
	MMP-1	44	0.007	1.01 (0.98-1.04)	0.62	42.98	0.47	0.00	-0.016	0.98 (0.94-1.03)	0.51	0.001	0.74	-0.017	0.98 (0.94-1.03)	0.47	-0.017	0.98 (0.94-1.03)	0.47	-0.018	0.98 (0.91-1.06)	0.63	0.37
	MMP-10	46	0.012	1.01 (0.98-1.04)	0.41	60.16	0.06	0.25	0.005	1.01 (0.96-1.05)	0.83	0.000	0.91	0.007	1.01 (0.96-1.05)	0.76	0.020	1.02 (0.97-1.07)	0.41	0.036	1.04 (0.95-1.13)	0.42	0.05
	NKR2B4	45	-0.010	0.99 (0.96-1.02)	0.50	50.70	0.23	0.13	-0.023	0.98 (0.94-1.02)	0.31	0.006	0.09	-0.014	0.99 (0.95-1.03)	0.49	-0.012	0.99 (0.95-1.03)	0.58	0.014	1.01 (0.95-1.09)	0.70	0.59

Supplementary Table 4. Associations of Hashimoto's Thyroiditis with inflammatory proteins from IEU Open GWAS using Mendelian randomization (with SNPs reaching $P < 5\times10^{-6}$)

Category	Exposures	n SNPs	IVW						MR-Egger					Weighted Median			Weighted Mode			Simple Mode			MR-PRESSO
			Beta	OR(95% CI)	P	Q	P_Q	I^2	Beta	95% CI	P	intercept	$P_{intercept}$	Beta	95% CI	P	Beta	95% CI	P	Beta	95% CI	P	$P_{Global\ Test}$
	OPG	46	-0.008	0.99 (0.96-1.03)	0.65	80.57	0.00	0.44	0.032	1.03 (0.99-1.08)	0.17	-0.005	0.17	0.019	1.02 (0.98-1.06)	0.38	0.008	1.01 (0.97-1.05)	0.73	0.000	1.00 (0.92-1.08)	0.99	0.02
	OSM	45	-0.002	1.00 (0.97-1.03)	0.87	54.79	0.13	0.20	-0.008	0.99 (0.94-1.05)	0.78	0.000	0.99	0.012	1.01 (0.97-1.06)	0.59	-0.005	1.00 (0.95-1.05)	0.85	-0.015	0.99 (0.90-1.08)	0.76	0.00
	PD-L1	45	-0.012	0.99 (0.96-1.02)	0.42	48.93	0.28	0.10	-0.007	0.99 (0.95-1.04)	0.75	0.001	0.87	0.015	1.01 (0.97-1.06)	0.52	0.013	1.01 (0.97-1.06)	0.59	-0.007	0.99 (0.90-1.09)	0.88	0.49
	S100A12	46	0.016	1.02 (0.99-1.05)	0.25	48.34	0.34	0.07	-0.011	0.99 (0.93-1.05)	0.70	0.001	0.80	-0.022	0.98 (0.94-1.02)	0.33	-0.023	0.98 (0.94-1.02)	0.28	-0.018	0.98 (0.91-1.06)	0.65	0.00
	SIRT2	46	-0.021	0.98 (0.95-1.01)	0.14	37.08	0.79	-0.21	-0.030	0.97 (0.92-1.02)	0.27	0.005	0.22	-0.034	0.97 (0.92-1.02)	0.22	-0.039	0.96 (0.91-1.02)	0.17	-0.047	0.95 (0.85-1.07)	0.41	0.02
	SLAM	45	-0.028	0.97 (0.95-1.00)	0.06	55.78	0.11	0.21	-0.014	0.99 (0.94-1.04)	0.58	0.003	0.55	0.001	1.00 (0.95-1.05)	0.98	0.004	1.00 (0.96-1.06)	0.88	0.004	1.00 (0.91-1.11)	0.94	0.15
	STAMBP	46	-0.015	0.98 (0.96-1.01)	0.28	30.54	0.95	-0.47	-0.018	0.98 (0.94-1.03)	0.44	0.003	0.38	-0.003	1.00 (0.95-1.05)	0.90	-0.006	0.99 (0.95-1.04)	0.81	-0.015	0.99 (0.90-1.08)	0.75	0.26
	SULT1A1	46	-0.020	0.98 (0.95-1.01)	0.22	43.60	0.53	-0.03	-0.027	0.97 (0.92-1.03)	0.34	0.005	0.24	-0.032	0.97 (0.92-1.01)	0.18	-0.042	0.96 (0.92-1.00)	0.08	-0.046	0.96 (0.86-1.06)	0.39	0.02
	uPA	45	0.021	1.02 (0.99-1.05)	0.14	48.06	0.31	0.08	-0.006	0.99 (0.95-1.04)	0.82	0.001	0.90	0.009	1.01 (0.96-1.06)	0.71	0.012	1.01 (0.97-1.06)	0.62	-0.030	0.97 (0.88-1.07)	0.56	0.16

Abbreviations: CI, Confidence interval; pval, p-value; Q, Cochran Q statistics; SNPs, single nucleotide polymorphisms. Beta and 95% CI represent change in SD of Inflammatory proteins per log odds increase in Hashimoto's Thyroiditis
